# Using custom-built primers and nanopore sequencing to evaluate CO-utilizer bacterial and archaeal populations linked to bioH_2_ production

**DOI:** 10.1038/s41598-023-44357-3

**Published:** 2023-10-09

**Authors:** İlayda Akaçin, Şeymanur Ersoy, Osman Doluca, Mine Güngörmüşler

**Affiliations:** 1 Division of Bioengineering, Graduate School, Izmir University of Economics, Sakarya Caddesi No: 156, 35330 Balçova, Izmir Türkiye; 2 Department of Biomedical Engineering, Faculty of Engineering, Izmir University of Economics, Sakarya Caddesi No: 156, 35330 Balçova, Izmir Türkiye; 3 Department of Genetics and Bioengineering, Faculty of Engineering, Izmir University of Economics, Sakarya Caddesi No: 156, 35330 Balçova, Izmir Türkiye

**Keywords:** Next-generation sequencing, Environmental biotechnology

## Abstract

The microbial community composition of five distinct thermophilic hot springs was effectively described in this work, using broad-coverage nanopore sequencing (ONT MinION sequencer). By examining environmental samples from the same source, but from locations with different temperatures, bioinformatic analysis revealed dramatic changes in microbial diversity and archaeal abundance. More specifically, no archaeal presence was reported with universal bacterial primers, whereas a significant archaea presence and also a wider variety of bacterial species were reported. These results revealed the significance of primer preference for microbiomes in extreme environments. Bioinformatic analysis was performed by aligning the reads to 16S microbial databases for identification using three different alignment methods, Epi2Me (Fastq 16S workflow), Kraken, and an in-house BLAST tool, including comparison at the genus and species levels. As a result, this approach to data analysis had a significant impact on the genera identified, and thus, it is recommended that use of multiple analysis tools to support findings on taxonomic identification using the 16S region until more precise bioinformatics tools become available. This study presents the first compilation of the ONT-based inventory of the hydrogen producers in the designated hot springs in Türkiye.

## Introduction

Many thermophiles have been found in hot springs, where geothermally heated groundwater is discharged from fissures in the Earth’s crust. Since the first phylogenetic characterization of an environmental microbial population by 16S rDNA sequencing at Yellowstone National Park, USA^1^, a steadily increasing number of studies have been conducted on metagenomics of extreme environments^2–7^. According to the scientists conducting these investigations, common bacterial primers designed from the genomic sequences of laboratory-grown bacteria are insufficient to represent and distinguish uncultured prokaryotic isolates from environmental samples taken under challenging circumstances, which are heavily influenced by seasonal fluctuations. In this study, comparative genomic data sets from the scientific literature were carefully examined in order to design effective primer sets for the investigation of the target microbial communities in extreme conditions. A wide range of bacteria remains unculturable under lab conditions, thus, their sensitive detection within their habitats, where they are already found in low abundances and in dormant states, rely on primers whose design is based on up-to-date and comprehensive 16S databases. Metagenomics is a cutting-edge technique for screening bacterial and archaeal species independent of cultures and serves as a crucial tool for research on the variety of microbial communities in extreme environments^8,9^. This tool allows the detection of all genes present in an organism, and their structures, functions, distribution and diversity in the context of their community by simultaneously sequencing, or arraying, genetic fragments obtained from the complete microbiome^10–12^. Natural water sources with extreme temperatures and their residences of thermophilic microorganisms remain highly unexplored, and unable to be cultivated in lab conditions. However, metagenomic investigations shed light into these wild microbial communities and help environmental microbiologists to better understand and uncover the great potential and diversity held in these rare-biospheres.

Sequencing technologies are mainly classified for their sequencing length in base pairs (bp), commonly used methods cover only a portion of the whole region (the V3–V4 region of the 16S rDNA gene^13^), however, the 16S region can be covered by all V1–V9 hypervariable regions in the newly developed long-read sequencing platforms allows for better taxonomical classification and functional profiling of uncultured organisms. Unlike many other methods, the Oxford Nanopore MinION technology (ONT) relies on direct detection and sequencing of a single-stranded DNA (ssDNA) molecule with characteristic electrical current changes of bases, rather than nucleotide incorporation^14,15^. DNA sequencing with nanopore sequencing distinguishes it from other next-generation sequencing (NGS) and third generation sequencing (TGS) platforms, as it enables on-field applications with rapid library preparation and real-time acquisition of genomic data, as well as cost-efficiency^16^. Considering these, nanopore sequencing has become an appealing tool for genomic characterization of complex environmental samples, improving the detection of unknown organisms, owing to the development of rapid protocols and analysis pipelines for metagenomic profiling^17^.

In this study, it was aimed to compare the capabilities of different pipelines in bioinformatics in nanopore studies by sequencing samples of thermal water sources, one of the habitats of extremophile microorganisms. We hypothesized that the microbial populations of five distinct hot springs in Izmir, Türkiye, may contain extreme thermophiles, such as carboxydotrophic hydrogenogenic profiles, which alter with the evolution of molecular and environmental growth conditions. In our study, using two separate 16S rDNA primers (universal and custom designed), we identified bacterial and archaeal communities associated with those that use carbon monoxide to produce hydrogen. In Türkiye, an inventory of hydrogen producers in the designated hot springs was first assembled using ONT technology.

## Results

### Metagenomic analysis of Doğanbey, Çeşme, and Bergama hot springs

Our data is presented using a sequential approach, which encompassed the steps of sample gathering and processing. Biohydrogen production capacities and SEM images are also presented to evaluate the sequencing results, and the practical outcomes.

Between the dates of 25/05/2021 and 23/08/2021, sampling of the microbial communities at the Izmir hot spring ecosystem revealed variations in temperature and ORP values across the sample location. For the Doğanbey hot spring, the following were observed: Upstream (US) 77.3 °C; 43.8 mV, midstream (MS) 52 °C; − 13.2 mV and downstream (DS) 49.2 °C; − 23.1 mV, with a slight change in pH values between 6.42 (US) to 7.43 (DS). On the other hand, Çeşme and Dikili-Bademli hot springs reported mesophilic conditions (38 °C; pH 7.2 and 43.2 °C; pH 6.75) whereas Dikili-Nebiler and Bergama hot springs reported thermophilic conditions (56.2 °C; pH 7.88 and 56.2 °C; pH 6.86).

According to taxonomic classification data obtained from all hot springs, bacteria (95%) with 6,376,218 reads, and archaea with 85,786 reads (1%) were classified as superkingdom in the taxonomic classification.

**Figure 1:**
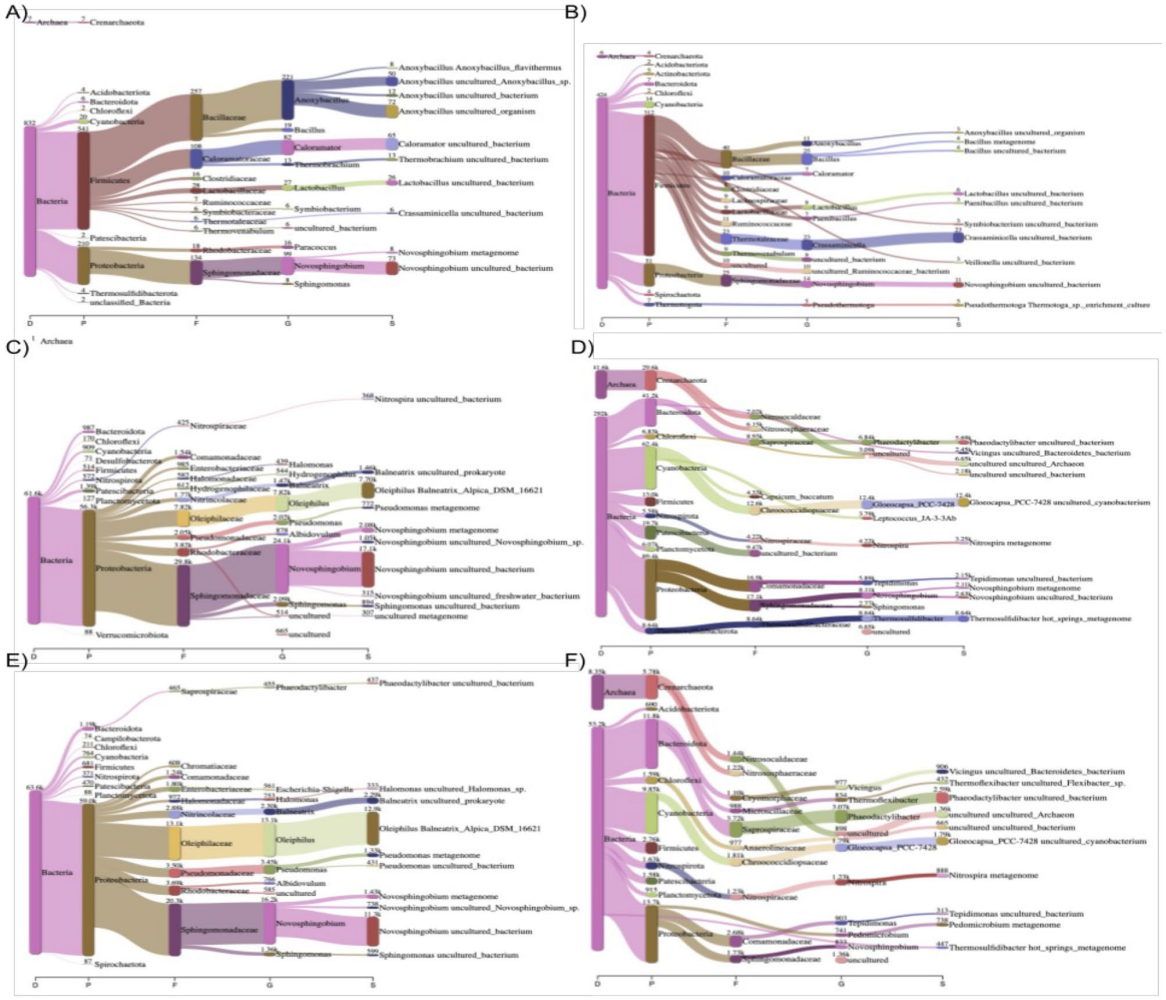
Representation of species level classification comparing archaeal distribution among samples collected from 3 different parts of the Doğanbey hot spring and amplified with two different 16S rDNA primers (universal and custom-built) using Kraken tool as indicated in the (Supplementary Information) including the Python codes and BLAST data. Samples were collected from upstream (**A,B**), midstream (**C,D**) and downstream (**E,F**) regions, and analyzed using universal (**A,C,E**) and archaea (**B,D,F**) primers.

**Figure 2:**
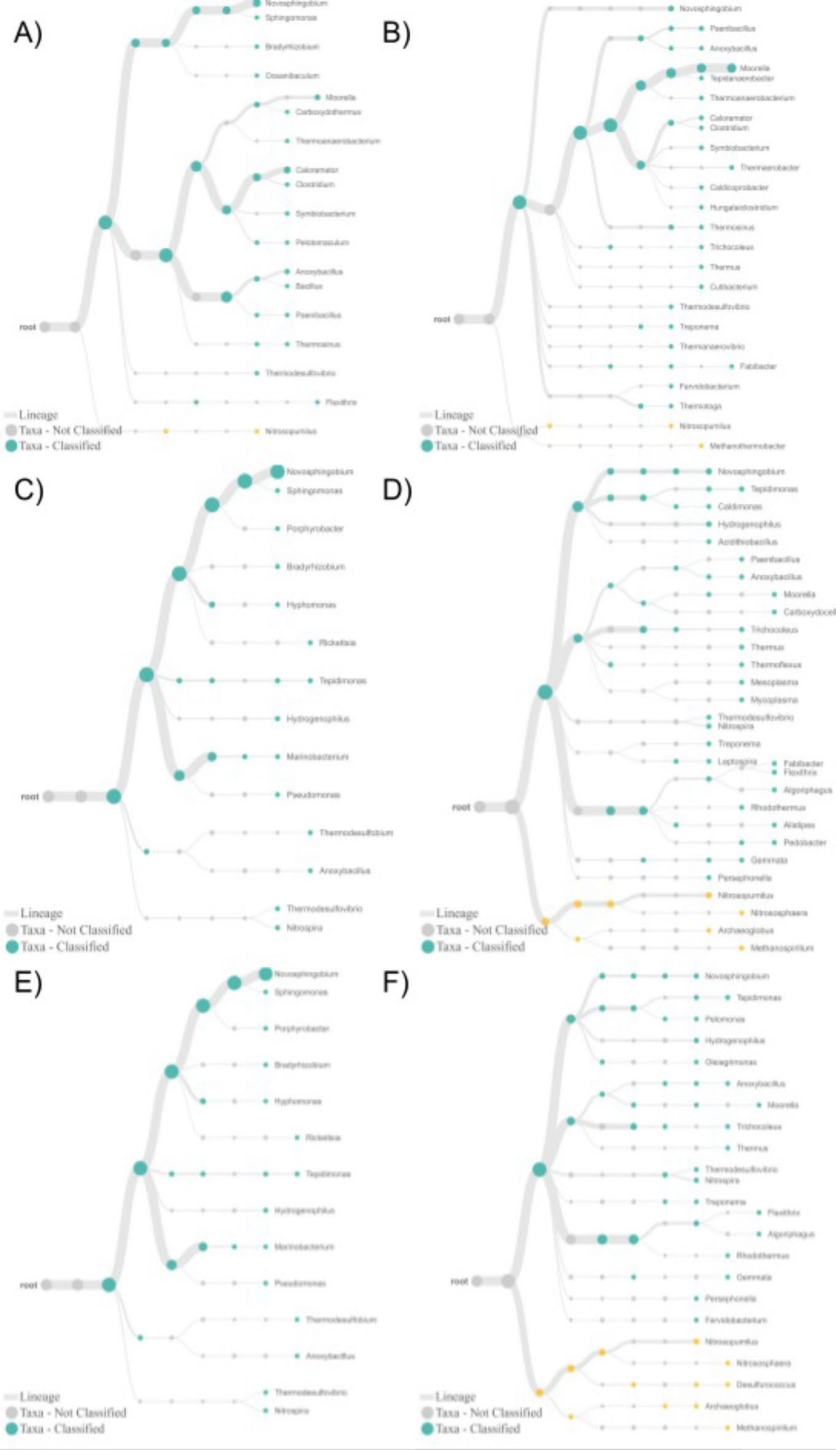
Representation of species level classification of samples collected from Doğanbey hot spring using the Epi2Me tool. Samples were collected from upstream (**A,B**), midstream (**C,D**) and downstream (**E,F**) regions, and analyzed using universal (**A,C,E**) and archaea (**B,D,F**) primers.

**Figure 3:**
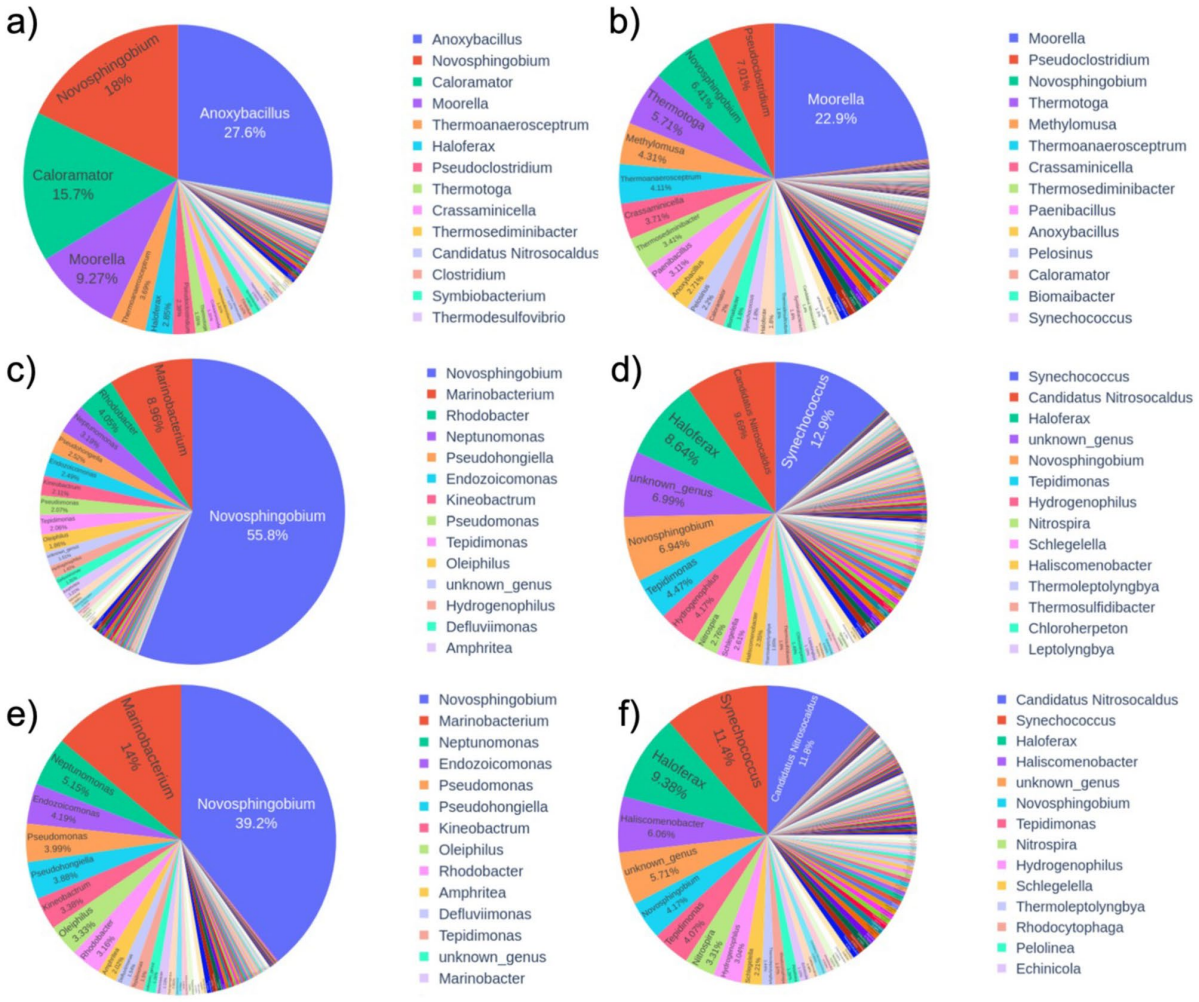
Representation of genus level classification of samples collected from Doğanbey hot spring using the in-house BLAST. Samples were collected from upstream (**a,b**), midstream (**c,d**) and downstream (**e,f**) regions, and analyzed using universal (**a,c,e**) and archaea (**b,d,f**) primers.

**Figure 4:**
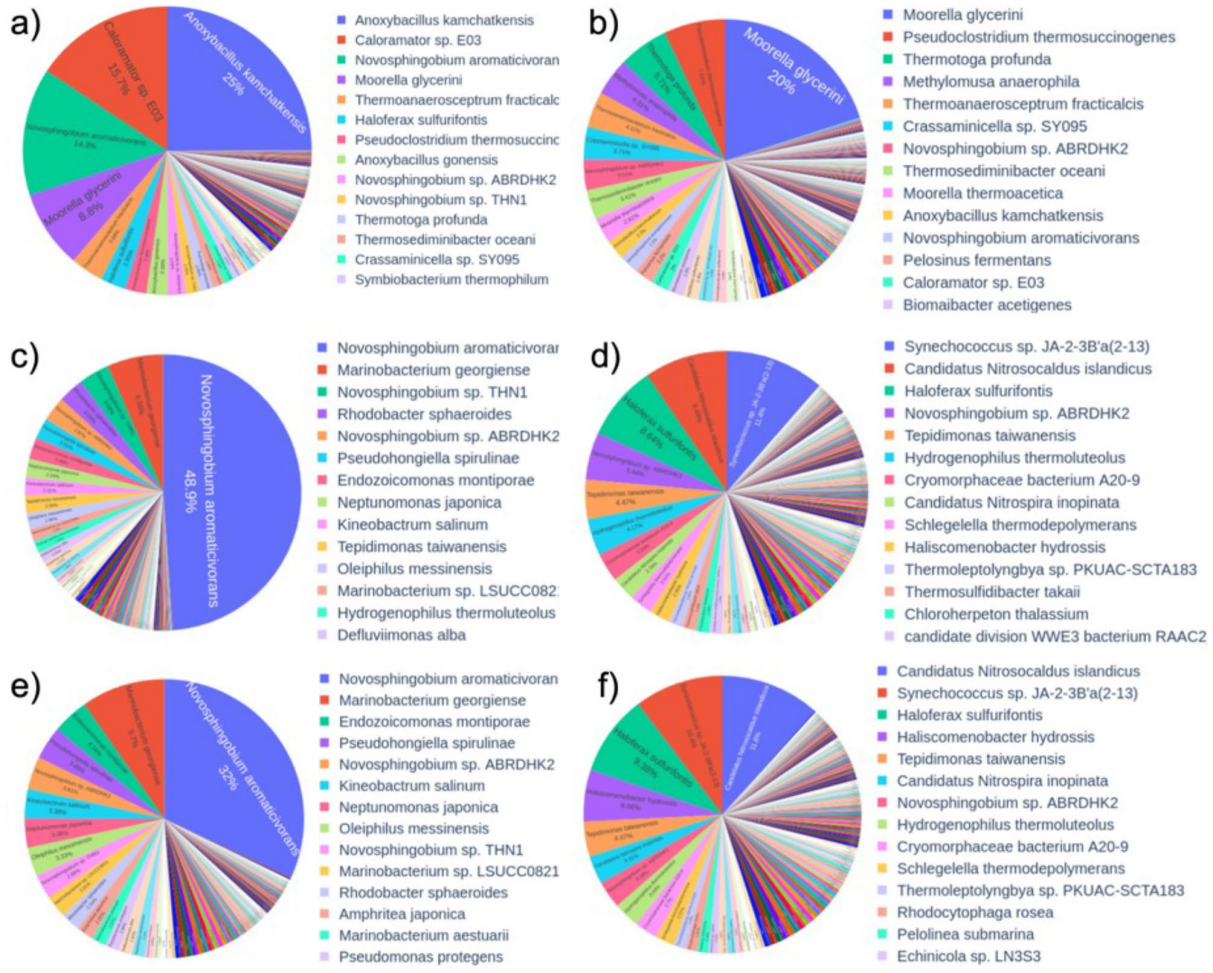
Representation of species level classification of samples collected from Doğanbey hot spring using the in-house BLAST. Samples were collected from upstream (**a,b**), midstream (**c,d**) and downstream (**e,f**) regions, and analyzed using universal (**a,c,e**) and archaea (**b,d,f**) primers.

Three different tools were used to compare the samples analyzed using archaea-specific primers. Overall, the analysis of Doğanbey samples indicated that the choice of primer pair has a significant impact on the range identified microorganisms and their abundances. Interestingly, when analyzed using Kraken (version number,citation), upstream samples showed no significant change with use of archaea primers at domain or phylum levels (Fig. 1). *Crassaminicella*, *Paenibacillus* and *Pseudothermotoga* at genus level were reported. Epi2Me (v3.6.2) tool also showed similar results. Using universal primers, *Nitrosopumilus* was the only archaeal genus detected at Doğanbey upstream, with no sign of archaea in the other locations. However, within all Doğanbey samples, the use of the archaea primers resulted in increased abundance of this genus, as well as the discovery of other archaea species. Newly detected archaea genera were *Methanothermobacter* for the upstream, and for the mid and downstream regions, *Nitrososphaera*, *Archaeoglobus*, *Methanospirillum* (Fig. 2). The in-house BLAST tool was used as an alternative for identification of genera (Figs. 3, 4). Using this tool obtained a similar microorganism profile as found by using Kraken and Epi2Me. In all samples analyzed using universal primers, reads were much more aligned to *Novoshingobium* species than in those analysed using archaea-specific primers. Moreover, using archaea-specific primers, the archaea genus *Haloferax*, rather than *Nitrososphaera* was identified as the dominant genus.

In-house BLAST classifications were compared to Kraken and Epi2me platforms, and indicated that these three different platforms were compatible, and result in very similar performances of bacteria as far as genus level classification. However, in species level classification, there are few differences, concerning most abundant species in the samples. For example, in Doğanbey downstream sample (E in Figs. 1, 4), the most abundant species, according to Kraken classification, is *Oleiphilus balneatrix*, and according to in-house BLAST classification, it is *Novosphingobium aromaticivorans.*

**Figure 5:**
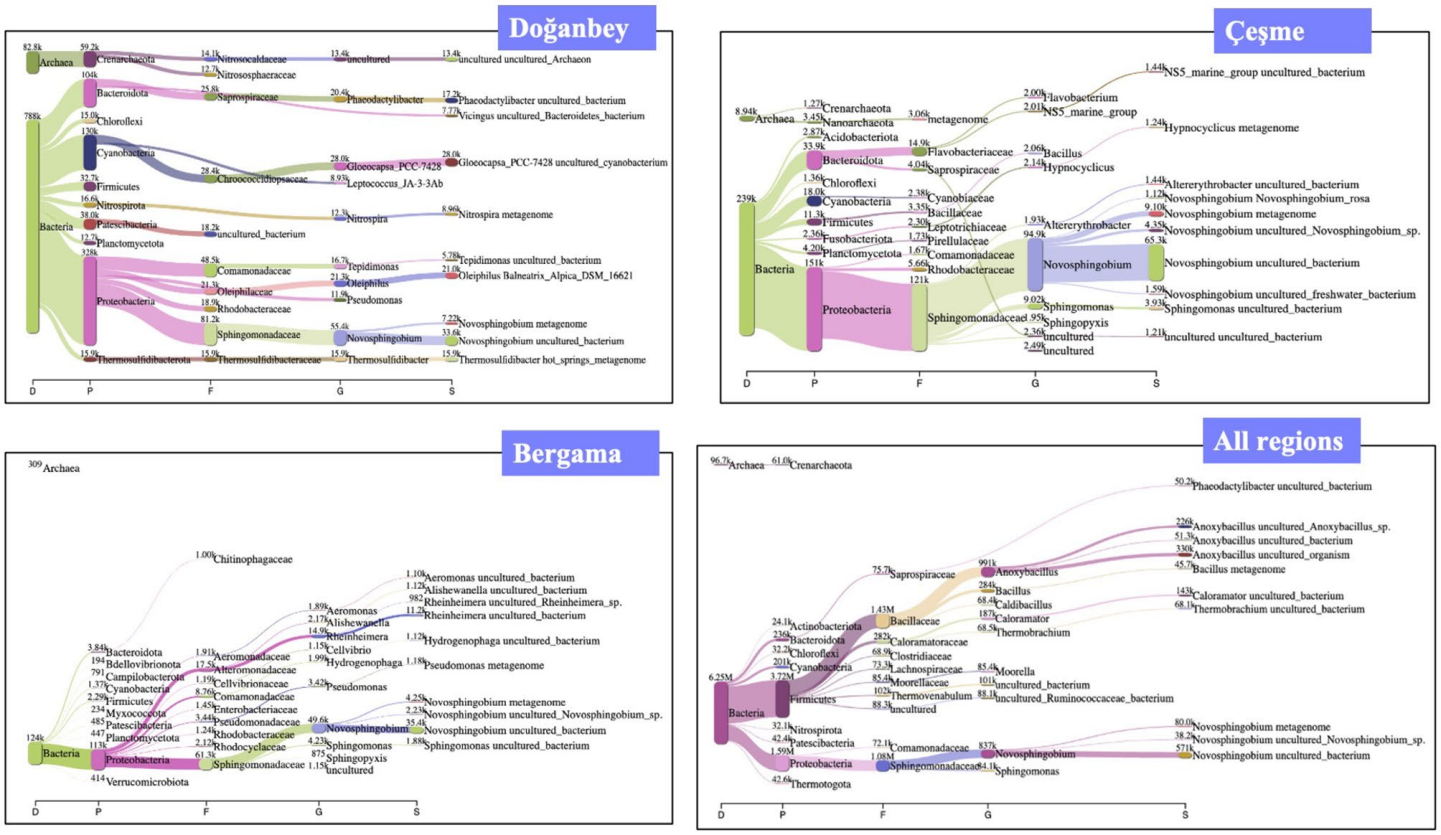
Metagenomic analysis and taxonomic classification of Doğanbey, Çeşme, Bergama hot springs and comparison of all regions in Izmir province using universal 16S rDNA bacterial primers (prepared using Kraken).

Based on the species obtained as a result of 16S rDNA metagenomic sequencing using universal primers depicted in Fig. 5, Doğanbey hot spring hosts dominantly *Novospingobium* species belonging in *Proteobacteria* genus, which is frequently encountered in thermal water resources^18^. *Novospingobium* sp., which live in milder temperature conditions (30–45 °C), also dominate the Çeşme hot spring community^19^. This was an expected finding considering the temperature of the hot spring was measured as 38–43 °C.

Among the 5 hot spring water communities, the archaea population was most evident in the Doğanbey thermal spring, from which more than 80 k reads were obtained. Archaea population in the hot spring was determined as a result of 16S analysis, and the three most common archaeal species were *Nitrosopumilus ureiphilus*, *Nitrosopumilus maritimus*, *Nitrososphaera viennensis*.

Only 4% of the reads could not be classified. *Novosphingobium* species capable of degrading aromatic compounds were found to be dominant (17%), but these bacterial species are moderately thermophilic (37–45 °C)^20^ and aerobic, and therefore not considered as one of the species of interest in terms of H_2_ production within the scope of the study.

Among the bacterial communities, *Moorella* (*Moorella humiferrea*, *Moorella thermoacetica*, *Moorella gylcerini*), a gram-positive thermophilic and anaerobic bacterium, was reported as the second most dominant genus after *Novospingobium* species (15%). Thermophilic, anaerobic and gram positive *Anoxybacillus* species and gram positive, facultative anaerobic and moderately thermophilic Caloramator species with 3% population density were determined. *Moorella, Anoxybacillus* and *Caloramator* genera, which dominate the community, are species with spore-forming properties, which have already been isolated from hot springs^21–23^.

**Figure 6:**
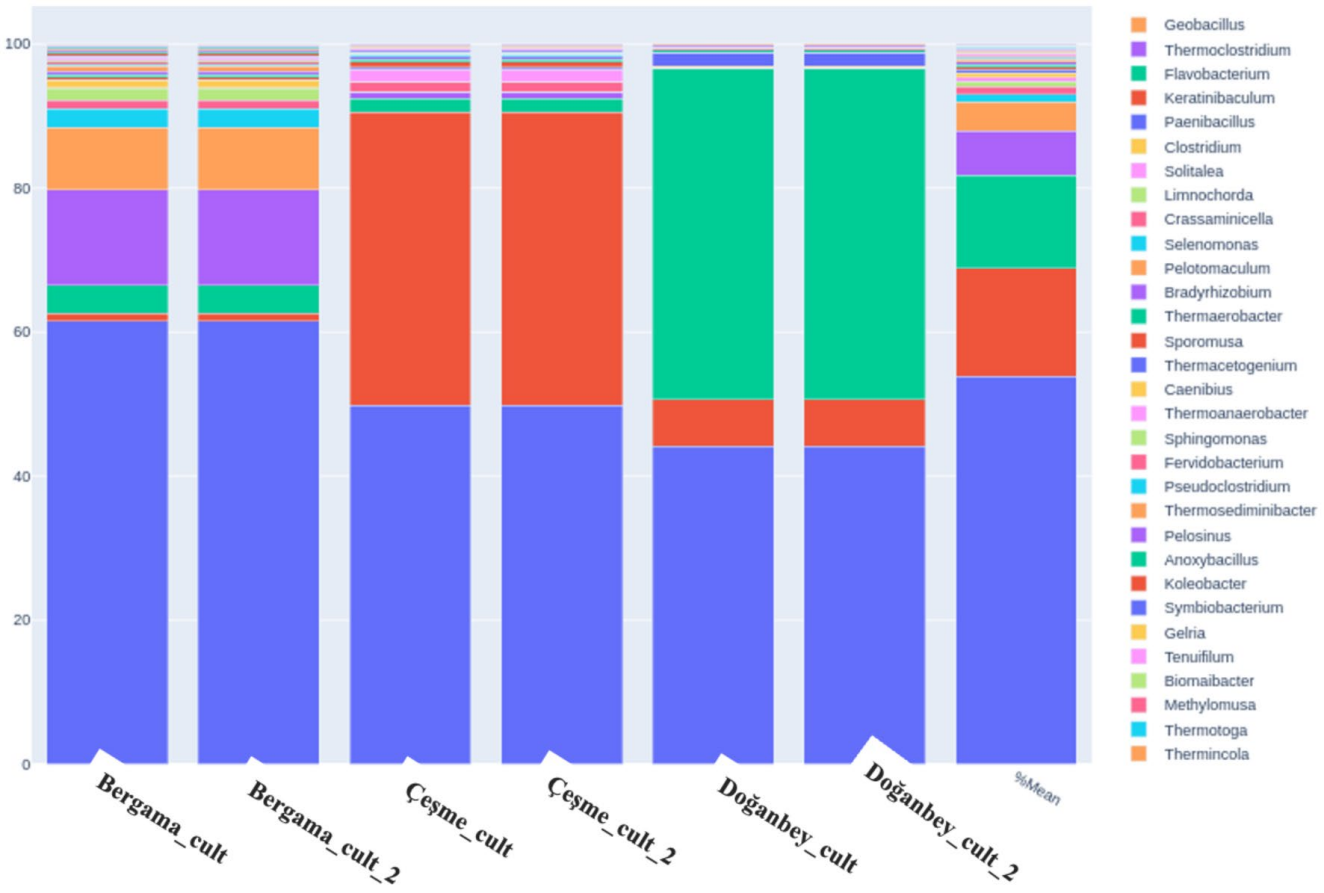
Percentage representation of microbial community diversity and quantity plots of samples at genus level bacteria and archaea in Bergama, Çeşme, and Doğanbey hot springs from enriched cultures.

Figure 6 demonstrates the diversity of the bacterial and archaeal specimens at the genus level, highlighting the variation of distinct organisms in three different hot springs (Bergama, Çeşme, Doğanbey) using both isolated directly from the spring water and cultivated isolates amplified with universal bacterial 16S rDNA primers.

Previously, reports were made of the presence of carboxydotrophic hydrogenogenic organisms in different hot springs of Izmir province: these were *Methanosarcina acetivorans*^24^*, Thermincola ferriacetica*^25^, *Moorella thermoacetica*^26^, *Thermosinus carboxydivorans* Nor1 and *Thermosinus carboxydivorans*^27–29^, *Carboxydothermus pertinax*^30^, *Carboxydocella ferrireducens* and *Carboxydocella thermautotrophica* and *Carboxydocella* spp.^31,32^, *Caldanaerobacter caldanaerobacter subterraneus and Caldanaerobacter thermoanaerobacter* sp.*_*RH0804^33–35^ with low abundances, with the most dominant being *M*. *stamsii* by % 0.254945 presence^26^. Consequently, in our study, the reported low abundances of these microorganisms were in agreement with the known rarity of carbon monoxide (CO)-oxidizing and H_2_-producing microorganisms in nature (< 0.1%), and these are usually found in dormant state, associated with little to zero growth^36–38^. In order to test their capacity to convert CO into H_2_, the enrichment of these CO-utilizing consortia was experimented under carboxydotrophic growth-specific media and CO gas as the substrate, suitable growth conditions were supplied for thermophilic hot spring microorganisms to cultivate under lab conditions, increase their abundances in the culture and produce hydrogen using necessary enzymes in their metabolisms.

A significant increase in the genus of *Moorella* was achieved, with their abundance reaching up to 5% in cultivated samples, *Caldanoerobacter* species up to 0.6% however *Carboxydocella* and *Carboxythermu*s genuses reached only 0.02% and 0.01% in abundance, respectively (Fig. 6). A comparative analysis of Doğanbey isolates cultured on different gas substrates showed a decrease in the number of species in the enriched mixed culture in lab conditions, and a significant difference between growth in syngas (see “Methods” section) and 100% CO. *Caloramator* sp., the species of greatest interest for hydrogen production in culture, is unable to grow in syngas-fed culture medium (less than 5% in abundance) and thus allowing the dominant growth of *Novosphingobium* (up to 60%), but grows dominantly in 100% CO gas-fed culture medium (up to 50%), with the remaining culture consortia consisting mainly of *Novosphingobium aromaticivorans* (38%) and *Moorella glycerini* (6%) (Fig. 6). Environmental and chemical growth conditions were adjusted for the thermophilic hot spring microorganisms to cultivate them under lab conditions. These conditions were applied in order to increase their abundances in the culture and simultaneously produce hydrogen with the help of the relevant enzymes in their metabolisms. Microorganisms were grown until hydrogen production was detected representing the end of their logarithmic phase as shown in our previous study^39^. The comparative results of taxonomic profiling in direct water isolations and in cultivated samples demonstrated that bacterial species belong to the Firmicutes genus *Anoxybacillus* and *Caloramator* alongside other thermophilic carboxydotrophic hydrogenogens (*Moorella stamsii, Tepidimicrobium ferriphilum, Thermodesulfovibrio, Geobacillus, Thermosediminibacter*)^40–42^ have been dominantly observed, and their abundances increased after being taken into anaerobic and thermophilic culture medium and feeding with 100% CO gas. Hydrogen production and CO utilizing activities of the cultivated samples were reported in our previous study^39^ and the results indicated that the presence of thermophiles together with anaerobes in the microbial community constructed an adequate mixed culture consortium for efficient hydrogen production.

### Scanning electron microscopy (SEM) imaging of cultivated hot spring isolates

**Figure 7:**
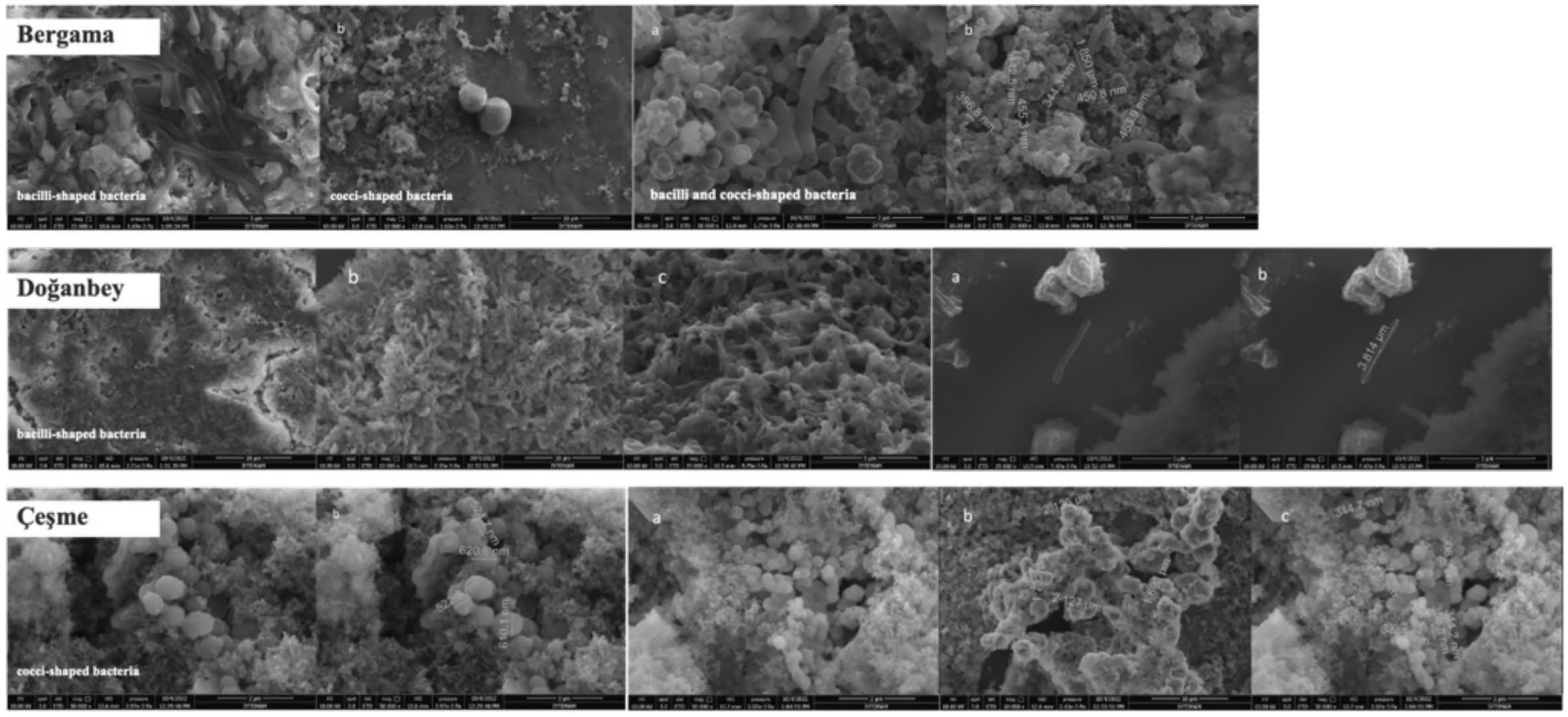
SEM images and dimensions of bacilli and cocci-shaped bacteria isolated from Bergama, Doğanbey, and Çeşme hot springs.

SEM imaging of 3 different H_2_-producing hot spring microbial communities showed that these communities consist of bacilli and cocci-shaped bacteria of various sizes and morphological features (Fig. 7). A dense population of a single bacilli-shaped bacteria 3.814 µm long and 362.8 nm wide was observed in Doğanbey. This single thermophilic and anaerobic microorganism showed significant morphological similarity to the genera of *Thermoanaerobacter*, *Carboxydocella*, and *Bacillus* associated with CO conversion to hydrogen and organic acids^43,44^. Çeşme samples exhibited mainly cocci-shaped bacterium with sizes ranging from 1.60 to 2.127 µm in length and 307.6 to 620.1 nm in width. In Bergama samples, both bacilli and cocci-shaped bacterium were observed simultaneously; the Bacilli-shaped bacteria were 1.850 µm in length and the cocci-shaped bacterium were 344.3 to 455.3 nm in width.

A recent publication by the authors reported the biohydrogen production capacities of the isolated microorganisms from the hot springs of Izmir province; the highest was found in Bergama, with a yield of 0.18 ΔH_2_ (mmol)/ΔCO (mmol), followed by Doğanbey (0.13 ΔH_2_ (mmol)/ΔCO (mmol)) and Çeşme (0.12 ΔH_2_ (mmol)/ΔCO (mmol))^39^.

## Discussion

The abundances of microorganisms were determined by nanopore sequencing, and the findings were compared in accordance with the comparative bioinformatic analysis methods, sampling sites, sequencing primer selections, and culturing techniques.

Following the quality check and barcoding, the reads were aligned to 16S microbial databases for identification using three different alignment tools, Epi2Me (Fastq 16S workflow), Kraken and in-house BLAST tool. A comparison of the differences at genus and species levels shows that there is agreement between these tools at genus level classification, but this agreement disappears at species level in all cases. While Kraken identifies *Oleiphilus* species among samples in mid- and low-course samples, the other tools would align these reads to *Marinobacterium*. It is important to note that both belong to the same *Oceanospirillales* order. On the other hand, unlike the other two tools, in-house BLAST showed a significant proportion of reads (up to 15%) aligning to *Haloferax sulfurifontis* in samples studied with Archaea-specific primers. Such differences in the three analysis tools we have employed suggests that the way the data is analyzed has a significant impact on the species level identification, however, shows a significant compatibility in identified genus of hot spring microbial communities. These differences in species level identifications might be due to the fact that the 16S region where classification is based, is too short a stretch of DNA, resulting in significant alignment score fluctuations by the algorithms, but it is also very likely to be due to the differences in the available databases they utilize. For that reason, until more precise tools are developed, use of more than one analysis tool is beneficial to support findings on species level taxonomic identification using the 16S region. Cumulative contribution of the scientific community to excel sequencing analysis of microbiota may eventually result in one of these or many other tools to be commonly accepted, however, it should be remembered that the methods and databases utilized by different tools may be more sensitive over the others in particular conditions or sample sources. For this reason, it may not be possible to claim any tool to be superior to others and the use of multiple tools will remain the optimal approach. The problem with such an approach is how to evaluate the disagreements. While different approaches may be utilized to overcome this, the choice is still a question of statistics and beyond the scope of this work.

Besides the choice of tool, there were significant differences between the results obtained using different primer pairs. Accordingly, the archaea-specific primers classified both archaea and bacteria, while universal bacterial primers showed fewer reads aligned to any archaeal species. It is crucial to note that to generate the archaea-specific primers, only archaea genomes were used to identify a consensus sequence, and primers were selected at highly conserved regions. Despite this, the bacterial sequences made up the greater portion of the reads, even with archaea-specific primers. This may indicate that, despite the PCR bias for archaeal species, the abundance of bacteria may be well above the archaea species in the sample and make up more than the abundance observed by the archaeal species. Supporting this, universal primers resulted in a marginal number of reads aligned to the archaea. Read numbers are a result of PCR amplification thus not represent the reads obtained directly from microorganisms and this PCR bias could always effect the abundance of microorganisms frequency. For this study to minimize the PCR bias we have implemented PCR with minimum cycles to obtain target DNA concentration.

Despite a general agreement between the studies at the genus level and above, there is no agreement at the species level. Even in pure culture samples, there are conflicts regarding the classification at the species level, for example the species of *Novosphingobium* and *Anoxybacillus* are detected and in every sample, but there is a distinct bias towards *Novosphingobium*. It is argued that microbial genomes from natural environments exhibit trait biases resulting from phylogeny-based approaches, and lacking whole-genome sequences of uncultured bacteria, thus the available reference genome datasets may exhibit biases towards more abundant organisms^45^. In pure cultures from Doğanbey hot spring, there is a bias towards this species in bioinformatic analyzes, especially in Epi2Me workflows. Considering that the *Novosphingobium* species live in moderately thermophilic environments^18,20^, this bias is explained by the frequent classification of this species (about 15%) in the Doğanbey hot spring samples, where water temperatures reach 75 °C, and where the growth of *Novosphingobium*, a mesophilic species, is usually very limited^20^.

**Figure 8:**
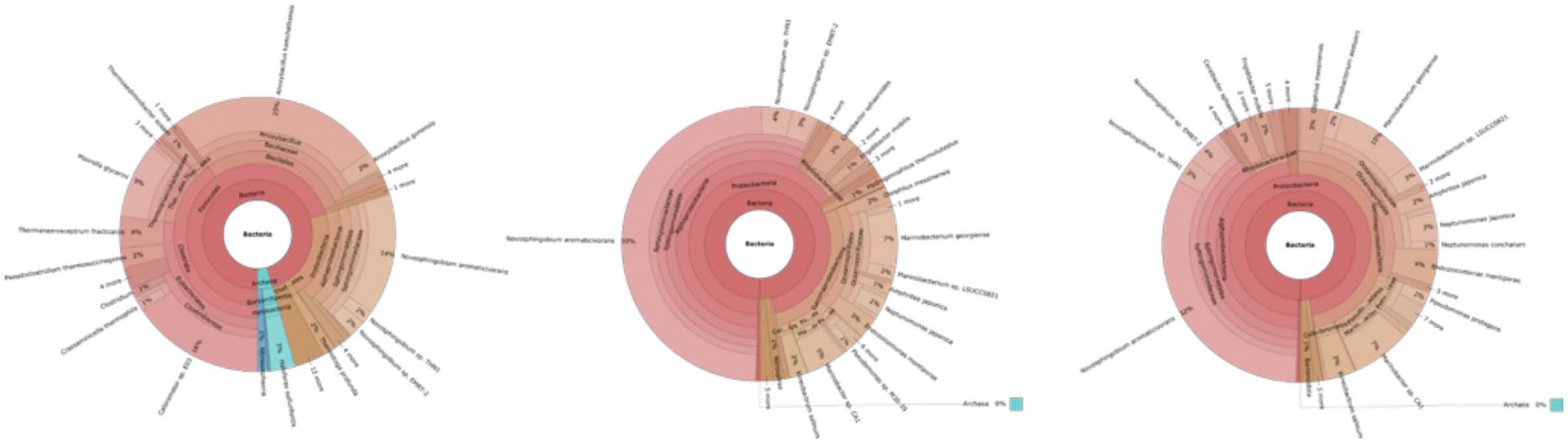
Krona plot analysis of three different points of decreasing temperatures of the hot spring: upstream, midstream and downstream, demonstrating significant loss of both archaeal presence and microbial variety using universal 16S rDNA bacterial primers.

Figure 8 shows a Krona plot analysis of the comparative classification for different parts of the hot spring stream with temperatures decreasing with distance from the source. This revealed that sequencing with universal bacterial primers, archaea and bacteria species was the most common in terms of microbial variety in the most thermophilic part of the source (upstream), then, as the temperature decreased downstream, the archaea disappeared, the bacterial diversity of species decreased and *Novosphingobium* species became the more dominant (up to 50%). However, this finding runs counter to investigations reported in the literature, where an increase in temperature results in decreasing microbial variety, due to mainly temperature stress and low adaptation rates of the microorganisms to the stressful thermophilic conditions^46,47^. This suggests that universal bacterial primers did not represent an accurate phylogenetic model of the hot spring in terms of microbial variety. On the other hand, using custom designed primers in this study, the phylogenetic model of the three parts of the hot spring suggested a reasonably accurate representation in terms of microbial diversity, showing a decline in microbial diversity with increasing temperatures (Fig. 8). Even though the custom primers were designed using archaeal genomes, these still demonstrate a better representation in terms of the detection and dissociation of bacterial species.

**Figure 9:**
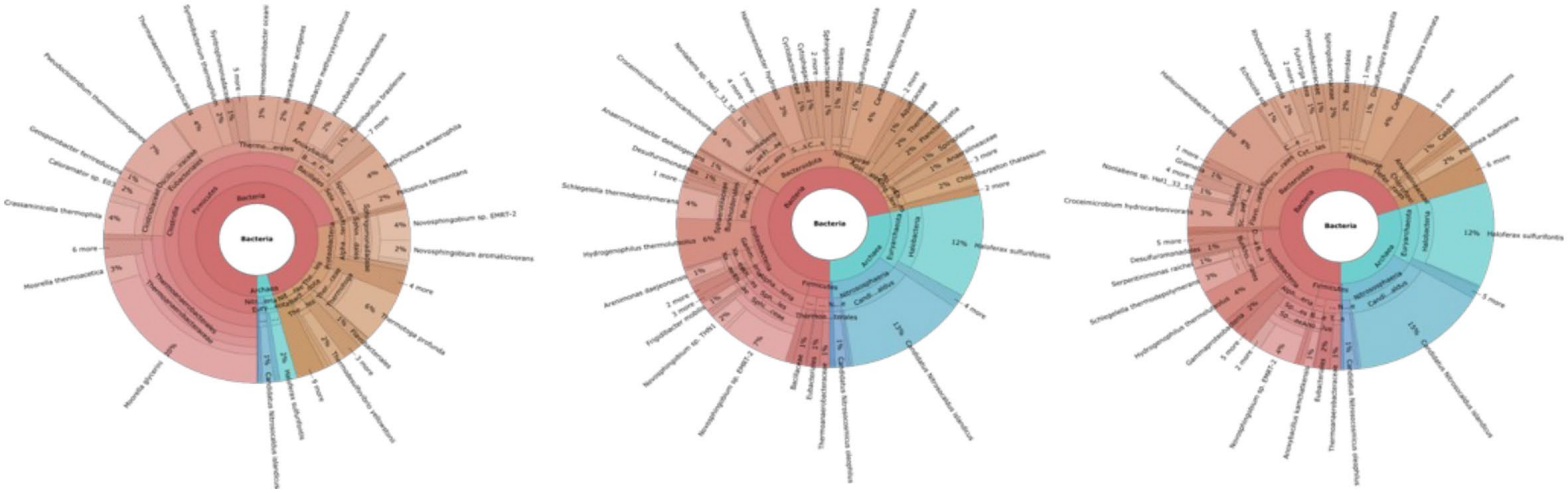
Krona plot analysis of three different points of decreasing temperatures of the hot spring: upstream, midstream and downstream, demonstrating significant loss of both archaeal presence and microbial variety using custom designed archaeal 16S rDNA primers.

Custom-made primers worked well on direct DNA isolation from water samples (uncultured); however, it was not possible to classify archaeal presence with universal bacterial primers. The amount of archaea increases in the midstream, and at the beginning, downstream distancing from the hot spring source. This can be explained by the lower number of reads in the upstream samples, while the sensitivity may also have decreased. These primers detected 4% of archaea at the most highly thermophilic part of the stream, 28% at the middle part, and 38% at the most highly mesophilic part (downstream). With the universal bacterial primers, the presence of archaea was highest at the source (upstream) with 4%, but no archaea were detected at the mid and downstream points (Fig. 9).

The designed archaea primers were sensitive in detecting the presence of archaea only at mild temperatures, while, at higher temperatures, no difference was observed in the classification of archaea between universal primers and custom-designed archaea primers. The use of two different primers in the upstream sample showed that the archaea primers were able to classify a wider variety of species, but the universal primers dominantly classified *Anoxybacillus* and *Novosphingobium* species.

A study^48^ demonstrated a custom-primer design for archaea, which included a wider variety of species that live in psychrophilic (4 °C from freshwater samples) to mesophilic (37 °C from bioreactor samples) and temperatures and involved alignment of 8500 of sequences. This study reported at least 38% higher coverage of archaea compared to more commonly used universal primers. Another study^49^, involved distinctive environmental conditions (1 to 4 °C and high pressure) and aimed for higher detection of microbial communities living in niche conditions, similar to the this current study. While as a difference, that study claimed that short read sequencing and V3–V4 hypervariable region of 16S gene in terms of taxonomic assignment of oceanic consortia, and the better performance of the archaeal primer designed in that study in terms of diversity of archaeal communities, especially on Thaumarchaeota (Crenarchaeota), compared to universal primers, and reported up to 70% coverage for archaea. The current study includes niche growth conditions (thermophilic temperatures 60 °C and above, anaerobic and CO-utilizing) of archaea with hot springs of similar ecological conditions, therefore we acknowledge that our custom-built primers are not more comprehensive in terms of the range of archaea covered, however, these primers offer a more sensitive and robust detection, and are able to capture thermophilic and anaerobic hot spring archaea where they can be found in very small abundances. Another study^50^ targeted pig fecal samples using a short read NGS sequencer, Illumina MiSeq, and designed universal prokaryotic primers based on V3–V4 hypervariable regions of 16S gene. In their study, custom designed primers matched to 94.6% of Archaea rRNA gene sequences in the database used as a reference. It was additionally shown, similar to the current study, that, where a primer bias existed, archaeal species were also detected with the bacterial universal primer. Custom designed prokaryotic primers increased performance by 0.8% for Archaea compared to previously described universal primer sequences in the database used in that study, and they successfully lowered the bias with the custom designed prokaryotic primers when compared to commonly used universal prokaryotic primers.

Some 16S rDNA primer sets have been reported to include biases, thus it is important to minimize these, and obtain an even coverage of both overall and desired microorganisms for an optimized resolution within primer sets. To achieve this, firstly, regarding 16S primers and their relative performance regarding microbial representation, it would be wise to conduct preliminary investigations with less complex samples or mock microbial communities before implementations on environmental extreme areas, especially for the investigation of archaeal species^51,52^. Imitation restrictions of these unknown areas in extreme conditions require sensitive and comprehensive studies to achieve maximum precision with microbial community profiling. Sequencing platforms directly affect the outcome of the study, as they differ greatly in terms of protocol directions (such as PCR conditions), and differences between MiSeq and HiSeq sequencing platforms were reported with their respective protocols using mock microbial communities^53^. High throughput sequencing platforms (Nanopore and PacBio technologies) present greater depths; however, another study^52^ suggested that observed biases using the MiSeq platform between different primers could not be solved by greater depths, but it is possible to resolve biases by the comparative approach, using mock communities and field samples. A study extensively comparing both platform and primer choice effects on 16S sequencing reported that primer choice had a greater biological effect than sequencing platforms and emphasized the importance of experimental methods for achieving accurate representation of abundance in microbial communities^54^.

This current study explored hot spring thermophilic communities with investigational parameters, including the comparison of the community structure in different parts of the hot spring waterflow where water temperature ranged from 38.0 to 77.3 °C. Furthermore, we have compared several bioinformatic pipelines and demonstrated unalike prokaryotic profiles with the utilization of conventional and novel primers (universal vs. custom design) for PCR amplification of the samples. Custom design primers indicated better results in terms of decreasing microbial diversity among increased water temperatures of hot springs while sequencing results with universal primers suggested the opposite. Study results also highlighted that both custom designed and universal prokaryotic primers have a certain bias towards *Novosphingobium* species, this was indicated by the samples where water temperature was too high for this mesophilic species (30–45 °C) to live and still showed an abundance of *Novosphingobium* species in bioinformatic analysis. Overall, this study is the crucial to report the effectiveness of different bioinformatic pipelines for nanopore sequencing studies together with representing valuable data with the custom design primers used.

As a conclusion, among the two methologies of using targeted custom-design primers and using different bioinformatic analysis pipelines for the classification of hot spring microorganisms, the primer choice had a critical effect on detection of archaeal species and also identification of the variety of bacterial species. Sequencing results revealed that enriched cultures demonstrated a significant increase of carboxydotrophic hydrogenogenic bacteria in abundance due to the manipulation of operational parameters in their favor. This approach has led to the discovery of a methodology for the detection of hydrogen producers from extreme environments. The current study also highlights the advantages of the nanopore sequencing tool for improving the feasibility of molecular workflows in microbial metagenomic studies and the proposed methodology could also be used in future works to investigate the roles of industrially important bacteria and archaea in similarly extreme environments.

## Methods

### Archaeal primer design for custom-built 16S rDNA primers

Custom-built primers were specifically designed for PCR amplification of 16S rDNA regions of thermophilic archaea that are likely to be isolated in Izmir province. Based on literature investigations, thermophilic and anaerobic archaea species reported in hot spring microbial community studies were identified and selected. Archaea sequences were downloaded from the NCBI database in FASTA format (Table 1).

**Table 1:** Archaeal species that are likely to be found in thermal waters in and around İzmir, living at a temperature of 50–65 °C, around pH 6–7.

Organism	Accession code	Reference
*Thermococcus barophilus*	NC_014804	^4^
*Thermococcus guaymasensis DSM11113*	NZ_CP007140	
*Thermococcus onnurineus NA1*	NC_011529	
*Thermococcus paralvinellae*	NZ_CP006965	
*Thermofilum adornatum*	NC_022093	
*Methanothermobacter thermautotrophicus*	NC_000916	
*Methanomethylovorans thermophila strain L2FAW*	NR_043089	^59^
*Methanomassiliicoccus luminyensis*	HQ896499	
*Methanococcus aeolicus*	(NC_009635)	
*Methanocaldococcus jannaschii*	NC_000909	^60^
*Archaeoglobus fulgidus*	NZ_CP006577	
*Thermosphaera aggregans*	NC_014160	^61^
*Methanocaldococcus vulcanius*	NC_013407	
*Methanocella conradii*	NC_017034	
*Methanothermobacter marburgensis*	NC_014408	^62^
*Methanosarcina thermophila*	NZ_CP009501	

In order to detect potential species, archaea species and their entire genomes were combined on the same text file. As an exemplary, it was decided to select a thermophilic archaeal species isolated from a hot spring *Thermococcus celer* and its 16s rDNA partial sequence (NCBI Reference Sequence: NR_113295.1) because species growth conditions showed similarity to the investigated hot springs in this study^55^. Archaeal sequence and the FASTA file were downloaded through the NCBI database.

For the alignment of the 16S rDNA sequence (query) and the merged archaeal sequences (subject) via BLAST + (BLASTN 2.10.1+), a Python code was programmed to give only the accession codes of the merged archaeal sequences. The program printed the access codes of the archaea, and codes were also transferred to BLAST+, so that it could load archaea sequences from its own database. The command ‘blastn -query “.\Methanocaldococcus 16S.txt” -subject Merged_archaea.fasta -outfmt 7 > output.tsv’ was entered to the BLAST + program. Tabular with comment lines was selected as a formatting option to save the results as output.tsv format in an Excel file. The start and end of the sequences in common to the 16 s sequence, and the archaea sequence in the tsv file were determined as the query start (q.start) and end points (q.end).

A Python program called SeqExtractor was coded in order to detect the archaea name and sequences from the ‘merged_archaea.fasta’ file, which was initially given in the Python program to detect the 8th (query start) and 9th (query end) rows in the query table in tsv file given by the BLAST program. Query start and end points from the archaea sequences were extracted and printed as alignments. Archaea accession codes and alignment sequences were recorded in a file named ‘16ssequences.fasta’. The script receives two files, a list of fasta files containing the genomic sequences of selected archaea species and a BLAST output where a reference 16S sequence was aligned to these sequences. The script extracts 16S sequences from each genome and assembles them in a single file in fasta format, ready to be aligned.

**Figure 10:**
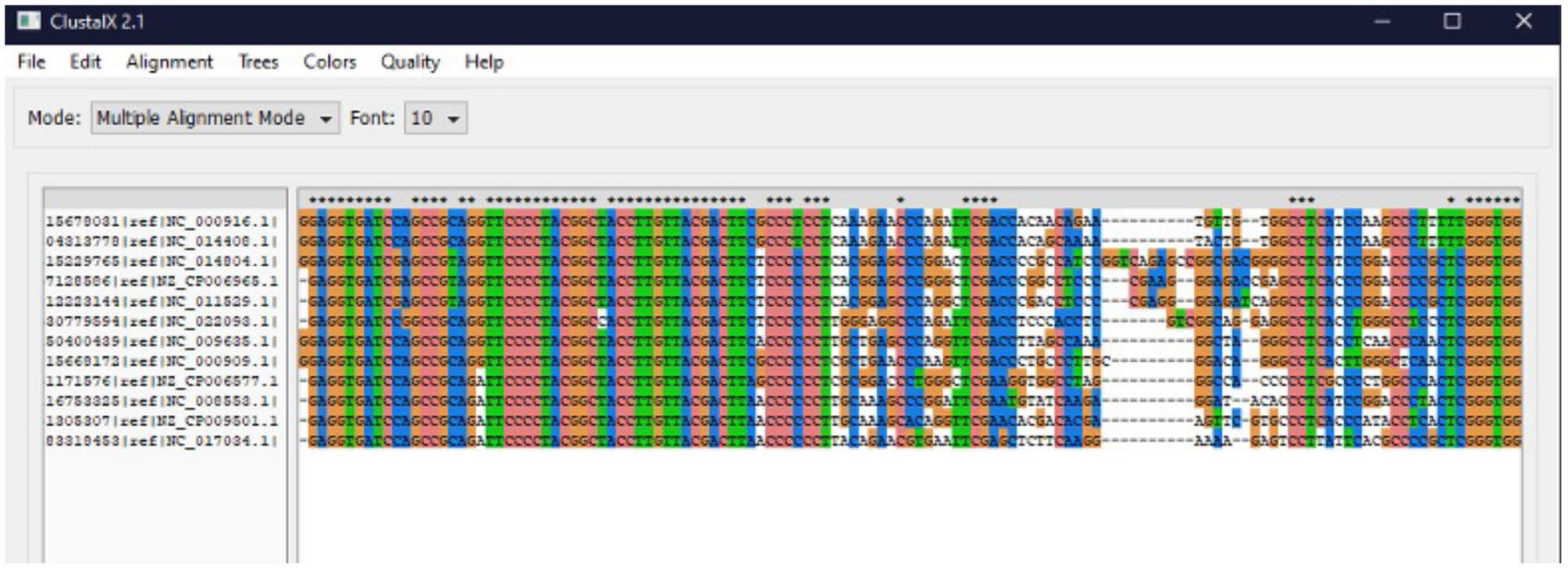
Alignment results on ClustalX for the archaea species likely to be found in the İzmir hot springs.

These sequences were aligned with the UGENE Multiple Sequence Alignment program (v.33)^56^. Some sequences were found to be inverted, otherwise the sequences were very similar and aligned. 16S sequences were also aligned with the ClustalX Multiple Sequence Alignment program (v2.0)^57^. Reverse complementary sequences of reversed sequences were obtained (via were loaded into the ClustalX program together with the sequences, and realigned (Fig. 10). Forward and reverse archaeal primers were determined according to the obtained alignment results (Table 2).

**Table 2:** Thermophilic bacterial and archaeal 16S rDNA primers.

Primers	Forward	Reverse
Bacteria primers (Universal)	Tailed-27For: 5ʹ TTTCTGTTGGTGCTGATATTGCAGAGTTTGATCMTGGCTCAG 3ʹ	Tailed-1492Rev: 5ʹ ACTTGCCTGTCGCTCTATCTTCCGGTTACCTTGTTACGACTT 3ʹ
Archaea primers (custom designed)	For27: ACTTGCCTGTCGCTCTATCTTCTACGGCTACCTTGTTACGAC	Rev621: TTTCTGTTGGTGCTGATATTGCCTGAAACTTAAAGGAATTGGC Rev1413: TTTCTGTTGGTGCTGATATTGCACKGCTCAGTAACACGTG

Universal 16S rDNA Bacterial primers were supplied by Nucleus Genetic Inc. In order to obtain the same T_m_ (melting temperature) value of the primers to perform PCR under the same conditions as the primers to be used in bacteria, the T_m_ value of the specific bacterial primers was first found on the website (https://tmcalculator.neb.com) and the Archaea primer sequences were shortened accordingly (Table 2). PCR trial conducted with custom designed 16S rDNA primers for archaea showed the success of the amplification of 16S region of the DNA samples isolated from hot springs.

### Sampling and cultivation of hot spring isolates

**Figure 11:**
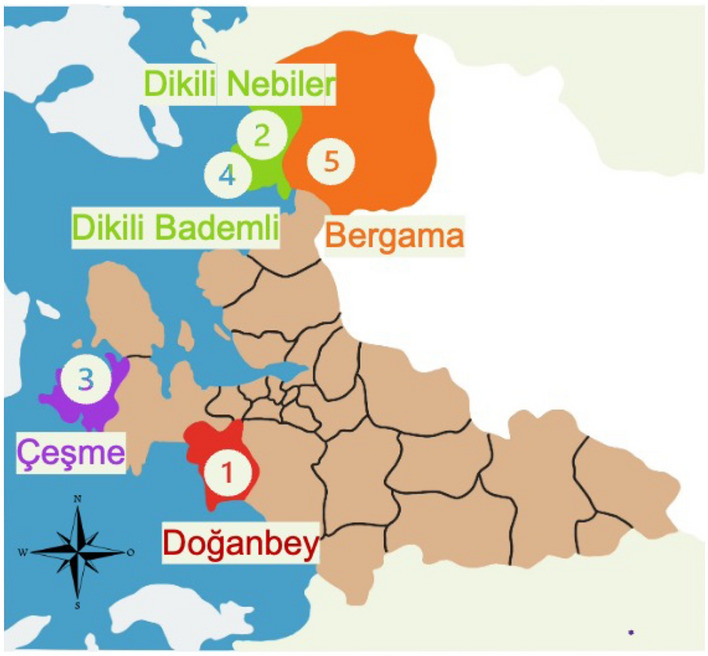
Schematic map locating the hot springs visited in this study in İzmir, Türkiye (The figure was created by the authors using Microsoft Office Power Point Tool (version 16.73; https://www.microsoft.com/tr-tr/microsoft-365/business/).

Five different hot springs located in Doğanbey, Çeşme, Dikili Bademli, Dikili Nebiler, and Bergama, Izmir, Türkiye (Table 3) were field-visited to collect samples between 25/05/2021 to 23/08/2021 (Fig. 11). Samples were collected in sterile plastic bottles and transferred to the laboratory in a heat-insulated container. pH, temperature, and oxidation–reduction potential (ORP) measurements were made from each collection point with a portable pH meter (MW 105 Max, Milwaukee, USA). The samples were processed in two different workflows:

1. Direct DNA isolation of hot spring samples was performed (5 L of volume) according to the manufacturer’s instructions with Thermo Fisher GeneJET Genomic DNA Purification Kit (Thermo Fisher Scientific, USA) in triplicate, without any enrichment. Isolated DNA was stored at − 20 °C for further 16S rDNA analysis.
2. The collected samples were transferred into sterile 50 mL falcon tubes and centrifuged at 1398×*g* for 10 min (Hanil Science Industrial, South Korea). The enrichment media content including macronutrients, micronutrients, and vitamins, was prepared as indicated in Ref.^32^. pH of the nutrient medium was fixed to 6.8 with 0.5 M HCl and 54 mL of prepared basal medium was transferred to glass bottles with a total volume of 100 mL. The bottles were closed with gas-tight rubber stoppers and metal caps, and autoclaved at 121 °C and 15 min. O_2_ in the headspace of the closed bottles was removed by purging with N_2_ gas. Following sterilization, 0.1 mL of sterile 10× vitamin solution was added to sealed vials containing 54 mL of sterile anaerobic thermophilic medium, and 6 mL (10%) collected pellet was inoculated. Bottles were fed with two different gaseous substrates: syngas (H_2_ (5%), O_2_ (5%), CO (10%), CH_4_ (5%), CO_2_ (20%) and N_2_ (40%)) and 100% CO gas for 30 s. The bottles were inoculated and placed in an incubator at a temperature appropriate to the isolates’ isolation temperatures (45, 55, 60 or 65 °C). Following serial cultures grown to the logarithmic phase, DNA isolation was performed according to the manufacturer’s instructions, and isolates were stored at − 20 °C for further 16S rDNA analysis.

**Table 3:** Summary of the 16S rDNA metagenomic investigation of the classification of 5 distinct hot springs in Izmir area.

Isolation source	Isolation technique	Number of raw reads	Classified reads (%)	Unclassified reads (%)	Microbial reads (%)	Bacterial reads (%)
All samples	Isolated cells + cultured cells	6.391.436	99.4	0.641	99.3	97.8
Doğanbey	Isolated cells	877.400	99.3	0.699	99.2	89.8
	Cultured cells	1.902.906	99.8	0.196	99.8	99.7
Çeşme	Isolated cells	248.028	99.9	0.0867	99.9	96.3
	Cultured cells	291.211	98.2	1.82	98.2	98.1
Dikili Bademli	Isolated cells	50.438	99.8	0.151	99.8	99.4
	Cultured cells	258.004	98.2	1.75	98.2	98.2
Dikili Nebiler	Isolated cells	1.148	12	88	12	11.9
Bergama	Isolated cells	125.354	99.5	0.534	99.5	99.2
	Cultured cells	999.771	99.6	0.436	99.6	99.4

Isolated cells describe the uncultured microorganisms directly analyzed from the water samples, and cultured cells describe the microorganisms grown under CO and enriched media.

### DNA library preparation

DNA libraries were prepared using consecutive PCR, to both amplify the 16S region and barcoding. 16S rDNA genomic sequences were amplified in two separate ways, using a universal primer set, and an in-house thermophilic archaea-specific primer. Sequencing bacterial 16S rDNA regions was conducted using two primers with barcoding tag in the upstream, 5ʹ TTT CTG TTG GTG CTG ATA TTG CAG AGT TTG ATC MTG GCT CAG 3ʹ and 5ʹ ACT TGC CTG TCG CTC TAT CTT CCG GTT ACC TTG TTA CGA CTT 3ʹ. To amplify archaeal 16S sequences, custom-designed thermophilic-specific primer set (from Table 2), the For27 and Rev1413 primers (5ʹ ACT TGC CTG TCG CTC TAT CTT CTA CGG CTA CCT TGT TAC GAC 3ʹ and 5ʹ TTT CTG TTG GTG CTG ATA TTG CAC *K*GC TCA GTA ACA CGT G 3ʹ) were used.

16S regions were amplified using the above-mentioned primers by standard PCR with 12 cycles. The amplicon sizes were confirmed on agarose gel and isolated using AMPure XP (Beckman Coulter) nucleic acid purification beads. Barcoding was performed according to the manufacturer’s protocol (Oxford Nanopore Technologies: SQK-LSK109 with EXP-PBC096). Briefly, samples were barcoded by PCR using the barcoding primers. Amplicons were purified using AMPure XP beads and quantified using a Qubit fluorometer (Thermo Fisher, Germany). The samples were pooled to yield 1 μg in 47 μL. Using Ultra II End Prep (NEB) 3ʹ ends were adenylated and 5ʹ ends were phosphorylated before AMPure XP clean-up. The adapters were ligated to pooled DNA using Quick T4 Ligase (NEB).

### Nanopore 16S sequencing and bioinformatic analysis

Nanopore sequencing was performed using an ONT MinION device connected to a workstation running MinKNOW software under Windows. Sequencing was stopped after 48 h without synchronous base calling and a minimum score threshold of 7.

After sequencing was completed, base calling, barcoding and trimming was performed using Guppy under Ubuntu 18 on a workstation equipped with a GTX 1050 for GPU acceleration. Barcoded reads were analyzed and taxonomically classified using three different bioinformatic pipelines: Kraken (v2.1.2), Epi2Me (v3.6.2), and Mothur platform^58^; these three approaches were conducted separately.

For the Kraken classification pipeline, barcoded samples classified with Kraken software were visualized employing Sankey visualization, using the Pavian tool, and these were demonstrated with default parameters (number of taxa at each level:10, scale distance between nodes: 0.9, figure height: 400, node border-width: 0, the opacity of links: 0.6, node label margins: 1).

For the in-house BLAST (Mothur) classification and visualization pipeline, for targeting the 16S rDNA region, the obtained reads between 1250 and 1750 bp in length were filtered using Trimmomatic and the remaining reads were excluded from the analysis. Filtered reads were analyzed with a customized workflow by matching each sequence with the BLAST algorithm during the filtering process. In the matching results, an operational taxonomic unit (OTU) was created by taking the taxonomic data of sequences with more than 60% reference coverage and 80% pairwise similarity. Mothur platform was used to demonstrate taxonomic classifications and Krona charts. Graphs and tables in the analyses were generated with Python libraries.

### Scanning electron microscopy (SEM) imaging

Hydrogen-producing isolates from Çeşme, Doğanbey, and Bergama mixed cultures were screened using Scanning Electron Microscopy (SEM) (FEI Quanta 250 FEG Philips, Netherlands) imaging for the detailed examination of their sizes and shapes. 15 mL of liquid culture were transferred to sterile falcon tubes and then centrifuged 1789×*g* for 10 min, and the supernatant was discarded. The pellet was suspended in a phosphate-buffered saline (PBS) solution (4.43 g NaCl, 0.546 g of Na_2_HPO_4,_ and 0.138 g of NaH_2_PO_4_ in 500 mL of UP water) with a pH of 7.5 and centrifuged (1789×*g* for 10 min). The supernatant was discarded and the pellet was re-suspended in 2.5% (v/v) formaldehyde solution for 1 h for cell fixation. Following the formaldehyde fixation, the falcon tubes were centrifuged three times for final pellet collection. The obtained pellet was fixed by dropping the final pellet onto glass slides and the matte side of a piece of aluminum foil, and air dried in an incubator at a temperature of 37 °C, followed by gold coating^39^.

### Supplementary Information

Supplementary Information 1.

Supplementary Information 2.

## Data Availability

The datasets generated and analysed during the current study are available in the NCBI repository with the SRA Bioproject Accession Number [PRJNA954581].
