# Supplementary material for: Using custom-built primers and nanopore sequencing to evaluate CO-utilizer bacterial and archaeal populations linked to bioH2 production
Source: Sci Rep. 2023 Oct 9;13:17025. doi: 10.1038/s41598-023-44357-3 (PMC10562470; doi:10.1038/s41598-023-44357-3)
Supplement: Supplementary file 1 — Supplementary Information 1. [file 41598_2023_44357_MOESM1_ESM.docx]

BLASTN 2.10.1+

Reference: Zheng Zhang, Scott Schwartz, Lukas Wagner, and Webb

Miller (2000), "A greedy algorithm for aligning DNA sequences", J

Comput Biol 2000; 7(1-2):203-14.

Database: User specified sequence set (Input: Merged_archaea.fasta).

31 sequences; 60,443,697 total letters

Query= AB603519.1 Thermococcus celer gene for 16S rRNA, partial sequence,

strain: JCM 8558

Length=1321

Score E

Sequences producing significant alignments: (Bits) Value

gi|1214752580|ref|NZ_CP014854.1| Thermococcus celer Vu 13 = JCM 8... 2433 0.0

gi|212223144|ref|NC_011529.1| Thermococcus onnurineus NA1, comple... 2396 0.0

gi|1011496410|ref|NZ_CP014750.1| Thermococcus peptonophilus strai... 2392 0.0

gi|57639935|ref|NC_006624.1| Thermococcus kodakarensis KOD1, comp... 2392 0.0

gi|1214747492|ref|NZ_CP014862.1| Thermococcus profundus strain DT... 2383 0.0

gi|240102057|ref|NC_012804.1| Thermococcus gammatolerans EJ3, com... 2381 0.0

gi|1214774922|ref|NZ_CP014855.1| Thermococcus gorgonarius strain ... 2375 0.0

gi|1011478452|ref|NZ_CP007140.1| Thermococcus guaymasensis DSM 11... 2375 0.0

gi|757139166|ref|NZ_CP007264.1| Thermococcus nautili strain 30-1 ... 2362 0.0

gi|390960176|ref|NC_018015.1| Thermococcus cleftensis strain CL1,... 2362 0.0

gi|910102422|ref|NZ_CP008887.1| Thermococcus eurythermalis strain... 2335 0.0

gi|1214743344|ref|NZ_CP015102.1| Thermococcus pacificus strain P-... 2318 0.0

gi|530547444|ref|NC_022084.1| Thermococcus litoralis DSM 5473, co... 2289 0.0

gi|757128586|ref|NZ_CP006965.1| Thermococcus paralvinellae strain... 2287 0.0

gi|315229765|ref|NC_014804.1| Thermococcus barophilus MP, complet... 2287 0.0

gi|1741192688|ref|NZ_CP023154.1| Pyrococcus furiosus DSM 3638 chr... 2218 0.0

gi|337283511|ref|NC_015680.1| Pyrococcus yayanosii CH1, complete ... 2213 0.0

gi|1057206559|ref|NZ_LN999010.1| Thermococcus chitonophagus isola... 2211 0.0

gi|851302729|ref|NZ_CP006019.1| Palaeococcus pacificus DY20341 ch... 2174 0.0

gi|261402131|ref|NC_013407.1| Methanocaldococcus vulcanius M7, co... 1565 0.0

gi|15668172|ref|NC_000909.1| Methanocaldococcus jannaschii DSM 26... 1535 0.0

gi|851171576|ref|NZ_CP006577.1| Archaeoglobus fulgidus DSM 8774 c... 1448 0.0

gi|530779594|ref|NC_022093.1| Thermofilum adornatum, complete seq... 1227 0.0

gi|304313778|ref|NC_014408.1| Methanothermobacter marburgensis st... 1195 0.0

gi|15678031|ref|NC_000916.1| Methanothermobacter thermautotrophic... 1194 0.0

gi|296241748|ref|NC_014160.1| Thermosphaera aggregans DSM 11486, ... 1153 0.0

gi|150400439|ref|NC_009635.1| Methanococcus aeolicus Nankai-3, co... 1088 0.0

gi|116753325|ref|NC_008553.1| Methanothrix thermoacetophila PT, c... 972 0.0

gi|386000717|ref|NC_017527.1| Methanosaeta harundinacea 6Ac, comp... 950 0.0

gi|851305307|ref|NZ_CP009501.1| Methanosarcina thermophila TM-1 c... 935 0.0

gi|383318453|ref|NC_017034.1| Methanocella conradii HZ254, comple... 787 0.0

> gi|1214752580|ref|NZ_CP014854.1| Thermococcus celer Vu 13 = JCM

8558, complete genome

Length=1866819

Score = 2433 bits (1317), Expect = 0.0

Identities = 1320/1321 (99%), Gaps = 1/1321 (0%)

Strand=Plus/Minus

Query 1 GTCCGACTAAGCCATGCGAGTCATGGGGCGCCTTGCGCGCACCGGCGGACGGCTCAGTAA 60

||||||||||||||||||||||||||||||||||||||||||||||||||||||||||||

Sbjct 1055903 GTCCGACTAAGCCATGCGAGTCATGGGGCGCCTTGCGCGCACCGGCGGACGGCTCAGTAA 1055844

Query 61 CACGTCGGTAACCTACCCTCGGGAGGGGGATAACCCCGGGAAACTGGGGCTAATCCCCCA 120

||||||||||||||||||||||||||||||||||||||||||||||||||||||||||||

Sbjct 1055843 CACGTCGGTAACCTACCCTCGGGAGGGGGATAACCCCGGGAAACTGGGGCTAATCCCCCA 1055784

Query 121 TAGGCCTGAGGTACTGGAAGGTCCTCAGGCCGAAAGGGGCTCTGCCCGCCCGAGGATGGG 180

||||||||||||||||||||||||||||||||||||||||||||||||||||||||||||

Sbjct 1055783 TAGGCCTGAGGTACTGGAAGGTCCTCAGGCCGAAAGGGGCTCTGCCCGCCCGAGGATGGG 1055724

Query 181 CCGGCGGCCGATTAGGTAGTTGGTGGGGTAACGGCCCACCAAGCCGAAGATCGGTACGGG 240

||||||||||||||||||||||||||||||||||||||||||||||||||||||||||||

Sbjct 1055723 CCGGCGGCCGATTAGGTAGTTGGTGGGGTAACGGCCCACCAAGCCGAAGATCGGTACGGG 1055664

Query 241 CCATGAGAGTGGGAGCCCGGAGATGGACACTGAGACACGGGTCCAGGCCCTACGGGGCGC 300

||||||||||||||||||||||||||||||||||||||||||||||||||||||||||||

Sbjct 1055663 CCATGAGAGTGGGAGCCCGGAGATGGACACTGAGACACGGGTCCAGGCCCTACGGGGCGC 1055604

Query 301 AGCAGGCGCGAAACCTCCGCAATGCGGGCAACCGCGACGGGGGGACCCCCAGTGCCGTGG 360

||||||||||||||||||||||||||||||||||||||||||||||||||||||||||||

Sbjct 1055603 AGCAGGCGCGAAACCTCCGCAATGCGGGCAACCGCGACGGGGGGACCCCCAGTGCCGTGG 1055544

Query 361 CAACGCCACGGCTTTTCCGGAGTGTAAAAAGCTCCGGGAATAAGGGCTGGGCAAGGCCGG 420

||||||||||||||||||||||||||||||||||||||||||||||||||||||||||||

Sbjct 1055543 CAACGCCACGGCTTTTCCGGAGTGTAAAAAGCTCCGGGAATAAGGGCTGGGCAAGGCCGG 1055484

Query 421 TGGCAGCCGCCGCGGTAATACCGGCGGCCCGAGTGGTGGCCGCTATTATTGGGCCTAAAG 480

||||||||||||||||||||||||||||||||||||||||||||||||||||||||||||

Sbjct 1055483 TGGCAGCCGCCGCGGTAATACCGGCGGCCCGAGTGGTGGCCGCTATTATTGGGCCTAAAG 1055424

Query 481 CGTCCGTAGCCGGGCCCGTAAGTCCCTGGCGAAATCCCACGGCTCAACCGTGGGGCTTGC 540

||||||||||||||||||||||||||||||||||||||||||||||||||||||||||||

Sbjct 1055423 CGTCCGTAGCCGGGCCCGTAAGTCCCTGGCGAAATCCCACGGCTCAACCGTGGGGCTTGC 1055364

Query 541 TGGGGATACTGCGGGCCTTGGGACCGGGAGAGGCCGGGGGTACCCCTGGGGTAGGGGTGA 600

||||||||||||||||||||||||||||||||||||||||||||||||||||||||||||

Sbjct 1055363 TGGGGATACTGCGGGCCTTGGGACCGGGAGAGGCCGGGGGTACCCCTGGGGTAGGGGTGA 1055304

Query 601 AATCCTATAATCCCAGGGGGACCGCCAGTGGCGAAGGCGCCCGGCTGGAACGGGTCCGAC 660

||||||||||||||||||||||||||||||||||||||||||||||||||||||||||||

Sbjct 1055303 AATCCTATAATCCCAGGGGGACCGCCAGTGGCGAAGGCGCCCGGCTGGAACGGGTCCGAC 1055244

Query 661 GGTGAGGGACGAAGGCCAGGGGAGCGAACCGGATTAGATACCCGGGTAGTCCTGGCTGTA 720

||||||||||||||||||||||||||||||||||||||||||||||||||||||||||||

Sbjct 1055243 GGTGAGGGACGAAGGCCAGGGGAGCGAACCGGATTAGATACCCGGGTAGTCCTGGCTGTA 1055184

Query 721 AAGGATGCGGGCTAGGTGTCGGGCGAGCTTCGAGCTCGCCCGGTGCCGAAGGGAAGCCGT 780

||||||||||||||||||||||||||||||||||||||||||||||||||||||||||||

Sbjct 1055183 AAGGATGCGGGCTAGGTGTCGGGCGAGCTTCGAGCTCGCCCGGTGCCGAAGGGAAGCCGT 1055124

Query 781 TAAGCCCGCCGCCTGGGGAGTACGGCCGCAAGGCTGAAACTTAAAGGAATTGGCGGGGGA 840

||||||||||||||||||||||||||||||||||||||||||||||||||||||||||||

Sbjct 1055123 TAAGCCCGCCGCCTGGGGAGTACGGCCGCAAGGCTGAAACTTAAAGGAATTGGCGGGGGA 1055064

Query 841 GCACTACAAGGGGTGGAGCGTGCGGTTTAATTGGATTCAACGCCGGGAACCTCACCGGGG 900

||||||||||||||||||||||||||||||||||||||||||||||||||||||||||||

Sbjct 1055063 GCACTACAAGGGGTGGAGCGTGCGGTTTAATTGGATTCAACGCCGGGAACCTCACCGGGG 1055004

Query 901 GCGACGGCAGGATGAAGGCCAGGCTGAAGGTCTTGCCGGACACGCCGAGAGGAGGTGCAT 960

||||||||||||||||||||||||||||||||||||||||||||||||||||||||||||

Sbjct 1055003 GCGACGGCAGGATGAAGGCCAGGCTGAAGGTCTTGCCGGACACGCCGAGAGGAGGTGCAT 1054944

Query 961 GGCCGCCGTCAGCTCGTACCGTGAGGCGTCCACTTAAGTGTGGTAACGAGCGAGACCCGC 1020

||||||||||||||||||||||||||||||||||||||||||||||||||||||||||||

Sbjct 1054943 GGCCGCCGTCAGCTCGTACCGTGAGGCGTCCACTTAAGTGTGGTAACGAGCGAGACCCGC 1054884

Query 1021 GCCCCCCAGTTGCCAGTCCTTCCCGCTGGGGAGGAGGCACTCTGGGGGGACCGCCGGCGA 1080

|||||| |||||||||||||||||||||||||||||||||||||||||||||||||||||

Sbjct 1054883 GCCCCC-AGTTGCCAGTCCTTCCCGCTGGGGAGGAGGCACTCTGGGGGGACCGCCGGCGA 1054825

Query 1081 TAAGCCGGAGGAAGGAGCGGGCGACGGTAGGTCAGTATGCCCCGAAACCCCCGGGCTACA 1140

||||||||||||||||||||||||||||||||||||||||||||||||||||||||||||

Sbjct 1054824 TAAGCCGGAGGAAGGAGCGGGCGACGGTAGGTCAGTATGCCCCGAAACCCCCGGGCTACA 1054765

Query 1141 CGCGCGCTACAATGGGCGGGACAATGGGATCCGACCCCGAAAGGGGAAGGGAATCCCCTA 1200

||||||||||||||||||||||||||||||||||||||||||||||||||||||||||||

Sbjct 1054764 CGCGCGCTACAATGGGCGGGACAATGGGATCCGACCCCGAAAGGGGAAGGGAATCCCCTA 1054705

Query 1201 AACCCGCCCCCAGTTCGGATCGCGGGCTGCAACTCGCCCGCGTGAAGCTGGAATCCCTAG 1260

||||||||||||||||||||||||||||||||||||||||||||||||||||||||||||

Sbjct 1054704 AACCCGCCCCCAGTTCGGATCGCGGGCTGCAACTCGCCCGCGTGAAGCTGGAATCCCTAG 1054645

Query 1261 TACCCGCGTGTCATCATCGCGCGGCGAATACGTCCCTGCTCCTTGCACACACCGCCCGTC 1320

||||||||||||||||||||||||||||||||||||||||||||||||||||||||||||

Sbjct 1054644 TACCCGCGTGTCATCATCGCGCGGCGAATACGTCCCTGCTCCTTGCACACACCGCCCGTC 1054585

Query 1321 A 1321

|

Sbjct 1054584 A 1054584

> gi|212223144|ref|NC_011529.1| Thermococcus onnurineus NA1, complete

sequence

Length=1847607

Score = 2396 bits (1297), Expect = 0.0

Identities = 1314/1322 (99%), Gaps = 2/1322 (0%)

Strand=Plus/Minus

Query 1 GTCCGACTAAGCCATGCGAGTCATGGGGCGCCTTGCGCGCACCGGCGGACGGCTCAGTAA 60

||||||||||||||||||||||||||||||||||||||||||||||||||||||||||||

Sbjct 1279558 GTCCGACTAAGCCATGCGAGTCATGGGGCGCCTTGCGCGCACCGGCGGACGGCTCAGTAA 1279499

Query 61 CACGTCGGTAACCTACCCTCGGGAGGGGGATAACCCCGGGAAACTGGGGCTAATCCCCCA 120

||||||||||||||||||||||||||||||||||||||||||||||||||||||||||||

Sbjct 1279498 CACGTCGGTAACCTACCCTCGGGAGGGGGATAACCCCGGGAAACTGGGGCTAATCCCCCA 1279439

Query 121 TAGGCCTGAGGTACTGGAAGGTCCTCAGGCCGAAAGGGGCT-CTGCCCGCCCGAGGATGG 179

||||||||||||||||||||||||||||||||||||||||| ||||||||||||||||||

Sbjct 1279438 TAGGCCTGAGGTACTGGAAGGTCCTCAGGCCGAAAGGGGCTTCTGCCCGCCCGAGGATGG 1279379

Query 180 GCCGGCGGCCGATTAGGTAGTTGGTGGGGTAACGGCCCACCAAGCCGAAGATCGGTACGG 239

||||||||||||||||||||||||||||||||||||||||||||||||||||||||||||

Sbjct 1279378 GCCGGCGGCCGATTAGGTAGTTGGTGGGGTAACGGCCCACCAAGCCGAAGATCGGTACGG 1279319

Query 240 GCCATGAGAGTGGGAGCCCGGAGATGGACACTGAGACACGGGTCCAGGCCCTACGGGGCG 299

||||||||||||||||||||||||||||||||||||||||||||||||||||||||||||

Sbjct 1279318 GCCATGAGAGTGGGAGCCCGGAGATGGACACTGAGACACGGGTCCAGGCCCTACGGGGCG 1279259

Query 300 CAGCAGGCGCGAAACCTCCGCAATGCGGGCAACCGCGACGGGGGGACCCCCAGTGCCGTG 359

||||||||||||||||||||||||||||| ||||||||||||||||||||||||||||||

Sbjct 1279258 CAGCAGGCGCGAAACCTCCGCAATGCGGGAAACCGCGACGGGGGGACCCCCAGTGCCGTG 1279199

Query 360 GCAACGCCACGGCTTTTCCGGAGTGTAAAAAGCTCCGGGAATAAGGGCTGGGCAAGGCCG 419

||| ||||||||||||||||||||||||||||||||||||||||||||||||||||||||

Sbjct 1279198 GCATCGCCACGGCTTTTCCGGAGTGTAAAAAGCTCCGGGAATAAGGGCTGGGCAAGGCCG 1279139

Query 420 GTGGCAGCCGCCGCGGTAATACCGGCGGCCCGAGTGGTGGCCGCTATTATTGGGCCTAAA 479

||||||||||||||||||||||||||||||||||||||||||||||||||||||||||||

Sbjct 1279138 GTGGCAGCCGCCGCGGTAATACCGGCGGCCCGAGTGGTGGCCGCTATTATTGGGCCTAAA 1279079

Query 480 GCGTCCGTAGCCGGGCCCGTAAGTCCCTGGCGAAATCCCACGGCTCAACCGTGGGGCTTG 539

||||||||||||||||||||||||||||||||||||| ||||||||||||||||||||||

Sbjct 1279078 GCGTCCGTAGCCGGGCCCGTAAGTCCCTGGCGAAATCTCACGGCTCAACCGTGGGGCTTG 1279019

Query 540 CTGGGGATACTGCGGGCCTTGGGACCGGGAGAGGCCGGGGGTACCCCTGGGGTAGGGGTG 599

||||||||||||||||||||||||||||||||||||||||||||||||||||||||||||

Sbjct 1279018 CTGGGGATACTGCGGGCCTTGGGACCGGGAGAGGCCGGGGGTACCCCTGGGGTAGGGGTG 1278959

Query 600 AAATCCTATAATCCCAGGGGGACCGCCAGTGGCGAAGGCGCCCGGCTGGAACGGGTCCGA 659

||||||||||||||||||||||||||||||||||||||||||||||||||||||||||||

Sbjct 1278958 AAATCCTATAATCCCAGGGGGACCGCCAGTGGCGAAGGCGCCCGGCTGGAACGGGTCCGA 1278899

Query 660 CGGTGAGGGACGAAGGCCAGGGGAGCGAACCGGATTAGATACCCGGGTAGTCCTGGCTGT 719

||||||||||||||||||||||||||||||||||||||||||||||||||||||||||||

Sbjct 1278898 CGGTGAGGGACGAAGGCCAGGGGAGCGAACCGGATTAGATACCCGGGTAGTCCTGGCTGT 1278839

Query 720 AAAGGATGCGGGCTAGGTGTCGGGCGAGCTTCGAGCTCGCCCGGTGCCGAAGGGAAGCCG 779

||||||||||||||||||||||||||||||||||||||||||||||||||||||||||||

Sbjct 1278838 AAAGGATGCGGGCTAGGTGTCGGGCGAGCTTCGAGCTCGCCCGGTGCCGAAGGGAAGCCG 1278779

Query 780 TTAAGCCCGCCGCCTGGGGAGTACGGCCGCAAGGCTGAAACTTAAAGGAATTGGCGGGGG 839

||||||||||||||||||||||||||||||||||||||||||||||||||||||||||||

Sbjct 1278778 TTAAGCCCGCCGCCTGGGGAGTACGGCCGCAAGGCTGAAACTTAAAGGAATTGGCGGGGG 1278719

Query 840 AGCACTACAAGGGGTGGAGCGTGCGGTTTAATTGGATTCAACGCCGGGAACCTCACCGGG 899

||||||||||||||||||||||||||||||||||||||||||||||||||||||||||||

Sbjct 1278718 AGCACTACAAGGGGTGGAGCGTGCGGTTTAATTGGATTCAACGCCGGGAACCTCACCGGG 1278659

Query 900 GGCGACGGCAGGATGAAGGCCAGGCTGAAGGTCTTGCCGGACACGCCGAGAGGAGGTGCA 959

||||||||||||||||||||||||||||||||||||||||||||||||||||||||||||

Sbjct 1278658 GGCGACGGCAGGATGAAGGCCAGGCTGAAGGTCTTGCCGGACACGCCGAGAGGAGGTGCA 1278599

Query 960 TGGCCGCCGTCAGCTCGTACCGTGAGGCGTCCACTTAAGTGTGGTAACGAGCGAGACCCG 1019

||||||||||||||||||||||||||||||||||||||||||||||||||||||||||||

Sbjct 1278598 TGGCCGCCGTCAGCTCGTACCGTGAGGCGTCCACTTAAGTGTGGTAACGAGCGAGACCCG 1278539

Query 1020 CGCCCCCCAGTTGCCAGTCCTTCCCGCTGGGGAGGAGGCACTCTGGGGGGACCGCCGGCG 1079

||||||| ||||||||||||| |||| |||||||||||||||||||||||||||||||||

Sbjct 1278538 CGCCCCC-AGTTGCCAGTCCTCCCCGTTGGGGAGGAGGCACTCTGGGGGGACCGCCGGCG 1278480

Query 1080 ATAAGCCGGAGGAAGGAGCGGGCGACGGTAGGTCAGTATGCCCCGAAACCCCCGGGCTAC 1139

||||||||||||||||||||||||||||||||||||||||||||||||||||||||||||

Sbjct 1278479 ATAAGCCGGAGGAAGGAGCGGGCGACGGTAGGTCAGTATGCCCCGAAACCCCCGGGCTAC 1278420

Query 1140 ACGCGCGCTACAATGGGCGGGACAATGGGATCCGACCCCGAAAGGGGAAGGGAATCCCCT 1199

||||||||||||||||||||||||||||||||||||||||||||||||||||||||||||

Sbjct 1278419 ACGCGCGCTACAATGGGCGGGACAATGGGATCCGACCCCGAAAGGGGAAGGGAATCCCCT 1278360

Query 1200 AAACCCGCCCCCAGTTCGGATCGCGGGCTGCAACTCGCCCGCGTGAAGCTGGAATCCCTA 1259

|||||||||| |||||||||||||||||||||||||||||||||||||||||||||||||

Sbjct 1278359 AAACCCGCCCTCAGTTCGGATCGCGGGCTGCAACTCGCCCGCGTGAAGCTGGAATCCCTA 1278300

Query 1260 GTACCCGCGTGTCATCATCGCGCGGCGAATACGTCCCTGCTCCTTGCACACACCGCCCGT 1319

||||||||||||||||||||||||||||||||||||||||||||||||||||||||||||

Sbjct 1278299 GTACCCGCGTGTCATCATCGCGCGGCGAATACGTCCCTGCTCCTTGCACACACCGCCCGT 1278240

Query 1320 CA 1321

||

Sbjct 1278239 CA 1278238

> gi|1011496410|ref|NZ_CP014750.1| Thermococcus peptonophilus strain

OG-1 chromosome, complete genome

Length=1847000

Score = 2392 bits (1295), Expect = 0.0

Identities = 1315/1324 (99%), Gaps = 4/1324 (0%)

Strand=Plus/Minus

Query 1 GTCCGACTAAGCCATGCGAGTCATGGGGCGC-CT-TGCGCGCACCGGCGGACGGCTCAGT 58

||||||||||||||||||||||||||||||| || |||||||||||||||||||||||||

Sbjct 785030 GTCCGACTAAGCCATGCGAGTCATGGGGCGCGCTCTGCGCGCACCGGCGGACGGCTCAGT 784971

Query 59 AACACGTCGGTAACCTACCCTCGGGAGGGGGATAACCCCGGGAAACTGGGGCTAATCCCC 118

||||||||||||||||||||||||||||||||||||||||||||||||||||||||||||

Sbjct 784970 AACACGTCGGTAACCTACCCTCGGGAGGGGGATAACCCCGGGAAACTGGGGCTAATCCCC 784911

Query 119 CATAGGCCTGAGGTACTGGAAGGTCCTCAGGCCGAAAGGGGC-TCTGCCCGCCCGAGGAT 177

|||||||||||||||||||||||||||||||||||||||||| |||||||||||||||||

Sbjct 784910 CATAGGCCTGAGGTACTGGAAGGTCCTCAGGCCGAAAGGGGCATCTGCCCGCCCGAGGAT 784851

Query 178 GGGCCGGCGGCCGATTAGGTAGTTGGTGGGGTAACGGCCCACCAAGCCGAAGATCGGTAC 237

||||||||||||||||||||||||||||||||||||||||||||||||||||||||||||

Sbjct 784850 GGGCCGGCGGCCGATTAGGTAGTTGGTGGGGTAACGGCCCACCAAGCCGAAGATCGGTAC 784791

Query 238 GGGCCATGAGAGTGGGAGCCCGGAGATGGACACTGAGACACGGGTCCAGGCCCTACGGGG 297

||||||||||||||||||||||||||||||||||||||||||||||||||||||||||||

Sbjct 784790 GGGCCATGAGAGTGGGAGCCCGGAGATGGACACTGAGACACGGGTCCAGGCCCTACGGGG 784731

Query 298 CGCAGCAGGCGCGAAACCTCCGCAATGCGGGCAACCGCGACGGGGGGACCCCCAGTGCCG 357

||||||||||||||||||||||||||||||||||||||||||||||||||||||||||||

Sbjct 784730 CGCAGCAGGCGCGAAACCTCCGCAATGCGGGCAACCGCGACGGGGGGACCCCCAGTGCCG 784671

Query 358 TGGCAACGCCACGGCTTTTCCGGAGTGTAAAAAGCTCCGGGAATAAGGGCTGGGCAAGGC 417

||||| |||||||||||||||||||||||||||||||||||||||||||||||||||||

Sbjct 784670 TGGCATAGCCACGGCTTTTCCGGAGTGTAAAAAGCTCCGGGAATAAGGGCTGGGCAAGGC 784611

Query 418 CGGTGGCAGCCGCCGCGGTAATACCGGCGGCCCGAGTGGTGGCCGCTATTATTGGGCCTA 477

||||||||||||||||||||||||||||||||||||||||||||||||||||||||||||

Sbjct 784610 CGGTGGCAGCCGCCGCGGTAATACCGGCGGCCCGAGTGGTGGCCGCTATTATTGGGCCTA 784551

Query 478 AAGCGTCCGTAGCCGGGCCCGTAAGTCCCTGGCGAAATCCCACGGCTCAACCGTGGGGCT 537

||||||||||||||||||||||||||||||||||||||||||||||||||||||||||||

Sbjct 784550 AAGCGTCCGTAGCCGGGCCCGTAAGTCCCTGGCGAAATCCCACGGCTCAACCGTGGGGCT 784491

Query 538 TGCTGGGGATACTGCGGGCCTTGGGACCGGGAGAGGCCGGGGGTACCCCTGGGGTAGGGG 597

||||||||||||||||||||||||||||||||||||||||||||||||||||||||||||

Sbjct 784490 TGCTGGGGATACTGCGGGCCTTGGGACCGGGAGAGGCCGGGGGTACCCCTGGGGTAGGGG 784431

Query 598 TGAAATCCTATAATCCCAGGGGGACCGCCAGTGGCGAAGGCGCCCGGCTGGAACGGGTCC 657

||||||||||||||||||||||||||||||||||||||||||||||||||||||||||||

Sbjct 784430 TGAAATCCTATAATCCCAGGGGGACCGCCAGTGGCGAAGGCGCCCGGCTGGAACGGGTCC 784371

Query 658 GACGGTGAGGGACGAAGGCCAGGGGAGCGAACCGGATTAGATACCCGGGTAGTCCTGGCT 717

||||||||||||||||||||||||||||||||||||||||||||||||||||||||||||

Sbjct 784370 GACGGTGAGGGACGAAGGCCAGGGGAGCGAACCGGATTAGATACCCGGGTAGTCCTGGCT 784311

Query 718 GTAAAGGATGCGGGCTAGGTGTCGGGCGAGCTTCGAGCTCGCCCGGTGCCGAAGGGAAGC 777

||||||||||||||||||||||||||||||||||||||||||||||||||| ||||||||

Sbjct 784310 GTAAAGGATGCGGGCTAGGTGTCGGGCGAGCTTCGAGCTCGCCCGGTGCCGGAGGGAAGC 784251

Query 778 CGTTAAGCCCGCCGCCTGGGGAGTACGGCCGCAAGGCTGAAACTTAAAGGAATTGGCGGG 837

||||||||||||||||||||||||||||||||||||||||||||||||||||||||||||

Sbjct 784250 CGTTAAGCCCGCCGCCTGGGGAGTACGGCCGCAAGGCTGAAACTTAAAGGAATTGGCGGG 784191

Query 838 GGAGCACTACAAGGGGTGGAGCGTGCGGTTTAATTGGATTCAACGCCGGGAACCTCACCG 897

||||||||||||||||||||||||||||||||||||||||||||||||||||||||||||

Sbjct 784190 GGAGCACTACAAGGGGTGGAGCGTGCGGTTTAATTGGATTCAACGCCGGGAACCTCACCG 784131

Query 898 GGGGCGACGGCAGGATGAAGGCCAGGCTGAAGGTCTTGCCGGACACGCCGAGAGGAGGTG 957

||||||||||||||||||||||||||||||||||||||||||||||||||||||||||||

Sbjct 784130 GGGGCGACGGCAGGATGAAGGCCAGGCTGAAGGTCTTGCCGGACACGCCGAGAGGAGGTG 784071

Query 958 CATGGCCGCCGTCAGCTCGTACCGTGAGGCGTCCACTTAAGTGTGGTAACGAGCGAGACC 1017

||||||||||||||||||||||||||||||||||||||||||||||||||||||||||||

Sbjct 784070 CATGGCCGCCGTCAGCTCGTACCGTGAGGCGTCCACTTAAGTGTGGTAACGAGCGAGACC 784011

Query 1018 CGCGCCCCCCAGTTGCCAGTCCTTCCCGCTGGGGAGGAGGCACTCTGGGGGGACCGCCGG 1077

||||||||| ||||||||||||| ||||||||||||||||||||||||||||||||||||

Sbjct 784010 CGCGCCCCC-AGTTGCCAGTCCTCCCCGCTGGGGAGGAGGCACTCTGGGGGGACCGCCGG 783952

Query 1078 CGATAAGCCGGAGGAAGGAGCGGGCGACGGTAGGTCAGTATGCCCCGAAACCCCCGGGCT 1137

||||||||||||||||||||||||||||||||||||||||||||||||||||||||||||

Sbjct 783951 CGATAAGCCGGAGGAAGGAGCGGGCGACGGTAGGTCAGTATGCCCCGAAACCCCCGGGCT 783892

Query 1138 ACACGCGCGCTACAATGGGCGGGACAATGGGATCCGACCCCGAAAGGGGAAGGGAATCCC 1197

||||||||||||||||||||||||||||||||||||||||||||||||||||||||||||

Sbjct 783891 ACACGCGCGCTACAATGGGCGGGACAATGGGATCCGACCCCGAAAGGGGAAGGGAATCCC 783832

Query 1198 CTAAACCCGCCCCCAGTTCGGATCGCGGGCTGCAACTCGCCCGCGTGAAGCTGGAATCCC 1257

|||||||||||| |||||||||||||||||||||||||||||||||||||||||||||||

Sbjct 783831 CTAAACCCGCCCTCAGTTCGGATCGCGGGCTGCAACTCGCCCGCGTGAAGCTGGAATCCC 783772

Query 1258 TAGTACCCGCGTGTCATCATCGCGCGGCGAATACGTCCCTGCTCCTTGCACACACCGCCC 1317

||||||||||||||||||||||||||||||||||||||||||||||||||||||||||||

Sbjct 783771 TAGTACCCGCGTGTCATCATCGCGCGGCGAATACGTCCCTGCTCCTTGCACACACCGCCC 783712

Query 1318 GTCA 1321

||||

Sbjct 783711 GTCA 783708

> gi|57639935|ref|NC_006624.1| Thermococcus kodakarensis KOD1,

complete sequence

Length=2088737

Score = 2392 bits (1295), Expect = 0.0

Identities = 1315/1324 (99%), Gaps = 4/1324 (0%)

Strand=Plus/Plus

Query 1 GTCCGACTAAGCCATGCGAGTCATGGGGCGC-CT-TGCGCGCACCGGCGGACGGCTCAGT 58

||||||||||||||||||||||||||||||| || |||||||||||||||||||||||||

Sbjct 2022901 GTCCGACTAAGCCATGCGAGTCATGGGGCGCGCTCTGCGCGCACCGGCGGACGGCTCAGT 2022960

Query 59 AACACGTCGGTAACCTACCCTCGGGAGGGGGATAACCCCGGGAAACTGGGGCTAATCCCC 118

||||||||||||||||||||||||||||||||||||||||||||||||||||||||||||

Sbjct 2022961 AACACGTCGGTAACCTACCCTCGGGAGGGGGATAACCCCGGGAAACTGGGGCTAATCCCC 2023020

Query 119 CATAGGCCTGAGGTACTGGAAGGTCCTCAGGCCGAAAGGGGC-TCTGCCCGCCCGAGGAT 177

|||||||||||||||||||||||||||||||||||||||||| |||||||||||||||||

Sbjct 2023021 CATAGGCCTGAGGTACTGGAAGGTCCTCAGGCCGAAAGGGGCATCTGCCCGCCCGAGGAT 2023080

Query 178 GGGCCGGCGGCCGATTAGGTAGTTGGTGGGGTAACGGCCCACCAAGCCGAAGATCGGTAC 237

||||||||||||||||||||||||||||||||||||||||||||||||||||||||||||

Sbjct 2023081 GGGCCGGCGGCCGATTAGGTAGTTGGTGGGGTAACGGCCCACCAAGCCGAAGATCGGTAC 2023140

Query 238 GGGCCATGAGAGTGGGAGCCCGGAGATGGACACTGAGACACGGGTCCAGGCCCTACGGGG 297

||||||||||||||||||||||||||||||||||||||||||||||||||||||||||||

Sbjct 2023141 GGGCCATGAGAGTGGGAGCCCGGAGATGGACACTGAGACACGGGTCCAGGCCCTACGGGG 2023200

Query 298 CGCAGCAGGCGCGAAACCTCCGCAATGCGGGCAACCGCGACGGGGGGACCCCCAGTGCCG 357

||||||||||||||||||||||||||||||||||||||||||||||||||||||||||||

Sbjct 2023201 CGCAGCAGGCGCGAAACCTCCGCAATGCGGGCAACCGCGACGGGGGGACCCCCAGTGCCG 2023260

Query 358 TGGCAACGCCACGGCTTTTCCGGAGTGTAAAAAGCTCCGGGAATAAGGGCTGGGCAAGGC 417

||||| |||||||||||||||||||||||||||||||||||||||||||||||||||||

Sbjct 2023261 TGGCATAGCCACGGCTTTTCCGGAGTGTAAAAAGCTCCGGGAATAAGGGCTGGGCAAGGC 2023320

Query 418 CGGTGGCAGCCGCCGCGGTAATACCGGCGGCCCGAGTGGTGGCCGCTATTATTGGGCCTA 477

||||||||||||||||||||||||||||||||||||||||||||||||||||||||||||

Sbjct 2023321 CGGTGGCAGCCGCCGCGGTAATACCGGCGGCCCGAGTGGTGGCCGCTATTATTGGGCCTA 2023380

Query 478 AAGCGTCCGTAGCCGGGCCCGTAAGTCCCTGGCGAAATCCCACGGCTCAACCGTGGGGCT 537

||||||||||||||||||||||||||||||||||||||||||||||||||||||||||||

Sbjct 2023381 AAGCGTCCGTAGCCGGGCCCGTAAGTCCCTGGCGAAATCCCACGGCTCAACCGTGGGGCT 2023440

Query 538 TGCTGGGGATACTGCGGGCCTTGGGACCGGGAGAGGCCGGGGGTACCCCTGGGGTAGGGG 597

||||||||||||||||||||||||||||||||||||||||||||||||||||||||||||

Sbjct 2023441 TGCTGGGGATACTGCGGGCCTTGGGACCGGGAGAGGCCGGGGGTACCCCTGGGGTAGGGG 2023500

Query 598 TGAAATCCTATAATCCCAGGGGGACCGCCAGTGGCGAAGGCGCCCGGCTGGAACGGGTCC 657

||||||||||||||||||||||||||||||||||||||||||||||||||||||||||||

Sbjct 2023501 TGAAATCCTATAATCCCAGGGGGACCGCCAGTGGCGAAGGCGCCCGGCTGGAACGGGTCC 2023560

Query 658 GACGGTGAGGGACGAAGGCCAGGGGAGCGAACCGGATTAGATACCCGGGTAGTCCTGGCT 717

||||||||||||||||||||||||||||||||||||||||||||||||||||||||||||

Sbjct 2023561 GACGGTGAGGGACGAAGGCCAGGGGAGCGAACCGGATTAGATACCCGGGTAGTCCTGGCT 2023620

Query 718 GTAAAGGATGCGGGCTAGGTGTCGGGCGAGCTTCGAGCTCGCCCGGTGCCGAAGGGAAGC 777

||||||||||||||||||||||||||||||||||||||||||||||||||| ||||||||

Sbjct 2023621 GTAAAGGATGCGGGCTAGGTGTCGGGCGAGCTTCGAGCTCGCCCGGTGCCGGAGGGAAGC 2023680

Query 778 CGTTAAGCCCGCCGCCTGGGGAGTACGGCCGCAAGGCTGAAACTTAAAGGAATTGGCGGG 837

||||||||||||||||||||||||||||||||||||||||||||||||||||||||||||

Sbjct 2023681 CGTTAAGCCCGCCGCCTGGGGAGTACGGCCGCAAGGCTGAAACTTAAAGGAATTGGCGGG 2023740

Query 838 GGAGCACTACAAGGGGTGGAGCGTGCGGTTTAATTGGATTCAACGCCGGGAACCTCACCG 897

||||||||||||||||||||||||||||||||||||||||||||||||||||||||||||

Sbjct 2023741 GGAGCACTACAAGGGGTGGAGCGTGCGGTTTAATTGGATTCAACGCCGGGAACCTCACCG 2023800

Query 898 GGGGCGACGGCAGGATGAAGGCCAGGCTGAAGGTCTTGCCGGACACGCCGAGAGGAGGTG 957

||||||||||||||||||||||||||||||||||||||||||||||||||||||||||||

Sbjct 2023801 GGGGCGACGGCAGGATGAAGGCCAGGCTGAAGGTCTTGCCGGACACGCCGAGAGGAGGTG 2023860

Query 958 CATGGCCGCCGTCAGCTCGTACCGTGAGGCGTCCACTTAAGTGTGGTAACGAGCGAGACC 1017

||||||||||||||||||||||||||||||||||||||||||||||||||||||||||||

Sbjct 2023861 CATGGCCGCCGTCAGCTCGTACCGTGAGGCGTCCACTTAAGTGTGGTAACGAGCGAGACC 2023920

Query 1018 CGCGCCCCCCAGTTGCCAGTCCTTCCCGCTGGGGAGGAGGCACTCTGGGGGGACCGCCGG 1077

|||| |||||||||||||||||| ||||||||||||||||||||||||||||||||||||

Sbjct 2023921 CGCG-CCCCCAGTTGCCAGTCCTCCCCGCTGGGGAGGAGGCACTCTGGGGGGACCGCCGG 2023979

Query 1078 CGATAAGCCGGAGGAAGGAGCGGGCGACGGTAGGTCAGTATGCCCCGAAACCCCCGGGCT 1137

||||||||||||||||||||||||||||||||||||||||||||||||||||||||||||

Sbjct 2023980 CGATAAGCCGGAGGAAGGAGCGGGCGACGGTAGGTCAGTATGCCCCGAAACCCCCGGGCT 2024039

Query 1138 ACACGCGCGCTACAATGGGCGGGACAATGGGATCCGACCCCGAAAGGGGAAGGGAATCCC 1197

||||||||||||||||||||||||||||||||||||||||||||||||||||||||||||

Sbjct 2024040 ACACGCGCGCTACAATGGGCGGGACAATGGGATCCGACCCCGAAAGGGGAAGGGAATCCC 2024099

Query 1198 CTAAACCCGCCCCCAGTTCGGATCGCGGGCTGCAACTCGCCCGCGTGAAGCTGGAATCCC 1257

|||||||||||| |||||||||||||||||||||||||||||||||||||||||||||||

Sbjct 2024100 CTAAACCCGCCCTCAGTTCGGATCGCGGGCTGCAACTCGCCCGCGTGAAGCTGGAATCCC 2024159

Query 1258 TAGTACCCGCGTGTCATCATCGCGCGGCGAATACGTCCCTGCTCCTTGCACACACCGCCC 1317

||||||||||||||||||||||||||||||||||||||||||||||||||||||||||||

Sbjct 2024160 TAGTACCCGCGTGTCATCATCGCGCGGCGAATACGTCCCTGCTCCTTGCACACACCGCCC 2024219

Query 1318 GTCA 1321

||||

Sbjct 2024220 GTCA 2024223

> gi|1214747492|ref|NZ_CP014862.1| Thermococcus profundus strain

DT 5432, complete sequence

Length=2005098

Score = 2383 bits (1290), Expect = 0.0

Identities = 1311/1321 (99%), Gaps = 1/1321 (0%)

Strand=Plus/Plus

Query 1 GTCCGACTAAGCCATGCGAGTCATGGGGCGCCTTGCGCGCACCGGCGGACGGCTCAGTAA 60

||||||||||||||||||||||||||||||||||||||||||||||||||||||||||||

Sbjct 408513 GTCCGACTAAGCCATGCGAGTCATGGGGCGCCTTGCGCGCACCGGCGGACGGCTCAGTAA 408572

Query 61 CACGTCGGTAACCTACCCTCGGGAGGGGGATAACCCCGGGAAACTGGGGCTAATCCCCCA 120

||||||||||||||||||||||||||||||||||||||||||||||||||||||||||||

Sbjct 408573 CACGTCGGTAACCTACCCTCGGGAGGGGGATAACCCCGGGAAACTGGGGCTAATCCCCCA 408632

Query 121 TAGGCCTGAGGTACTGGAAGGTCCTCAGGCCGAAAGGGGCTCTGCCCGCCCGAGGATGGG 180

||||||||||||||||||||||||||||||||||||||||| ||||||||||||||||||

Sbjct 408633 TAGGCCTGAGGTACTGGAAGGTCCTCAGGCCGAAAGGGGCTTTGCCCGCCCGAGGATGGG 408692

Query 181 CCGGCGGCCGATTAGGTAGTTGGTGGGGTAACGGCCCACCAAGCCGAAGATCGGTACGGG 240

||||||||||||||||||||||||||||||||||||||||||||||||||||||||||||

Sbjct 408693 CCGGCGGCCGATTAGGTAGTTGGTGGGGTAACGGCCCACCAAGCCGAAGATCGGTACGGG 408752

Query 241 CCATGAGAGTGGGAGCCCGGAGATGGACACTGAGACACGGGTCCAGGCCCTACGGGGCGC 300

||||||||||||||||||||||||||||||||||||||||||||||||||||||||||||

Sbjct 408753 CCATGAGAGTGGGAGCCCGGAGATGGACACTGAGACACGGGTCCAGGCCCTACGGGGCGC 408812

Query 301 AGCAGGCGCGAAACCTCCGCAATGCGGGCAACCGCGACGGGGGGACCCCCAGTGCCGTGG 360

||||||||||||||||||||||||||||||||||||||||||||||||||||||||||||

Sbjct 408813 AGCAGGCGCGAAACCTCCGCAATGCGGGCAACCGCGACGGGGGGACCCCCAGTGCCGTGG 408872

Query 361 CAACGCCACGGCTTTTCCGGAGTGTAAAAAGCTCCGGGAATAAGGGCTGGGCAAGGCCGG 420

|| ||||||||||||||||||||||| |||||||||||||||||||||||||||||||||

Sbjct 408873 CATCGCCACGGCTTTTCCGGAGTGTACAAAGCTCCGGGAATAAGGGCTGGGCAAGGCCGG 408932

Query 421 TGGCAGCCGCCGCGGTAATACCGGCGGCCCGAGTGGTGGCCGCTATTATTGGGCCTAAAG 480

||||||||||||||||||||||||||||||||||||||||||||||||||||||||||||

Sbjct 408933 TGGCAGCCGCCGCGGTAATACCGGCGGCCCGAGTGGTGGCCGCTATTATTGGGCCTAAAG 408992

Query 481 CGTCCGTAGCCGGGCCCGTAAGTCCCTGGCGAAATCCCACGGCTCAACCGTGGGGCTTGC 540

|||||||||||||||||||||||||||||||||||| |||||||||||||||||||||||

Sbjct 408993 CGTCCGTAGCCGGGCCCGTAAGTCCCTGGCGAAATCTCACGGCTCAACCGTGGGGCTTGC 409052

Query 541 TGGGGATACTGCGGGCCTTGGGACCGGGAGAGGCCGGGGGTACCCCTGGGGTAGGGGTGA 600

||||||||||||||||||||||||||||||||||||||||||||||||||||||||||||

Sbjct 409053 TGGGGATACTGCGGGCCTTGGGACCGGGAGAGGCCGGGGGTACCCCTGGGGTAGGGGTGA 409112

Query 601 AATCCTATAATCCCAGGGGGACCGCCAGTGGCGAAGGCGCCCGGCTGGAACGGGTCCGAC 660

|||||| |||||||||||||||||||||||||||||||||||||||||||||||||||||

Sbjct 409113 AATCCTGTAATCCCAGGGGGACCGCCAGTGGCGAAGGCGCCCGGCTGGAACGGGTCCGAC 409172

Query 661 GGTGAGGGACGAAGGCCAGGGGAGCGAACCGGATTAGATACCCGGGTAGTCCTGGCTGTA 720

||||||||||||||||||||||||||||||||||||||||||||||||||||||||||||

Sbjct 409173 GGTGAGGGACGAAGGCCAGGGGAGCGAACCGGATTAGATACCCGGGTAGTCCTGGCTGTA 409232

Query 721 AAGGATGCGGGCTAGGTGTCGGGCGAGCTTCGAGCTCGCCCGGTGCCGAAGGGAAGCCGT 780

||||||||||||||||||||||| ||||||||||||||||||||||||||||||||||||

Sbjct 409233 AAGGATGCGGGCTAGGTGTCGGGTGAGCTTCGAGCTCGCCCGGTGCCGAAGGGAAGCCGT 409292

Query 781 TAAGCCCGCCGCCTGGGGAGTACGGCCGCAAGGCTGAAACTTAAAGGAATTGGCGGGGGA 840

||||||||||||||||||||||||||||||||||||||||||||||||||||||||||||

Sbjct 409293 TAAGCCCGCCGCCTGGGGAGTACGGCCGCAAGGCTGAAACTTAAAGGAATTGGCGGGGGA 409352

Query 841 GCACTACAAGGGGTGGAGCGTGCGGTTTAATTGGATTCAACGCCGGGAACCTCACCGGGG 900

||||||||||||||||||||||||||||||||||||||||||||||||||||||||||||

Sbjct 409353 GCACTACAAGGGGTGGAGCGTGCGGTTTAATTGGATTCAACGCCGGGAACCTCACCGGGG 409412

Query 901 GCGACGGCAGGATGAAGGCCAGGCTGAAGGTCTTGCCGGACACGCCGAGAGGAGGTGCAT 960

||||||||||||||||||||||||||||||||||||||||||||||||||||||||||||

Sbjct 409413 GCGACGGCAGGATGAAGGCCAGGCTGAAGGTCTTGCCGGACACGCCGAGAGGAGGTGCAT 409472

Query 961 GGCCGCCGTCAGCTCGTACCGTGAGGCGTCCACTTAAGTGTGGTAACGAGCGAGACCCGC 1020

||||||||||||||||||||||||||||||||||||||||||||||||||||||||||||

Sbjct 409473 GGCCGCCGTCAGCTCGTACCGTGAGGCGTCCACTTAAGTGTGGTAACGAGCGAGACCCGC 409532

Query 1021 GCCCCCCAGTTGCCAGTCCTTCCCGCTGGGGAGGAGGCACTCTGGGGGGACCGCCGGCGA 1080

| |||||||||||||||||| |||||||||||||||||||||||||||||||||||||||

Sbjct 409533 G-CCCCCAGTTGCCAGTCCTCCCCGCTGGGGAGGAGGCACTCTGGGGGGACCGCCGGCGA 409591

Query 1081 TAAGCCGGAGGAAGGAGCGGGCGACGGTAGGTCAGTATGCCCCGAAACCCCCGGGCTACA 1140

||||||||||||||||||||||||||||||||||||||||||||||||||||||||||||

Sbjct 409592 TAAGCCGGAGGAAGGAGCGGGCGACGGTAGGTCAGTATGCCCCGAAACCCCCGGGCTACA 409651

Query 1141 CGCGCGCTACAATGGGCGGGACAATGGGATCCGACCCCGAAAGGGGAAGGGAATCCCCTA 1200

||||||||||||||||||||||||||||||||||||||||||||||||||||||||||||

Sbjct 409652 CGCGCGCTACAATGGGCGGGACAATGGGATCCGACCCCGAAAGGGGAAGGGAATCCCCTA 409711

Query 1201 AACCCGCCCCCAGTTCGGATCGCGGGCTGCAACTCGCCCGCGTGAAGCTGGAATCCCTAG 1260

|||||||||||||||||||| |||||||||||||||||||| ||||||||||||||||||

Sbjct 409712 AACCCGCCCCCAGTTCGGATTGCGGGCTGCAACTCGCCCGCATGAAGCTGGAATCCCTAG 409771

Query 1261 TACCCGCGTGTCATCATCGCGCGGCGAATACGTCCCTGCTCCTTGCACACACCGCCCGTC 1320

||||||||||||||||||||||||||||||||||||||||||||||||||||||||||||

Sbjct 409772 TACCCGCGTGTCATCATCGCGCGGCGAATACGTCCCTGCTCCTTGCACACACCGCCCGTC 409831

Query 1321 A 1321

|

Sbjct 409832 A 409832

> gi|240102057|ref|NC_012804.1| Thermococcus gammatolerans EJ3,

complete sequence

Length=2045438

Score = 2381 bits (1289), Expect = 0.0

Identities = 1313/1324 (99%), Gaps = 4/1324 (0%)

Strand=Plus/Minus

Query 1 GTCCGACTAAGCCATGCGAGTCATGGGGCGC-CT-TGCGCGCACCGGCGGACGGCTCAGT 58

||||||||||||||||||||||||||||||| || |||||||||||||||||||||||||

Sbjct 1950335 GTCCGACTAAGCCATGCGAGTCATGGGGCGCGCTCTGCGCGCACCGGCGGACGGCTCAGT 1950276

Query 59 AACACGTCGGTAACCTACCCTCGGGAGGGGGATAACCCCGGGAAACTGGGGCTAATCCCC 118

||||||||||||||||||||||||||||||||||||||||||||||||||||||||||||

Sbjct 1950275 AACACGTCGGTAACCTACCCTCGGGAGGGGGATAACCCCGGGAAACTGGGGCTAATCCCC 1950216

Query 119 CATAGGCCTGAGGTACTGGAAGGTCCTCAGGCCGAAAGGGGC-TCTGCCCGCCCGAGGAT 177

|||||||||||||||||||||||||||||||||||||||||| |||||||||||||||||

Sbjct 1950215 CATAGGCCTGAGGTACTGGAAGGTCCTCAGGCCGAAAGGGGCATCTGCCCGCCCGAGGAT 1950156

Query 178 GGGCCGGCGGCCGATTAGGTAGTTGGTGGGGTAACGGCCCACCAAGCCGAAGATCGGTAC 237

||||||||||||||||||||||||||||||||||||||||||||||||||||||||||||

Sbjct 1950155 GGGCCGGCGGCCGATTAGGTAGTTGGTGGGGTAACGGCCCACCAAGCCGAAGATCGGTAC 1950096

Query 238 GGGCCATGAGAGTGGGAGCCCGGAGATGGACACTGAGACACGGGTCCAGGCCCTACGGGG 297

||||||||||||||||||||||||||||||||||||||||||||||||||||||||||||

Sbjct 1950095 GGGCCATGAGAGTGGGAGCCCGGAGATGGACACTGAGACACGGGTCCAGGCCCTACGGGG 1950036

Query 298 CGCAGCAGGCGCGAAACCTCCGCAATGCGGGCAACCGCGACGGGGGGACCCCCAGTGCCG 357

||||||||||||||||||||||||||||||||||||||||||||||||||||||||||||

Sbjct 1950035 CGCAGCAGGCGCGAAACCTCCGCAATGCGGGCAACCGCGACGGGGGGACCCCCAGTGCCG 1949976

Query 358 TGGCAACGCCACGGCTTTTCCGGAGTGTAAAAAGCTCCGGGAATAAGGGCTGGGCAAGGC 417

||||| |||||||||||||||||||||||||||||||||||||||||||||||||||||

Sbjct 1949975 TGGCACAGCCACGGCTTTTCCGGAGTGTAAAAAGCTCCGGGAATAAGGGCTGGGCAAGGC 1949916

Query 418 CGGTGGCAGCCGCCGCGGTAATACCGGCGGCCCGAGTGGTGGCCGCTATTATTGGGCCTA 477

||||||||||||||||||||||||||||||||| ||||||||||||||||||||||||||

Sbjct 1949915 CGGTGGCAGCCGCCGCGGTAATACCGGCGGCCCAAGTGGTGGCCGCTATTATTGGGCCTA 1949856

Query 478 AAGCGTCCGTAGCCGGGCCCGTAAGTCCCTGGCGAAATCCCACGGCTCAACCGTGGGGCT 537

||||||||||||||||||||||||||||||||||||||||||||||||||||||||||||

Sbjct 1949855 AAGCGTCCGTAGCCGGGCCCGTAAGTCCCTGGCGAAATCCCACGGCTCAACCGTGGGGCT 1949796

Query 538 TGCTGGGGATACTGCGGGCCTTGGGACCGGGAGAGGCCGGGGGTACCCCTGGGGTAGGGG 597

||||||||||||||||||||||||||||||||||||||||||||||||||||||||||||

Sbjct 1949795 TGCTGGGGATACTGCGGGCCTTGGGACCGGGAGAGGCCGGGGGTACCCCTGGGGTAGGGG 1949736

Query 598 TGAAATCCTATAATCCCAGGGGGACCGCCAGTGGCGAAGGCGCCCGGCTGGAACGGGTCC 657

||||||||| ||||||||||||||||||||||||||||||||||||||||||||||||||

Sbjct 1949735 TGAAATCCTGTAATCCCAGGGGGACCGCCAGTGGCGAAGGCGCCCGGCTGGAACGGGTCC 1949676

Query 658 GACGGTGAGGGACGAAGGCCAGGGGAGCGAACCGGATTAGATACCCGGGTAGTCCTGGCT 717

||||||||||||||||||||||||||||||||||||||||||||||||||||||||||||

Sbjct 1949675 GACGGTGAGGGACGAAGGCCAGGGGAGCGAACCGGATTAGATACCCGGGTAGTCCTGGCT 1949616

Query 718 GTAAAGGATGCGGGCTAGGTGTCGGGCGAGCTTCGAGCTCGCCCGGTGCCGAAGGGAAGC 777

||||||||||||||||||||||||||||||||||||||||||||||||||||||||||||

Sbjct 1949615 GTAAAGGATGCGGGCTAGGTGTCGGGCGAGCTTCGAGCTCGCCCGGTGCCGAAGGGAAGC 1949556

Query 778 CGTTAAGCCCGCCGCCTGGGGAGTACGGCCGCAAGGCTGAAACTTAAAGGAATTGGCGGG 837

||||||||||||||||||||||||||||||||||||||||||||||||||||||||||||

Sbjct 1949555 CGTTAAGCCCGCCGCCTGGGGAGTACGGCCGCAAGGCTGAAACTTAAAGGAATTGGCGGG 1949496

Query 838 GGAGCACTACAAGGGGTGGAGCGTGCGGTTTAATTGGATTCAACGCCGGGAACCTCACCG 897

||||||||||||||||||||||||||||||||||||||||||||||||||||||||||||

Sbjct 1949495 GGAGCACTACAAGGGGTGGAGCGTGCGGTTTAATTGGATTCAACGCCGGGAACCTCACCG 1949436

Query 898 GGGGCGACGGCAGGATGAAGGCCAGGCTGAAGGTCTTGCCGGACACGCCGAGAGGAGGTG 957

|||||||||||||||||||||||||||||||||||||||||||| |||||||||||||||

Sbjct 1949435 GGGGCGACGGCAGGATGAAGGCCAGGCTGAAGGTCTTGCCGGACGCGCCGAGAGGAGGTG 1949376

Query 958 CATGGCCGCCGTCAGCTCGTACCGTGAGGCGTCCACTTAAGTGTGGTAACGAGCGAGACC 1017

||||||||||||||||||||||||||||||||||||||||||||||||||||||||||||

Sbjct 1949375 CATGGCCGCCGTCAGCTCGTACCGTGAGGCGTCCACTTAAGTGTGGTAACGAGCGAGACC 1949316

Query 1018 CGCGCCCCCCAGTTGCCAGTCCTTCCCGCTGGGGAGGAGGCACTCTGGGGGGACCGCCGG 1077

||||||||| ||||||||||||| ||||||||||||||||||||||||||||||||||||

Sbjct 1949315 CGCGCCCCC-AGTTGCCAGTCCTCCCCGCTGGGGAGGAGGCACTCTGGGGGGACCGCCGG 1949257

Query 1078 CGATAAGCCGGAGGAAGGAGCGGGCGACGGTAGGTCAGTATGCCCCGAAACCCCCGGGCT 1137

||||||||||||||||||||||||||||||||||||||||||||||||||||||||||||

Sbjct 1949256 CGATAAGCCGGAGGAAGGAGCGGGCGACGGTAGGTCAGTATGCCCCGAAACCCCCGGGCT 1949197

Query 1138 ACACGCGCGCTACAATGGGCGGGACAATGGGATCCGACCCCGAAAGGGGAAGGGAATCCC 1197

||||||||||||||||||||||||||||||||||||||||||||||||||||||||||||

Sbjct 1949196 ACACGCGCGCTACAATGGGCGGGACAATGGGATCCGACCCCGAAAGGGGAAGGGAATCCC 1949137

Query 1198 CTAAACCCGCCCCCAGTTCGGATCGCGGGCTGCAACTCGCCCGCGTGAAGCTGGAATCCC 1257

|||||||||||| |||||||||||||||||||||||||||||||||||||||||||||||

Sbjct 1949136 CTAAACCCGCCCTCAGTTCGGATCGCGGGCTGCAACTCGCCCGCGTGAAGCTGGAATCCC 1949077

Query 1258 TAGTACCCGCGTGTCATCATCGCGCGGCGAATACGTCCCTGCTCCTTGCACACACCGCCC 1317

||||||||||||||||||||||||||||||||||||||||||||||||||||||||||||

Sbjct 1949076 TAGTACCCGCGTGTCATCATCGCGCGGCGAATACGTCCCTGCTCCTTGCACACACCGCCC 1949017

Query 1318 GTCA 1321

||||

Sbjct 1949016 GTCA 1949013

> gi|1214774922|ref|NZ_CP014855.1| Thermococcus gorgonarius strain

W-12, complete genome

Length=1674122

Score = 2375 bits (1286), Expect = 0.0

Identities = 1312/1324 (99%), Gaps = 4/1324 (0%)

Strand=Plus/Plus

Query 1 GTCCGACTAAGCCATGCGAGTCATGGGGCGC-CT-TGCGCGCACCGGCGGACGGCTCAGT 58

||||||||||||||||||||||||||||||| || |||||||||||||||||||||||||

Sbjct 78868 GTCCGACTAAGCCATGCGAGTCATGGGGCGCGCTCTGCGCGCACCGGCGGACGGCTCAGT 78927

Query 59 AACACGTCGGTAACCTACCCTCGGGAGGGGGATAACCCCGGGAAACTGGGGCTAATCCCC 118

||||||||||||||||||||||||||||||||||||||||||||||||||||||||||||

Sbjct 78928 AACACGTCGGTAACCTACCCTCGGGAGGGGGATAACCCCGGGAAACTGGGGCTAATCCCC 78987

Query 119 CATAGGCCTGAGGTACTGGAAGGTCCTCAGGCCGAAAGGGGC-TCTGCCCGCCCGAGGAT 177

|||||||||||||||||||||||||||||||||||||||||| | |||||||||||||||

Sbjct 78988 CATAGGCCTGAGGTACTGGAAGGTCCTCAGGCCGAAAGGGGCTTTTGCCCGCCCGAGGAT 79047

Query 178 GGGCCGGCGGCCGATTAGGTAGTTGGTGGGGTAACGGCCCACCAAGCCGAAGATCGGTAC 237

||||||||||||||||||||||||||||||||||||||||||||||||||||||||||||

Sbjct 79048 GGGCCGGCGGCCGATTAGGTAGTTGGTGGGGTAACGGCCCACCAAGCCGAAGATCGGTAC 79107

Query 238 GGGCCATGAGAGTGGGAGCCCGGAGATGGACACTGAGACACGGGTCCAGGCCCTACGGGG 297

||||||||||||||||||||||||||||||||||||||||||||||||||||||||||||

Sbjct 79108 GGGCCATGAGAGTGGGAGCCCGGAGATGGACACTGAGACACGGGTCCAGGCCCTACGGGG 79167

Query 298 CGCAGCAGGCGCGAAACCTCCGCAATGCGGGCAACCGCGACGGGGGGACCCCCAGTGCCG 357

||||||||||||||||||||||||||||||| ||||||||||||||||||||||||||||

Sbjct 79168 CGCAGCAGGCGCGAAACCTCCGCAATGCGGGAAACCGCGACGGGGGGACCCCCAGTGCCG 79227

Query 358 TGGCAACGCCACGGCTTTTCCGGAGTGTAAAAAGCTCCGGGAATAAGGGCTGGGCAAGGC 417

||||| ||||||||||||||||||||||||||||||||||||||||||||||||||||||

Sbjct 79228 TGGCATCGCCACGGCTTTTCCGGAGTGTAAAAAGCTCCGGGAATAAGGGCTGGGCAAGGC 79287

Query 418 CGGTGGCAGCCGCCGCGGTAATACCGGCGGCCCGAGTGGTGGCCGCTATTATTGGGCCTA 477

||||||||||||||||||||||||||||||||||||||||||||||||||||||||||||

Sbjct 79288 CGGTGGCAGCCGCCGCGGTAATACCGGCGGCCCGAGTGGTGGCCGCTATTATTGGGCCTA 79347

Query 478 AAGCGTCCGTAGCCGGGCCCGTAAGTCCCTGGCGAAATCCCACGGCTCAACCGTGGGGCT 537

||||||||||||||||||||||||||||||||||||||| ||||||||||||||||||||

Sbjct 79348 AAGCGTCCGTAGCCGGGCCCGTAAGTCCCTGGCGAAATCTCACGGCTCAACCGTGGGGCT 79407

Query 538 TGCTGGGGATACTGCGGGCCTTGGGACCGGGAGAGGCCGGGGGTACCCCTGGGGTAGGGG 597

||||||||||||||||||||||||||||||||||||||||||||||||||||||||||||

Sbjct 79408 TGCTGGGGATACTGCGGGCCTTGGGACCGGGAGAGGCCGGGGGTACCCCTGGGGTAGGGG 79467

Query 598 TGAAATCCTATAATCCCAGGGGGACCGCCAGTGGCGAAGGCGCCCGGCTGGAACGGGTCC 657

||||||||||||||||||||||||||||||||||||||||||||||||||||||||||||

Sbjct 79468 TGAAATCCTATAATCCCAGGGGGACCGCCAGTGGCGAAGGCGCCCGGCTGGAACGGGTCC 79527

Query 658 GACGGTGAGGGACGAAGGCCAGGGGAGCGAACCGGATTAGATACCCGGGTAGTCCTGGCT 717

|||||||||||||||||||||||||||| |||||||||||||||||||||||||||||||

Sbjct 79528 GACGGTGAGGGACGAAGGCCAGGGGAGCAAACCGGATTAGATACCCGGGTAGTCCTGGCT 79587

Query 718 GTAAAGGATGCGGGCTAGGTGTCGGGCGAGCTTCGAGCTCGCCCGGTGCCGAAGGGAAGC 777

||||||||||||||||||||||||||||||||||||||||||||||||||| ||||||||

Sbjct 79588 GTAAAGGATGCGGGCTAGGTGTCGGGCGAGCTTCGAGCTCGCCCGGTGCCGGAGGGAAGC 79647

Query 778 CGTTAAGCCCGCCGCCTGGGGAGTACGGCCGCAAGGCTGAAACTTAAAGGAATTGGCGGG 837

||||||||||||||||||||||||||||||||||||||||||||||||||||||||||||

Sbjct 79648 CGTTAAGCCCGCCGCCTGGGGAGTACGGCCGCAAGGCTGAAACTTAAAGGAATTGGCGGG 79707

Query 838 GGAGCACTACAAGGGGTGGAGCGTGCGGTTTAATTGGATTCAACGCCGGGAACCTCACCG 897

||||||||||||||||||||||||||||||||||||||||||||||||||||||||||||

Sbjct 79708 GGAGCACTACAAGGGGTGGAGCGTGCGGTTTAATTGGATTCAACGCCGGGAACCTCACCG 79767

Query 898 GGGGCGACGGCAGGATGAAGGCCAGGCTGAAGGTCTTGCCGGACACGCCGAGAGGAGGTG 957

||||||||||||||||||||||||||||||||||||||||||||||||||||||||||||

Sbjct 79768 GGGGCGACGGCAGGATGAAGGCCAGGCTGAAGGTCTTGCCGGACACGCCGAGAGGAGGTG 79827

Query 958 CATGGCCGCCGTCAGCTCGTACCGTGAGGCGTCCACTTAAGTGTGGTAACGAGCGAGACC 1017

||||||||||||||||||||||||||||||||||||||||||||||||||||||||||||

Sbjct 79828 CATGGCCGCCGTCAGCTCGTACCGTGAGGCGTCCACTTAAGTGTGGTAACGAGCGAGACC 79887

Query 1018 CGCGCCCCCCAGTTGCCAGTCCTTCCCGCTGGGGAGGAGGCACTCTGGGGGGACCGCCGG 1077

|||| |||||||||||||||||| ||||||||||||||||||||||||||||||||||||

Sbjct 79888 CGCG-CCCCCAGTTGCCAGTCCTCCCCGCTGGGGAGGAGGCACTCTGGGGGGACCGCCGG 79946

Query 1078 CGATAAGCCGGAGGAAGGAGCGGGCGACGGTAGGTCAGTATGCCCCGAAACCCCCGGGCT 1137

||||||||||||||||||||||||||||||||||||||||||||||||||||||||||||

Sbjct 79947 CGATAAGCCGGAGGAAGGAGCGGGCGACGGTAGGTCAGTATGCCCCGAAACCCCCGGGCT 80006

Query 1138 ACACGCGCGCTACAATGGGCGGGACAATGGGATCCGACCCCGAAAGGGGAAGGGAATCCC 1197

||||||||||||||||||||||||||||||||||||||||||||||||||||||||||||

Sbjct 80007 ACACGCGCGCTACAATGGGCGGGACAATGGGATCCGACCCCGAAAGGGGAAGGGAATCCC 80066

Query 1198 CTAAACCCGCCCCCAGTTCGGATCGCGGGCTGCAACTCGCCCGCGTGAAGCTGGAATCCC 1257

|||||||||||| |||||||||||||||||||||||||||||||||||||||||||||||

Sbjct 80067 CTAAACCCGCCCTCAGTTCGGATCGCGGGCTGCAACTCGCCCGCGTGAAGCTGGAATCCC 80126

Query 1258 TAGTACCCGCGTGTCATCATCGCGCGGCGAATACGTCCCTGCTCCTTGCACACACCGCCC 1317

||||||||||||||||||||||||||||||||||||||||||||||||||||||||||||

Sbjct 80127 TAGTACCCGCGTGTCATCATCGCGCGGCGAATACGTCCCTGCTCCTTGCACACACCGCCC 80186

Query 1318 GTCA 1321

||||

Sbjct 80187 GTCA 80190

> gi|1011478452|ref|NZ_CP007140.1| Thermococcus guaymasensis DSM

11113 chromosome, complete genome

Length=1920914

Score = 2375 bits (1286), Expect = 0.0

Identities = 1312/1324 (99%), Gaps = 4/1324 (0%)

Strand=Plus/Plus

Query 1 GTCCGACTAAGCCATGCGAGTCATGGGGCGC-CT-TGCGCGCACCGGCGGACGGCTCAGT 58

||||||||||||||||||||||||||||||| || |||||||||||||||||||||||||

Sbjct 942548 GTCCGACTAAGCCATGCGAGTCATGGGGCGCGCTCTGCGCGCACCGGCGGACGGCTCAGT 942607

Query 59 AACACGTCGGTAACCTACCCTCGGGAGGGGGATAACCCCGGGAAACTGGGGCTAATCCCC 118

||||||||||||||||||||||||||||||||||||||||||||||||||||||||||||

Sbjct 942608 AACACGTCGGTAACCTACCCTCGGGAGGGGGATAACCCCGGGAAACTGGGGCTAATCCCC 942667

Query 119 CATAGGCCTGAGGTACTGGAAGGTCCTCAGGCCGAAAGGGGC-TCTGCCCGCCCGAGGAT 177

|||||||||||||||||||||||||||||||||||||||||| | |||||||||||||||

Sbjct 942668 CATAGGCCTGAGGTACTGGAAGGTCCTCAGGCCGAAAGGGGCTTTTGCCCGCCCGAGGAT 942727

Query 178 GGGCCGGCGGCCGATTAGGTAGTTGGTGGGGTAACGGCCCACCAAGCCGAAGATCGGTAC 237

||||||||||||||||||||||||||||||||||||||||||||||||||||||||||||

Sbjct 942728 GGGCCGGCGGCCGATTAGGTAGTTGGTGGGGTAACGGCCCACCAAGCCGAAGATCGGTAC 942787

Query 238 GGGCCATGAGAGTGGGAGCCCGGAGATGGACACTGAGACACGGGTCCAGGCCCTACGGGG 297

||||||||||||||||||||||||||||||||||||||||||||||||||||||||||||

Sbjct 942788 GGGCCATGAGAGTGGGAGCCCGGAGATGGACACTGAGACACGGGTCCAGGCCCTACGGGG 942847

Query 298 CGCAGCAGGCGCGAAACCTCCGCAATGCGGGCAACCGCGACGGGGGGACCCCCAGTGCCG 357

||||||||||||||||||||||||||||||| ||||||||||||||||||||||||||||

Sbjct 942848 CGCAGCAGGCGCGAAACCTCCGCAATGCGGGAAACCGCGACGGGGGGACCCCCAGTGCCG 942907

Query 358 TGGCAACGCCACGGCTTTTCCGGAGTGTAAAAAGCTCCGGGAATAAGGGCTGGGCAAGGC 417

||||| ||||||||||||||||||||||||||||||||||||||||||||||||||||||

Sbjct 942908 TGGCATCGCCACGGCTTTTCCGGAGTGTAAAAAGCTCCGGGAATAAGGGCTGGGCAAGGC 942967

Query 418 CGGTGGCAGCCGCCGCGGTAATACCGGCGGCCCGAGTGGTGGCCGCTATTATTGGGCCTA 477

||||||||||||||||||||||||||||||||| ||||||||||||||||||||||||||

Sbjct 942968 CGGTGGCAGCCGCCGCGGTAATACCGGCGGCCCAAGTGGTGGCCGCTATTATTGGGCCTA 943027

Query 478 AAGCGTCCGTAGCCGGGCCCGTAAGTCCCTGGCGAAATCCCACGGCTCAACCGTGGGGCT 537

||||||||||||||||||||||||||||||||||||||||||||||||||||||||||||

Sbjct 943028 AAGCGTCCGTAGCCGGGCCCGTAAGTCCCTGGCGAAATCCCACGGCTCAACCGTGGGGCT 943087

Query 538 TGCTGGGGATACTGCGGGCCTTGGGACCGGGAGAGGCCGGGGGTACCCCTGGGGTAGGGG 597

||||||||||||||||||||||||||||||||||||||||||||||||||||||||||||

Sbjct 943088 TGCTGGGGATACTGCGGGCCTTGGGACCGGGAGAGGCCGGGGGTACCCCTGGGGTAGGGG 943147

Query 598 TGAAATCCTATAATCCCAGGGGGACCGCCAGTGGCGAAGGCGCCCGGCTGGAACGGGTCC 657

||||||||| ||||||||||||||||||||||||||||||||||||||||||||||||||

Sbjct 943148 TGAAATCCTGTAATCCCAGGGGGACCGCCAGTGGCGAAGGCGCCCGGCTGGAACGGGTCC 943207

Query 658 GACGGTGAGGGACGAAGGCCAGGGGAGCGAACCGGATTAGATACCCGGGTAGTCCTGGCT 717

||||||||||||||||||||||||||||||||||||||||||||||||||||||||||||

Sbjct 943208 GACGGTGAGGGACGAAGGCCAGGGGAGCGAACCGGATTAGATACCCGGGTAGTCCTGGCT 943267

Query 718 GTAAAGGATGCGGGCTAGGTGTCGGGCGAGCTTCGAGCTCGCCCGGTGCCGAAGGGAAGC 777

||||||||||||||||||||||||||||||||||||||||||||||||||||||||||||

Sbjct 943268 GTAAAGGATGCGGGCTAGGTGTCGGGCGAGCTTCGAGCTCGCCCGGTGCCGAAGGGAAGC 943327

Query 778 CGTTAAGCCCGCCGCCTGGGGAGTACGGCCGCAAGGCTGAAACTTAAAGGAATTGGCGGG 837

||||||||||||||||||||||||||||||||||||||||||||||||||||||||||||

Sbjct 943328 CGTTAAGCCCGCCGCCTGGGGAGTACGGCCGCAAGGCTGAAACTTAAAGGAATTGGCGGG 943387

Query 838 GGAGCACTACAAGGGGTGGAGCGTGCGGTTTAATTGGATTCAACGCCGGGAACCTCACCG 897

||||||||||||||||||||||||||||||||||||||||||||||||||||||||||||

Sbjct 943388 GGAGCACTACAAGGGGTGGAGCGTGCGGTTTAATTGGATTCAACGCCGGGAACCTCACCG 943447

Query 898 GGGGCGACGGCAGGATGAAGGCCAGGCTGAAGGTCTTGCCGGACACGCCGAGAGGAGGTG 957

|||||||||||||||||||||||||||||||||||||||||||| |||||||||||||||

Sbjct 943448 GGGGCGACGGCAGGATGAAGGCCAGGCTGAAGGTCTTGCCGGACGCGCCGAGAGGAGGTG 943507

Query 958 CATGGCCGCCGTCAGCTCGTACCGTGAGGCGTCCACTTAAGTGTGGTAACGAGCGAGACC 1017

||||||||||||||||||||||||||||||||||||||||||||||||||||||||||||

Sbjct 943508 CATGGCCGCCGTCAGCTCGTACCGTGAGGCGTCCACTTAAGTGTGGTAACGAGCGAGACC 943567

Query 1018 CGCGCCCCCCAGTTGCCAGTCCTTCCCGCTGGGGAGGAGGCACTCTGGGGGGACCGCCGG 1077

|||| |||||||||||||||||| ||||||||||||||||||||||||||||||||||||

Sbjct 943568 CGCG-CCCCCAGTTGCCAGTCCTCCCCGCTGGGGAGGAGGCACTCTGGGGGGACCGCCGG 943626

Query 1078 CGATAAGCCGGAGGAAGGAGCGGGCGACGGTAGGTCAGTATGCCCCGAAACCCCCGGGCT 1137

||||||||||||||||||||||||||||||||||||||||||||||||||||||||||||

Sbjct 943627 CGATAAGCCGGAGGAAGGAGCGGGCGACGGTAGGTCAGTATGCCCCGAAACCCCCGGGCT 943686

Query 1138 ACACGCGCGCTACAATGGGCGGGACAATGGGATCCGACCCCGAAAGGGGAAGGGAATCCC 1197

||||||||||||||||||||||||||||||||||||||||||||||||||||||||||||

Sbjct 943687 ACACGCGCGCTACAATGGGCGGGACAATGGGATCCGACCCCGAAAGGGGAAGGGAATCCC 943746

Query 1198 CTAAACCCGCCCCCAGTTCGGATCGCGGGCTGCAACTCGCCCGCGTGAAGCTGGAATCCC 1257

|||||||||||| |||||||||||||||||||||||||||||||||||||||||||||||

Sbjct 943747 CTAAACCCGCCCTCAGTTCGGATCGCGGGCTGCAACTCGCCCGCGTGAAGCTGGAATCCC 943806

Query 1258 TAGTACCCGCGTGTCATCATCGCGCGGCGAATACGTCCCTGCTCCTTGCACACACCGCCC 1317

||||||||||||||||||||||||||||||||||||||||||||||||||||||||||||

Sbjct 943807 TAGTACCCGCGTGTCATCATCGCGCGGCGAATACGTCCCTGCTCCTTGCACACACCGCCC 943866

Query 1318 GTCA 1321

||||

Sbjct 943867 GTCA 943870

> gi|757139166|ref|NZ_CP007264.1| Thermococcus nautili strain 30-1

chromosome, complete genome

Length=1976356

Score = 2362 bits (1279), Expect = 0.0

Identities = 1309/1323 (99%), Gaps = 3/1323 (0%)

Strand=Plus/Plus

Query 1 GTCCGACTAAGCCATGCGAGTCATGGGGCGC-CT-TGCGCGCACCGGCGGACGGCTCAGT 58

||||||||||||||||||||||||||||||| || |||||||||||||||||||||||||

Sbjct 1093107 GTCCGACTAAGCCATGCGAGTCATGGGGCGCGCTCTGCGCGCACCGGCGGACGGCTCAGT 1093166

Query 59 AACACGTCGGTAACCTACCCTCGGGAGGGGGATAACCCCGGGAAACTGGGGCTAATCCCC 118

||||||||||||||||||||||||||||||||||||||||||||||||||||||||||||

Sbjct 1093167 AACACGTCGGTAACCTACCCTCGGGAGGGGGATAACCCCGGGAAACTGGGGCTAATCCCC 1093226

Query 119 CATAGGCCTGAGGTACTGGAAGGTCCTCAGGCCGAAAGGGGCTCTGCCCGCCCGAGGATG 178

|||||||||| ||||||||||||||| |||||||||||||||||||||||||||||||||

Sbjct 1093227 CATAGGCCTGGGGTACTGGAAGGTCCCCAGGCCGAAAGGGGCTCTGCCCGCCCGAGGATG 1093286

Query 179 GGCCGGCGGCCGATTAGGTAGTTGGTGGGGTAACGGCCCACCAAGCCGAAGATCGGTACG 238

||||||||||||||||||||||||||||||||||||||||||||||||||||||||||||

Sbjct 1093287 GGCCGGCGGCCGATTAGGTAGTTGGTGGGGTAACGGCCCACCAAGCCGAAGATCGGTACG 1093346

Query 239 GGCCATGAGAGTGGGAGCCCGGAGATGGACACTGAGACACGGGTCCAGGCCCTACGGGGC 298

||||||||||||||||||||||||||||||||||||||||||||||||||||||||||||

Sbjct 1093347 GGCCATGAGAGTGGGAGCCCGGAGATGGACACTGAGACACGGGTCCAGGCCCTACGGGGC 1093406

Query 299 GCAGCAGGCGCGAAACCTCCGCAATGCGGGCAACCGCGACGGGGGGACCCCCAGTGCCGT 358

|||||||||||||||||||||||||||||| |||||||||||||||||||||||||||||

Sbjct 1093407 GCAGCAGGCGCGAAACCTCCGCAATGCGGGAAACCGCGACGGGGGGACCCCCAGTGCCGT 1093466

Query 359 GGCAACGCCACGGCTTTTCCGGAGTGTAAAAAGCTCCGGGAATAAGGGCTGGGCAAGGCC 418

|||| ||||||||||||||||||||||||||||||||||||||||||||||||||||||

Sbjct 1093467 GGCACAGCCACGGCTTTTCCGGAGTGTAAAAAGCTCCGGGAATAAGGGCTGGGCAAGGCC 1093526

Query 419 GGTGGCAGCCGCCGCGGTAATACCGGCGGCCCGAGTGGTGGCCGCTATTATTGGGCCTAA 478

|||||||||||||||||||||||||||||||| |||||||||||||||||||||||||||

Sbjct 1093527 GGTGGCAGCCGCCGCGGTAATACCGGCGGCCCAAGTGGTGGCCGCTATTATTGGGCCTAA 1093586

Query 479 AGCGTCCGTAGCCGGGCCCGTAAGTCCCTGGCGAAATCCCACGGCTCAACCGTGGGGCTT 538

||||||||||||||||||||||||||||||||||||||||||||||||||||||||||||

Sbjct 1093587 AGCGTCCGTAGCCGGGCCCGTAAGTCCCTGGCGAAATCCCACGGCTCAACCGTGGGGCTT 1093646

Query 539 GCTGGGGATACTGCGGGCCTTGGGACCGGGAGAGGCCGGGGGTACCCCTGGGGTAGGGGT 598

||||||||||||||||||||||||||||||||||||||||||||||||||||||||||||

Sbjct 1093647 GCTGGGGATACTGCGGGCCTTGGGACCGGGAGAGGCCGGGGGTACCCCTGGGGTAGGGGT 1093706

Query 599 GAAATCCTATAATCCCAGGGGGACCGCCAGTGGCGAAGGCGCCCGGCTGGAACGGGTCCG 658

||||||||||||||||||||||||||||||||||||||||||||||||||||||||||||

Sbjct 1093707 GAAATCCTATAATCCCAGGGGGACCGCCAGTGGCGAAGGCGCCCGGCTGGAACGGGTCCG 1093766

Query 659 ACGGTGAGGGACGAAGGCCAGGGGAGCGAACCGGATTAGATACCCGGGTAGTCCTGGCTG 718

||||||||||||||||||||||||||||||||||||||||||||||||||||||||||||

Sbjct 1093767 ACGGTGAGGGACGAAGGCCAGGGGAGCGAACCGGATTAGATACCCGGGTAGTCCTGGCTG 1093826

Query 719 TAAAGGATGCGGGCTAGGTGTCGGGCGAGCTTCGAGCTCGCCCGGTGCCGAAGGGAAGCC 778

|||||||||||||||||||||||||||||||||||||||||||||||||| |||||||||

Sbjct 1093827 TAAAGGATGCGGGCTAGGTGTCGGGCGAGCTTCGAGCTCGCCCGGTGCCGGAGGGAAGCC 1093886

Query 779 GTTAAGCCCGCCGCCTGGGGAGTACGGCCGCAAGGCTGAAACTTAAAGGAATTGGCGGGG 838

||||||||||||||||||||||||||||||||||||||||||||||||||||||||||||

Sbjct 1093887 GTTAAGCCCGCCGCCTGGGGAGTACGGCCGCAAGGCTGAAACTTAAAGGAATTGGCGGGG 1093946

Query 839 GAGCACTACAAGGGGTGGAGCGTGCGGTTTAATTGGATTCAACGCCGGGAACCTCACCGG 898

||||||||||||||||||||||||||||||||||||||||||||||||||||||||||||

Sbjct 1093947 GAGCACTACAAGGGGTGGAGCGTGCGGTTTAATTGGATTCAACGCCGGGAACCTCACCGG 1094006

Query 899 GGGCGACGGCAGGATGAAGGCCAGGCTGAAGGTCTTGCCGGACACGCCGAGAGGAGGTGC 958

||||||||||||||||||||||||||||||||||||||||||| ||||||||||||||||

Sbjct 1094007 GGGCGACGGCAGGATGAAGGCCAGGCTGAAGGTCTTGCCGGACGCGCCGAGAGGAGGTGC 1094066

Query 959 ATGGCCGCCGTCAGCTCGTACCGTGAGGCGTCCACTTAAGTGTGGTAACGAGCGAGACCC 1018

||||||||||||||||||||||||||||||||||||||||||||||||||||||||||||

Sbjct 1094067 ATGGCCGCCGTCAGCTCGTACCGTGAGGCGTCCACTTAAGTGTGGTAACGAGCGAGACCC 1094126

Query 1019 GCGCCCCCCAGTTGCCAGTCCTTCCCGCTGGGGAGGAGGCACTCTGGGGGGACCGCCGGC 1078

||| |||||||||||||||||| |||||||||||||||||||||||||||||||||||||

Sbjct 1094127 GCG-CCCCCAGTTGCCAGTCCTCCCCGCTGGGGAGGAGGCACTCTGGGGGGACCGCCGGC 1094185

Query 1079 GATAAGCCGGAGGAAGGAGCGGGCGACGGTAGGTCAGTATGCCCCGAAACCCCCGGGCTA 1138

||||||||||||||||||||||||||||||||||||||||||||||||||||||||||||

Sbjct 1094186 GATAAGCCGGAGGAAGGAGCGGGCGACGGTAGGTCAGTATGCCCCGAAACCCCCGGGCTA 1094245

Query 1139 CACGCGCGCTACAATGGGCGGGACAATGGGATCCGACCCCGAAAGGGGAAGGGAATCCCC 1198

||||||||||||||||||||||||||||||| ||||||||||||||||||||||||||||

Sbjct 1094246 CACGCGCGCTACAATGGGCGGGACAATGGGAACCGACCCCGAAAGGGGAAGGGAATCCCC 1094305

Query 1199 TAAACCCGCCCCCAGTTCGGATCGCGGGCTGCAACTCGCCCGCGTGAAGCTGGAATCCCT 1258

||||||||||| ||||||||||||||||||||||||||||||||||||||||||||||||

Sbjct 1094306 TAAACCCGCCCTCAGTTCGGATCGCGGGCTGCAACTCGCCCGCGTGAAGCTGGAATCCCT 1094365

Query 1259 AGTACCCGCGTGTCATCATCGCGCGGCGAATACGTCCCTGCTCCTTGCACACACCGCCCG 1318

||||||||||||||||||||||||||||||||||||||||||||||||||||||||||||

Sbjct 1094366 AGTACCCGCGTGTCATCATCGCGCGGCGAATACGTCCCTGCTCCTTGCACACACCGCCCG 1094425

Query 1319 TCA 1321

|||

Sbjct 1094426 TCA 1094428

> gi|390960176|ref|NC_018015.1| Thermococcus cleftensis strain

CL1, complete genome

Length=1950313

Score = 2362 bits (1279), Expect = 0.0

Identities = 1309/1323 (99%), Gaps = 4/1323 (0%)

Strand=Plus/Plus

Query 1 GTCCGACTAAGCCATGCGAGTCATGGGGCGC-CT-TGCGCGCACCGGCGGACGGCTCAGT 58

||||||||||||||||||||||||||||||| || |||||||||||||||||||||||||

Sbjct 1280901 GTCCGACTAAGCCATGCGAGTCATGGGGCGCGCTCTGCGCGCACCGGCGGACGGCTCAGT 1280960

Query 59 AACACGTCGGTAACCTACCCTCGGGAGGGGGATAACCCCGGGAAACTGGGGCTAATCCCC 118

||||||||||||||||||||||||||||||||||||||||||||||||||||||||||||

Sbjct 1280961 AACACGTCGGTAACCTACCCTCGGGAGGGGGATAACCCCGGGAAACTGGGGCTAATCCCC 1281020

Query 119 CATAGGCCTGAGGTACTGGAAGGTCCTCAGGCCGAAAGGGGCTCTGCCCGCCCGAGGATG 178

|||||||||||||||||||||||||||||||||||||||| | | |||||||||||||

Sbjct 1281021 CATAGGCCTGAGGTACTGGAAGGTCCTCAGGCCGAAAGGGCGTAAG-CCGCCCGAGGATG 1281079

Query 179 GGCCGGCGGCCGATTAGGTAGTTGGTGGGGTAACGGCCCACCAAGCCGAAGATCGGTACG 238

||||||||||||||||||||||||||||||||||||||||||||||||||||||||||||

Sbjct 1281080 GGCCGGCGGCCGATTAGGTAGTTGGTGGGGTAACGGCCCACCAAGCCGAAGATCGGTACG 1281139

Query 239 GGCCATGAGAGTGGGAGCCCGGAGATGGACACTGAGACACGGGTCCAGGCCCTACGGGGC 298

||||||||||||||||||||||||||||||||||||||||||||||||||||||||||||

Sbjct 1281140 GGCCATGAGAGTGGGAGCCCGGAGATGGACACTGAGACACGGGTCCAGGCCCTACGGGGC 1281199

Query 299 GCAGCAGGCGCGAAACCTCCGCAATGCGGGCAACCGCGACGGGGGGACCCCCAGTGCCGT 358

|||||||||||||||||||||||||||||| |||||||||||||||||||||||||||||

Sbjct 1281200 GCAGCAGGCGCGAAACCTCCGCAATGCGGGAAACCGCGACGGGGGGACCCCCAGTGCCGT 1281259

Query 359 GGCAACGCCACGGCTTTTCCGGAGTGTAAAAAGCTCCGGGAATAAGGGCTGGGCAAGGCC 418

|||| ||||||||||||||||||||||||| |||||||||||||||||||||||||||||

Sbjct 1281260 GGCATCGCCACGGCTTTTCCGGAGTGTAAAGAGCTCCGGGAATAAGGGCTGGGCAAGGCC 1281319

Query 419 GGTGGCAGCCGCCGCGGTAATACCGGCGGCCCGAGTGGTGGCCGCTATTATTGGGCCTAA 478

||||||||||||||||||||||||||||||||||||||||||||||||||||||||||||

Sbjct 1281320 GGTGGCAGCCGCCGCGGTAATACCGGCGGCCCGAGTGGTGGCCGCTATTATTGGGCCTAA 1281379

Query 479 AGCGTCCGTAGCCGGGCCCGTAAGTCCCTGGCGAAATCCCACGGCTCAACCGTGGGGCTT 538

||||||||||||||||||||||||||||||||||||||||||||||||||||||||||||

Sbjct 1281380 AGCGTCCGTAGCCGGGCCCGTAAGTCCCTGGCGAAATCCCACGGCTCAACCGTGGGGCTT 1281439

Query 539 GCTGGGGATACTGCGGGCCTTGGGACCGGGAGAGGCCGGGGGTACCCCTGGGGTAGGGGT 598

||||||||||||||||||||||||||||||||||||||||||||| ||||||||||||||

Sbjct 1281440 GCTGGGGATACTGCGGGCCTTGGGACCGGGAGAGGCCGGGGGTACTCCTGGGGTAGGGGT 1281499

Query 599 GAAATCCTATAATCCCAGGGGGACCGCCAGTGGCGAAGGCGCCCGGCTGGAACGGGTCCG 658

||||||||||||||||||| ||||||||||||||||||||||||||||||||||||||||

Sbjct 1281500 GAAATCCTATAATCCCAGGAGGACCGCCAGTGGCGAAGGCGCCCGGCTGGAACGGGTCCG 1281559

Query 659 ACGGTGAGGGACGAAGGCCAGGGGAGCGAACCGGATTAGATACCCGGGTAGTCCTGGCTG 718

||||||||||||||||||||||||||||||||||||||||||||||||||||||||||||

Sbjct 1281560 ACGGTGAGGGACGAAGGCCAGGGGAGCGAACCGGATTAGATACCCGGGTAGTCCTGGCTG 1281619

Query 719 TAAAGGATGCGGGCTAGGTGTCGGGCGAGCTTCGAGCTCGCCCGGTGCCGAAGGGAAGCC 778

||||||||||||||||||||||||||||||||||||||||||||||||||||||||||||

Sbjct 1281620 TAAAGGATGCGGGCTAGGTGTCGGGCGAGCTTCGAGCTCGCCCGGTGCCGAAGGGAAGCC 1281679

Query 779 GTTAAGCCCGCCGCCTGGGGAGTACGGCCGCAAGGCTGAAACTTAAAGGAATTGGCGGGG 838

||||||||||||||||||||||||||||||||||||||||||||||||||||||||||||

Sbjct 1281680 GTTAAGCCCGCCGCCTGGGGAGTACGGCCGCAAGGCTGAAACTTAAAGGAATTGGCGGGG 1281739

Query 839 GAGCACTACAAGGGGTGGAGCGTGCGGTTTAATTGGATTCAACGCCGGGAACCTCACCGG 898

||||||||||||||||||||||||||||||||||||||||||||||||||||||||||||

Sbjct 1281740 GAGCACTACAAGGGGTGGAGCGTGCGGTTTAATTGGATTCAACGCCGGGAACCTCACCGG 1281799

Query 899 GGGCGACGGCAGGATGAAGGCCAGGCTGAAGGTCTTGCCGGACACGCCGAGAGGAGGTGC 958

||||||||||||||||||||||||||||||||||||||||||||||||||||||||||||

Sbjct 1281800 GGGCGACGGCAGGATGAAGGCCAGGCTGAAGGTCTTGCCGGACACGCCGAGAGGAGGTGC 1281859

Query 959 ATGGCCGCCGTCAGCTCGTACCGTGAGGCGTCCACTTAAGTGTGGTAACGAGCGAGACCC 1018

||||||||||||||||||||||||||||||||||||||||||||||||||||||||||||

Sbjct 1281860 ATGGCCGCCGTCAGCTCGTACCGTGAGGCGTCCACTTAAGTGTGGTAACGAGCGAGACCC 1281919

Query 1019 GCGCCCCCCAGTTGCCAGTCCTTCCCGCTGGGGAGGAGGCACTCTGGGGGGACCGCCGGC 1078

||| |||||||||||||||||| |||||||||||||||||||||||||||||||||||||

Sbjct 1281920 GCG-CCCCCAGTTGCCAGTCCTCCCCGCTGGGGAGGAGGCACTCTGGGGGGACCGCCGGC 1281978

Query 1079 GATAAGCCGGAGGAAGGAGCGGGCGACGGTAGGTCAGTATGCCCCGAAACCCCCGGGCTA 1138

||||||||||||||||||||||||||||||||||||||||||||||||||||||||||||

Sbjct 1281979 GATAAGCCGGAGGAAGGAGCGGGCGACGGTAGGTCAGTATGCCCCGAAACCCCCGGGCTA 1282038

Query 1139 CACGCGCGCTACAATGGGCGGGACAATGGGATCCGACCCCGAAAGGGGAAGGGAATCCCC 1198

||||||||||||||||||||||||||||||||||||||||||||||||||||||||||||

Sbjct 1282039 CACGCGCGCTACAATGGGCGGGACAATGGGATCCGACCCCGAAAGGGGAAGGGAATCCCC 1282098

Query 1199 TAAACCCGCCCCCAGTTCGGATCGCGGGCTGCAACTCGCCCGCGTGAAGCTGGAATCCCT 1258

||||||||||||||||||||||||||||||||||||||||||||||||||||||||||||

Sbjct 1282099 TAAACCCGCCCCCAGTTCGGATCGCGGGCTGCAACTCGCCCGCGTGAAGCTGGAATCCCT 1282158

Query 1259 AGTACCCGCGTGTCATCATCGCGCGGCGAATACGTCCCTGCTCCTTGCACACACCGCCCG 1318

||||||||||||||||||||||||||||||||||||||||||||||||||||||||||||

Sbjct 1282159 AGTACCCGCGTGTCATCATCGCGCGGCGAATACGTCCCTGCTCCTTGCACACACCGCCCG 1282218

Query 1319 TCA 1321

|||

Sbjct 1282219 TCA 1282221

> gi|910102422|ref|NZ_CP008887.1| Thermococcus eurythermalis strain

A501 chromosome, complete genome

Length=2122535

Score = 2335 bits (1264), Expect = 0.0

Identities = 1304/1323 (99%), Gaps = 3/1323 (0%)

Strand=Plus/Minus

Query 1 GTCCGACTAAGCCATGCGAGTCATGGGGCGC-CT-TGCGCGCACCGGCGGACGGCTCAGT 58

||||||||||||||||||||||||||||||| || |||||||||||||||||||||||||

Sbjct 176781 GTCCGACTAAGCCATGCGAGTCATGGGGCGCGCTCTGCGCGCACCGGCGGACGGCTCAGT 176722

Query 59 AACACGTCGGTAACCTACCCTCGGGAGGGGGATAACCCCGGGAAACTGGGGCTAATCCCC 118

||||||||||||||||||||||||||||||||||||||||||||||||||||||||||||

Sbjct 176721 AACACGTCGGTAACCTACCCTCGGGAGGGGGATAACCCCGGGAAACTGGGGCTAATCCCC 176662

Query 119 CATAGGCCTGAGGTACTGGAAGGTCCTCAGGCCGAAAGGGGCTCTGCCCGCCCGAGGATG 178

|||||||||| ||||||||||||||| |||||||||||||||||||||||||||||||||

Sbjct 176661 CATAGGCCTGGGGTACTGGAAGGTCCCCAGGCCGAAAGGGGCTCTGCCCGCCCGAGGATG 176602

Query 179 GGCCGGCGGCCGATTAGGTAGTTGGTGGGGTAACGGCCCACCAAGCCGAAGATCGGTACG 238

|||||||||||||||||||||||||||||||||||||||||||||||||||||| |||||

Sbjct 176601 GGCCGGCGGCCGATTAGGTAGTTGGTGGGGTAACGGCCCACCAAGCCGAAGATCCGTACG 176542

Query 239 GGCCATGAGAGTGGGAGCCCGGAGATGGACACTGAGACACGGGTCCAGGCCCTACGGGGC 298

||||||||||||||||||||||||||||||||||||||||||||||||||||||||||||

Sbjct 176541 GGCCATGAGAGTGGGAGCCCGGAGATGGACACTGAGACACGGGTCCAGGCCCTACGGGGC 176482

Query 299 GCAGCAGGCGCGAAACCTCCGCAATGCGGGCAACCGCGACGGGGGGACCCCCAGTGCCGT 358

|||||||||||||||||||||||||||||| |||||||||||||||||||||||||||||

Sbjct 176481 GCAGCAGGCGCGAAACCTCCGCAATGCGGGAAACCGCGACGGGGGGACCCCCAGTGCCGT 176422

Query 359 GGCAACGCCACGGCTTTTCCGGAGTGTAAAAAGCTCCGGGAATAAGGGCTGGGCAAGGCC 418

| | | || |||||||||||||||||||||||||||||||||||||||||||||||||

Sbjct 176421 GCCTCAGGCATGGCTTTTCCGGAGTGTAAAAAGCTCCGGGAATAAGGGCTGGGCAAGGCC 176362

Query 419 GGTGGCAGCCGCCGCGGTAATACCGGCGGCCCGAGTGGTGGCCGCTATTATTGGGCCTAA 478

||||||| |||||||||||||||||||||||| |||||||||||||||||||||||||||

Sbjct 176361 GGTGGCATCCGCCGCGGTAATACCGGCGGCCCAAGTGGTGGCCGCTATTATTGGGCCTAA 176302

Query 479 AGCGTCCGTAGCCGGGCCCGTAAGTCCCTGGCGAAATCCCACGGCTCAACCGTGGGGCTT 538

||||||||||||||||||||||||||||||||||||||||||||||||||||||||||||

Sbjct 176301 AGCGTCCGTAGCCGGGCCCGTAAGTCCCTGGCGAAATCCCACGGCTCAACCGTGGGGCTT 176242

Query 539 GCTGGGGATACTGCGGGCCTTGGGACCGGGAGAGGCCGGGGGTACCCCTGGGGTAGGGGT 598

||||||||||||||||||||||||||||||||||||||||||||||||||||||||||||

Sbjct 176241 GCTGGGGATACTGCGGGCCTTGGGACCGGGAGAGGCCGGGGGTACCCCTGGGGTAGGGGT 176182

Query 599 GAAATCCTATAATCCCAGGGGGACCGCCAGTGGCGAAGGCGCCCGGCTGGAACGGGTCCG 658

||||||||||||||||||||||||||||||||||||||||||||||||||||||||||||

Sbjct 176181 GAAATCCTATAATCCCAGGGGGACCGCCAGTGGCGAAGGCGCCCGGCTGGAACGGGTCCG 176122

Query 659 ACGGTGAGGGACGAAGGCCAGGGGAGCGAACCGGATTAGATACCCGGGTAGTCCTGGCTG 718

||||||||||||||||||||||||||||||||||||||||||||||||||||||||||||

Sbjct 176121 ACGGTGAGGGACGAAGGCCAGGGGAGCGAACCGGATTAGATACCCGGGTAGTCCTGGCTG 176062

Query 719 TAAAGGATGCGGGCTAGGTGTCGGGCGAGCTTCGAGCTCGCCCGGTGCCGAAGGGAAGCC 778

|||||||||||||||||||||||||||||||||||||||||||||||||| |||||||||

Sbjct 176061 TAAAGGATGCGGGCTAGGTGTCGGGCGAGCTTCGAGCTCGCCCGGTGCCGGAGGGAAGCC 176002

Query 779 GTTAAGCCCGCCGCCTGGGGAGTACGGCCGCAAGGCTGAAACTTAAAGGAATTGGCGGGG 838

||||||||||||||||||||||||||||||||||||||||||||||||||||||||||||

Sbjct 176001 GTTAAGCCCGCCGCCTGGGGAGTACGGCCGCAAGGCTGAAACTTAAAGGAATTGGCGGGG 175942

Query 839 GAGCACTACAAGGGGTGGAGCGTGCGGTTTAATTGGATTCAACGCCGGGAACCTCACCGG 898

||||||||||||||||||||||||||||||||||||||||||||||||||||||||||||

Sbjct 175941 GAGCACTACAAGGGGTGGAGCGTGCGGTTTAATTGGATTCAACGCCGGGAACCTCACCGG 175882

Query 899 GGGCGACGGCAGGATGAAGGCCAGGCTGAAGGTCTTGCCGGACACGCCGAGAGGAGGTGC 958

||||||||||||||||||||||||||||||||||||||||||| ||||||||||||||||

Sbjct 175881 GGGCGACGGCAGGATGAAGGCCAGGCTGAAGGTCTTGCCGGACGCGCCGAGAGGAGGTGC 175822

Query 959 ATGGCCGCCGTCAGCTCGTACCGTGAGGCGTCCACTTAAGTGTGGTAACGAGCGAGACCC 1018

||||||||||||||||||||||||||||||||||||||||||||||||||||||||||||

Sbjct 175821 ATGGCCGCCGTCAGCTCGTACCGTGAGGCGTCCACTTAAGTGTGGTAACGAGCGAGACCC 175762

Query 1019 GCGCCCCCCAGTTGCCAGTCCTTCCCGCTGGGGAGGAGGCACTCTGGGGGGACCGCCGGC 1078

|||||||| ||||||||||||| |||||||||||||||||||||||||||||||||||||

Sbjct 175761 GCGCCCCC-AGTTGCCAGTCCTCCCCGCTGGGGAGGAGGCACTCTGGGGGGACCGCCGGC 175703

Query 1079 GATAAGCCGGAGGAAGGAGCGGGCGACGGTAGGTCAGTATGCCCCGAAACCCCCGGGCTA 1138

||||||||||||||||||||||||||||||||||||||||||||||||||||||||||||

Sbjct 175702 GATAAGCCGGAGGAAGGAGCGGGCGACGGTAGGTCAGTATGCCCCGAAACCCCCGGGCTA 175643

Query 1139 CACGCGCGCTACAATGGGCGGGACAATGGGATCCGACCCCGAAAGGGGAAGGGAATCCCC 1198

||||||||||||||||||||||||||||||||||||||||||||||||||||||||||||

Sbjct 175642 CACGCGCGCTACAATGGGCGGGACAATGGGATCCGACCCCGAAAGGGGAAGGGAATCCCC 175583

Query 1199 TAAACCCGCCCCCAGTTCGGATCGCGGGCTGCAACTCGCCCGCGTGAAGCTGGAATCCCT 1258

||||||||||| ||||||||||||||||||||||||||||||||||||||||||||||||

Sbjct 175582 TAAACCCGCCCTCAGTTCGGATCGCGGGCTGCAACTCGCCCGCGTGAAGCTGGAATCCCT 175523

Query 1259 AGTACCCGCGTGTCATCATCGCGCGGCGAATACGTCCCTGCTCCTTGCACACACCGCCCG 1318

||||||||||||||||||||||||||||||||||||||||||||||||||||||||||||

Sbjct 175522 AGTACCCGCGTGTCATCATCGCGCGGCGAATACGTCCCTGCTCCTTGCACACACCGCCCG 175463

Query 1319 TCA 1321

|||

Sbjct 175462 TCA 175460

> gi|1214743344|ref|NZ_CP015102.1| Thermococcus pacificus strain

P-4, complete genome

Length=1785673

Score = 2318 bits (1255), Expect = 0.0

Identities = 1300/1322 (98%), Gaps = 2/1322 (0%)

Strand=Plus/Minus

Query 1 GTCCGACTAAGCCATGCGAGTCATGGGGCGCCTTGCGCG-CACCGGCGGACGGCTCAGTA 59

||||||||||||||||||||||| |||| |||| | | ||||||||||||||||||||

Sbjct 153633 GTCCGACTAAGCCATGCGAGTCACGGGGTCCCTTCGGGGACACCGGCGGACGGCTCAGTA 153574

Query 60 ACACGTCGGTAACCTACCCTCGGGAGGGGGATAACCCCGGGAAACTGGGGCTAATCCCCC 119

||||||||||||||||||||||||||||||||||||||||||||||||||||||||||||

Sbjct 153573 ACACGTCGGTAACCTACCCTCGGGAGGGGGATAACCCCGGGAAACTGGGGCTAATCCCCC 153514

Query 120 ATAGGCCTGAGGTACTGGAAGGTCCTCAGGCCGAAAGGGGCTCTGCCCGCCCGAGGATGG 179

||||||||||||||||||||||||||||||||||||||| ||||| ||||||||||||||

Sbjct 153513 ATAGGCCTGAGGTACTGGAAGGTCCTCAGGCCGAAAGGGTCTCTGACCGCCCGAGGATGG 153454

Query 180 GCCGGCGGCCGATTAGGTAGTTGGTGGGGTAACGGCCCACCAAGCCGAAGATCGGTACGG 239

||||||||||||||||||||||||||||||||||||||||||||||||||||||||||||

Sbjct 153453 GCCGGCGGCCGATTAGGTAGTTGGTGGGGTAACGGCCCACCAAGCCGAAGATCGGTACGG 153394

Query 240 GCCATGAGAGTGGGAGCCCGGAGATGGACACTGAGACACGGGTCCAGGCCCTACGGGGCG 299

||||||||||||||||||||||||||||||||||||||||||||||||||||||||||||

Sbjct 153393 GCCATGAGAGTGGGAGCCCGGAGATGGACACTGAGACACGGGTCCAGGCCCTACGGGGCG 153334

Query 300 CAGCAGGCGCGAAACCTCCGCAATGCGGGCAACCGCGACGGGGGGACCCCCAGTGCCGTG 359

||||||||||||||||||||||||||||| ||||||||||||||||||||||||||||||

Sbjct 153333 CAGCAGGCGCGAAACCTCCGCAATGCGGGAAACCGCGACGGGGGGACCCCCAGTGCCGTG 153274

Query 360 GCAACGCCACGGCTTTTCCGGAGTGTAAAAAGCTCCGGGAATAAGGGCTGGGCAAGGCCG 419

||| ||||||||||||||||||||||||| ||||||||||||||||||||||||||||||

Sbjct 153273 GCACCGCCACGGCTTTTCCGGAGTGTAAAGAGCTCCGGGAATAAGGGCTGGGCAAGGCCG 153214

Query 420 GTGGCAGCCGCCGCGGTAATACCGGCGGCCCGAGTGGTGGCCGCTATTATTGGGCCTAAA 479

||||||||||||||||||||||||||||||||||||||||||||||||||||||||||||

Sbjct 153213 GTGGCAGCCGCCGCGGTAATACCGGCGGCCCGAGTGGTGGCCGCTATTATTGGGCCTAAA 153154

Query 480 GCGTCCGTAGCCGGGCCCGTAAGTCCCTGGCGAAATCCCACGGCTCAACCGTGGGGCTTG 539

||||||||||||||||||||||||||||||||||||||||||||||||||||||||||||

Sbjct 153153 GCGTCCGTAGCCGGGCCCGTAAGTCCCTGGCGAAATCCCACGGCTCAACCGTGGGGCTTG 153094

Query 540 CTGGGGATACTGCGGGCCTTGGGACCGGGAGAGGCCGGGGGTACCCCTGGGGTAGGGGTG 599

|||||||||||||||||||||||||||||||||||||||||||| |||||||||||||||

Sbjct 153093 CTGGGGATACTGCGGGCCTTGGGACCGGGAGAGGCCGGGGGTACTCCTGGGGTAGGGGTG 153034

Query 600 AAATCCTATAATCCCAGGGGGACCGCCAGTGGCGAAGGCGCCCGGCTGGAACGGGTCCGA 659

|||||||||||||||||| |||||||||||||||||||||||||||||||||||||||||

Sbjct 153033 AAATCCTATAATCCCAGGAGGACCGCCAGTGGCGAAGGCGCCCGGCTGGAACGGGTCCGA 152974

Query 660 CGGTGAGGGACGAAGGCCAGGGGAGCGAACCGGATTAGATACCCGGGTAGTCCTGGCTGT 719

|||||||||||||||||||||||||| |||||||||||||||||||||||||||||||||

Sbjct 152973 CGGTGAGGGACGAAGGCCAGGGGAGCAAACCGGATTAGATACCCGGGTAGTCCTGGCTGT 152914

Query 720 AAAGGATGCGGGCTAGGTGTCGGGCGAGCTTCGAGCTCGCCCGGTGCCGAAGGGAAGCCG 779

|||||||||||||||||||||||| |||||||| ||||||||||||||||||||||||||

Sbjct 152913 AAAGGATGCGGGCTAGGTGTCGGGTGAGCTTCGGGCTCGCCCGGTGCCGAAGGGAAGCCG 152854

Query 780 TTAAGCCCGCCGCCTGGGGAGTACGGCCGCAAGGCTGAAACTTAAAGGAATTGGCGGGGG 839

||||||||||||||||||||||||||||||||||||||||||||||||||||||||||||

Sbjct 152853 TTAAGCCCGCCGCCTGGGGAGTACGGCCGCAAGGCTGAAACTTAAAGGAATTGGCGGGGG 152794

Query 840 AGCACTACAAGGGGTGGAGCGTGCGGTTTAATTGGATTCAACGCCGGGAACCTCACCGGG 899

||||||||||||||||||||||||||||||||||||||||||||||||||||||||||||

Sbjct 152793 AGCACTACAAGGGGTGGAGCGTGCGGTTTAATTGGATTCAACGCCGGGAACCTCACCGGG 152734

Query 900 GGCGACGGCAGGATGAAGGCCAGGCTGAAGGTCTTGCCGGACACGCCGAGAGGAGGTGCA 959

||||||||||||||||||||||||||||||||||||||||||||||||||||||||||||

Sbjct 152733 GGCGACGGCAGGATGAAGGCCAGGCTGAAGGTCTTGCCGGACACGCCGAGAGGAGGTGCA 152674

Query 960 TGGCCGCCGTCAGCTCGTACCGTGAGGCGTCCACTTAAGTGTGGTAACGAGCGAGACCCG 1019

||||||||||||||||||||||||||||||||||||||||||||||||||||||||||||

Sbjct 152673 TGGCCGCCGTCAGCTCGTACCGTGAGGCGTCCACTTAAGTGTGGTAACGAGCGAGACCCG 152614

Query 1020 CGCCCCCCAGTTGCCAGTCCTTCCCGCTGGGGAGGAGGCACTCTGGGGGGACCGCCGGCG 1079

||||||| ||||||||||||| |||||||||||||||||||||||||||||| |||||||

Sbjct 152613 CGCCCCC-AGTTGCCAGTCCTCCCCGCTGGGGAGGAGGCACTCTGGGGGGACTGCCGGCG 152555

Query 1080 ATAAGCCGGAGGAAGGAGCGGGCGACGGTAGGTCAGTATGCCCCGAAACCCCCGGGCTAC 1139

||||||||||||||||||||||||||||||||||||||||||||||||||||||||||||

Sbjct 152554 ATAAGCCGGAGGAAGGAGCGGGCGACGGTAGGTCAGTATGCCCCGAAACCCCCGGGCTAC 152495

Query 1140 ACGCGCGCTACAATGGGCGGGACAATGGGATCCGACCCCGAAAGGGGAAGGGAATCCCCT 1199

||||||||||||||||||||||||||||||||||||||||||||||||||||||||||||

Sbjct 152494 ACGCGCGCTACAATGGGCGGGACAATGGGATCCGACCCCGAAAGGGGAAGGGAATCCCCT 152435

Query 1200 AAACCCGCCCCCAGTTCGGATCGCGGGCTGCAACTCGCCCGCGTGAAGCTGGAATCCCTA 1259

||||||||||||||||||||| |||||||||||||||||||| |||||||||||||||||

Sbjct 152434 AAACCCGCCCCCAGTTCGGATTGCGGGCTGCAACTCGCCCGCATGAAGCTGGAATCCCTA 152375

Query 1260 GTACCCGCGTGTCATCATCGCGCGGCGAATACGTCCCTGCTCCTTGCACACACCGCCCGT 1319

||||||||||||||||||||||||||||||||||||||||||||||||||||||||||||

Sbjct 152374 GTACCCGCGTGTCATCATCGCGCGGCGAATACGTCCCTGCTCCTTGCACACACCGCCCGT 152315

Query 1320 CA 1321

||

Sbjct 152314 CA 152313

> gi|530547444|ref|NC_022084.1| Thermococcus litoralis DSM 5473,

complete sequence

Length=2215172

Score = 2289 bits (1239), Expect = 0.0

Identities = 1299/1327 (98%), Gaps = 7/1327 (1%)

Strand=Plus/Minus

Query 1 GTCCGACTAAGCCATGCGAGTCA-TGGGGCG--CCT-TGCG-C-GCACCGGCGGACGGCT 54

||||||||||||||||||||||| |||| | ||| || | | |||||||||||||||

Sbjct 775715 GTCCGACTAAGCCATGCGAGTCAAGGGGGTGTCCCTCTGGGACACCACCGGCGGACGGCT 775656

Query 55 CAGTAACACGTCGGTAACCTACCCTCGGGAGGGGGATAACCCCGGGAAACTGGGGCTAAT 114

||||||||||||||||||||||||||||||||||||||||||||||||||||||||||||

Sbjct 775655 CAGTAACACGTCGGTAACCTACCCTCGGGAGGGGGATAACCCCGGGAAACTGGGGCTAAT 775596

Query 115 CCCCCATAGGCCTGAGGTACTGGAAGGTCCTCAGGCCGAAAGGGGCTCTGCCCGCCCGAG 174

||||||||||||||||||||||||||||||||||||||||||||||||||||||||||||

Sbjct 775595 CCCCCATAGGCCTGAGGTACTGGAAGGTCCTCAGGCCGAAAGGGGCTCTGCCCGCCCGAG 775536

Query 175 GATGGGCCGGCGGCCGATTAGGTAGTTGGTGGGGTAACGGCCCACCAAGCCGAAGATCGG 234

||||||||||||||||||||||||||||||||||||||||||||||||||||||||||||

Sbjct 775535 GATGGGCCGGCGGCCGATTAGGTAGTTGGTGGGGTAACGGCCCACCAAGCCGAAGATCGG 775476

Query 235 TACGGGCCATGAGAGTGGGAGCCCGGAGATGGACACTGAGACACGGGTCCAGGCCCTACG 294

||||||| |||||| |||||||||||||||||||||||||||||||||||||||||||

Sbjct 775475 TACGGGCTGTGAGAGCAGGAGCCCGGAGATGGACACTGAGACACGGGTCCAGGCCCTACG 775416

Query 295 GGGCGCAGCAGGCGCGAAACCTCCGCAATGCGGGCAACCGCGACGGGGGGACCCCCAGTG 354

|||||||||||||||||||||||||||||||||| |||||||||||||||||||| ||||

Sbjct 775415 GGGCGCAGCAGGCGCGAAACCTCCGCAATGCGGGAAACCGCGACGGGGGGACCCCGAGTG 775356

Query 355 CCGTGGCAACGCCACGGCTTTTCCGGAGTGTAAAAAGCTCCGGGAATAAGGGCTGGGCAA 414

|||||||| |||||||||||||||||||||||||||||||||||||||||||||||||||

Sbjct 775355 CCGTGGCATCGCCACGGCTTTTCCGGAGTGTAAAAAGCTCCGGGAATAAGGGCTGGGCAA 775296

Query 415 GGCCGGTGGCAGCCGCCGCGGTAATACCGGCGGCCCGAGTGGTGGCCGCTATTATTGGGC 474

||||||||||||||||||||||||||||||||||||||||||||||||||||||||||||

Sbjct 775295 GGCCGGTGGCAGCCGCCGCGGTAATACCGGCGGCCCGAGTGGTGGCCGCTATTATTGGGC 775236

Query 475 CTAAAGCGTCCGTAGCCGGGCCCGTAAGTCCCTGGCGAAATCCCACGGCTCAACCGTGGG 534

||||||||||||||||||||||||||||||||||||||||||||||||||||||||||||

Sbjct 775235 CTAAAGCGTCCGTAGCCGGGCCCGTAAGTCCCTGGCGAAATCCCACGGCTCAACCGTGGG 775176

Query 535 GCTTGCTGGGGATACTGCGGGCCTTGGGACCGGGAGAGGCCGGGGGTACCCCTGGGGTAG 594

|||||||||||||||||||||||||||||||||||||||| |||||||||||||||||||

Sbjct 775175 GCTTGCTGGGGATACTGCGGGCCTTGGGACCGGGAGAGGCGGGGGGTACCCCTGGGGTAG 775116

Query 595 GGGTGAAATCCTATAATCCCAGGGGGACCGCCAGTGGCGAAGGCGCCCGGCTGGAACGGG 654

|||||||||||||||||||||||||||||||||||||||||||||||| |||||||||||

Sbjct 775115 GGGTGAAATCCTATAATCCCAGGGGGACCGCCAGTGGCGAAGGCGCCCCGCTGGAACGGG 775056

Query 655 TCCGACGGTGAGGGACGAAGGCCAGGGGAGCGAACCGGATTAGATACCCGGGTAGTCCTG 714

||||||||||||||||||||||||||||||| ||||||||||||||||||||||||||||

Sbjct 775055 TCCGACGGTGAGGGACGAAGGCCAGGGGAGCAAACCGGATTAGATACCCGGGTAGTCCTG 774996

Query 715 GCTGTAAAGGATGCGGGCTAGGTGTCGGGCGAGCTTCGAGCTCGCCCGGTGCCGAAGGGA 774

||||||||||||||||||||||||||||| |||||||| ||||||||||||||| |||||

Sbjct 774995 GCTGTAAAGGATGCGGGCTAGGTGTCGGGTGAGCTTCGGGCTCGCCCGGTGCCGTAGGGA 774936

Query 775 AGCCGTTAAGCCCGCCGCCTGGGGAGTACGGCCGCAAGGCTGAAACTTAAAGGAATTGGC 834

||||||||||||||||||||||||||||||||||||||||||||||||||||||||||||

Sbjct 774935 AGCCGTTAAGCCCGCCGCCTGGGGAGTACGGCCGCAAGGCTGAAACTTAAAGGAATTGGC 774876

Query 835 GGGGGAGCACTACAAGGGGTGGAGCGTGCGGTTTAATTGGATTCAACGCCGGGAACCTCA 894

||||||||||||||||||||||||||||||||||||||||||||||||||||||||||||

Sbjct 774875 GGGGGAGCACTACAAGGGGTGGAGCGTGCGGTTTAATTGGATTCAACGCCGGGAACCTCA 774816

Query 895 CCGGGGGCGACGGCAGGATGAAGGCCAGGCTGAAGGTCTTGCCGGACACGCCGAGAGGAG 954

||||||||||||||||||||||||||||||||||||||||||||||||||||||||||||

Sbjct 774815 CCGGGGGCGACGGCAGGATGAAGGCCAGGCTGAAGGTCTTGCCGGACACGCCGAGAGGAG 774756

Query 955 GTGCATGGCCGCCGTCAGCTCGTACCGTGAGGCGTCCACTTAAGTGTGGTAACGAGCGAG 1014

||||||||||||||||||||||||||||||||||||||||||||||||||||||||||||

Sbjct 774755 GTGCATGGCCGCCGTCAGCTCGTACCGTGAGGCGTCCACTTAAGTGTGGTAACGAGCGAG 774696

Query 1015 ACCCGCGCCCCCCAGTTGCCAGTCCTTCCCGCTGGGGAGGAGGCACTCTGGGGGGACCGC 1074

|||||||||||| ||||||||| ||||||||||||| ||| |||||||||||||||| ||

Sbjct 774695 ACCCGCGCCCCC-AGTTGCCAGCCCTTCCCGCTGGGAAGGGGGCACTCTGGGGGGACTGC 774637

Query 1075 CGGCGATAAGCCGGAGGAAGGAGCGGGCGACGGTAGGTCAGTATGCCCCGAAACCCCCGG 1134

||||||||||||||||||||||||||||||||||||||||||||||||||||||||||||

Sbjct 774636 CGGCGATAAGCCGGAGGAAGGAGCGGGCGACGGTAGGTCAGTATGCCCCGAAACCCCCGG 774577

Query 1135 GCTACACGCGCGCTACAATGGGCGGGACAATGGGATCCGACCCCGAAAGGGGAAGGGAAT 1194

||||||||||||||||||||||||||||||||||||||||||||||||||||||||||||

Sbjct 774576 GCTACACGCGCGCTACAATGGGCGGGACAATGGGATCCGACCCCGAAAGGGGAAGGGAAT 774517

Query 1195 CCCCTAAACCCGCCCCCAGTTCGGATCGCGGGCTGCAACTCGCCCGCGTGAAGCTGGAAT 1254

||||||||||||||||||||||||||||||||||||||||||||||||||||||||||||

Sbjct 774516 CCCCTAAACCCGCCCCCAGTTCGGATCGCGGGCTGCAACTCGCCCGCGTGAAGCTGGAAT 774457

Query 1255 CCCTAGTACCCGCGTGTCATCATCGCGCGGCGAATACGTCCCTGCTCCTTGCACACACCG 1314

||||||||||||||||||||||||||||||||||||||||||||||||||||||||||||

Sbjct 774456 CCCTAGTACCCGCGTGTCATCATCGCGCGGCGAATACGTCCCTGCTCCTTGCACACACCG 774397

Query 1315 CCCGTCA 1321

|||||||

Sbjct 774396 CCCGTCA 774390

> gi|757128586|ref|NZ_CP006965.1| Thermococcus paralvinellae strain

ES1 chromosome, complete genome

Length=1957742

Score = 2287 bits (1238), Expect = 0.0

Identities = 1301/1330 (98%), Gaps = 10/1330 (1%)

Strand=Plus/Minus

Query 1 GTCCGACTAAGCCATGCGAGTCATGGGG-CG--CC-T-TGCG-C-GCACCGGCGGACGGC 53

|||||||||||||||||||||||||||| | || | || | | ||||||||||||||

Sbjct 966643 GTCCGACTAAGCCATGCGAGTCATGGGGGTGTCCCTTCTGGGACACCACCGGCGGACGGC 966584

Query 54 TCAGTAACACGTCGGTAACCTACCCTCGGGAGGGGGATAACCCCGGGAAACTGGGGCTAA 113

||||||||||||||||||||||||||||||||||||||||||||||||||||||||||||

Sbjct 966583 TCAGTAACACGTCGGTAACCTACCCTCGGGAGGGGGATAACCCCGGGAAACTGGGGCTAA 966524

Query 114 TCCCCCATAGGCCTGAGGTACTGGAAGGTCCTCAGGCCGAAAGGGGCT-C-TGCCCGCCC 171

||||||||||| || ||||||||||||||| || |||||||||||| | |||||||||

Sbjct 966523 TCCCCCATAGGTGTGGGGTACTGGAAGGTCCCCACACCGAAAGGGGCTTCGTGCCCGCCC 966464

Query 172 GAGGATGGGCCGGCGGCCGATTAGGTAGTTGGTGGGGTAACGGCCCACCAAGCCGAAGAT 231

||||||||||||||||||||||||||||||||||||||||||||||||||||||||||||

Sbjct 966463 GAGGATGGGCCGGCGGCCGATTAGGTAGTTGGTGGGGTAACGGCCCACCAAGCCGAAGAT 966404

Query 232 CGGTACGGGCCATGAGAGTGGGAGCCCGGAGATGGACACTGAGACACGGGTCCAGGCCCT 291

||||||||||||||||||||||||||||||||||||||||||||||||||||||||||||

Sbjct 966403 CGGTACGGGCCATGAGAGTGGGAGCCCGGAGATGGACACTGAGACACGGGTCCAGGCCCT 966344

Query 292 ACGGGGCGCAGCAGGCGCGAAACCTCCGCAATGCGGGCAACCGCGACGGGGGGACCCCCA 351

||||||||||||||||||||||||||||||||||||| ||||||||||||||||||||||

Sbjct 966343 ACGGGGCGCAGCAGGCGCGAAACCTCCGCAATGCGGGAAACCGCGACGGGGGGACCCCCA 966284

Query 352 GTGCCGTGGCAACGCCACGGCTTTTCCGGAGTGTAAAAAGCTCCGGGAATAAGGGCTGGG 411

||||||||||||||||||||||||||||||||||||||||||||||||||||||||||||

Sbjct 966283 GTGCCGTGGCAACGCCACGGCTTTTCCGGAGTGTAAAAAGCTCCGGGAATAAGGGCTGGG 966224

Query 412 CAAGGCCGGTGGCAGCCGCCGCGGTAATACCGGCGGCCCGAGTGGTGGCCGCTATTATTG 471

||||||||||||||||||||||||||||||||||||||||||||||||||||||||||||

Sbjct 966223 CAAGGCCGGTGGCAGCCGCCGCGGTAATACCGGCGGCCCGAGTGGTGGCCGCTATTATTG 966164

Query 472 GGCCTAAAGCGTCCGTAGCCGGGCCCGTAAGTCCCTGGCGAAATCCCACGGCTCAACCGT 531

||||||||||||||||||||||||||||||||||||||||||||||||||||||||||||

Sbjct 966163 GGCCTAAAGCGTCCGTAGCCGGGCCCGTAAGTCCCTGGCGAAATCCCACGGCTCAACCGT 966104

Query 532 GGGGCTTGCTGGGGATACTGCGGGCCTTGGGACCGGGAGAGGCCGGGGGTACCCCTGGGG 591

||||||||||||||||||||||||||||||||||||||||||| ||||||||||||||||

Sbjct 966103 GGGGCTTGCTGGGGATACTGCGGGCCTTGGGACCGGGAGAGGCGGGGGGTACCCCTGGGG 966044

Query 592 TAGGGGTGAAATCCTATAATCCCAGGGGGACCGCCAGTGGCGAAGGCGCCCGGCTGGAAC 651

||||||||||||||||||||||||||||||||||||||||||||||||||| ||||||||

Sbjct 966043 TAGGGGTGAAATCCTATAATCCCAGGGGGACCGCCAGTGGCGAAGGCGCCCCGCTGGAAC 965984

Query 652 GGGTCCGACGGTGAGGGACGAAGGCCAGGGGAGCGAACCGGATTAGATACCCGGGTAGTC 711

||||||||||||||||||||||||||||||||||||||||||||||||||||||||||||

Sbjct 965983 GGGTCCGACGGTGAGGGACGAAGGCCAGGGGAGCGAACCGGATTAGATACCCGGGTAGTC 965924

Query 712 CTGGCTGTAAAGGATGCGGGCTAGGTGTCGGGCGAGCTTCGAGCTCGCCCGGTGCCGAAG 771

|||||||||||||||||||||||||||||||||||||| |||||||||||||||||| ||

Sbjct 965923 CTGGCTGTAAAGGATGCGGGCTAGGTGTCGGGCGAGCTCCGAGCTCGCCCGGTGCCGTAG 965864

Query 772 GGAAGCCGTTAAGCCCGCCGCCTGGGGAGTACGGCCGCAAGGCTGAAACTTAAAGGAATT 831

||||||||||||||||||||||||||||||||||||||||||||||||||||||||||||

Sbjct 965863 GGAAGCCGTTAAGCCCGCCGCCTGGGGAGTACGGCCGCAAGGCTGAAACTTAAAGGAATT 965804

Query 832 GGCGGGGGAGCACTACAAGGGGTGGAGCGTGCGGTTTAATTGGATTCAACGCCGGGAACC 891

||||||||||||||||||||||||||||||||||||||||||||||||||||||||||||

Sbjct 965803 GGCGGGGGAGCACTACAAGGGGTGGAGCGTGCGGTTTAATTGGATTCAACGCCGGGAACC 965744

Query 892 TCACCGGGGGCGACGGCAGGATGAAGGCCAGGCTGAAGGTCTTGCCGGACACGCCGAGAG 951

||||||||||||||||||||||||||||||||||||||||||||||||||||||||||||

Sbjct 965743 TCACCGGGGGCGACGGCAGGATGAAGGCCAGGCTGAAGGTCTTGCCGGACACGCCGAGAG 965684

Query 952 GAGGTGCATGGCCGCCGTCAGCTCGTACCGTGAGGCGTCCACTTAAGTGTGGTAACGAGC 1011

||||||||||||||||||||||||||||||||||||||||||||||||||||||||||||

Sbjct 965683 GAGGTGCATGGCCGCCGTCAGCTCGTACCGTGAGGCGTCCACTTAAGTGTGGTAACGAGC 965624

Query 1012 GAGACCCGCGCCCCCCAGTTGCCAGTCCTTCCCGCTGGGGAGGAGGCACTCTGGGGGGAC 1071

||||||||||||||| |||||||||||| |||||||||| |||||||||||||||||||

Sbjct 965623 GAGACCCGCGCCCCC-AGTTGCCAGTCCCTCCCGCTGGGAGGGAGGCACTCTGGGGGGAC 965565

Query 1072 CGCCGGCGATAAGCCGGAGGAAGGAGCGGGCGACGGTAGGTCAGTATGCCCCGAAACCCC 1131

||||||||||||||||||||||||||||||||||||||||||||||||||||||||||||

Sbjct 965564 CGCCGGCGATAAGCCGGAGGAAGGAGCGGGCGACGGTAGGTCAGTATGCCCCGAAACCCC 965505

Query 1132 CGGGCTACACGCGCGCTACAATGGGCGGGACAATGGGATCCGACCCCGAAAGGGGAAGGG 1191

|||||||||||||||||||||||||||||||||||||||||||||||||||||||||||

Sbjct 965504 CGGGCTACACGCGCGCTACAATGGGCGGGACAATGGGATCCGACCCCGAAAGGGGAAGGA 965445

Query 1192 AATCCCCTAAACCCGCCCCCAGTTCGGATCGCGGGCTGCAACTCGCCCGCGTGAAGCTGG 1251

|||||||||||||||||| |||||||||||||||||||||||||||||||||||||||||

Sbjct 965444 AATCCCCTAAACCCGCCCTCAGTTCGGATCGCGGGCTGCAACTCGCCCGCGTGAAGCTGG 965385

Query 1252 AATCCCTAGTACCCGCGTGTCATCATCGCGCGGCGAATACGTCCCTGCTCCTTGCACACA 1311

||||||||||||||||||||||||||||||||||||||||||||||||||||||||||||

Sbjct 965384 AATCCCTAGTACCCGCGTGTCATCATCGCGCGGCGAATACGTCCCTGCTCCTTGCACACA 965325

Query 1312 CCGCCCGTCA 1321

||||||||||

Sbjct 965324 CCGCCCGTCA 965315

> gi|315229765|ref|NC_014804.1| Thermococcus barophilus MP, complete

sequence

Length=2010078

Score = 2287 bits (1238), Expect = 0.0

Identities = 1301/1330 (98%), Gaps = 10/1330 (1%)

Strand=Plus/Plus

Query 1 GTCCGACTAAGCCATGCGAGTCATGGGG-CG--CC-T-TGCG-C-GCACCGGCGGACGGC 53

|||||||||||||||||||||||||||| | || | || | | ||||||||||||||

Sbjct 812462 GTCCGACTAAGCCATGCGAGTCATGGGGGTGTCCCTTCTGGGACACCACCGGCGGACGGC 812521

Query 54 TCAGTAACACGTCGGTAACCTACCCTCGGGAGGGGGATAACCCCGGGAAACTGGGGCTAA 113

||||||||||||||||||||||||||||||||||||||||||||||||||||||||||||

Sbjct 812522 TCAGTAACACGTCGGTAACCTACCCTCGGGAGGGGGATAACCCCGGGAAACTGGGGCTAA 812581

Query 114 TCCCCCATAGGCCTGAGGTACTGGAAGGTCCTCAGGCCGAAAGGGGC-TC-TGCCCGCCC 171

||||||||||| || ||||||||||||||| || ||||||||||| || |||||||||

Sbjct 812582 TCCCCCATAGGTGTGGGGTACTGGAAGGTCCCCACACCGAAAGGGGCCTCGTGCCCGCCC 812641

Query 172 GAGGATGGGCCGGCGGCCGATTAGGTAGTTGGTGGGGTAACGGCCCACCAAGCCGAAGAT 231

||||||||||||||||||||||||||||||||||||||||||||||||||||||||||||

Sbjct 812642 GAGGATGGGCCGGCGGCCGATTAGGTAGTTGGTGGGGTAACGGCCCACCAAGCCGAAGAT 812701

Query 232 CGGTACGGGCCATGAGAGTGGGAGCCCGGAGATGGACACTGAGACACGGGTCCAGGCCCT 291

||||||||||||||||||||||||||||||||||||||||||||||||||||||||||||

Sbjct 812702 CGGTACGGGCCATGAGAGTGGGAGCCCGGAGATGGACACTGAGACACGGGTCCAGGCCCT 812761

Query 292 ACGGGGCGCAGCAGGCGCGAAACCTCCGCAATGCGGGCAACCGCGACGGGGGGACCCCCA 351

||||||||||||||||||||||||||||||||||||| ||||||||||||||||||||||

Sbjct 812762 ACGGGGCGCAGCAGGCGCGAAACCTCCGCAATGCGGGAAACCGCGACGGGGGGACCCCCA 812821

Query 352 GTGCCGTGGCAACGCCACGGCTTTTCCGGAGTGTAAAAAGCTCCGGGAATAAGGGCTGGG 411

||||||||||||||||||||||||||||||||||||||||||||||||||||||||||||

Sbjct 812822 GTGCCGTGGCAACGCCACGGCTTTTCCGGAGTGTAAAAAGCTCCGGGAATAAGGGCTGGG 812881

Query 412 CAAGGCCGGTGGCAGCCGCCGCGGTAATACCGGCGGCCCGAGTGGTGGCCGCTATTATTG 471

||||||||||||||||||||||||||||||||||||||||||||||||||||||||||||

Sbjct 812882 CAAGGCCGGTGGCAGCCGCCGCGGTAATACCGGCGGCCCGAGTGGTGGCCGCTATTATTG 812941

Query 472 GGCCTAAAGCGTCCGTAGCCGGGCCCGTAAGTCCCTGGCGAAATCCCACGGCTCAACCGT 531

||||||||||||||||||||||||||||||||||||||||||||||||||||||||||||

Sbjct 812942 GGCCTAAAGCGTCCGTAGCCGGGCCCGTAAGTCCCTGGCGAAATCCCACGGCTCAACCGT 813001

Query 532 GGGGCTTGCTGGGGATACTGCGGGCCTTGGGACCGGGAGAGGCCGGGGGTACCCCTGGGG 591

||||||||||||||||||||||||||||||||||||||||||| ||||||||||||||||

Sbjct 813002 GGGGCTTGCTGGGGATACTGCGGGCCTTGGGACCGGGAGAGGCGGGGGGTACCCCTGGGG 813061

Query 592 TAGGGGTGAAATCCTATAATCCCAGGGGGACCGCCAGTGGCGAAGGCGCCCGGCTGGAAC 651

||||||||||||||||||||||||||||||||||||||||||||||||||| ||||||||

Sbjct 813062 TAGGGGTGAAATCCTATAATCCCAGGGGGACCGCCAGTGGCGAAGGCGCCCCGCTGGAAC 813121

Query 652 GGGTCCGACGGTGAGGGACGAAGGCCAGGGGAGCGAACCGGATTAGATACCCGGGTAGTC 711

||||||||||||||||||||||||||||||||||||||||||||||||||||||||||||

Sbjct 813122 GGGTCCGACGGTGAGGGACGAAGGCCAGGGGAGCGAACCGGATTAGATACCCGGGTAGTC 813181

Query 712 CTGGCTGTAAAGGATGCGGGCTAGGTGTCGGGCGAGCTTCGAGCTCGCCCGGTGCCGAAG 771

|||||||||||||||||||||||||||||||||||||| |||||||||||||||||| ||

Sbjct 813182 CTGGCTGTAAAGGATGCGGGCTAGGTGTCGGGCGAGCTCCGAGCTCGCCCGGTGCCGTAG 813241

Query 772 GGAAGCCGTTAAGCCCGCCGCCTGGGGAGTACGGCCGCAAGGCTGAAACTTAAAGGAATT 831

||||||||||||||||||||||||||||||||||||||||||||||||||||||||||||

Sbjct 813242 GGAAGCCGTTAAGCCCGCCGCCTGGGGAGTACGGCCGCAAGGCTGAAACTTAAAGGAATT 813301

Query 832 GGCGGGGGAGCACTACAAGGGGTGGAGCGTGCGGTTTAATTGGATTCAACGCCGGGAACC 891

||||||||||||||||||||||||||||||||||||||||||||||||||||||||||||

Sbjct 813302 GGCGGGGGAGCACTACAAGGGGTGGAGCGTGCGGTTTAATTGGATTCAACGCCGGGAACC 813361

Query 892 TCACCGGGGGCGACGGCAGGATGAAGGCCAGGCTGAAGGTCTTGCCGGACACGCCGAGAG 951

||||||||||||||||||||||||||||||||||||||||||||||||||||||||||||

Sbjct 813362 TCACCGGGGGCGACGGCAGGATGAAGGCCAGGCTGAAGGTCTTGCCGGACACGCCGAGAG 813421

Query 952 GAGGTGCATGGCCGCCGTCAGCTCGTACCGTGAGGCGTCCACTTAAGTGTGGTAACGAGC 1011

||||||||||||||||||||||||||||||||||||||||||||||||||||||||||||

Sbjct 813422 GAGGTGCATGGCCGCCGTCAGCTCGTACCGTGAGGCGTCCACTTAAGTGTGGTAACGAGC 813481

Query 1012 GAGACCCGCGCCCCCCAGTTGCCAGTCCTTCCCGCTGGGGAGGAGGCACTCTGGGGGGAC 1071

|||||||||| ||||||||||||||||| ||||| |||| |||||||||||||||||||

Sbjct 813482 GAGACCCGCG-CCCCCAGTTGCCAGTCCCTCCCGATGGGAGGGAGGCACTCTGGGGGGAC 813540

Query 1072 CGCCGGCGATAAGCCGGAGGAAGGAGCGGGCGACGGTAGGTCAGTATGCCCCGAAACCCC 1131

||||||||||||||||||||||||||||||||||||||||||||||||||||||||||||

Sbjct 813541 CGCCGGCGATAAGCCGGAGGAAGGAGCGGGCGACGGTAGGTCAGTATGCCCCGAAACCCC 813600

Query 1132 CGGGCTACACGCGCGCTACAATGGGCGGGACAATGGGATCCGACCCCGAAAGGGGAAGGG 1191

||||||||||||||||||||||||||||||||||||||||||||||||||||||||||||

Sbjct 813601 CGGGCTACACGCGCGCTACAATGGGCGGGACAATGGGATCCGACCCCGAAAGGGGAAGGG 813660

Query 1192 AATCCCCTAAACCCGCCCCCAGTTCGGATCGCGGGCTGCAACTCGCCCGCGTGAAGCTGG 1251

|||||||||||||||||| |||||||||||||||||||||||||||||||||||||||||

Sbjct 813661 AATCCCCTAAACCCGCCCTCAGTTCGGATCGCGGGCTGCAACTCGCCCGCGTGAAGCTGG 813720

Query 1252 AATCCCTAGTACCCGCGTGTCATCATCGCGCGGCGAATACGTCCCTGCTCCTTGCACACA 1311

||||||||||||||||||||||||||||||||||||||||||||||||||||||||||||

Sbjct 813721 AATCCCTAGTACCCGCGTGTCATCATCGCGCGGCGAATACGTCCCTGCTCCTTGCACACA 813780

Query 1312 CCGCCCGTCA 1321

||||||||||

Sbjct 813781 CCGCCCGTCA 813790

> gi|1741192688|ref|NZ_CP023154.1| Pyrococcus furiosus DSM 3638

chromosome, complete genome

Length=1889914

Score = 2218 bits (1201), Expect = 0.0

Identities = 1292/1334 (97%), Gaps = 14/1334 (1%)

Strand=Plus/Plus

Query 1 GTCCGACTAAGCCATGCGAGTCA-TGGGGCG--CC-T-TGCG-CG-CACCGGCGGACGGC 53

||||||||||||||||||||||| |||||| || | || | || ||||||||||||||

Sbjct 120724 GTCCGACTAAGCCATGCGAGTCAAGGGGGCGTCCCTTCTGGGACGCCACCGGCGGACGGC 120783

Query 54 TCAGTAACACGTCGGTAACCTACCCTCGGGAGGGGGATAACCCCGGGAAACTGGGGCTAA 113

||||||||||||||||||||||||||||||||||||||||||||||||||||||||||||

Sbjct 120784 TCAGTAACACGTCGGTAACCTACCCTCGGGAGGGGGATAACCCCGGGAAACTGGGGCTAA 120843

Query 114 TCCCCCATAGGCCTGAGGTACTGGAAGGTCCTCAGGCCGAAAGGG-G-C-T-CTGC-CCG 168

||||||||||||||| ||||||||||||||| ||||||||||||| | | | || |||

Sbjct 120844 TCCCCCATAGGCCTGGGGTACTGGAAGGTCCCCAGGCCGAAAGGGAGCCGTAAGGCTCCG 120903

Query 169 CCCGAGGATGGGCCGGCGGCCGATTAGGTAGTTGGTGGGGTAACGGCCCACCAAGCCGAA 228

||||||||||||||||||||||||||||||||||||||||||||||||||||||||||||

Sbjct 120904 CCCGAGGATGGGCCGGCGGCCGATTAGGTAGTTGGTGGGGTAACGGCCCACCAAGCCGAA 120963

Query 229 GATCGGTACGGGCCATGAGAGTGGGAGCCCGGAGATGGACACTGAGACACGGGTCCAGGC 288

|||||||||||||| |||||| ||||||||||||||||||||||||||||||||||||||

Sbjct 120964 GATCGGTACGGGCCGTGAGAGCGGGAGCCCGGAGATGGACACTGAGACACGGGTCCAGGC 121023

Query 289 CCTACGGGGCGCAGCAGGCGCGAAACCTCCGCAATGCGGGCAACCGCGACGGGGGGACCC 348

|||||||||||||||||||||||||||||||||||||||| |||||||||||||||||||

Sbjct 121024 CCTACGGGGCGCAGCAGGCGCGAAACCTCCGCAATGCGGGAAACCGCGACGGGGGGACCC 121083

Query 349 CCAGTGCCGTGGCAACGCCACGGCTTTTCCGGAGTGTAAAAAGCTCCGGGAATAAGGGCT 408

||||||||||| | | ||||||||||||||||||||||||||||||||||||||||||

Sbjct 121084 CCAGTGCCGTGCCTCTGGCACGGCTTTTCCGGAGTGTAAAAAGCTCCGGGAATAAGGGCT 121143

Query 409 GGGCAAGGCCGGTGGCAGCCGCCGCGGTAATACCGGCGGCCCGAGTGGTGGCCGCTATTA 468

||||||||||||||||||||||||||||||||||||||||||||||||||||| ||||||

Sbjct 121144 GGGCAAGGCCGGTGGCAGCCGCCGCGGTAATACCGGCGGCCCGAGTGGTGGCCACTATTA 121203

Query 469 TTGGGCCTAAAGCGTCCGTAGCCGGGCCCGTAAGTCCCTGGCGAAATCCCACGGCTCAAC 528

|||||||||||||| |||||||||||||||||||||||||||||||||||||||||||||

Sbjct 121204 TTGGGCCTAAAGCGGCCGTAGCCGGGCCCGTAAGTCCCTGGCGAAATCCCACGGCTCAAC 121263

Query 529 CGTGGGGCTTGCTGGGGATACTGCGGGCCTTGGGACCGGGAGAGGCCGGGGGTACCCCTG 588

||||||||| |||||||||||||||||||||||||||||||||||||||||||||||| |

Sbjct 121264 CGTGGGGCTCGCTGGGGATACTGCGGGCCTTGGGACCGGGAGAGGCCGGGGGTACCCCCG 121323

Query 589 GGGTAGGGGTGAAATCCTATAATCCCAGGGGGACCGCCAGTGGCGAAGGCGCCCGGCTGG 648

|||||||||||||||||||||||||| |||||||||||||||||||||||||||||||||

Sbjct 121324 GGGTAGGGGTGAAATCCTATAATCCCGGGGGGACCGCCAGTGGCGAAGGCGCCCGGCTGG 121383

Query 649 AACGGGTCCGACGGTGAGGGACGAAGGCCAGGGGAGCGAACCGGATTAGATACCCGGGTA 708

|||||||||||||||||||| |||||||||||||||||||||||||||||||||||||||

Sbjct 121384 AACGGGTCCGACGGTGAGGGCCGAAGGCCAGGGGAGCGAACCGGATTAGATACCCGGGTA 121443

Query 709 GTCCTGGCTGTAAAGGATGCGGGCTAGGTGTCGGGCGAGCTTCGAGCTCGCCCGGTGCCG 768

||||||||||||||||||||||||||||||||||||||||||||||||||||||||||||

Sbjct 121444 GTCCTGGCTGTAAAGGATGCGGGCTAGGTGTCGGGCGAGCTTCGAGCTCGCCCGGTGCCG 121503

Query 769 AAGGGAAGCCGTTAAGCCCGCCGCCTGGGGAGTACGGCCGCAAGGCTGAAACTTAAAGGA 828

|||||||||||||||||||||||||||||||||||||||||||||||||||||||||||

Sbjct 121504 TAGGGAAGCCGTTAAGCCCGCCGCCTGGGGAGTACGGCCGCAAGGCTGAAACTTAAAGGA 121563

Query 829 ATTGGCGGGGGAGCACTACAAGGGGTGGAGCGTGCGGTTTAATTGGATTCAACGCCGGGA 888

||||||||||||||||||||||||||||||||||||||||||||||||||||||||||||

Sbjct 121564 ATTGGCGGGGGAGCACTACAAGGGGTGGAGCGTGCGGTTTAATTGGATTCAACGCCGGGA 121623

Query 889 ACCTCACCGGGGGCGACGGCAGGATGAAGGCCAGGCTGAAGGTCTTGCCGGACACGCCGA 948

||||||||||||||||||||||||||||||||||||||||||||||||||||| ||||||

Sbjct 121624 ACCTCACCGGGGGCGACGGCAGGATGAAGGCCAGGCTGAAGGTCTTGCCGGACGCGCCGA 121683

Query 949 GAGGAGGTGCATGGCCGCCGTCAGCTCGTACCGTGAGGCGTCCACTTAAGTGTGGTAACG 1008

||||||||||||||||||||||||||||||||||||||||||||||||||||||||||||

Sbjct 121684 GAGGAGGTGCATGGCCGCCGTCAGCTCGTACCGTGAGGCGTCCACTTAAGTGTGGTAACG 121743

Query 1009 AGCGAGACCCGCGCCCCCCAGTTGCCAGTCCTTCCCGCTGGGGA-GGAGGCACTCTGGGG 1067

||||||||||||| ||||||||||||||||| ||||||| |||| |||||||||||||||

Sbjct 121744 AGCGAGACCCGCG-CCCCCAGTTGCCAGTCCCTCCCGCTCGGGAGGGAGGCACTCTGGGG 121802

Query 1068 GGACCGCCGGCGATAAGCCGGAGGAAGGAGCGGGCGACGGTAGGTCAGTATGCCCCGAAA 1127

|||| ||||||||||||||||||||||| |||||||||||||||||||||||||||||||

Sbjct 121803 GGACTGCCGGCGATAAGCCGGAGGAAGGGGCGGGCGACGGTAGGTCAGTATGCCCCGAAA 121862

Query 1128 CCCCCGGGCTACACGCGCGCTACAATGGGCGGGACAATGGGATCCGACCCCGAAAGGGGA 1187

|||||||||||||||||||||||||||||||||||||||||| |||||||||||||||||

Sbjct 121863 CCCCCGGGCTACACGCGCGCTACAATGGGCGGGACAATGGGACCCGACCCCGAAAGGGGA 121922

Query 1188 AGGGAATCCCCTAAACCCGCCCCCAGTTCGGATCGCGGGCTGCAACTCGCCCGCGTGAAG 1247

|||||||||||||||||||||| |||||||||||||||||||||||||||||||||||||

Sbjct 121923 AGGGAATCCCCTAAACCCGCCCTCAGTTCGGATCGCGGGCTGCAACTCGCCCGCGTGAAG 121982

Query 1248 CTGGAATCCCTAGTACCCGCGTGTCATCATCGCGCGGCGAATACGTCCCTGCTCCTTGCA 1307

||||||||||||||||||||||||||||||||||||||||||||||||||||||||||||

Sbjct 121983 CTGGAATCCCTAGTACCCGCGTGTCATCATCGCGCGGCGAATACGTCCCTGCTCCTTGCA 122042

Query 1308 CACACCGCCCGTCA 1321

||||||||||||||

Sbjct 122043 CACACCGCCCGTCA 122056

> gi|337283511|ref|NC_015680.1| Pyrococcus yayanosii CH1, complete

sequence

Length=1716818

Score = 2213 bits (1198), Expect = 0.0

Identities = 1291/1334 (97%), Gaps = 14/1334 (1%)

Strand=Plus/Plus

Query 1 GTCCGACTAAGCCATGCGAGTCA-TGGGGCG--CC-T-TGCG-CG-CACCGGCGGACGGC 53

||||||||||||||||||||||| |||||| || | || | || ||||||||||||||

Sbjct 1482232 GTCCGACTAAGCCATGCGAGTCAAGGGGGCGTCCCTTCTGGGACGCCACCGGCGGACGGC 1482291

Query 54 TCAGTAACACGTCGGTAACCTACCCTCGGGAGGGGGATAACCCCGGGAAACTGGGGCTAA 113

||||||||||||||||||||||||||||||||||||||||||||||||||||||||||||

Sbjct 1482292 TCAGTAACACGTCGGTAACCTACCCTCGGGAGGGGGATAACCCCGGGAAACTGGGGCTAA 1482351

Query 114 TCCCCCATAGGCCTGAGGTACTGGAAGGTCCTCAGGCCGAAAGGG-G-C-T-CTGC-CCG 168

||||||||||||||| ||||||||||||||| ||||||||||||| | | | || |||

Sbjct 1482352 TCCCCCATAGGCCTGGGGTACTGGAAGGTCCCCAGGCCGAAAGGGAGCCGTAAGGCTCCG 1482411

Query 169 CCCGAGGATGGGCCGGCGGCCGATTAGGTAGTTGGTGGGGTAACGGCCCACCAAGCCGAA 228

||||||||||||||||||||||||||||||||||||||||||||||||||||||||||||

Sbjct 1482412 CCCGAGGATGGGCCGGCGGCCGATTAGGTAGTTGGTGGGGTAACGGCCCACCAAGCCGAA 1482471

Query 229 GATCGGTACGGGCCATGAGAGTGGGAGCCCGGAGATGGACACTGAGACACGGGTCCAGGC 288

|||||||||||||| |||||| ||||||||||||||||||||||||||||||||||||||

Sbjct 1482472 GATCGGTACGGGCCGTGAGAGCGGGAGCCCGGAGATGGACACTGAGACACGGGTCCAGGC 1482531

Query 289 CCTACGGGGCGCAGCAGGCGCGAAACCTCCGCAATGCGGGCAACCGCGACGGGGGGACCC 348

|||||||||||||||||||||||||||||||||||||||| |||||||||||||||||||

Sbjct 1482532 CCTACGGGGCGCAGCAGGCGCGAAACCTCCGCAATGCGGGAAACCGCGACGGGGGGACCC 1482591

Query 349 CCAGTGCCGTGGCAACGCCACGGCTTTTCCGGAGTGTAAAAAGCTCCGGGAATAAGGGCT 408

||||||||||| | | ||||||||||||||||||||||||||||||||||||||||||

Sbjct 1482592 CCAGTGCCGTGCCTCTGGCACGGCTTTTCCGGAGTGTAAAAAGCTCCGGGAATAAGGGCT 1482651

Query 409 GGGCAAGGCCGGTGGCAGCCGCCGCGGTAATACCGGCGGCCCGAGTGGTGGCCGCTATTA 468

||||||||||||||||||||||||||||||||||||||||||||||||||||| ||||||

Sbjct 1482652 GGGCAAGGCCGGTGGCAGCCGCCGCGGTAATACCGGCGGCCCGAGTGGTGGCCACTATTA 1482711

Query 469 TTGGGCCTAAAGCGTCCGTAGCCGGGCCCGTAAGTCCCTGGCGAAATCCCACGGCTCAAC 528

|||||||||||||| |||||||||||||||||||||||||||||||||||||||||||||

Sbjct 1482712 TTGGGCCTAAAGCGGCCGTAGCCGGGCCCGTAAGTCCCTGGCGAAATCCCACGGCTCAAC 1482771

Query 529 CGTGGGGCTTGCTGGGGATACTGCGGGCCTTGGGACCGGGAGAGGCCGGGGGTACCCCTG 588

||||||||| |||||||||||||||||||||||||||||||||||||||||||||||| |

Sbjct 1482772 CGTGGGGCTCGCTGGGGATACTGCGGGCCTTGGGACCGGGAGAGGCCGGGGGTACCCCCG 1482831

Query 589 GGGTAGGGGTGAAATCCTATAATCCCAGGGGGACCGCCAGTGGCGAAGGCGCCCGGCTGG 648

|||||||||||||||||||||||||| |||||||||||||||||||||||||||||||||

Sbjct 1482832 GGGTAGGGGTGAAATCCTATAATCCCGGGGGGACCGCCAGTGGCGAAGGCGCCCGGCTGG 1482891

Query 649 AACGGGTCCGACGGTGAGGGACGAAGGCCAGGGGAGCGAACCGGATTAGATACCCGGGTA 708

|||||||||||||||||||| |||||||||||||||||||||||||||||||||||||||

Sbjct 1482892 AACGGGTCCGACGGTGAGGGCCGAAGGCCAGGGGAGCGAACCGGATTAGATACCCGGGTA 1482951

Query 709 GTCCTGGCTGTAAAGGATGCGGGCTAGGTGTCGGGCGAGCTTCGAGCTCGCCCGGTGCCG 768

||||||||||||||||||||||||||||||||||||||||||||||||||||||||||||

Sbjct 1482952 GTCCTGGCTGTAAAGGATGCGGGCTAGGTGTCGGGCGAGCTTCGAGCTCGCCCGGTGCCG 1483011

Query 769 AAGGGAAGCCGTTAAGCCCGCCGCCTGGGGAGTACGGCCGCAAGGCTGAAACTTAAAGGA 828

|||||||||||||||||||||||||||||||||||||||||||||||||||||||||||

Sbjct 1483012 TAGGGAAGCCGTTAAGCCCGCCGCCTGGGGAGTACGGCCGCAAGGCTGAAACTTAAAGGA 1483071

Query 829 ATTGGCGGGGGAGCACTACAAGGGGTGGAGCGTGCGGTTTAATTGGATTCAACGCCGGGA 888

||||||||||||||||||||||||||||||||||||||||||||||||||||||||||||

Sbjct 1483072 ATTGGCGGGGGAGCACTACAAGGGGTGGAGCGTGCGGTTTAATTGGATTCAACGCCGGGA 1483131

Query 889 ACCTCACCGGGGGCGACGGCAGGATGAAGGCCAGGCTGAAGGTCTTGCCGGACACGCCGA 948

||||||||||||||||||||||||||||||||||||||||||||||||||||| ||||||

Sbjct 1483132 ACCTCACCGGGGGCGACGGCAGGATGAAGGCCAGGCTGAAGGTCTTGCCGGACGCGCCGA 1483191

Query 949 GAGGAGGTGCATGGCCGCCGTCAGCTCGTACCGTGAGGCGTCCACTTAAGTGTGGTAACG 1008

||||||||||||||||||||||||||||||||||||||||||||||||||||||||||||

Sbjct 1483192 GAGGAGGTGCATGGCCGCCGTCAGCTCGTACCGTGAGGCGTCCACTTAAGTGTGGTAACG 1483251

Query 1009 AGCGAGACCCGCGCCCCCCAGTTGCCAGTCCTTCCCGCTGGGGA-GGAGGCACTCTGGGG 1067

||||||||||||| ||||||||||||||||| ||||||| |||| |||||||||||||||

Sbjct 1483252 AGCGAGACCCGCG-CCCCCAGTTGCCAGTCCCTCCCGCTCGGGAGGGAGGCACTCTGGGG 1483310

Query 1068 GGACCGCCGGCGATAAGCCGGAGGAAGGAGCGGGCGACGGTAGGTCAGTATGCCCCGAAA 1127

|||| ||||||||||||||||||||||| |||||||||||||||||||||||||||||||

Sbjct 1483311 GGACTGCCGGCGATAAGCCGGAGGAAGGGGCGGGCGACGGTAGGTCAGTATGCCCCGAAA 1483370

Query 1128 CCCCCGGGCTACACGCGCGCTACAATGGGCGGGACAATGGGATCCGACCCCGAAAGGGGA 1187

|||||||||||||||||||||||||||||||||||||||||| |||||||||||||||||

Sbjct 1483371 CCCCCGGGCTACACGCGCGCTACAATGGGCGGGACAATGGGACCCGACCCCGAAAGGGGA 1483430

Query 1188 AGGGAATCCCCTAAACCCGCCCCCAGTTCGGATCGCGGGCTGCAACTCGCCCGCGTGAAG 1247

|||||||||||||||||||||| |||||||||||||||||||||||||||||||||||||

Sbjct 1483431 AGGGAATCCCCTAAACCCGCCCTCAGTTCGGATCGCGGGCTGCAACTCGCCCGCGTGAAG 1483490

Query 1248 CTGGAATCCCTAGTACCCGCGTGTCATCATCGCGCGGCGAATACGTCCCTGCTCCTTGCA 1307

||||||||||||||||||||| ||||||||||||||||||||||||||||||||||||||

Sbjct 1483491 CTGGAATCCCTAGTACCCGCGCGTCATCATCGCGCGGCGAATACGTCCCTGCTCCTTGCA 1483550

Query 1308 CACACCGCCCGTCA 1321

||||||||||||||

Sbjct 1483551 CACACCGCCCGTCA 1483564

> gi|1057206559|ref|NZ_LN999010.1| Thermococcus chitonophagus isolate

1 chromosome I, complete sequence

Length=1969648

Score = 2211 bits (1197), Expect = 0.0

Identities = 1290/1333 (97%), Gaps = 13/1333 (1%)

Strand=Plus/Plus

Query 1 GTCCGACTAAGCCATGCGAGTCA-TGGGGCG--CC-T-TGCG-CG-CACCGGCGGACGGC 53

||||||||||||||||||||||| |||||| || | || | || ||||||||||||||

Sbjct 307951 GTCCGACTAAGCCATGCGAGTCAAGGGGGCGTCCCTTCTGGGACGCCACCGGCGGACGGC 308010

Query 54 TCAGTAACACGTCGGTAACCTACCCTCGGGAGGGGGATAACCCCGGGAAACTGGGGCTAA 113

||||||||||||||||||||||||||||||||||||||||||||||||||||||||||||

Sbjct 308011 TCAGTAACACGTCGGTAACCTACCCTCGGGAGGGGGATAACCCCGGGAAACTGGGGCTAA 308070

Query 114 TCCCCCATAGGCCTGAGGTACTGGAAGGTCCTCAGGCCGAAAGGGG--C-T-CTG-CCCG 168

||||||||||||||| ||||||||||||||| |||||||||||||| | | | ||||

Sbjct 308071 TCCCCCATAGGCCTGGGGTACTGGAAGGTCCCCAGGCCGAAAGGGGACCGTAAGGTCCCG 308130

Query 169 CCCGAGGATGGGCCGGCGGCCGATTAGGTAGTTGGTGGGGTAACGGCCCACCAAGCCGAA 228

||||||||||||||||||||||||||||||||||||||||||||||||||||||||||||

Sbjct 308131 CCCGAGGATGGGCCGGCGGCCGATTAGGTAGTTGGTGGGGTAACGGCCCACCAAGCCGAA 308190

Query 229 GATCGGTACGGGCCATGAGAGTGGGAGCCCGGAGATGGACACTGAGACACGGGTCCAGGC 288

|||||||||||||| |||||| ||||||||||||||||||||||||||||||||||||||

Sbjct 308191 GATCGGTACGGGCCGTGAGAGCGGGAGCCCGGAGATGGACACTGAGACACGGGTCCAGGC 308250

Query 289 CCTACGGGGCGCAGCAGGCGCGAAACCTCCGCAATGCGGGCAACCGCGACGGGGGGACCC 348

|||||||||||||||||||||||||||||||||||||||| |||||||||||||||||||

Sbjct 308251 CCTACGGGGCGCAGCAGGCGCGAAACCTCCGCAATGCGGGAAACCGCGACGGGGGGACCC 308310

Query 349 CCAGTGCCGTGGCAACGCCACGGCTTTTCCGGAGTGTAAAAAGCTCCGGGAATAAGGGCT 408

||||||||||| | | ||||||||||||||||||||||||||||||||||||||||||

Sbjct 308311 CCAGTGCCGTGCCTCTGGCACGGCTTTTCCGGAGTGTAAAAAGCTCCGGGAATAAGGGCT 308370

Query 409 GGGCAAGGCCGGTGGCAGCCGCCGCGGTAATACCGGCGGCCCGAGTGGTGGCCGCTATTA 468

||||||||||||||||||||||||||||||||||||||||||||||||||||| ||||||

Sbjct 308371 GGGCAAGGCCGGTGGCAGCCGCCGCGGTAATACCGGCGGCCCGAGTGGTGGCCACTATTA 308430

Query 469 TTGGGCCTAAAGCGTCCGTAGCCGGGCCCGTAAGTCCCTGGCGAAATCCCACGGCTCAAC 528

|||||||||||||| |||||||||||||||||||||||||||||||||||||||||||||

Sbjct 308431 TTGGGCCTAAAGCGGCCGTAGCCGGGCCCGTAAGTCCCTGGCGAAATCCCACGGCTCAAC 308490

Query 529 CGTGGGGCTTGCTGGGGATACTGCGGGCCTTGGGACCGGGAGAGGCCGGGGGTACCCCTG 588

||||||||| |||||||||||||||||||||||||||||||||||||||||||||||| |

Sbjct 308491 CGTGGGGCTCGCTGGGGATACTGCGGGCCTTGGGACCGGGAGAGGCCGGGGGTACCCCCG 308550

Query 589 GGGTAGGGGTGAAATCCTATAATCCCAGGGGGACCGCCAGTGGCGAAGGCGCCCGGCTGG 648

|||||||||||||||||||||||||| |||||||||||||||||||||||||||||||||

Sbjct 308551 GGGTAGGGGTGAAATCCTATAATCCCGGGGGGACCGCCAGTGGCGAAGGCGCCCGGCTGG 308610

Query 649 AACGGGTCCGACGGTGAGGGACGAAGGCCAGGGGAGCGAACCGGATTAGATACCCGGGTA 708

|||||||||||||||||||| |||||||||||||||||||||||||||||||||||||||

Sbjct 308611 AACGGGTCCGACGGTGAGGGCCGAAGGCCAGGGGAGCGAACCGGATTAGATACCCGGGTA 308670

Query 709 GTCCTGGCTGTAAAGGATGCGGGCTAGGTGTCGGGCGAGCTTCGAGCTCGCCCGGTGCCG 768

||||||||||||||||||||||||||||||||||||||||||||||||||||||||||||

Sbjct 308671 GTCCTGGCTGTAAAGGATGCGGGCTAGGTGTCGGGCGAGCTTCGAGCTCGCCCGGTGCCG 308730

Query 769 AAGGGAAGCCGTTAAGCCCGCCGCCTGGGGAGTACGGCCGCAAGGCTGAAACTTAAAGGA 828

|||||||||||||||||||||||||||||||||||||||||||||||||||||||||||

Sbjct 308731 TAGGGAAGCCGTTAAGCCCGCCGCCTGGGGAGTACGGCCGCAAGGCTGAAACTTAAAGGA 308790

Query 829 ATTGGCGGGGGAGCACTACAAGGGGTGGAGCGTGCGGTTTAATTGGATTCAACGCCGGGA 888

||||||||||||||||||||||||||||||||||||||||||||||||||||||||||||

Sbjct 308791 ATTGGCGGGGGAGCACTACAAGGGGTGGAGCGTGCGGTTTAATTGGATTCAACGCCGGGA 308850

Query 889 ACCTCACCGGGGGCGACGGCAGGATGAAGGCCAGGCTGAAGGTCTTGCCGGACACGCCGA 948

||||||||||||||||||||||||||||||||||||||||||||||||||||| ||||||

Sbjct 308851 ACCTCACCGGGGGCGACGGCAGGATGAAGGCCAGGCTGAAGGTCTTGCCGGACGCGCCGA 308910

Query 949 GAGGAGGTGCATGGCCGCCGTCAGCTCGTACCGTGAGGCGTCCACTTAAGTGTGGTAACG 1008

||||||||||||||||||||||||||||||||||||||||||||||||||||||||||||

Sbjct 308911 GAGGAGGTGCATGGCCGCCGTCAGCTCGTACCGTGAGGCGTCCACTTAAGTGTGGTAACG 308970

Query 1009 AGCGAGACCCGCGCCCCCCAGTTGCCAGTCCTTCCCGCTGGGGAGGAGGCACTCTGGGGG 1068

||||||||||||| ||||||||||||||||| |||||||||| ||||||||||||||||

Sbjct 308971 AGCGAGACCCGCG-CCCCCAGTTGCCAGTCCCTCCCGCTGGGAGGGAGGCACTCTGGGGG 309029

Query 1069 GACCGCCGGCGATAAGCCGGAGGAAGGAGCGGGCGACGGTAGGTCAGTATGCCCCGAAAC 1128

||| ||||||||||||||||||||||| ||||||||||||||||||||||||||||||||

Sbjct 309030 GACTGCCGGCGATAAGCCGGAGGAAGGGGCGGGCGACGGTAGGTCAGTATGCCCCGAAAC 309089

Query 1129 CCCCGGGCTACACGCGCGCTACAATGGGCGGGACAATGGGATCCGACCCCGAAAGGGGAA 1188

||||||||||||||||||||||||||||||||||||||||| ||||||||||||||||||

Sbjct 309090 CCCCGGGCTACACGCGCGCTACAATGGGCGGGACAATGGGACCCGACCCCGAAAGGGGAA 309149

Query 1189 GGGAATCCCCTAAACCCGCCCCCAGTTCGGATCGCGGGCTGCAACTCGCCCGCGTGAAGC 1248

||||||||||||||||||||| ||||||||||||||||||||||||||||||||||||||

Sbjct 309150 GGGAATCCCCTAAACCCGCCCTCAGTTCGGATCGCGGGCTGCAACTCGCCCGCGTGAAGC 309209

Query 1249 TGGAATCCCTAGTACCCGCGTGTCATCATCGCGCGGCGAATACGTCCCTGCTCCTTGCAC 1308

|||||||||||||||||||| |||||||||||||||||||||||||||||||||||||||

Sbjct 309210 TGGAATCCCTAGTACCCGCGCGTCATCATCGCGCGGCGAATACGTCCCTGCTCCTTGCAC 309269

Query 1309 ACACCGCCCGTCA 1321

|||||||||||||

Sbjct 309270 ACACCGCCCGTCA 309282

> gi|851302729|ref|NZ_CP006019.1| Palaeococcus pacificus DY20341

chromosome, complete genome

Length=1859370

Score = 2174 bits (1177), Expect = 0.0

Identities = 1274/1321 (96%), Gaps = 6/1321 (0%)

Strand=Plus/Plus

Query 1 GTCCGACTAAGCCATGCGAGTCATGGGGCGCCTTGCGCGCACCGGCGGACGGCTCAGTAA 60

||||||||||||||||||||||| |||||| |||||||||||||||| ||||||||||||

Sbjct 763225 GTCCGACTAAGCCATGCGAGTCAAGGGGCG-CTTGCGCGCACCGGCGTACGGCTCAGTAA 763283

Query 61 CACGTCGGTAACCTACCCTCGGGAGGGGGATAACCCCGGGAAACTGGGGCTAATCCCCCA 120

|||||||||||||||||||||||||||||| |||||||||||||||||||||||||||||

Sbjct 763284 CACGTCGGTAACCTACCCTCGGGAGGGGGACAACCCCGGGAAACTGGGGCTAATCCCCCA 763343

Query 121 TAGGCCTGAGGTACTGGAAGGTCCTCAGGCCGAAAGGGGCTCTGCCCGCCCGAGGATGGG 180

||||| || |||||||||| |||| || |||||||| ||| | ||||||||||||||

Sbjct 763344 TAGGCTTGGGGTACTGGAAAGTCCCCAAGCCGAAAG---CTCCG-GCGCCCGAGGATGGG 763399

Query 181 CCGGCGGCCGATTAGGTAGTTGGTGGGGTAACGGCCCACCAAGCCGAAGATCGGTACGGG 240

||||||||||||||||||||||||||||||||||||||||||||||| |||||||||||

Sbjct 763400 CCGGCGGCCGATTAGGTAGTTGGTGGGGTAACGGCCCACCAAGCCGACAATCGGTACGGG 763459

Query 241 CCATGAGAGTGGGAGCCCGGAGATGGACACTGAGACACGGGTCCAGGCCCTACGGGGCGC 300

| |||||| |||||||||||||||||||||||||||||||||||||||||||||||||

Sbjct 763460 CAGTGAGAGCTGGAGCCCGGAGATGGACACTGAGACACGGGTCCAGGCCCTACGGGGCGC 763519

Query 301 AGCAGGCGCGAAACCTCCGCAATGCGGGCAACCGCGACGGGGGGACCCCCAGTGCCGTGG 360

|||||||||||||||||||||||||||| |||||||||||||||||||| ||||||||||

Sbjct 763520 AGCAGGCGCGAAACCTCCGCAATGCGGGAAACCGCGACGGGGGGACCCCGAGTGCCGTGG 763579

Query 361 CAACGCCACGGCTTTTCCGGAGTGTAAAAAGCTCCGGGAATAAGGGCTGGGCAAGGCCGG 420

|| |||||||||||||||||||| || |||||||||||||||||||||||||||||||

Sbjct 763580 CACAGCCACGGCTTTTCCGGAGTGCAAGGAGCTCCGGGAATAAGGGCTGGGCAAGGCCGG 763639

Query 421 TGGCAGCCGCCGCGGTAATACCGGCGGCCCGAGTGGTGGCCGCTATTATTGGGCCTAAAG 480

|||||||||||||||||||||||||||||| |||||||||| ||||||||||||||||||

Sbjct 763640 TGGCAGCCGCCGCGGTAATACCGGCGGCCCAAGTGGTGGCCACTATTATTGGGCCTAAAG 763699

Query 481 CGTCCGTAGCCGGGCCCGTAAGTCCCTGGCGAAATCCCACGGCTCAACCGTGGGGCTTGC 540

|||||||||||||||| |||||||||||||||||||||||||||||||||||||||||||

Sbjct 763700 CGTCCGTAGCCGGGCCTGTAAGTCCCTGGCGAAATCCCACGGCTCAACCGTGGGGCTTGC 763759

Query 541 TGGGGATACTGCGGGCCTTGGGACCGGGAGAGGCCGGGGGTACCCCTGGGGTAGGGGTGA 600

|||||||||||| |||||||||||||||||||||||||||||||||||||||||||||||

Sbjct 763760 TGGGGATACTGCAGGCCTTGGGACCGGGAGAGGCCGGGGGTACCCCTGGGGTAGGGGTGA 763819

Query 601 AATCCTATAATCCCAGGGGGACCGCCAGTGGCGAAGGCGCCCGGCTGGAACGGGTCCGAC 660

||||||||||||||||||||||||||||||||||||||||||||||||||||||||||||

Sbjct 763820 AATCCTATAATCCCAGGGGGACCGCCAGTGGCGAAGGCGCCCGGCTGGAACGGGTCCGAC 763879

Query 661 GGTGAGGGACGAAGGCCAGGGGAGCGAACCGGATTAGATACCCGGGTAGTCCTGGCTGTA 720

||||||||||||||||||||||||| ||||||||||||||||||||||||||||||||||

Sbjct 763880 GGTGAGGGACGAAGGCCAGGGGAGCAAACCGGATTAGATACCCGGGTAGTCCTGGCTGTA 763939

Query 721 AAGGATGCGGGCTAGGTGTCGGGCGAGCTTCGAGCTCGCCCGGTGCCGAAGGGAAGCCGT 780

|||||||||||||||||||||||||||| |||||||| |||||||||| |||||||||||

Sbjct 763940 AAGGATGCGGGCTAGGTGTCGGGCGAGCCTCGAGCTCACCCGGTGCCGCAGGGAAGCCGT 763999

Query 781 TAAGCCCGCCGCCTGGGGAGTACGGCCGCAAGGCTGAAACTTAAAGGAATTGGCGGGGGA 840

||||||||||||||||||||||||||||||||||||||||||||||||||||||||||||

Sbjct 764000 TAAGCCCGCCGCCTGGGGAGTACGGCCGCAAGGCTGAAACTTAAAGGAATTGGCGGGGGA 764059

Query 841 GCACTACAAGGGGTGGAGCGTGCGGTTTAATTGGATTCAACGCCGGGAACCTCACCGGGG 900

||||||||||||||||||||||||||||||||||||||||||||||||||||||||||||

Sbjct 764060 GCACTACAAGGGGTGGAGCGTGCGGTTTAATTGGATTCAACGCCGGGAACCTCACCGGGG 764119

Query 901 GCGACGGCAGGATGAAGGCCAGGCTGAAGGTCTTGCCGGACACGCCGAGAGGAGGTGCAT 960

||||||||||||||||||||||||||||||||||||||||||||||||||||||||||||

Sbjct 764120 GCGACGGCAGGATGAAGGCCAGGCTGAAGGTCTTGCCGGACACGCCGAGAGGAGGTGCAT 764179

Query 961 GGCCGCCGTCAGCTCGTACCGTGAGGCGTCCACTTAAGTGTGGTAACGAGCGAGACCCGC 1020

||||||||||||||||||||||||||||||||||||||||||||||||||||||||||||

Sbjct 764180 GGCCGCCGTCAGCTCGTACCGTGAGGCGTCCACTTAAGTGTGGTAACGAGCGAGACCCGC 764239

Query 1021 GCCCCCCAGTTGCCAGTCCTTCCCGCTGGGGAGGAGGCACTCTGGGGGGACCGCCGGCGA 1080

| |||||||||||||| || ||||| |||| ||||||||||||||||||| ||||||||

Sbjct 764240 G-CCCCCAGTTGCCAGCCCCTCCCGTTGGGAGGGAGGCACTCTGGGGGGACTGCCGGCGA 764298

Query 1081 TAAGCCGGAGGAAGGAGCGGGCGACGGTAGGTCAGTATGCCCCGAAACCCCCGGGCTACA 1140

||||||||||||||||||||||||||||||||||||||||||||||||||||||||||||

Sbjct 764299 TAAGCCGGAGGAAGGAGCGGGCGACGGTAGGTCAGTATGCCCCGAAACCCCCGGGCTACA 764358

Query 1141 CGCGCGCTACAATGGGCGGGACAATGGGATCCGACCCCGAAAGGGGAAGGGAATCCCCTA 1200

||||||||||||||||||||||||||||| |||||||||||||||||||| |||||||||

Sbjct 764359 CGCGCGCTACAATGGGCGGGACAATGGGAACCGACCCCGAAAGGGGAAGGAAATCCCCTA 764418

Query 1201 AACCCGCCCCCAGTTCGGATCGCGGGCTGCAACTCGCCCGCGTGAAGCTGGAATCCCTAG 1260

|||||||||||||||||||| |||||||||||||||||||| ||||||||||||||||||

Sbjct 764419 AACCCGCCCCCAGTTCGGATTGCGGGCTGCAACTCGCCCGCATGAAGCTGGAATCCCTAG 764478

Query 1261 TACCCGCGTGTCATCATCGCGCGGCGAATACGTCCCTGCTCCTTGCACACACCGCCCGTC 1320

||||||||||||||||||||||||||||||||||||||||||||||||||||||||||||

Sbjct 764479 TACCCGCGTGTCATCATCGCGCGGCGAATACGTCCCTGCTCCTTGCACACACCGCCCGTC 764538

Query 1321 A 1321

|

Sbjct 764539 A 764539

Score = 2174 bits (1177), Expect = 0.0

Identities = 1274/1321 (96%), Gaps = 6/1321 (0%)

Strand=Plus/Minus

Query 1 GTCCGACTAAGCCATGCGAGTCATGGGGCGCCTTGCGCGCACCGGCGGACGGCTCAGTAA 60

||||||||||||||||||||||| ||||||| ||||||||||||||| ||||||||||||

Sbjct 1528803 GTCCGACTAAGCCATGCGAGTCAAGGGGCGC-TTGCGCGCACCGGCGTACGGCTCAGTAA 1528745

Query 61 CACGTCGGTAACCTACCCTCGGGAGGGGGATAACCCCGGGAAACTGGGGCTAATCCCCCA 120

|||||||||||||||||||||||||||||| |||||||||||||||||||||||||||||

Sbjct 1528744 CACGTCGGTAACCTACCCTCGGGAGGGGGACAACCCCGGGAAACTGGGGCTAATCCCCCA 1528685

Query 121 TAGGCCTGAGGTACTGGAAGGTCCTCAGGCCGAAAGGGGCTCTGCCCGCCCGAGGATGGG 180

||||| || |||||||||| |||| || |||||||| ||| | ||||||||||||||

Sbjct 1528684 TAGGCTTGGGGTACTGGAAAGTCCCCAAGCCGAAAG---CTCCG-GCGCCCGAGGATGGG 1528629

Query 181 CCGGCGGCCGATTAGGTAGTTGGTGGGGTAACGGCCCACCAAGCCGAAGATCGGTACGGG 240

||||||||||||||||||||||||||||||||||||||||||||||| |||||||||||

Sbjct 1528628 CCGGCGGCCGATTAGGTAGTTGGTGGGGTAACGGCCCACCAAGCCGACAATCGGTACGGG 1528569

Query 241 CCATGAGAGTGGGAGCCCGGAGATGGACACTGAGACACGGGTCCAGGCCCTACGGGGCGC 300

| |||||| |||||||||||||||||||||||||||||||||||||||||||||||||

Sbjct 1528568 CAGTGAGAGCTGGAGCCCGGAGATGGACACTGAGACACGGGTCCAGGCCCTACGGGGCGC 1528509

Query 301 AGCAGGCGCGAAACCTCCGCAATGCGGGCAACCGCGACGGGGGGACCCCCAGTGCCGTGG 360

|||||||||||||||||||||||||||| |||||||||||||||||||| ||||||||||

Sbjct 1528508 AGCAGGCGCGAAACCTCCGCAATGCGGGAAACCGCGACGGGGGGACCCCGAGTGCCGTGG 1528449

Query 361 CAACGCCACGGCTTTTCCGGAGTGTAAAAAGCTCCGGGAATAAGGGCTGGGCAAGGCCGG 420

|| |||||||||||||||||||| || |||||||||||||||||||||||||||||||

Sbjct 1528448 CACAGCCACGGCTTTTCCGGAGTGCAAGGAGCTCCGGGAATAAGGGCTGGGCAAGGCCGG 1528389

Query 421 TGGCAGCCGCCGCGGTAATACCGGCGGCCCGAGTGGTGGCCGCTATTATTGGGCCTAAAG 480

|||||||||||||||||||||||||||||| |||||||||| ||||||||||||||||||

Sbjct 1528388 TGGCAGCCGCCGCGGTAATACCGGCGGCCCAAGTGGTGGCCACTATTATTGGGCCTAAAG 1528329

Query 481 CGTCCGTAGCCGGGCCCGTAAGTCCCTGGCGAAATCCCACGGCTCAACCGTGGGGCTTGC 540

|||||||||||||||| |||||||||||||||||||||||||||||||||||||||||||

Sbjct 1528328 CGTCCGTAGCCGGGCCTGTAAGTCCCTGGCGAAATCCCACGGCTCAACCGTGGGGCTTGC 1528269

Query 541 TGGGGATACTGCGGGCCTTGGGACCGGGAGAGGCCGGGGGTACCCCTGGGGTAGGGGTGA 600

|||||||||||| |||||||||||||||||||||||||||||||||||||||||||||||

Sbjct 1528268 TGGGGATACTGCAGGCCTTGGGACCGGGAGAGGCCGGGGGTACCCCTGGGGTAGGGGTGA 1528209

Query 601 AATCCTATAATCCCAGGGGGACCGCCAGTGGCGAAGGCGCCCGGCTGGAACGGGTCCGAC 660

||||||||||||||||||||||||||||||||||||||||||||||||||||||||||||

Sbjct 1528208 AATCCTATAATCCCAGGGGGACCGCCAGTGGCGAAGGCGCCCGGCTGGAACGGGTCCGAC 1528149

Query 661 GGTGAGGGACGAAGGCCAGGGGAGCGAACCGGATTAGATACCCGGGTAGTCCTGGCTGTA 720

||||||||||||||||||||||||| ||||||||||||||||||||||||||||||||||

Sbjct 1528148 GGTGAGGGACGAAGGCCAGGGGAGCAAACCGGATTAGATACCCGGGTAGTCCTGGCTGTA 1528089

Query 721 AAGGATGCGGGCTAGGTGTCGGGCGAGCTTCGAGCTCGCCCGGTGCCGAAGGGAAGCCGT 780

|||||||||||||||||||||||||||| |||||||| |||||||||| |||||||||||

Sbjct 1528088 AAGGATGCGGGCTAGGTGTCGGGCGAGCCTCGAGCTCACCCGGTGCCGCAGGGAAGCCGT 1528029

Query 781 TAAGCCCGCCGCCTGGGGAGTACGGCCGCAAGGCTGAAACTTAAAGGAATTGGCGGGGGA 840

||||||||||||||||||||||||||||||||||||||||||||||||||||||||||||

Sbjct 1528028 TAAGCCCGCCGCCTGGGGAGTACGGCCGCAAGGCTGAAACTTAAAGGAATTGGCGGGGGA 1527969

Query 841 GCACTACAAGGGGTGGAGCGTGCGGTTTAATTGGATTCAACGCCGGGAACCTCACCGGGG 900

||||||||||||||||||||||||||||||||||||||||||||||||||||||||||||

Sbjct 1527968 GCACTACAAGGGGTGGAGCGTGCGGTTTAATTGGATTCAACGCCGGGAACCTCACCGGGG 1527909

Query 901 GCGACGGCAGGATGAAGGCCAGGCTGAAGGTCTTGCCGGACACGCCGAGAGGAGGTGCAT 960

||||||||||||||||||||||||||||||||||||||||||||||||||||||||||||

Sbjct 1527908 GCGACGGCAGGATGAAGGCCAGGCTGAAGGTCTTGCCGGACACGCCGAGAGGAGGTGCAT 1527849

Query 961 GGCCGCCGTCAGCTCGTACCGTGAGGCGTCCACTTAAGTGTGGTAACGAGCGAGACCCGC 1020

||||||||||||||||||||||||||||||||||||||||||||||||||||||||||||

Sbjct 1527848 GGCCGCCGTCAGCTCGTACCGTGAGGCGTCCACTTAAGTGTGGTAACGAGCGAGACCCGC 1527789

Query 1021 GCCCCCCAGTTGCCAGTCCTTCCCGCTGGGGAGGAGGCACTCTGGGGGGACCGCCGGCGA 1080

|||||| ||||||||| || ||||| |||| ||||||||||||||||||| ||||||||

Sbjct 1527788 GCCCCC-AGTTGCCAGCCCCTCCCGTTGGGAGGGAGGCACTCTGGGGGGACTGCCGGCGA 1527730

Query 1081 TAAGCCGGAGGAAGGAGCGGGCGACGGTAGGTCAGTATGCCCCGAAACCCCCGGGCTACA 1140

||||||||||||||||||||||||||||||||||||||||||||||||||||||||||||

Sbjct 1527729 TAAGCCGGAGGAAGGAGCGGGCGACGGTAGGTCAGTATGCCCCGAAACCCCCGGGCTACA 1527670

Query 1141 CGCGCGCTACAATGGGCGGGACAATGGGATCCGACCCCGAAAGGGGAAGGGAATCCCCTA 1200

||||||||||||||||||||||||||||| |||||||||||||||||||| |||||||||

Sbjct 1527669 CGCGCGCTACAATGGGCGGGACAATGGGAACCGACCCCGAAAGGGGAAGGAAATCCCCTA 1527610

Query 1201 AACCCGCCCCCAGTTCGGATCGCGGGCTGCAACTCGCCCGCGTGAAGCTGGAATCCCTAG 1260

|||||||||||||||||||| |||||||||||||||||||| ||||||||||||||||||

Sbjct 1527609 AACCCGCCCCCAGTTCGGATTGCGGGCTGCAACTCGCCCGCATGAAGCTGGAATCCCTAG 1527550

Query 1261 TACCCGCGTGTCATCATCGCGCGGCGAATACGTCCCTGCTCCTTGCACACACCGCCCGTC 1320

||||||||||||||||||||||||||||||||||||||||||||||||||||||||||||

Sbjct 1527549 TACCCGCGTGTCATCATCGCGCGGCGAATACGTCCCTGCTCCTTGCACACACCGCCCGTC 1527490

Query 1321 A 1321

|

Sbjct 1527489 A 1527489

> gi|261402131|ref|NC_013407.1| Methanocaldococcus vulcanius M7,

complete sequence

Length=1746329

Score = 1565 bits (847), Expect = 0.0

Identities = 1172/1329 (88%), Gaps = 22/1329 (2%)

Strand=Plus/Plus

Query 1 GTCCGACTAAGCCATGCGAGTCATGGGGCGCCTT-GCGCGCACCGGCGGACGGCTCAGTA 59

||||||||||||||||||||||| ||||| |||| | | ||||||||| |||||||||||

Sbjct 622180 GTCCGACTAAGCCATGCGAGTCAAGGGGCTCCTTCGGGAGCACCGGCGCACGGCTCAGTA 622239

Query 60 ACACGTCGGTAACCTACCCTCGGGAGGGGGATAACCCCGGGAAACTGGGGCTAATCCCCC 119

|||||| | ||||||||||||||||||||||||||| |||||||||| ||||||||||||

Sbjct 622240 ACACGTGGCTAACCTACCCTCGGGAGGGGGATAACCTCGGGAAACTGAGGCTAATCCCCC 622299

Query 120 ATAGG-CCTGAGGTACTGGAA-GGT-CCTCAGGCCGAAAGGGGCTCTGCCCGCCCGAGGA 176

||||| ||||| |||||| | | |||| |||||| |||| | ||||||||||

Sbjct 622300 ATAGGGGAGGAGGT-CTGGAACGATCCCTC--CCCGAAA---GCTCCG-GCGCCCGAGGA 622352

Query 177 TGGGCCGGCGGCCGATTAGGTAGTTGGTGGGGTAACGGCCCACCAAGCCGAAGATCGGTA 236

|||| | ||||| |||||||||||||||||||||||||||||||||||||| |||| |||

Sbjct 622353 TGGGGCTGCGGCGGATTAGGTAGTTGGTGGGGTAACGGCCCACCAAGCCGACGATCCGTA 622412

Query 237 CGGGCCATGAGAGTGGGAGCCCGGAGATGGACACTGAGACACGGGTCCAGGCCCTACGGG 296

|||||| |||||| ||||||||||||||||||||||||||||||||||||||||||||||

Sbjct 622413 CGGGCCCTGAGAGGGGGAGCCCGGAGATGGACACTGAGACACGGGTCCAGGCCCTACGGG 622472

Query 297 GCGCAGCAGGCGCGAAACCTCCGCAATGCGGGCAACCGCGACGGGGGGACCCCCAGTGCC 356

||||||||||||||||||||||||||||| | || |||||||||||||||| ||||||

Sbjct 622473 GCGCAGCAGGCGCGAAACCTCCGCAATGCACGAAAGTGCGACGGGGGGACCCCGAGTGCC 622532

Query 357 GTGGCAACGCCACGGCTTTTCCGGAGTGTAAAAAGCTCCGGGAATAAGGGCTGGGCAAGG 416

| ||||| | ||||||||||||||||||| ||||||||||||||||||||||||||

Sbjct 622533 ATCCCAACGGGATGGCTTTTCCGGAGTGTAAACAGCTCCGGGAATAAGGGCTGGGCAAGT 622592

Query 417 CCGGTGGCAGCCGCCGCGGTAATACCGGCGGCCCGAGTGGTGGCCGCTATTATTGGGCCT 476

|||||| |||| |||||||||||||||||||||| |||||||||| ||||||||||||||

Sbjct 622593 CCGGTGCCAGCAGCCGCGGTAATACCGGCGGCCCAAGTGGTGGCCACTATTATTGGGCCT 622652

Query 477 AAAGCGTCCGTAGCCGGGCCCGTAAGTCCCTGGCGAAATCCCACGGCTCAACCGTGGGGC 536

||||||||||||||||| || ||||||| ||| |||||| ||||||||||| ||||

Sbjct 622653 AAAGCGTCCGTAGCCGGCCCAGTAAGTCTCTGCTTAAATCCTGCGGCTCAACCGCAGGGC 622712

Query 537 TTGCTGGGGATACTGCGGGCCTTGGGACCGGGAGAGGCCGGGGGTACCCCTGGGGTAGGG 596

| || | | ||||||| || |||||||||||||||||||||||||||||| ||||||| |

Sbjct 622713 TGGCAGAG-ATACTGCTGGGCTTGGGACCGGGAGAGGCCGGGGGTACCCCAGGGGTAGCG 622771

Query 597 GTGAAATCCTATAATCCCAGGGGGACCGCCAGTGGCGAAGGCGCCCGGCTGGAACGGGTC 656

||||||| | | ||||| |||||||| || |||||||||||||||||||||||||||||

Sbjct 622772 GTGAAATGCGTTGATCCCTGGGGGACCACCTGTGGCGAAGGCGCCCGGCTGGAACGGGTC 622831

Query 657 CGACGGTGAGGGACGAAGGCCAGGGGAGCGAACCGGATTAGATACCCGGGTAGTCCTGGC 716

||||||||||||||||||||||||||||| ||||||||||||||||||||||||||||||

Sbjct 622832 CGACGGTGAGGGACGAAGGCCAGGGGAGCAAACCGGATTAGATACCCGGGTAGTCCTGGC 622891

Query 717 TGTAAAGGATGCGGGCTAGGTGTCGGG-CGAGCTTCGAGCTCGCC-CGGTGCCGAAGGGA 774

|||||| ||||| |||||||||| | || |||||| || || | ||||||||||||||

Sbjct 622892 TGTAAACTCTGCGGACTAGGTGTCGCGTCG-GCTTCGGGC-CGACGCGGTGCCGAAGGGA 622949

Query 775 AGCCGTTAAGCCCGCCGCCTGGGGAGTACGGCCGCAAGGCTGAAACTTAAAGGAATTGGC 834

|||||||||| |||||||||||||||||||| |||||| |||||||||||||||||||||

Sbjct 622950 AGCCGTTAAGTCCGCCGCCTGGGGAGTACGGTCGCAAGACTGAAACTTAAAGGAATTGGC 623009

Query 835 GGGGGAGCACTACAAGGGGTGGAGCGTGCGGTTTAATTGGATTCAACGCCGGGAACCTCA 894

||||||||||||||| ||||||||| ||||||||||||||||||||||||||| | ||||

Sbjct 623010 GGGGGAGCACTACAACGGGTGGAGCCTGCGGTTTAATTGGATTCAACGCCGGGCATCTCA 623069

Query 895 CCGGGGGCGACGGCAGGATGAAGGCCAGGCTGAAGGTCTTGCCGGACACGCCGAGAGGAG 954

|| |||||||||||||||||||||||||| ||| | |||||| ||| |||||||||| |

Sbjct 623070 CCAGGGGCGACGGCAGGATGAAGGCCAGGTTGACGACCTTGCCAGACGCGCCGAGAGGTG 623129

Query 955 GTGCATGGCCGCCGTCAGCTCGTACCGTGAGGCGTCCACTTAAGTGTGGTAACGAGCGAG 1014

||||||||||| ||||||||||||||||||||||||| |||||| |||||||||||||

Sbjct 623130 GTGCATGGCCGTCGTCAGCTCGTACCGTGAGGCGTCCTGTTAAGTCAGGTAACGAGCGAG 623189

Query 1015 ACCCGCGCCCCCCAGTTGCCAG-TCCTTCCCGCTGGGGAGGAGG-CACTCTGGGGGGACC 1072

||||| ||||| ||||| | |||| | | | ||| |||||| ||||| ||||||||

Sbjct 623190 ACCCGTGCCCCAT-GTTGCAACCTCCTCCTC-C-GGG-AGGAGGGCACTCATGGGGGACC 623245

Query 1073 GCCGGCGATAAGCCGGAGGAAGGAGCGGGCGACGGTAGGTCAGTATGCCCCGAAACCCCC 1132

||||||| ||||||||||||||| |||||| ||| ||||| | |||||||||| ||||

Sbjct 623246 GCCGGCGCTAAGCCGGAGGAAGGTGCGGGCAACGACAGGTCCGCATGCCCCGAATCCCCT 623305

Query 1133 GGGCTACACGCGCGCTACAATGGGCGGGACAATGGGATCCGACCCCGAAAGGGGAAGGGA 1192

|||||||||||| |||||||||| ||||||||||||| ||||||||||||||| || ||

Sbjct 623306 GGGCTACACGCGGGCTACAATGGCCGGGACAATGGGAAGCGACCCCGAAAGGGGGAGCGA 623365

Query 1193 ATCCCCTAAACCCGCCCCCAGTTCGGATCGCGGGCTGCAACTCGCCCGCGTGAAGCTGGA 1252

|||||||||||||| | ||| ||||||| |||||| ||||||||| |||||||| |||

Sbjct 623366 ATCCCCTAAACCCGGTCGTAGTCCGGATCGAGGGCTGTAACTCGCCCTCGTGAAGCCGGA 623425

Query 1253 ATCCCTAGTACCCGCGTGTCATCATCGCGCGGCGAATACGTCCCTGCTCCTTGCACACAC 1312

|||| ||||| |||| ||| ||| |||||| |||| ||||||||||||||||||||||

Sbjct 623426 ATCCGTAGTAATCGCGCCTCACCATGGCGCGGTGAATGCGTCCCTGCTCCTTGCACACAC 623485

Query 1313 CGCCCGTCA 1321

|||||||||

Sbjct 623486 CGCCCGTCA 623494

Score = 1565 bits (847), Expect = 0.0

Identities = 1172/1329 (88%), Gaps = 22/1329 (2%)

Strand=Plus/Minus

Query 1 GTCCGACTAAGCCATGCGAGTCATGGGGCGCCTT-GCGCGCACCGGCGGACGGCTCAGTA 59

||||||||||||||||||||||| ||||| |||| | | ||||||||| |||||||||||

Sbjct 1574799 GTCCGACTAAGCCATGCGAGTCAAGGGGCTCCTTCGGGAGCACCGGCGCACGGCTCAGTA 1574740

Query 60 ACACGTCGGTAACCTACCCTCGGGAGGGGGATAACCCCGGGAAACTGGGGCTAATCCCCC 119

|||||| | ||||||||||||||||||||||||||| |||||||||| ||||||||||||

Sbjct 1574739 ACACGTGGCTAACCTACCCTCGGGAGGGGGATAACCTCGGGAAACTGAGGCTAATCCCCC 1574680

Query 120 ATAGG-CCTGAGGTACTGGAA-GGT-CCTCAGGCCGAAAGGGGCTCTGCCCGCCCGAGGA 176

||||| ||||| |||||| | | |||| |||||| |||| | ||||||||||

Sbjct 1574679 ATAGGGGAGGAGGT-CTGGAACGATCCCTC--CCCGAAA---GCTCCG-GCGCCCGAGGA 1574627

Query 177 TGGGCCGGCGGCCGATTAGGTAGTTGGTGGGGTAACGGCCCACCAAGCCGAAGATCGGTA 236

|||| | ||||| |||||||||||||||||||||||||||||||||||||| |||| |||

Sbjct 1574626 TGGGGCTGCGGCGGATTAGGTAGTTGGTGGGGTAACGGCCCACCAAGCCGACGATCCGTA 1574567

Query 237 CGGGCCATGAGAGTGGGAGCCCGGAGATGGACACTGAGACACGGGTCCAGGCCCTACGGG 296

|||||| |||||| ||||||||||||||||||||||||||||||||||||||||||||||

Sbjct 1574566 CGGGCCCTGAGAGGGGGAGCCCGGAGATGGACACTGAGACACGGGTCCAGGCCCTACGGG 1574507

Query 297 GCGCAGCAGGCGCGAAACCTCCGCAATGCGGGCAACCGCGACGGGGGGACCCCCAGTGCC 356

||||||||||||||||||||||||||||| | || |||||||||||||||| ||||||

Sbjct 1574506 GCGCAGCAGGCGCGAAACCTCCGCAATGCACGAAAGTGCGACGGGGGGACCCCGAGTGCC 1574447

Query 357 GTGGCAACGCCACGGCTTTTCCGGAGTGTAAAAAGCTCCGGGAATAAGGGCTGGGCAAGG 416

| ||||| | ||||||||||||||||||| ||||||||||||||||||||||||||

Sbjct 1574446 ATCCCAACGGGATGGCTTTTCCGGAGTGTAAACAGCTCCGGGAATAAGGGCTGGGCAAGT 1574387

Query 417 CCGGTGGCAGCCGCCGCGGTAATACCGGCGGCCCGAGTGGTGGCCGCTATTATTGGGCCT 476

|||||| |||| |||||||||||||||||||||| |||||||||| ||||||||||||||

Sbjct 1574386 CCGGTGCCAGCAGCCGCGGTAATACCGGCGGCCCAAGTGGTGGCCACTATTATTGGGCCT 1574327

Query 477 AAAGCGTCCGTAGCCGGGCCCGTAAGTCCCTGGCGAAATCCCACGGCTCAACCGTGGGGC 536

||||||||||||||||| || ||||||| ||| |||||| ||||||||||| ||||

Sbjct 1574326 AAAGCGTCCGTAGCCGGCCCAGTAAGTCTCTGCTTAAATCCTGCGGCTCAACCGCAGGGC 1574267

Query 537 TTGCTGGGGATACTGCGGGCCTTGGGACCGGGAGAGGCCGGGGGTACCCCTGGGGTAGGG 596

| || | | ||||||| || |||||||||||||||||||||||||||||| ||||||| |

Sbjct 1574266 TGGCAGAG-ATACTGCTGGGCTTGGGACCGGGAGAGGCCGGGGGTACCCCAGGGGTAGCG 1574208

Query 597 GTGAAATCCTATAATCCCAGGGGGACCGCCAGTGGCGAAGGCGCCCGGCTGGAACGGGTC 656

||||||| | | ||||| |||||||| || |||||||||||||||||||||||||||||

Sbjct 1574207 GTGAAATGCGTTGATCCCTGGGGGACCACCTGTGGCGAAGGCGCCCGGCTGGAACGGGTC 1574148

Query 657 CGACGGTGAGGGACGAAGGCCAGGGGAGCGAACCGGATTAGATACCCGGGTAGTCCTGGC 716

||||||||||||||||||||||||||||| ||||||||||||||||||||||||||||||

Sbjct 1574147 CGACGGTGAGGGACGAAGGCCAGGGGAGCAAACCGGATTAGATACCCGGGTAGTCCTGGC 1574088

Query 717 TGTAAAGGATGCGGGCTAGGTGTCGGG-CGAGCTTCGAGCTCGCC-CGGTGCCGAAGGGA 774

|||||| ||||| |||||||||| | || |||||| || || | ||||||||||||||

Sbjct 1574087 TGTAAACTCTGCGGACTAGGTGTCGCGTCG-GCTTCGGGC-CGACGCGGTGCCGAAGGGA 1574030

Query 775 AGCCGTTAAGCCCGCCGCCTGGGGAGTACGGCCGCAAGGCTGAAACTTAAAGGAATTGGC 834

|||||||||| |||||||||||||||||||| |||||| |||||||||||||||||||||

Sbjct 1574029 AGCCGTTAAGTCCGCCGCCTGGGGAGTACGGTCGCAAGACTGAAACTTAAAGGAATTGGC 1573970

Query 835 GGGGGAGCACTACAAGGGGTGGAGCGTGCGGTTTAATTGGATTCAACGCCGGGAACCTCA 894

||||||||||||||| ||||||||| ||||||||||||||||||||||||||| | ||||

Sbjct 1573969 GGGGGAGCACTACAACGGGTGGAGCCTGCGGTTTAATTGGATTCAACGCCGGGCATCTCA 1573910

Query 895 CCGGGGGCGACGGCAGGATGAAGGCCAGGCTGAAGGTCTTGCCGGACACGCCGAGAGGAG 954

|| |||||||||||||||||||||||||| ||| | |||||| ||| |||||||||| |

Sbjct 1573909 CCAGGGGCGACGGCAGGATGAAGGCCAGGTTGACGACCTTGCCAGACGCGCCGAGAGGTG 1573850

Query 955 GTGCATGGCCGCCGTCAGCTCGTACCGTGAGGCGTCCACTTAAGTGTGGTAACGAGCGAG 1014

||||||||||| ||||||||||||||||||||||||| |||||| |||||||||||||

Sbjct 1573849 GTGCATGGCCGTCGTCAGCTCGTACCGTGAGGCGTCCTGTTAAGTCAGGTAACGAGCGAG 1573790

Query 1015 ACCCGCGCCCCCCAGTTGCCAG-TCCTTCCCGCTGGGGAGGAGG-CACTCTGGGGGGACC 1072

||||| ||||| ||||| | |||| || | ||| |||||| ||||| ||||||||

Sbjct 1573789 ACCCGTGCCCCAT-GTTGCAACCTCCT-CCTCC-GGG-AGGAGGGCACTCATGGGGGACC 1573734

Query 1073 GCCGGCGATAAGCCGGAGGAAGGAGCGGGCGACGGTAGGTCAGTATGCCCCGAAACCCCC 1132

||||||| ||||||||||||||| |||||| ||| ||||| | |||||||||| ||||

Sbjct 1573733 GCCGGCGCTAAGCCGGAGGAAGGTGCGGGCAACGACAGGTCCGCATGCCCCGAATCCCCT 1573674

Query 1133 GGGCTACACGCGCGCTACAATGGGCGGGACAATGGGATCCGACCCCGAAAGGGGAAGGGA 1192

|||||||||||| |||||||||| ||||||||||||| ||||||||||||||| || ||

Sbjct 1573673 GGGCTACACGCGGGCTACAATGGCCGGGACAATGGGAAGCGACCCCGAAAGGGGGAGCGA 1573614

Query 1193 ATCCCCTAAACCCGCCCCCAGTTCGGATCGCGGGCTGCAACTCGCCCGCGTGAAGCTGGA 1252

|||||||||||||| | ||| ||||||| |||||| ||||||||| |||||||| |||

Sbjct 1573613 ATCCCCTAAACCCGGTCGTAGTCCGGATCGAGGGCTGTAACTCGCCCTCGTGAAGCCGGA 1573554

Query 1253 ATCCCTAGTACCCGCGTGTCATCATCGCGCGGCGAATACGTCCCTGCTCCTTGCACACAC 1312

|||| ||||| |||| ||| ||| |||||| |||| ||||||||||||||||||||||

Sbjct 1573553 ATCCGTAGTAATCGCGCCTCACCATGGCGCGGTGAATGCGTCCCTGCTCCTTGCACACAC 1573494

Query 1313 CGCCCGTCA 1321

|||||||||

Sbjct 1573493 CGCCCGTCA 1573485

> gi|15668172|ref|NC_000909.1| Methanocaldococcus jannaschii DSM

2661, complete sequence

Length=1664970

Score = 1535 bits (831), Expect = 0.0

Identities = 1168/1331 (88%), Gaps = 22/1331 (2%)

Strand=Plus/Plus

Query 1 GTCCGACTAAGCCATGCGAGTCATGGGGC-GCCTT--GCGCGCACCGGCGGACGGCTCAG 57

||||||||||||||||||||||| ||||| |||| | | ||||||||| |||||||||

Sbjct 638489 GTCCGACTAAGCCATGCGAGTCAAGGGGCTCCCTTCGGGGAGCACCGGCGCACGGCTCAG 638548

Query 58 TAACACGTCGGTAACCTACCCTCGGGAGGGGGATAACCCCGGGAAACTGGGGCTAATCCC 117

|||||||| | ||||||||||||||| ||||||||||| |||||||||| ||||||||||

Sbjct 638549 TAACACGTGGCTAACCTACCCTCGGGTGGGGGATAACCTCGGGAAACTGAGGCTAATCCC 638608

Query 118 CCATAGG-CCTGAGGTACTGGAA-GGT-CCTCAGGCCGAAAGGGGCTCTGCCCGCCCGAG 174

||||||| ||||| |||||| | | |||| |||||||| | | | |||||||||

Sbjct 638609 CCATAGGGGAGGAGGT-CTGGAATGATCCCTC--CCCGAAAGGCG-TAAG-CCGCCCGAG 638663

Query 175 GATGGGCCGGCGGCCGATTAGGTAGTTGGTGGGGTAACGGCCCACCAAGCCGAAGATCGG 234

|||||| | ||||| |||||||||||||||||||||||||||||||||||| | |||| |

Sbjct 638664 GATGGGGCTGCGGCGGATTAGGTAGTTGGTGGGGTAACGGCCCACCAAGCCTACGATCCG 638723

Query 235 TACGGGCCATGAGAGTGGGAGCCCGGAGATGGACACTGAGACACGGGTCCAGGCCCTACG 294

|||||||| |||||| ||||||||||||||||||||||||||||||||||||||||||||

Sbjct 638724 TACGGGCCCTGAGAGGGGGAGCCCGGAGATGGACACTGAGACACGGGTCCAGGCCCTACG 638783

Query 295 GGGCGCAGCAGGCGCGAAACCTCCGCAATGCGGGCAACCGCGACGGGGGGACCCCCAGTG 354

|||||||||||||||||||||||||||||||| | || ||||||||||||||||| ||||

Sbjct 638784 GGGCGCAGCAGGCGCGAAACCTCCGCAATGCGCGAAAGCGCGACGGGGGGACCCCGAGTG 638843

Query 355 CCGTGGCAACGCCACGGCTTTTCCGGAGTGTAAAAAGCTCCGGGAATAAGGGCTGGGCAA 414

|| || || ||||||||||||||||||| |||||||||||||||||||||||||

Sbjct 638844 CCCACGCCCTGCGTGGGCTTTTCCGGAGTGTAAACAGCTCCGGGAATAAGGGCTGGGCAA 638903

Query 415 GGCCGGTGGCAGCCGCCGCGGTAATACCGGCGGCCCGAGTGGTGGCCGCTATTATTGGGC 474

| |||||| |||| |||||||||||||||||||||| |||||||||| || |||||||||

Sbjct 638904 GTCCGGTGCCAGCAGCCGCGGTAATACCGGCGGCCCAAGTGGTGGCCACTGTTATTGGGC 638963

Query 475 CTAAAGCGTCCGTAGCCGGGCCCGTAAGTCCCTGGCGAAATCCCACGGCTCAACCGTGGG 534

||||||||||||||||||| || ||||||| ||| |||||| ||||||||||| ||

Sbjct 638964 CTAAAGCGTCCGTAGCCGGCCCGGTAAGTCTCTGCTTAAATCCTGCGGCTCAACCGCAGG 639023

Query 535 GCTTGCTGGGGATACTGCGGGCCTTGGGACCGGGAGAGGCCGGGGGTACCCCTGGGGTAG 594

||| || | |||||||| || |||||||||||||||||||||||||||||| |||||||

Sbjct 639024 GCTGGC-AGAGATACTGCCGGGCTTGGGACCGGGAGAGGCCGGGGGTACCCCAGGGGTAG 639082

Query 595 GGGTGAAATCCTATAATCCCAGGGGGACCGCCAGTGGCGAAGGCGCCCGGCTGGAACGGG 654

|||||||| | | ||||| |||||||| || |||||||||||||||||||||||||||

Sbjct 639083 CGGTGAAATGCGTTGATCCCTGGGGGACCACCTGTGGCGAAGGCGCCCGGCTGGAACGGG 639142

Query 655 TCCGACGGTGAGGGACGAAGGCCAGGGGAGCGAACCGGATTAGATACCCGGGTAGTCCTG 714

||||||||||||||||||||||||||||||| ||||||||||||||||||||||||||||

Sbjct 639143 TCCGACGGTGAGGGACGAAGGCCAGGGGAGCAAACCGGATTAGATACCCGGGTAGTCCTG 639202

Query 715 GCTGTAAAGGATGCGGGCTAGGTGTCGGG-CGAGCTTCGAGCTCGCC-CGGTGCCGAAGG 772

|||||||| ||||| |||||||||| | || |||||| || || | ||||||||||||

Sbjct 639203 GCTGTAAACTCTGCGGACTAGGTGTCGCGTCG-GCTTCGGGC-CGACGCGGTGCCGAAGG 639260

Query 773 GAAGCCGTTAAGCCCGCCGCCTGGGGAGTACGGCCGCAAGGCTGAAACTTAAAGGAATTG 832

|||||||||||| |||||||||||||||||||| |||||| |||||||||||||||||||

Sbjct 639261 GAAGCCGTTAAGTCCGCCGCCTGGGGAGTACGGTCGCAAGACTGAAACTTAAAGGAATTG 639320

Query 833 GCGGGGGAGCACTACAAGGGGTGGAGCGTGCGGTTTAATTGGATTCAACGCCGGGAACCT 892

||||||||||||||||| ||||||||| ||||||||||||||||||||||||||| | ||

Sbjct 639321 GCGGGGGAGCACTACAACGGGTGGAGCCTGCGGTTTAATTGGATTCAACGCCGGGCATCT 639380

Query 893 CACCGGGGGCGACGGCAGGATGAAGGCCAGGCTGAAGGTCTTGCCGGACACGCCGAGAGG 952

||| |||||||||||||||||||||||||| ||| | |||||| ||| ||||||||||

Sbjct 639381 TACCAGGGGCGACGGCAGGATGAAGGCCAGGTTGACGACCTTGCCAGACGCGCCGAGAGG 639440

Query 953 AGGTGCATGGCCGCCGTCAGCTCGTACCGTGAGGCGTCCACTTAAGTGTGGTAACGAGCG 1012

|||||||||||| ||||||||||||||||||||||||| |||||| |||||||||||

Sbjct 639441 TGGTGCATGGCCGTCGTCAGCTCGTACCGTGAGGCGTCCTGTTAAGTCAGGTAACGAGCG 639500

Query 1013 AGACCCGCGCCCCCCAGTTGCCAG-TCCTTCCCGCTGGGGAGGAGG-CACTCTGGGGGGA 1070

||||||| ||||| ||||| | |||| | | | ||| |||||| ||||| ||||||

Sbjct 639501 AGACCCGTGCCCCAT-GTTGCTACCTCCTCCTC-C-GGG-AGGAGGGCACTCATGGGGGA 639556

Query 1071 CCGCCGGCGATAAGCCGGAGGAAGGAGCGGGCGACGGTAGGTCAGTATGCCCCGAAACCC 1130

||||||||| ||||||||||||||| |||||| ||| ||||| | |||||||||| |||

Sbjct 639557 CCGCCGGCGCTAAGCCGGAGGAAGGTGCGGGCAACGACAGGTCCGCATGCCCCGAATCCC 639616

Query 1131 CCGGGCTACACGCGCGCTACAATGGGCGGGACAATGGGATCCGACCCCGAAAGGGGAAGG 1190

| |||||||||||| |||||||||| ||||||||||||| ||||||||||||||| ||

Sbjct 639617 CTGGGCTACACGCGGGCTACAATGGCCGGGACAATGGGACGCGACCCCGAAAGGGGGAGC 639676

Query 1191 GAATCCCCTAAACCCGCCCCCAGTTCGGATCGCGGGCTGCAACTCGCCCGCGTGAAGCTG 1250

|||||||||||||||| | ||| ||||||| |||||| ||||||||| |||||||| |

Sbjct 639677 GAATCCCCTAAACCCGGTCGTAGTCCGGATCGAGGGCTGTAACTCGCCCTCGTGAAGCCG 639736

Query 1251 GAATCCCTAGTACCCGCGTGTCATCATCGCGCGGCGAATACGTCCCTGCTCCTTGCACAC 1310

|||||| ||||| |||| ||| ||| |||||| |||| ||||||||||||||||||||

Sbjct 639737 GAATCCGTAGTAATCGCGCCTCACCATGGCGCGGTGAATGCGTCCCTGCTCCTTGCACAC 639796

Query 1311 ACCGCCCGTCA 1321

|||||||||||

Sbjct 639797 ACCGCCCGTCA 639807

Score = 1517 bits (821), Expect = 0.0

Identities = 1165/1331 (88%), Gaps = 24/1331 (2%)

Strand=Plus/Minus

Query 1 GTCCGACTAAGCCATGCGAGTCATGGGGC-GCCTT--GCGCGCACCGGCGGACGGCTCAG 57

||||||||||||||||||||||| ||||| |||| | | ||||||||| |||||||||

Sbjct 159422 GTCCGACTAAGCCATGCGAGTCAAGGGGCTCCCTTCGGGGAGCACCGGCGCACGGCTCAG 159363

Query 58 TAACACGTCGGTAACCTACCCTCGGGAGGGGGATAACCCCGGGAAACTGGGGCTAATCCC 117

|||||||| | ||||||||||||||| ||||||||||| |||||||||| ||||||||||

Sbjct 159362 TAACACGTGGCTAACCTACCCTCGGGTGGGGGATAACCTCGGGAAACTGAGGCTAATCCC 159303

Query 118 CCATAGG-CCTGAGGTACTGGAA-GGT-CCTCAGGCCGAAAGGGGCTCTGCCCGCCCGAG 174

||||||| ||||| |||||| | | |||| |||||||| | | | | |||||||

Sbjct 159302 CCATAGGGGAGGAGGT-CTGGAATGATCCCTC--CCCGAAAGGCG-TAAG-CTGCCCGAG 159248

Query 175 GATGGGCCGGCGGCCGATTAGGTAGTTGGTGGGGTAACGGCCCACCAAGCCGAAGATCGG 234

|||||| | ||||| |||||||||||||||||||||||||||||||||||| | |||| |

Sbjct 159247 GATGGGGCTGCGGCGGATTAGGTAGTTGGTGGGGTAACGGCCCACCAAGCCTACGATCCG 159188

Query 235 TACGGGCCATGAGAGTGGGAGCCCGGAGATGGACACTGAGACACGGGTCCAGGCCCTACG 294

|||||||| |||||| ||||||||||||||||||||||||||||||||||||||| ||||

Sbjct 159187 TACGGGCCCTGAGAGGGGGAGCCCGGAGATGGACACTGAGACACGGGTCCAGGCC-TACG 159129

Query 295 GGGCGCAGCAGGCGCGAAACCTCCGCAATGCGGGCAACCGCGACGGGGGGACCCCCAGTG 354

|||||||||||||||||||||||||||||||| | || ||||||||||||||||| ||||

Sbjct 159128 GGGCGCAGCAGGCGCGAAACCTCCGCAATGCGCGAAAGCGCGACGGGGGGACCCCGAGTG 159069

Query 355 CCGTGGCAACGCCACGGCTTTTCCGGAGTGTAAAAAGCTCCGGGAATAAGGGCTGGGCAA 414

|| || || ||||||||||||||||||| |||||||||||||||||||||||||

Sbjct 159068 CCCACGCCCTGCGTGGGCTTTTCCGGAGTGTAAACAGCTCCGGGAATAAGGGCTGGGCAA 159009

Query 415 GGCCGGTGGCAGCCGCCGCGGTAATACCGGCGGCCCGAGTGGTGGCCGCTATTATTGGGC 474

| |||||| |||| |||||||||||||||||||||| |||||||||| || |||||||||

Sbjct 159008 GTCCGGTGCCAGCAGCCGCGGTAATACCGGCGGCCCAAGTGGTGGCCACTGTTATTGGGC 158949

Query 475 CTAAAGCGTCCGTAGCCGGGCCCGTAAGTCCCTGGCGAAATCCCACGGCTCAACCGTGGG 534

||||||||||||||||||| || ||||||| ||| |||| | ||||||||||| ||

Sbjct 158948 CTAAAGCGTCCGTAGCCGGCCCGGTAAGTCTCTGCTTAAAT-CTGCGGCTCAACCGCAGG 158890

Query 535 GCTTGCTGGGGATACTGCGGGCCTTGGGACCGGGAGAGGCCGGGGGTACCCCTGGGGTAG 594

||| || | |||||||| || |||||||||||||||||||||||||||||| |||||||

Sbjct 158889 GCTGGC-AGAGATACTGCCGGGCTTGGGACCGGGAGAGGCCGGGGGTACCCCAGGGGTAG 158831

Query 595 GGGTGAAATCCTATAATCCCAGGGGGACCGCCAGTGGCGAAGGCGCCCGGCTGGAACGGG 654

|||||||| | | ||||| |||||||| || |||||||||||||||||||||||||||

Sbjct 158830 CGGTGAAATGCGTTGATCCCTGGGGGACCACCTGTGGCGAAGGCGCCCGGCTGGAACGGG 158771

Query 655 TCCGACGGTGAGGGACGAAGGCCAGGGGAGCGAACCGGATTAGATACCCGGGTAGTCCTG 714

||||||||||||||||||||||||||||||| ||||||||||||||||||||||||||||

Sbjct 158770 TCCGACGGTGAGGGACGAAGGCCAGGGGAGCAAACCGGATTAGATACCCGGGTAGTCCTG 158711

Query 715 GCTGTAAAGGATGCGGGCTAGGTGTCGGG-CGAGCTTCGAGCTCGCC-CGGTGCCGAAGG 772

|||||||| ||||| |||||||||| | || |||||| || || | ||||||||||||

Sbjct 158710 GCTGTAAACTCTGCGGACTAGGTGTCGCGTCG-GCTTCGGGC-CGACGCGGTGCCGAAGG 158653

Query 773 GAAGCCGTTAAGCCCGCCGCCTGGGGAGTACGGCCGCAAGGCTGAAACTTAAAGGAATTG 832

|||||||||||| |||||||||||||||||||| |||||| |||||||||||||||||||

Sbjct 158652 GAAGCCGTTAAGTCCGCCGCCTGGGGAGTACGGTCGCAAGACTGAAACTTAAAGGAATTG 158593

Query 833 GCGGGGGAGCACTACAAGGGGTGGAGCGTGCGGTTTAATTGGATTCAACGCCGGGAACCT 892

||||||||||||||||| ||||||||| ||||||||||||||||||||||||||| | ||

Sbjct 158592 GCGGGGGAGCACTACAACGGGTGGAGCCTGCGGTTTAATTGGATTCAACGCCGGGCATCT 158533

Query 893 CACCGGGGGCGACGGCAGGATGAAGGCCAGGCTGAAGGTCTTGCCGGACACGCCGAGAGG 952

||| |||||||||||||||||||||||||| ||| | |||||| ||| ||||||||||

Sbjct 158532 TACCAGGGGCGACGGCAGGATGAAGGCCAGGTTGACGACCTTGCCAGACGCGCCGAGAGG 158473

Query 953 AGGTGCATGGCCGCCGTCAGCTCGTACCGTGAGGCGTCCACTTAAGTGTGGTAACGAGCG 1012

|||||||||||| ||||||||||||||||||||||||| |||||| |||||||||||

Sbjct 158472 TGGTGCATGGCCGTCGTCAGCTCGTACCGTGAGGCGTCCTGTTAAGTCAGGTAACGAGCG 158413

Query 1013 AGACCCGCGCCCCCCAGTTGCCAG-TCCTTCCCGCTGGGGAGGAGG-CACTCTGGGGGGA 1070

||||||| ||||| ||||| | |||| || | ||| |||||| ||||| ||||||

Sbjct 158412 AGACCCGTGCCCCAT-GTTGCTACCTCCT-CCTCC-GGG-AGGAGGGCACTCATGGGGGA 158357

Query 1071 CCGCCGGCGATAAGCCGGAGGAAGGAGCGGGCGACGGTAGGTCAGTATGCCCCGAAACCC 1130

||||||||| ||||||||||||||| |||||| ||| ||||| | |||||||||| |||

Sbjct 158356 CCGCCGGCGCTAAGCCGGAGGAAGGTGCGGGCAACGACAGGTCCGCATGCCCCGAATCCC 158297

Query 1131 CCGGGCTACACGCGCGCTACAATGGGCGGGACAATGGGATCCGACCCCGAAAGGGGAAGG 1190

| |||||||||||| |||||||||| ||||||||||||| ||||||||||||||| ||

Sbjct 158296 CTGGGCTACACGCGGGCTACAATGGCCGGGACAATGGGACGCGACCCCGAAAGGGGGAGC 158237

Query 1191 GAATCCCCTAAACCCGCCCCCAGTTCGGATCGCGGGCTGCAACTCGCCCGCGTGAAGCTG 1250

|||||||||||||||| | ||| ||||||| |||||| ||||||||| |||||||| |

Sbjct 158236 GAATCCCCTAAACCCGGTCGTAGTCCGGATCGAGGGCTGTAACTCGCCCTCGTGAAGCCG 158177

Query 1251 GAATCCCTAGTACCCGCGTGTCATCATCGCGCGGCGAATACGTCCCTGCTCCTTGCACAC 1310

|||||| ||||| |||| ||| ||| |||||| |||| ||||||||||||||||||||

Sbjct 158176 GAATCCGTAGTAATCGCGCCTCACCATGGCGCGGTGAATGCGTCCCTGCTCCTTGCACAC 158117

Query 1311 ACCGCCCGTCA 1321

|||||||||||

Sbjct 158116 ACCGCCCGTCA 158106

> gi|851171576|ref|NZ_CP006577.1| Archaeoglobus fulgidus DSM 8774

chromosome, complete genome

Length=2316287

Score = 1448 bits (784), Expect = 0.0

Identities = 1129/1295 (87%), Gaps = 25/1295 (2%)

Strand=Plus/Minus

Query 39 GCACCGGCGGACGGCTCAGTAACACGTCGGTAACCTACCCTCGGGAGGGGGATAACCCCG 98

||||||||||||||||||||||||||| | ||||| |||||||| ||||||||||||||

Sbjct 1952477 GCACCGGCGGACGGCTCAGTAACACGTGGACAACCTGCCCTCGGGTGGGGGATAACCCCG 1952418

Query 99 GGAAACTGGGGCTAATCCCCCATAGG-CCTGAGGTACTGGAAGGTCCTCA-GGCCGAAAG 156

|||||||||||||||||||||||||| || |||||||||| ||| ||| ||||||

Sbjct 1952417 GGAAACTGGGGCTAATCCCCCATAGGGGATG-GGTACTGGAATGTC-TCATCTCCGAAA- 1952361

Query 157 GGGCTCTGCCCGCCCGAGGATGGGCCGGCGGCCGATTAGGTAGTTGGTGGGGTAACGGCC 216

| ||| | |||||||||||||| | ||||| |||||||| ||||||||||||||||||

Sbjct 1952360 GCGCT-T-AGCGCCCGAGGATGGGTCTGCGGCGGATTAGGTTGTTGGTGGGGTAACGGCC 1952303

Query 217 CACCAAGCCGAAGATCGGTACGGGCCATGAGAGTGGGAGCCCGGAGATGGACACTGAGAC 276

|||||||||||||||| ||||||||||||||||||||||||||||||||||| |||||||

Sbjct 1952302 CACCAAGCCGAAGATCCGTACGGGCCATGAGAGTGGGAGCCCGGAGATGGACCCTGAGAC 1952243

Query 277 ACGGGTCCAGGCCCTACGGGGCGCAGCAGGCGCGAAACCTCCGCAATGCGGGCAACCGCG 336

|||||||||||||||||||||||||||||||||||||||||||||||||||| |||||||

Sbjct 1952242 ACGGGTCCAGGCCCTACGGGGCGCAGCAGGCGCGAAACCTCCGCAATGCGGGAAACCGCG 1952183

Query 337 ACGGGGGGACCCCCAGTGC-CGTGGCAACGCCACGGCTTTTCCGGAGTG--TAAAAAGC- 392

|||||| | || ||||| || ||| || | ||| | | ||| ||| ||||||||

Sbjct 1952182 ACGGGGTCAGCCGGAGTGCTCG-CGCATCG-CGCGGGCTGT-CGGGGTGCCTAAAAAGCA 1952126

Query 393 TCCGGGAATAAGGGCTGGGCAAGGCCGGTGGCAGCCGCCGCGGTAATACCGGCGGCCCGA 452

|| | |||||| ||||||||||||||||||||||||||||||||||||||||||||

Sbjct 1952125 CCCCACAGCAAGGGCCGGGCAAGGCCGGTGGCAGCCGCCGCGGTAATACCGGCGGCCCGA 1952066

Query 453 GTGGTGGCCGCTATTATTGGGCCTAAAGCGTCCGTAGCCGGGCCCGTAAGTCC-CTGGCG 511

|||| |||| || |||||||||||||||||||||||||||||| |||||||| | || |

Sbjct 1952065 GTGGCGGCCACTTTTATTGGGCCTAAAGCGTCCGTAGCCGGGCTGGTAAGTCCTCCGG-G 1952007

Query 512 AAATCCCACGGCTCAACCGTGGGGCTTGCTGG-GGATACTGCGGGCCTTGGGACCGGGAG 570

||||| ||||| |||||| | || || || ||||||||| |||| |||||||||||

Sbjct 1952006 AAATCTGGCGGCTTAACCGTCAGACT-GCCGGAGGATACTGCCAGCCTAGGGACCGGGAG 1951948

Query 571 AGGCCGGGGGTACCCCTGGGGTAGGGGTGAAATCCTATAATCCCAGGGGGACCGCCAGTG 630

|||||||||||| || || |||||||||||||||| ||||||| || ||||| || |||

Sbjct 1951947 AGGCCGGGGGTATTCCCGGAGTAGGGGTGAAATCCTGTAATCCCGGGAGGACCACCTGTG 1951888

Query 631 GCGAAGGCGCCCGGCTGGAACGGGTCCGACGGTGAGGGACGAAGGCCAGGGGAGCGAACC 690

||||||||||||||||||||||||||||||||||||||||||||||||||||||||||||

Sbjct 1951887 GCGAAGGCGCCCGGCTGGAACGGGTCCGACGGTGAGGGACGAAGGCCAGGGGAGCGAACC 1951828

Query 691 GGATTAGATACCCGGGTAGTCCTGGCTGTAAAGGATGCGGGCTAGGTGTCGGGCGA-GCT 749

|||||||||||||||||||||||||||||||| ||||||| ||||||||| ||| |||

Sbjct 1951827 GGATTAGATACCCGGGTAGTCCTGGCTGTAAACGATGCGGACTAGGTGTCAC-CGAAGCT 1951769

Query 750 TCGAGCT-CGCCCGGTGCCGAAGGGAAGCCGTTAAGCCCGCCGCCTGGGGAGTACGGCCG 808

|||||| || ||||||| ||||||||||||||| |||||||||||||||||||||||

Sbjct 1951768 ACGAGCTTCGGT-GGTGCCGGAGGGAAGCCGTTAAGTCCGCCGCCTGGGGAGTACGGCCG 1951710

Query 809 CAAGGCTGAAACTTAAAGGAATTGGCGGGGGAGCACTACAAGGGGTGGAGCGTGCGGTTT 868

||||||||||||||||||||||||||||||||||||||||| ||||||||| ||||||||

Sbjct 1951709 CAAGGCTGAAACTTAAAGGAATTGGCGGGGGAGCACTACAACGGGTGGAGCCTGCGGTTT 1951650

Query 869 AATTGGATTCAACGCCGGGAACCTCACCGGGGGCGACGGCAGGATGAAGGCCAGGCTGAA 928

||||||||||||||||||||| || |||||||| ||| || ||||||||| | |||||||

Sbjct 1951649 AATTGGATTCAACGCCGGGAAGCTTACCGGGGGAGACAGCGGGATGAAGGTCGGGCTGAA 1951590

Query 929 GGTCTTGCCGGACACGCCGAGAGGAGGTGCATGGCCGCCGTCAGCTCGTACCGTGAGGCG 988

| ||| || ||| || |||||| ||||||||||||||||||| |||||| |||| ||

Sbjct 1951589 GACCTTACCAGACTAGCTGAGAGGTGGTGCATGGCCGCCGTCAGTTCGTACTGTGAAGCA 1951530

Query 989 TCCACTTAAGTGTGGTAACGAGCGAGACCCGCGCCCCCCAGTTGCCAGTCCTTCCCG-CT 1047

||| |||||| || |||||||||||||||||||||| ||||||||| ||||| |

Sbjct 1951529 TCCTGTTAAGTCAGGCAACGAGCGAGACCCGCGCCCCC-AGTTGCCAGCGGTTCCCTTCG 1951471

Query 1048 GGGGAGGA-GGCACTCTGGGGGGACCGCCGGCGATAAGCCGGAGGAAGGAGCGGGCGACG 1106

||| || ||||| |||||||||| ||||||| ||||||||||||||| |||||| |||

Sbjct 1951470 GGGAAGCCGGGCACACTGGGGGGACTGCCGGCGCTAAGCCGGAGGAAGGTGCGGGCAACG 1951411

Query 1107 GTAGGTCAGTATGCCCCGAAACCCCCGGGCTACACGCGCGCTACAATGGGCGGGACAATG 1166

| ||||| |||||||||||| ||||||||||||||||| |||||||||| ||||||||||

Sbjct 1951410 GCAGGTCCGTATGCCCCGAATCCCCCGGGCTACACGCGGGCTACAATGGCCGGGACAATG 1951351

Query 1167 GGATCCGACCCCGAAAGGGGAAGGGAATCCCCTAAACCCGCCCCCAGTTCGGATCGCGGG 1226

|| |||||||||||||||| ||| ||||||||||||||| | | | |||||| |||

Sbjct 1951350 GGTACCGACCCCGAAAGGGGTAGGTAATCCCCTAAACCCGGTCTAACCTGGGATCGAGGG 1951291

Query 1227 CTGCAACTCGCCCGCGTGAAGCTGGAATCCCTAGTACCCGCGTGTCATCATCGCGCGGCG 1286

||||||||||||| |||||| ||||||||| ||||| |||| ||| || |||||| |

Sbjct 1951290 CTGCAACTCGCCCTCGTGAACCTGGAATCCGTAGTAATCGCGCCTCAAAATGGCGCGGTG 1951231

Query 1287 AATACGTCCCTGCTCCTTGCACACACCGCCCGTCA 1321

|||||||||||||||||||||||||||||||||||

Sbjct 1951230 AATACGTCCCTGCTCCTTGCACACACCGCCCGTCA 1951196

> gi|530779594|ref|NC_022093.1| Thermofilum adornatum, complete

sequence

Length=1750259

Score = 1227 bits (664), Expect = 0.0

Identities = 1097/1304 (84%), Gaps = 38/1304 (3%)

Strand=Plus/Minus

Query 42 CCGGCGGACGGCTCAGTAACACGTCGGTAACCTACCCTCGGGAGGGGGATAACCCCGGGA 101

|||||||||||||||||||||||| | |||||||||||||||||||||||||||||||||

Sbjct 538149 CCGGCGGACGGCTCAGTAACACGTGGCTAACCTACCCTCGGGAGGGGGATAACCCCGGGA 538090

Query 102 AACTGGGGCTAATCCCCCATAGGCCTG-AG-GTACTGGAA-GGTCCTCAGGCCGAAA-GG 157

|||||||| |||||||||||||| || || | |||||| || || | ||||||| ||

Sbjct 538089 AACTGGGGATAATCCCCCATAGG--TGTAGACTTCTGGAATGGGTCT-ACGCCGAAAGGG 538033

Query 158 ----GG---C-T--CTG-C----CCGCCCGAGGATGGGCCGGCGGCCGATTAGGTAGTTG 202

|| | | ||| | ||||||||||||||| | |||||| || |||||||||

Sbjct 538032 CTACGGCACCATCCCTGCCGTAGCCGCCCGAGGATGGGGCTGCGGCCTATCAGGTAGTTG 537973

Query 203 GTGGGGTAACGGCCCACCAAGCCGAAGATCGGTACGGGCCATGAGAGTGGGAGCCCGGAG 262

| ||||||||||||| ||||||| | | |||| ||||| |||||| |||||||| |||

Sbjct 537972 GCGGGGTAACGGCCCGCCAAGCCTATAACGGGTAGGGGCCGTGAGAGCGGGAGCCCCGAG 537913

Query 263 ATGGACACTGAGACACGGGTCCAGGCCCTACGGGGCGCAGCAGGCGCGAAACCTCCGCAA 322

|||| |||||||||| ||| ||||||||||||||| ||| |||| ||||||| |||||||

Sbjct 537912 ATGGGCACTGAGACAAGGGCCCAGGCCCTACGGGGTGCACCAGGGGCGAAACTTCCGCAA 537853

Query 323 TGCGGGCAACCGCGACGGGGGGACCCCCAGTGCCGTGGCAACG-CCACGGCTTTT-CCGG 380

|||||| ||||| ||||| | ||||| |||||| | | | ||||||| || |

Sbjct 537852 TGCGGGAAACCGTGACGGAGTCACCCCGAGTGCC-ACCCGATGAGGGTGGCTTTTGCCCG 537794

Query 381 AGTGTAAAAAGCTCCGG-GAATAAGGGCTGGGCAAGGCCGGTGGCAGCCGCCGCGGTAAT 439

|| |||||||| | || ||||||| | ||||||| |||| ||||||||||||||||

Sbjct 537793 -GTCTAAAAAGC-CGGGCGAATAAGCGGGGGGCAAGCTTGGTGTCAGCCGCCGCGGTAAT 537736

Query 440 ACCGGCGGCCCGAGTGGT--GGCCGCTATTATTGGGCCTAAAGCGTCCGTAGCCGGGCCC 497

||| | | |||||||| || || | |||||||||||||||||||||||||||| ||

Sbjct 537735 ACCAACCCCGCGAGTGGTCGGGACG-T-TTATTGGGCCTAAAGCGTCCGTAGCCGGCCCG 537678

Query 498 GTAAGTCCCTGGCGAAATCCCACGGCTCAACCGTGGGGCTTGCTG-GGGATACTGCGGGC 556

|||||||||| ||| ||||||||||||||||||| || | |||||||||| ||

Sbjct 537677 GTAAGTCCCTCCTTAAAGCCCACGGCTCAACCGTGGG---AGCGGAGGGATACTGCCGGG 537621

Query 557 CTTGGGACCGGGAGAGGCCGGGGGTACCCCTGGGGTAGGGGTGAAATCCTATAATCCCAG 616

|| ||| ||||||||||||||||||| ||||||||||||| ||||||||||||||||||

Sbjct 537620 CTAGGGGGCGGGAGAGGCCGGGGGTACTCCTGGGGTAGGGGCGAAATCCTATAATCCCAG 537561

Query 617 GGGGACCGCCAGTGGCGAAGGCGCCCGGCTGGAACGGGTCCGACGGTGAGGGACGAAGGC 676

| ||||| |||||||||||||||||||||| | ||| | |||||||||||||||||| ||

Sbjct 537560 GAGGACCACCAGTGGCGAAGGCGCCCGGCTAGCACGCGCCCGACGGTGAGGGACGAAAGC 537501

Query 677 CAGGGGAGCGAACCGGATTAGATACCCGGGTAGTCCTGGCTGTAAAGGATGCGGGCTAGG 736

||||||| || ||||||||||||| ||||||| |||||||| ||||| |||||||

Sbjct 537500 TGGGGGAGCAAAGGGGATTAGATACCCCCGTAGTCCCAGCTGTAAACGATGCAGGCTAGG 537441

Query 737 TGTCGGGCGAGCTTCGAGCTCGCCCGGTGCCGAAGGGAAGCCGTTAAGCCCGCCGCCTGG 796

||| |||| |||||| || |||| |||||| ||||||||||||||||| |||||||||

Sbjct 537440 TGTTAGGCGGGCTTCGTGCCCGCCTAGTGCCGTAGGGAAGCCGTTAAGCCTGCCGCCTGG 537381

Query 797 GGAGTACGGCCGCAAGGCTGAAACTTAAAGGAATTGGCGGGGGAGCACTACAAGGGGTGG 856

|||||||||||||||||||||||||||||||||||||||||||||||| ||||||||||

Sbjct 537380 GGAGTACGGCCGCAAGGCTGAAACTTAAAGGAATTGGCGGGGGAGCACCACAAGGGGTGA 537321

Query 857 AGCGTGCGGTTTAATTGGATTCAACGCCGGGAACCTCACCGGGGGCGACGGCAGGATGAA 916

||| ||||||||||||||| |||||||||| ||||| |||||||||||| ||||||||||

Sbjct 537320 AGCTTGCGGTTTAATTGGAGTCAACGCCGGAAACCTTACCGGGGGCGACAGCAGGATGAA 537261

Query 917 GGCCAGGCTGAAGGTCTTGCCGGACACGCCGAGAGGAGGTGCATGGCCGCCGTCAGCTCG 976

||||||||| | | |||||| ||| || ||||||||||||||||||| || | |||||

Sbjct 537260 GGCCAGGCTAACGACCTTGCCAGACGAGCTGAGAGGAGGTGCATGGCCGTCGCCGGCTCG 537201

Query 977 TACCGTGAGGCGTCCACTTAAGTGTGGTAACGAGCGAGACCCGCGCCCCCCAGTTGCCAG 1036

| |||||||| |||| |||||| || |||||||||||||| |||||| |||||| |

Sbjct 537200 TGCCGTGAGGTGTCCTGTTAAGTCAGGGAACGAGCGAGACCCCCGCCCCT-AGTTGCTAC 537142

Query 1037 TCCTTCCCGCTGGGGAGGAGGCACTCTGGGGGGACCGCCGGCGATAAGCCGGAGGAAGGA 1096

| ||| | ||| | |||||||| ||||||| |||||||||||||||||||||||

Sbjct 537141 CCGGTCCTTC-GGGACCGGGGCACTCTAGGGGGACTGCCGGCGATAAGCCGGAGGAAGGT 537083

Query 1097 GCGGGCGACGGTAGGTCAGTATGCCCCGAAACCCCCGGGCTACACGCGCGCTACAATGGG 1156

| |||| |||| |||||||||||||||||||||||||||||||||||| ||| ||||||

Sbjct 537082 GGGGGCTACGGCAGGTCAGTATGCCCCGAAACCCCCGGGCTACACGCGAGCTGCAATGGC 537023

Query 1157 CGGGACAATGGGATCCGACCCCGAAAGGGGAAGGGAATCCCCTAAACCCGCCCCCAGTTC 1216

||||||| ||| ||||||||||||||||| ||| |||||| ||||||| || ||||

Sbjct 537022 GGGGACAACGGGTTCCGACCCCGAAAGGGGGAGGCAATCCCTTAAACCCCGCCTCAGTAG 536963

Query 1217 GGATCGCGGGCTGCAACTCGCCCGCGTGAAGCTGGAATCCCTAGTACCCGCGTGTCATCA 1276

| |||| |||||||||||||||| |||||| ||||||||||||||| |||||||||||||

Sbjct 536962 GAATCGAGGGCTGCAACTCGCCCTCGTGAACCTGGAATCCCTAGTAACCGCGTGTCATCA 536903

Query 1277 TCGCGCGGCGAATACGTCCCTGCTCCTTGCACACACCGCCCGTC 1320

||||||| |||||||||||||||||||||||||||||||||||

Sbjct 536902 ACGCGCGGTGAATACGTCCCTGCTCCTTGCACACACCGCCCGTC 536859

> gi|304313778|ref|NC_014408.1| Methanothermobacter marburgensis

str. Marburg, complete sequence

Length=1634695

Score = 1195 bits (647), Expect = 0.0

Identities = 1076/1287 (84%), Gaps = 14/1287 (1%)

Strand=Plus/Plus

Query 44 GGCGGACGGCTCAGTAACACGTCGGTAACCTACCCTCGGGAGGGGGATAACCCCGGGAAA 103

|||| ||||||||||||||||| | |||||| |||| |||| |||||||||||||||||

Sbjct 433603 GGCGAACGGCTCAGTAACACGTGGATAACCTGCCCTTGGGACCGGGATAACCCCGGGAAA 433662

Query 104 CTGGGGCTAATCCCCCATAGGCCTGA-G-GTACTGGAA-GGT-CCTCAGGCCGAAAGGGG 159

|||||| ||| || ||||| ||| | | |||||| ||| | ||| ||||||

Sbjct 433663 CTGGGGATAAACCTGGATAGG--TGATGCGGCCTGGAATGGTGCTTCA--CCGAAACACC 433718

Query 160 C-TCTGCCCGCCCGAGGATGGGCCGGCGGCCGATTAGGTAGTTGGTGGGGTAACGGCCCA 218

| || | |||| |||||||| | ||||||||||||||||||||| ||||||||||| |

Sbjct 433719 CTTCGGGGTGCCCAAGGATGGGTCTGCGGCCGATTAGGTAGTTGGTAGGGTAACGGCCTA 433778

Query 219 CCAAGCCGAAGATCGGTACGGGCCATGAGAGTGGGAGCCCGGAGATGGACACTGAGACAC 278

||||||| | ||||||||||| |||||| ||||||||||||||| ||||||||

Sbjct 433779 CCAAGCCCATCATCGGTACGGGTTGTGAGAGCAAGAGCCCGGAGATGGAACCTGAGACAA 433838

Query 279 GGGTCCAGGCCCTACGGGGCGCAGCAGGCGCGAAACCTCCGCAATGCGGGCAACCGCGAC 338

|| |||||||||||||||||||||||||||||||||||||||||||| |||| |||||

Sbjct 433839 GGTTCCAGGCCCTACGGGGCGCAGCAGGCGCGAAACCTCCGCAATGCACGCAAGTGCGAC 433898

Query 339 GGGGGGACCCCCAGTGCC-GTGGCAACGCCACGGCTTTTCCGGAGTGTAAAAAGCTCCGG 397

||||| ||||| |||||| | |||| |||||||| | ||||||||||||| | |

Sbjct 433899 GGGGGAACCCCAAGTGCCACTCTTAACGGGGTGGCTTTTCAGAAGTGTAAAAAGCTTCTG 433958

Query 398 GAATAAGGGCTGGGCAAGGCCGGTGGCAGCCGCCGCGGTAATACCGGCGGCCCGAGTGGT 457

|||||||||||||||||| |||||| ||||||||||||||| |||||| || | ||||||

Sbjct 433959 GAATAAGGGCTGGGCAAGACCGGTGCCAGCCGCCGCGGTAACACCGGCAGCTCAAGTGGT 434018

Query 458 GGCCGCTATTATTGGGCCTAAAGCGTCCGTAGCCGGGCCCGTAAGTCCCTGGCGAAATCC 517

|||||| |||||||||||||||||||||||||||| | |||||| |||| |||||||

Sbjct 434019 AGCCGCTTTTATTGGGCCTAAAGCGTCCGTAGCCGGTCTGATAAGTCTCTGGTGAAATCC 434078

Query 518 CACGGCTCAACCGTGGGGCTTGCTGGGGATACT-GCGGGCCTTGGGACCGGGAGAGGCCG 576

| | ||| ||| ||||| ||||||| |||||| | | || | | |||||||||| |

Sbjct 434079 CGCAGCTTAACTGTGGGAATTGCTGGAGATACTATCATGACTCGAGGTCGGGAGAGGCTG 434138

Query 577 GGGGTACCCCTGGGGTAGGGGTGAAATCCTATAATCCCAGGGGGACCGCCAGTGGCGAAG 636

| ||||| || |||||||||||||||||| |||||| || ||||| || |||||||||

Sbjct 434139 GAGGTACTCCCAGGGTAGGGGTGAAATCCTGTAATCCTGGGAGGACCACCTGTGGCGAAG 434198

Query 637 GCGCCCGGCTGGAACGGGTCCGACGGTGAGGGACGAAGGCCAGGGGAGCGAACCGGATTA 696

||| || ||||||||| | |||||||||||||||| |||||||| |||||||||||||

Sbjct 434199 GCGTCCAGCTGGAACGAACCTGACGGTGAGGGACGAAAGCCAGGGGCGCGAACCGGATTA 434258

Query 697 GATACCCGGGTAGTCCTGGCTGTAAAGGATGCGGGCTAGGTGTCGGGCGAGCTTCGAGCT 756

|||||||||||||||||||| ||||| |||| || || ||||| ||| ||||||||||

Sbjct 434259 GATACCCGGGTAGTCCTGGCCGTAAACGATGTGGACTTGGTGTTGGGATGGCTTCGAGCT 434318

Query 757 CGCCCGGTGCCGAAGGGAAGCCGTTAAGCCCGCCGCCTGGGGAGTACGGCCGCAAGGCTG 816

||| ||||||||||||||| |||||| || ||||||||| ||||||||||||||||||

Sbjct 434319 GCCCCAGTGCCGAAGGGAAGCTGTTAAGTCCACCGCCTGGGAAGTACGGCCGCAAGGCTG 434378

Query 817 AAACTTAAAGGAATTGGCGGGGGAGCACTACAAGGGGTGGAGCGTGCGGTTTAATTGGAT 876

|||||||||||||||||||||||||||| |||| | ||||||| ||||||||||||||||

Sbjct 434379 AAACTTAAAGGAATTGGCGGGGGAGCACCACAACGCGTGGAGCCTGCGGTTTAATTGGAT 434438

Query 877 TCAACGCCGGGAACCTCACCGGGGGCGACGGCAGGATGAAGGCCAGGCTGAAGGTCTTGC 936

|||||||||| | |||||| |||||||| |||| |||| ||||||| ||| | |||||

Sbjct 434439 TCAACGCCGGACATCTCACCAGGGGCGACAGCAGTATGATGGCCAGGTTGATGACCTTGC 434498

Query 937 CGGACACGCCGAGAGGAGGTGCATGGCCGCCGTCAGCTCGTACCGTGAGGCGTCCACTTA 996

| ||| || ||||||||||||||||||||||||||||||||||||||||||||| |||

Sbjct 434499 CTGACGAGCTGAGAGGAGGTGCATGGCCGCCGTCAGCTCGTACCGTGAGGCGTCCTGTTA 434558

Query 997 AGTGTGGTAACGAGCGAGACCCGCGCCCCCCAGTTGCCAGTCCTTCCCGCTGGGGAGGA- 1055

||| || |||||||||||||| ||||| |||| |||| ||| ||||| |

Sbjct 434559 AGTCAGGCAACGAGCGAGACCCACGCCCTT-AGTTACCAGCGGGACCCTTTGGGGTTGCC 434617

Query 1056 -GGCACTCTGGGGGGACCGCCGGCGATAAGCCGGAGGAAGGAGCGGGCGACGGTAGGTCA 1114

||||| || |||||||||| | ||||| | ||||||||||| || ||||||||||||

Sbjct 434618 GGGCACACTAAGGGGACCGCCAGTGATAAACTGGAGGAAGGAGTGGACGACGGTAGGTCC 434677

Query 1115 GTATGCCCCGAAACCCCCGGGCTACACGCGCGCTACAATGGGCGGGACAATGGGATCCGA 1174

|||||||||||| |||| |||| ||||||| |||||||||| | |||||||||| |||||

Sbjct 434678 GTATGCCCCGAATCCCCTGGGCAACACGCGGGCTACAATGGCCTGGACAATGGGTTCCGA 434737

Query 1175 CCCCGAAAGGGGAAGGGAATCCCCTAAACCCGCCCCCAGTTCGGATCGCGGGCTGCAACT 1234

| | |||||| | ||| ||||||||||||| | | ||||||||||| |||||| ||||

Sbjct 434738 CACTGAAAGGTGGAGGTAATCCCCTAAACCAGGTCGTAGTTCGGATCGAGGGCTGTAACT 434797

Query 1235 CGCCCGCGTGAAGCTGGAATCCCTAGTACCCGCGTGTCATCATCGCGCGGCGAATACGTC 1294

||||| |||||||||||||| | ||||| |||||||||| ||||||||| |||||||||

Sbjct 434798 CGCCCTCGTGAAGCTGGAATGCGTAGTAATCGCGTGTCATTATCGCGCGGTGAATACGTC 434857

Query 1295 CCTGCTCCTTGCACACACCGCCCGTCA 1321

|||||||||||||||||||||||||||

Sbjct 434858 CCTGCTCCTTGCACACACCGCCCGTCA 434884

Score = 1190 bits (644), Expect = 0.0

Identities = 1075/1287 (84%), Gaps = 14/1287 (1%)

Strand=Plus/Plus

Query 44 GGCGGACGGCTCAGTAACACGTCGGTAACCTACCCTCGGGAGGGGGATAACCCCGGGAAA 103

|||| ||||||||||||||||| | |||||| |||| |||| |||||||||||||||||

Sbjct 319767 GGCGAACGGCTCAGTAACACGTGGATAACCTGCCCTTGGGACTGGGATAACCCCGGGAAA 319826

Query 104 CTGGGGCTAATCCCCCATAGGCCTGA-G-GTACTGGAA-GGT-CCTCAGGCCGAAAGGGG 159

|||||| ||| || ||||| ||| | | |||||| ||| | ||| ||||||

Sbjct 319827 CTGGGGATAAACCTGGATAGG--TGATGCGGCCTGGAATGGTGCTTCA--CCGAAACACC 319882

Query 160 C-TCTGCCCGCCCGAGGATGGGCCGGCGGCCGATTAGGTAGTTGGTGGGGTAACGGCCCA 218

| || | |||| |||||||| | ||||||||||||||||||||| ||||||||||| |

Sbjct 319883 CTTCGGGGTGCCCAAGGATGGGTCTGCGGCCGATTAGGTAGTTGGTAGGGTAACGGCCTA 319942

Query 219 CCAAGCCGAAGATCGGTACGGGCCATGAGAGTGGGAGCCCGGAGATGGACACTGAGACAC 278

||||||| | ||||||||||| |||||| ||||||||||||||| ||||||||

Sbjct 319943 CCAAGCCCATCATCGGTACGGGTTGTGAGAGCAAGAGCCCGGAGATGGAACCTGAGACAA 320002

Query 279 GGGTCCAGGCCCTACGGGGCGCAGCAGGCGCGAAACCTCCGCAATGCGGGCAACCGCGAC 338

|| |||||||||||||||||||||||||||||||||||||||||||| |||| |||||

Sbjct 320003 GGTTCCAGGCCCTACGGGGCGCAGCAGGCGCGAAACCTCCGCAATGCACGCAAGTGCGAC 320062

Query 339 GGGGGGACCCCCAGTGCC-GTGGCAACGCCACGGCTTTTCCGGAGTGTAAAAAGCTCCGG 397

||||| ||||| |||||| | |||| |||||||| | ||||||||||||| | |

Sbjct 320063 GGGGGAACCCCAAGTGCCACTCTTAACGGGGTGGCTTTTCAGAAGTGTAAAAAGCTTCTG 320122

Query 398 GAATAAGGGCTGGGCAAGGCCGGTGGCAGCCGCCGCGGTAATACCGGCGGCCCGAGTGGT 457

|||||||||||||||||| |||||| ||||||||||||||| |||||| || | ||||||

Sbjct 320123 GAATAAGGGCTGGGCAAGACCGGTGCCAGCCGCCGCGGTAACACCGGCAGCTCAAGTGGT 320182

Query 458 GGCCGCTATTATTGGGCCTAAAGCGTCCGTAGCCGGGCCCGTAAGTCCCTGGCGAAATCC 517

|||||| |||||||||||||||||||||||||||| | |||||| |||| |||||||

Sbjct 320183 AGCCGCTTTTATTGGGCCTAAAGCGTCCGTAGCCGGTCTGATAAGTCTCTGGTGAAATCC 320242

Query 518 CACGGCTCAACCGTGGGGCTTGCTGGGGATACT-GCGGGCCTTGGGACCGGGAGAGGCCG 576

| | ||| ||| ||||| ||||||| |||||| | | || | | |||||||||| |

Sbjct 320243 CGCAGCTTAACTGTGGGAATTGCTGGAGATACTATCATGACTCGAGGTCGGGAGAGGCTG 320302

Query 577 GGGGTACCCCTGGGGTAGGGGTGAAATCCTATAATCCCAGGGGGACCGCCAGTGGCGAAG 636

| ||||| || |||||||||||||||||| |||||| || ||||| || |||||||||

Sbjct 320303 GAGGTACTCCCAGGGTAGGGGTGAAATCCTGTAATCCTGGGAGGACCACCTGTGGCGAAG 320362

Query 637 GCGCCCGGCTGGAACGGGTCCGACGGTGAGGGACGAAGGCCAGGGGAGCGAACCGGATTA 696

||| || ||||||||| | |||||||||||||||| || ||||| |||||||||||||

Sbjct 320363 GCGTCCAGCTGGAACGAACCTGACGGTGAGGGACGAAAGCTAGGGGCGCGAACCGGATTA 320422

Query 697 GATACCCGGGTAGTCCTGGCTGTAAAGGATGCGGGCTAGGTGTCGGGCGAGCTTCGAGCT 756

|||||||||||||||||||| ||||| |||| || || ||||| ||| ||||||||||

Sbjct 320423 GATACCCGGGTAGTCCTGGCCGTAAACGATGTGGACTTGGTGTTGGGATGGCTTCGAGCT 320482

Query 757 CGCCCGGTGCCGAAGGGAAGCCGTTAAGCCCGCCGCCTGGGGAGTACGGCCGCAAGGCTG 816

||| ||||||||||||||| |||||| || ||||||||| ||||||||||||||||||

Sbjct 320483 GCCCCAGTGCCGAAGGGAAGCTGTTAAGTCCACCGCCTGGGAAGTACGGCCGCAAGGCTG 320542

Query 817 AAACTTAAAGGAATTGGCGGGGGAGCACTACAAGGGGTGGAGCGTGCGGTTTAATTGGAT 876

|||||||||||||||||||||||||||| |||| | ||||||| ||||||||||||||||

Sbjct 320543 AAACTTAAAGGAATTGGCGGGGGAGCACCACAACGCGTGGAGCCTGCGGTTTAATTGGAT 320602

Query 877 TCAACGCCGGGAACCTCACCGGGGGCGACGGCAGGATGAAGGCCAGGCTGAAGGTCTTGC 936

|||||||||| | |||||| |||||||| |||| |||| ||||||| ||| | |||||

Sbjct 320603 TCAACGCCGGACATCTCACCAGGGGCGACAGCAGTATGATGGCCAGGTTGATGACCTTGC 320662

Query 937 CGGACACGCCGAGAGGAGGTGCATGGCCGCCGTCAGCTCGTACCGTGAGGCGTCCACTTA 996

| ||| || ||||||||||||||||||||||||||||||||||||||||||||| |||

Sbjct 320663 CTGACGAGCTGAGAGGAGGTGCATGGCCGCCGTCAGCTCGTACCGTGAGGCGTCCTGTTA 320722

Query 997 AGTGTGGTAACGAGCGAGACCCGCGCCCCCCAGTTGCCAGTCCTTCCCGCTGGGGAGGA- 1055

||| || |||||||||||||| ||||| |||| |||| ||| ||||| |

Sbjct 320723 AGTCAGGCAACGAGCGAGACCCACGCCCTT-AGTTACCAGCGGGACCCTTTGGGGTTGCC 320781

Query 1056 -GGCACTCTGGGGGGACCGCCGGCGATAAGCCGGAGGAAGGAGCGGGCGACGGTAGGTCA 1114

||||| || |||||||||| | ||||| | ||||||||||| || ||||||||||||

Sbjct 320782 GGGCACACTAAGGGGACCGCCAGTGATAAACTGGAGGAAGGAGTGGACGACGGTAGGTCC 320841

Query 1115 GTATGCCCCGAAACCCCCGGGCTACACGCGCGCTACAATGGGCGGGACAATGGGATCCGA 1174

|||||||||||| |||| |||| ||||||| |||||||||| | |||||||||| |||||

Sbjct 320842 GTATGCCCCGAATCCCCTGGGCAACACGCGGGCTACAATGGCCTGGACAATGGGTTCCGA 320901

Query 1175 CCCCGAAAGGGGAAGGGAATCCCCTAAACCCGCCCCCAGTTCGGATCGCGGGCTGCAACT 1234

| | |||||| | ||| ||||||||||||| | | ||||||||||| |||||| ||||

Sbjct 320902 CACTGAAAGGTGGAGGTAATCCCCTAAACCAGGTCGTAGTTCGGATCGAGGGCTGTAACT 320961

Query 1235 CGCCCGCGTGAAGCTGGAATCCCTAGTACCCGCGTGTCATCATCGCGCGGCGAATACGTC 1294

||||| |||||||||||||| | ||||| |||||||||| ||||||||| |||||||||

Sbjct 320962 CGCCCTCGTGAAGCTGGAATGCGTAGTAATCGCGTGTCATTATCGCGCGGTGAATACGTC 321021

Query 1295 CCTGCTCCTTGCACACACCGCCCGTCA 1321

|||||||||||||||||||||||||||

Sbjct 321022 CCTGCTCCTTGCACACACCGCCCGTCA 321048

> gi|15678031|ref|NC_000916.1| Methanothermobacter thermautotrophicus

str. Delta H, complete sequence

Length=1751377

Score = 1194 bits (646), Expect = 0.0

Identities = 1075/1286 (84%), Gaps = 14/1286 (1%)

Strand=Plus/Plus

Query 44 GGCGGACGGCTCAGTAACACGTCGGTAACCTACCCTCGGGAGGGGGATAACCCCGGGAAA 103

|||| ||||||||||||||||| | |||||| |||| |||| |||||||||||||||||

Sbjct 1602287 GGCGAACGGCTCAGTAACACGTGGATAACCTGCCCTTGGGACCGGGATAACCCCGGGAAA 1602346

Query 104 CTGGGGCTAATCCCCCATAGGCCTGA-GGT-ACTGGAA-GG-TCCTCAGGCCGAAAGGGG 159

|||||| ||| ||| ||||| ||| | | |||||| || || ||| ||||||

Sbjct 1602347 CTGGGGATAAACCCGGATAGG--TGATGCTGCCTGGAATGGTTCTTCA--CCGAAACACC 1602402

Query 160 CTCTGCCCGCCCGAGGATGGGCCGGCGGCCGATTAGGTAGTTGGTGGGGTAACGGCCCAC 219

|| | |||| |||||||| | |||||||||||||| |||||| ||||||||||| ||

Sbjct 1602403 TTC-GGGTGCCCAAGGATGGGTCTGCGGCCGATTAGGTTGTTGGTAGGGTAACGGCCTAC 1602461

Query 220 CAAGCCGAAGATCGGTACGGGCCATGAGAGTGGGAGCCCGGAGATGGACACTGAGACACG 279

|||||||| ||||||||||| |||||| ||||||||||||||| |||||||| |

Sbjct 1602462 CAAGCCGATCATCGGTACGGGTTGTGAGAGCAAGAGCCCGGAGATGGAACCTGAGACAAG 1602521

Query 280 GGTCCAGGCCCTACGGGGCGCAGCAGGCGCGAAACCTCCGCAATGCGGGCAACCGCGACG 339

| |||||||||||||||||||||||||||||||||||||||||||| |||| ||||||

Sbjct 1602522 GTTCCAGGCCCTACGGGGCGCAGCAGGCGCGAAACCTCCGCAATGCACGCAAGTGCGACG 1602581

Query 340 GGGGGACCCCCAGTGCC-GTGGCAACGCCACGGCTTTTCCGGAGTGTAAAAAGCTCCGGG 398

|||| ||||| |||||| | |||| |||||||| | ||||||||||||| | ||

Sbjct 1602582 GGGGAACCCCAAGTGCCACTCTTAACGGGGTGGCTTTTCAGAAGTGTAAAAAGCTTCTGG 1602641

Query 399 AATAAGGGCTGGGCAAGGCCGGTGGCAGCCGCCGCGGTAATACCGGCGGCCCGAGTGGTG 458

||||||||||||||||| |||||| ||||||||||||||| |||||| || | ||||||

Sbjct 1602642 AATAAGGGCTGGGCAAGACCGGTGCCAGCCGCCGCGGTAACACCGGCAGCTCAAGTGGTA 1602701

Query 459 GCCGCTATTATTGGGCCTAAAGCGTCCGTAGCCGGGCCCGTAAGTCCCTGGCGAAATCCC 518

|||||| |||||||||||||||||||||||||||| | |||||| |||| ||||||||

Sbjct 1602702 GCCGCTTTTATTGGGCCTAAAGCGTCCGTAGCCGGTCTGATAAGTCTCTGGTGAAATCCC 1602761

Query 519 ACGGCTCAACCGTGGGGCTTGCTGGGGATACT-GCGGGCCTTGGGACCGGGAGAGGCCGG 577

|| ||| ||| ||||| ||||||| |||||| | | || | | |||||||||| ||

Sbjct 1602762 ACAGCTTAACTGTGGGAATTGCTGGAGATACTATCATGACTCGAGGTCGGGAGAGGCTGG 1602821

Query 578 GGGTACCCCTGGGGTAGGGGTGAAATCCTATAATCCCAGGGGGACCGCCAGTGGCGAAGG 637

||||| || |||||||||||||||||| |||||| || ||||| || ||||||||||

Sbjct 1602822 AGGTACTCCCAGGGTAGGGGTGAAATCCTGTAATCCTGGGAGGACCACCTGTGGCGAAGG 1602881

Query 638 CGCCCGGCTGGAACGGGTCCGACGGTGAGGGACGAAGGCCAGGGGAGCGAACCGGATTAG 697

|| || ||||||||| | |||||||||||||||| |||||||| ||||||||||||||

Sbjct 1602882 CGTCCAGCTGGAACGAACCTGACGGTGAGGGACGAAAGCCAGGGGCGCGAACCGGATTAG 1602941

Query 698 ATACCCGGGTAGTCCTGGCTGTAAAGGATGCGGGCTAGGTGTCGGGCGAGCTTCGAGCTC 757

||||||||||||||||||| ||||| |||| || || ||||| ||| ||||||||||

Sbjct 1602942 ATACCCGGGTAGTCCTGGCCGTAAACGATGTGGACTTGGTGTTGGGATGGCTTCGAGCTG 1603001

Query 758 GCCCGGTGCCGAAGGGAAGCCGTTAAGCCCGCCGCCTGGGGAGTACGGCCGCAAGGCTGA 817

||| ||||||||||||||| |||||| || ||||||||| |||||||||||||||||||

Sbjct 1603002 CCCCAGTGCCGAAGGGAAGCTGTTAAGTCCACCGCCTGGGAAGTACGGCCGCAAGGCTGA 1603061

Query 818 AACTTAAAGGAATTGGCGGGGGAGCACTACAAGGGGTGGAGCGTGCGGTTTAATTGGATT 877

||||||||||||||||||||||||||| |||| | ||||||| |||||||||||||||||

Sbjct 1603062 AACTTAAAGGAATTGGCGGGGGAGCACCACAACGCGTGGAGCCTGCGGTTTAATTGGATT 1603121

Query 878 CAACGCCGGGAACCTCACCGGGGGCGACGGCAGGATGAAGGCCAGGCTGAAGGTCTTGCC 937

||||||||| | |||||| |||||||| |||| |||| ||||||| ||| | ||||||

Sbjct 1603122 CAACGCCGGACATCTCACCAGGGGCGACAGCAGTATGATGGCCAGGTTGATGACCTTGCC 1603181

Query 938 GGACACGCCGAGAGGAGGTGCATGGCCGCCGTCAGCTCGTACCGTGAGGCGTCCACTTAA 997

||| || ||||||||||||||||||||||||||||||||||||||||||||| ||||

Sbjct 1603182 TGACGAGCTGAGAGGAGGTGCATGGCCGCCGTCAGCTCGTACCGTGAGGCGTCCTGTTAA 1603241

Query 998 GTGTGGTAACGAGCGAGACCCGCGCCCCCCAGTTGCCAGTCCTTCCCGC-TGGGGAGGA- 1055

|| || |||||||||||||| ||||| |||| |||| ||| |||| |

Sbjct 1603242 GTCAGGCAACGAGCGAGACCCACGCCCTT-AGTTACCAGCGGAACCCTTATGGGTTGCCG 1603300

Query 1056 GGCACTCTGGGGGGACCGCCGGCGATAAGCCGGAGGAAGGAGCGGGCGACGGTAGGTCAG 1115

||||| || |||||||||| | ||||| | ||||||||||| || |||||||||||| |

Sbjct 1603301 GGCACACTAAGGGGACCGCCAGTGATAAACTGGAGGAAGGAGTGGACGACGGTAGGTCCG 1603360

Query 1116 TATGCCCCGAAACCCCCGGGCTACACGCGCGCTACAATGGGCGGGACAATGGGATCCGAC 1175

||||||||||| |||| |||| ||||||| |||||||||| | |||||||||| ||||||

Sbjct 1603361 TATGCCCCGAATCCCCTGGGCAACACGCGGGCTACAATGGCCTGGACAATGGGTTCCGAC 1603420

Query 1176 CCCGAAAGGGGAAGGGAATCCCCTAAACCCGCCCCCAGTTCGGATCGCGGGCTGCAACTC 1235

|||||||| | ||| ||||||||||||| | | ||||||||||| |||||| ||| |

Sbjct 1603421 ACCGAAAGGTGGAGGTAATCCCCTAAACCAGGTCGTAGTTCGGATCGAGGGCTGTAACCC 1603480

Query 1236 GCCCGCGTGAAGCTGGAATCCCTAGTACCCGCGTGTCATCATCGCGCGGCGAATACGTCC 1295

|||| |||||||||||||| | ||||| ||||||||| ||||||||| ||||||||||

Sbjct 1603481 GCCCTCGTGAAGCTGGAATGCGTAGTAATCGCGTGTCACTATCGCGCGGTGAATACGTCC 1603540

Query 1296 CTGCTCCTTGCACACACCGCCCGTCA 1321

||||||||||||||||||||||||||

Sbjct 1603541 CTGCTCCTTGCACACACCGCCCGTCA 1603566

Score = 1188 bits (643), Expect = 0.0

Identities = 1074/1286 (84%), Gaps = 14/1286 (1%)

Strand=Plus/Plus

Query 44 GGCGGACGGCTCAGTAACACGTCGGTAACCTACCCTCGGGAGGGGGATAACCCCGGGAAA 103

|||| ||||||||||||||||| | |||||| |||| |||| |||||||||||||||||

Sbjct 1718864 GGCGAACGGCTCAGTAACACGTGGATAACCTGCCCTTGGGACCGGGATAACCCCGGGAAA 1718923

Query 104 CTGGGGCTAATCCCCCATAGGCCTGA-GGT-ACTGGAA-GG-TCCTCAGGCCGAAAGGGG 159

|||||| ||| ||| ||||| ||| | | |||||| || || ||| ||||||

Sbjct 1718924 CTGGGGATAAACCCGGATAGG--TGATGCTGCCTGGAATGGTTCTTCA--CCGAAACACC 1718979

Query 160 CTCTGCCCGCCCGAGGATGGGCCGGCGGCCGATTAGGTAGTTGGTGGGGTAACGGCCCAC 219

|| | |||| |||||||| | |||||||||||||| |||||| ||||||||||| ||

Sbjct 1718980 TTC-GGGTGCCCAAGGATGGGTCTGCGGCCGATTAGGTTGTTGGTAGGGTAACGGCCTAC 1719038

Query 220 CAAGCCGAAGATCGGTACGGGCCATGAGAGTGGGAGCCCGGAGATGGACACTGAGACACG 279

|||||||| ||||||||||| |||||| ||||||||||||||| |||||||| |

Sbjct 1719039 CAAGCCGATCATCGGTACGGGTTGTGAGAGCAAGAGCCCGGAGATGGAACCTGAGACAAG 1719098

Query 280 GGTCCAGGCCCTACGGGGCGCAGCAGGCGCGAAACCTCCGCAATGCGGGCAACCGCGACG 339

| |||||||||||||||||||||||||||||||||||||||||||| |||| ||||||

Sbjct 1719099 GTTCCAGGCCCTACGGGGCGCAGCAGGCGCGAAACCTCCGCAATGCACGCAAGTGCGACG 1719158

Query 340 GGGGGACCCCCAGTGCC-GTGGCAACGCCACGGCTTTTCCGGAGTGTAAAAAGCTCCGGG 398

|||| ||||| |||||| | |||| |||||||| | ||||||||||||| | ||

Sbjct 1719159 GGGGAACCCCAAGTGCCACTCTTAACGGGGTGGCTTTTCAGAAGTGTAAAAAGCTTCTGG 1719218

Query 399 AATAAGGGCTGGGCAAGGCCGGTGGCAGCCGCCGCGGTAATACCGGCGGCCCGAGTGGTG 458

||||||||||||||||| |||||| ||||||||||||||| |||||| || | ||||||

Sbjct 1719219 AATAAGGGCTGGGCAAGACCGGTGCCAGCCGCCGCGGTAACACCGGCAGCTCAAGTGGTA 1719278

Query 459 GCCGCTATTATTGGGCCTAAAGCGTCCGTAGCCGGGCCCGTAAGTCCCTGGCGAAATCCC 518

|||||| |||||||||||||||||||||||||||| | |||||| |||| ||||||||

Sbjct 1719279 GCCGCTTTTATTGGGCCTAAAGCGTCCGTAGCCGGTCTGATAAGTCTCTGGTGAAATCCC 1719338

Query 519 ACGGCTCAACCGTGGGGCTTGCTGGGGATACT-GCGGGCCTTGGGACCGGGAGAGGCCGG 577

|| ||| ||| ||||| ||||||| |||||| | | || | | |||||||||| ||

Sbjct 1719339 ACAGCTTAACTGTGGGAATTGCTGGAGATACTATCATGACTCGAGGTCGGGAGAGGCTGG 1719398

Query 578 GGGTACCCCTGGGGTAGGGGTGAAATCCTATAATCCCAGGGGGACCGCCAGTGGCGAAGG 637

||||| || |||||||||||||||||| |||||| || ||||| || ||||||||||

Sbjct 1719399 AGGTACTCCCAGGGTAGGGGTGAAATCCTGTAATCCTGGGAGGACCACCTGTGGCGAAGG 1719458

Query 638 CGCCCGGCTGGAACGGGTCCGACGGTGAGGGACGAAGGCCAGGGGAGCGAACCGGATTAG 697

|| || ||||||||| | |||||||||||||||| |||||||| ||||||||||||||

Sbjct 1719459 CGTCCAGCTGGAACGAACCTGACGGTGAGGGACGAAAGCCAGGGGCGCGAACCGGATTAG 1719518

Query 698 ATACCCGGGTAGTCCTGGCTGTAAAGGATGCGGGCTAGGTGTCGGGCGAGCTTCGAGCTC 757

|||| |||||||||||||| ||||| |||| || || ||||| ||| ||||||||||

Sbjct 1719519 ATACTCGGGTAGTCCTGGCCGTAAACGATGTGGACTTGGTGTTGGGATGGCTTCGAGCTG 1719578

Query 758 GCCCGGTGCCGAAGGGAAGCCGTTAAGCCCGCCGCCTGGGGAGTACGGCCGCAAGGCTGA 817

||| ||||||||||||||| |||||| || ||||||||| |||||||||||||||||||

Sbjct 1719579 CCCCAGTGCCGAAGGGAAGCTGTTAAGTCCACCGCCTGGGAAGTACGGCCGCAAGGCTGA 1719638

Query 818 AACTTAAAGGAATTGGCGGGGGAGCACTACAAGGGGTGGAGCGTGCGGTTTAATTGGATT 877

||||||||||||||||||||||||||| |||| | ||||||| |||||||||||||||||

Sbjct 1719639 AACTTAAAGGAATTGGCGGGGGAGCACCACAACGCGTGGAGCCTGCGGTTTAATTGGATT 1719698

Query 878 CAACGCCGGGAACCTCACCGGGGGCGACGGCAGGATGAAGGCCAGGCTGAAGGTCTTGCC 937

||||||||| | |||||| |||||||| |||| |||| ||||||| ||| | ||||||

Sbjct 1719699 CAACGCCGGACATCTCACCAGGGGCGACAGCAGTATGATGGCCAGGTTGATGACCTTGCC 1719758

Query 938 GGACACGCCGAGAGGAGGTGCATGGCCGCCGTCAGCTCGTACCGTGAGGCGTCCACTTAA 997

||| || ||||||||||||||||||||||||||||||||||||||||||||| ||||

Sbjct 1719759 TGACGAGCTGAGAGGAGGTGCATGGCCGCCGTCAGCTCGTACCGTGAGGCGTCCTGTTAA 1719818

Query 998 GTGTGGTAACGAGCGAGACCCGCGCCCCCCAGTTGCCAGTCCTTCCCGC-TGGGGAGGA- 1055

|| || |||||||||||||| ||||| |||| |||| ||| |||| |

Sbjct 1719819 GTCAGGCAACGAGCGAGACCCACGCCCTT-AGTTACCAGCGGAACCCTTATGGGTTGCCG 1719877

Query 1056 GGCACTCTGGGGGGACCGCCGGCGATAAGCCGGAGGAAGGAGCGGGCGACGGTAGGTCAG 1115

||||| || |||||||||| | ||||| | ||||||||||| || |||||||||||| |

Sbjct 1719878 GGCACACTAAGGGGACCGCCAGTGATAAACTGGAGGAAGGAGTGGACGACGGTAGGTCCG 1719937

Query 1116 TATGCCCCGAAACCCCCGGGCTACACGCGCGCTACAATGGGCGGGACAATGGGATCCGAC 1175

||||||||||| |||| |||| ||||||| |||||||||| | |||||||||| ||||||

Sbjct 1719938 TATGCCCCGAATCCCCTGGGCAACACGCGGGCTACAATGGCCTGGACAATGGGTTCCGAC 1719997

Query 1176 CCCGAAAGGGGAAGGGAATCCCCTAAACCCGCCCCCAGTTCGGATCGCGGGCTGCAACTC 1235

|||||||| | ||| ||||||||||||| | | ||||||||||| |||||| ||| |

Sbjct 1719998 ACCGAAAGGTGGAGGTAATCCCCTAAACCAGGTCGTAGTTCGGATCGAGGGCTGTAACCC 1720057

Query 1236 GCCCGCGTGAAGCTGGAATCCCTAGTACCCGCGTGTCATCATCGCGCGGCGAATACGTCC 1295

|||| |||||||||||||| | ||||| ||||||||| ||||||||| ||||||||||

Sbjct 1720058 GCCCTCGTGAAGCTGGAATGCGTAGTAATCGCGTGTCACTATCGCGCGGTGAATACGTCC 1720117

Query 1296 CTGCTCCTTGCACACACCGCCCGTCA 1321

||||||||||||||||||||||||||

Sbjct 1720118 CTGCTCCTTGCACACACCGCCCGTCA 1720143

> gi|296241748|ref|NC_014160.1| Thermosphaera aggregans DSM 11486,

complete sequence

Length=1316595

Score = 1153 bits (624), Expect = 0.0

Identities = 1080/1300 (83%), Gaps = 32/1300 (2%)

Strand=Plus/Plus

Query 44 GGCGGACGGCTCAGTAACACGTCGGTAACCTACCCTCGGGAGGGGGATAACCCCGGGAAA 103

|||| |||||| |||| ||||| | |||||| ||||||||||||||||||| ||||||||

Sbjct 52907 GGCGCACGGCTGAGTAGCACGTGGCTAACCTGCCCTCGGGAGGGGGATAACACCGGGAAA 52966

Query 104 CTGGGGCTAATCCCCCATAGGCCTGAGG-TACTGGAA-GG----TCCTC----AGG-CC- 151

|||| |||||||||||||||| ||| |||||| || ||||| ||| ||

Sbjct 52967 CTGGTGCTAATCCCCCATAGG-GGAAGGCGCCTGGAAGGGTCCTTCCTCGAAAAGGCCCG 53025

Query 152 GAAAGGG---GCTCTG-C---CCGCCCGAGGATGGGCCGGCGGCCGATTAGGTAGTTGGT 204

| | ||| || ||| | ||||||||||||||| | ||||| || ||||||||||

Sbjct 53026 GCAGGGGTTAGCGCTGCCGAGCCGCCCGAGGATGGGGCTACGGCCCATCAGGTAGTTGGC 53085

Query 205 GGGGTAACGGCCCACCAAGCCGAAGATCGGTACGGGCCATGAGAGTGGGAGCCCGGAGAT 264

||||||||||||| ||||||||| | ||| ||||| |||||| |||||||| ||||

Sbjct 53086 GGGGTAACGGCCCGCCAAGCCGATAACGGGTGGGGGCCGTGAGAGCGGGAGCCCCCAGAT 53145

Query 265 GGACACTGAGACACGGGTCCAGGCCCTACGGGGCGCAGCAGGCGCGAAACCTCCGCAATG 324

|| |||||||||| ||| |||||||||| |||||||| ||||||||||||||||||||||

Sbjct 53146 GGGCACTGAGACAAGGGCCCAGGCCCTAAGGGGCGCACCAGGCGCGAAACCTCCGCAATG 53205

Query 325 CGGGCAACCGCGACGGGGGGACCCCCAGTGCCGTGGCAACGCCACGGCTTTTCCGGAGTG 384

|||| ||||| ||||||| ||||| |||||| || ||||||||| ||

Sbjct 53206 CGGGAAACCGTGACGGGGCCACCCCGAGTGCCCCCTTTCCG--GGGGCTTTTCCCCGCTG 53263

Query 385 TAAAAAGCTCCGGGAATAAGGGCTGGGCAAGGCCGGTGGCAGCCGCCGCGGTAATACCGG 444

|| ||| ||||||||| | ||||||| | |||| ||||||||||||||||||| |

Sbjct 53264 TAGGAAGGCGGGGGAATAAGCGGGGGGCAAGTCTGGTGTCAGCCGCCGCGGTAATACCAG 53323

Query 445 CGGCCCGAGTGGTGGCCGCTATTATTGGGCCTAAAGCGTCCGTAGCCGGGCCCGTAAGTC 504

| | |||||||| | | ||||||||||||||||| |||||||||| || | |||||

Sbjct 53324 CCCCGCGAGTGGTCGGGACGGTTATTGGGCCTAAAGCGCCCGTAGCCGGCCCGGCAAGTC 53383

Query 505 CCTGGCGAAATCCCACGGCTCAACCGTGGGGCTTGCTGGGGATACTGCGGGCCTTGGGAC 564

|| | ||||||| ||||||||| ||||| || ||||||||||| || || |||

Sbjct 53384 CCCTCCTAAATCCCCGGGCTCAACC-TGGGGACTGGGGGGGATACTGCCGGGCTAGGGGG 53442

Query 565 CGGGAGAGGCCGGGGGTACCCCTGGGGTAGGGGTGAAATCCTATAATCCCAGGGGGACCG 624

|||||||||||| |||||| || |||||||||| |||||||||||||||| || |||||

Sbjct 53443 CGGGAGAGGCCGAGGGTACTCCCGGGGTAGGGGCGAAATCCTATAATCCCGGGAGGACCA 53502

Query 625 CCAGTGGCGAAGGCGCCCGGCTGGAACGGGTCCGACGGTGAGGGACGAAGGCCAGGGGAG 684

|||||||||||||||| ||||||||||| | ||||||||||||| |||| ||| ||||||

Sbjct 53503 CCAGTGGCGAAGGCGCTCGGCTGGAACGCGCCCGACGGTGAGGGGCGAAAGCCGGGGGAG 53562

Query 685 CGAACCGGATTAGATACCCGGGTAGTCCTGGCTGTAAAGGATGCGGGCTAGGTGTCGGGC 744

| |||||||||||||||||||||||||| ||||||||| |||||||||||| ||| |||

Sbjct 53563 CAAACCGGATTAGATACCCGGGTAGTCCCGGCTGTAAACGATGCGGGCTAGCTGTTGGGT 53622

Query 745 GAGCTTCGAGCTCGCCCGGTGCCGAAGGGAAGCCGTTAAGCCCGCCGCCTGGGGAGTACG 804

| |||| |||| | ||| ||| || |||||||||||||||||||||||||||||||||||

Sbjct 53623 GGGCTTAGAGCCCACCCAGTGGCGCAGGGAAGCCGTTAAGCCCGCCGCCTGGGGAGTACG 53682

Query 805 GCCGCAAGGCTGAAACTTAAAGGAATTGGCGGGGGAGCACTACAAGGGGTGGAGCGTGCG 864

|||||||||||||||||||||||||||||||||||||||| |||||||||||||| ||||

Sbjct 53683 GCCGCAAGGCTGAAACTTAAAGGAATTGGCGGGGGAGCACCACAAGGGGTGGAGCCTGCG 53742

Query 865 GTTTAATTGGATTCAACGCCGGGAACCTCACCGGGGGCGACGGCAGGATGAAGGCCAGGC 924

||| ||||||| ||||||||||||| ||||||||||||||| ||||||||| ||||||||

Sbjct 53743 GTTCAATTGGAGTCAACGCCGGGAATCTCACCGGGGGCGACAGCAGGATGACGGCCAGGC 53802

Query 925 TGAAGGTCTTGCCGGACACGCCGAGAGGAGGTGCATGGCCGCCGTCAGCTCGTACCGTGA 984

| | | |||||| ||| ||| ||||||||||||||||||| || |||||||| ||||||

Sbjct 53803 TAACGACCTTGCCTGACGCGCTGAGAGGAGGTGCATGGCCGTCGCCAGCTCGTGCCGTGA 53862

Query 985 GGCGTCCACTTAAGTGTGGTAACGAGCGAGACCCGCGCCCCCCAGTTGCCAGTCCTTCCC 1044

|| |||| |||||| || |||||||||||||| |||||| |||||| | ||

Sbjct 53863 GGTGTCCGGTTAAGTCCGGAAACGAGCGAGACCCCTGCCCCC-AGTTGCGA--CC-CAGG 53918

Query 1045 GCTGGGGA--GGAGGCACTCTGGGGGGACCGCCGGCGAT-AAGCCGGAGGAAGGAGCGGG 1101

||| || | ||||| |||||||||| |||| || | ||| |||||||||||| |||

Sbjct 53919 GCTACGGCCCTGGGGCACACTGGGGGGACTGCCGCCGTTCAAGGCGGAGGAAGGAGGGGG 53978

Query 1102 CGACGGTAGGTCAGTATGCCCCGAAACCCCCGGGCTACACGCGCGCTACAATGGGCGGGA 1161

| |||| ||||||| |||||||||| ||||||||||||||||| |||||||||| ||||

Sbjct 53979 CCACGGCAGGTCAGCATGCCCCGAATCCCCCGGGCTACACGCGGGCTACAATGGCGGGGA 54038

Query 1162 CAATGGGATCCGACCCCGAAAGGGGAAGGGAATCCCCTAAACCCGCCCCCAGTTCGGATC 1221

|| ||| ||||||||||||||||| ||| |||||| |||||| || ||||| |||||

Sbjct 54039 CAGCGGGTTCCGACCCCGAAAGGGGGAGGCAATCCCTCAAACCCCGCCGCAGTTGGGATC 54098

Query 1222 GCGGGCTGCAACTCGCCCGCGTGAA-GCTGGAATCCCTAGTACCCGCGTGTCATCATCGC 1280

| |||||||||||||||| |||||| || ||||||||||||| ||||| |||| ||||||

Sbjct 54099 GAGGGCTGCAACTCGCCCTCGTGAACGC-GGAATCCCTAGTAACCGCGCGTCAACATCGC 54157

Query 1281 GCGGCGAATACGTCCCTGCTCCTTGCACACACCGCCCGTC 1320

|||| |||||||||||||||||||||||||||||||||||

Sbjct 54158 GCGGTGAATACGTCCCTGCTCCTTGCACACACCGCCCGTC 54197

> gi|150400439|ref|NC_009635.1| Methanococcus aeolicus Nankai-3,

complete sequence

Length=1569500

Score = 1088 bits (589), Expect = 0.0

Identities = 1082/1323 (82%), Gaps = 21/1323 (2%)

Strand=Plus/Minus

Query 4 CGACTAAGCCATGCGAGTCATGGGGCGCCTTGCGCGCACCGGCGGACGGCTCAGTAACAC 63

||||||||||||||||||| | | | ||| | || ||||||||||||||||||||

Sbjct 1196063 CGACTAAGCCATGCGAGTC-T-ATG-GACTTCGGTCCA-TGGCGGACGGCTCAGTAACAC 1196008

Query 64 GTCGGTAACCTACCCTCGGGAGGGGGATAACCCCGGGAAACTGGGGCTAATCCCCCATAG 123

|| | |||||||||||| || |||| |||||| |||||||||| || |||| ||||||||

Sbjct 1196007 GTGGCTAACCTACCCTCAGGTGGGGCATAACCTCGGGAAACTGAGGATAATACCCCATAG 1195948

Query 124 G-CCTGAGGTACTGGAA-GGTCCTCAGGCCGAAAGGGGCTCTGCCCGCCCGAGGATGGGC 181

| ||||| ||||| |||| | |||| || | | ||||| |||||||||

Sbjct 1195947 GAAAAGAGGT-TTGGAATAATCCT-TTTCTGAAA--GGATAT--CCGCCTGAGGATGGGG 1195894

Query 182 CGGCGGCCGATTAGGTAGTTGGTGGGGTAACGGCCCACCAAGCCGAAGATCGGTACGGGC 241

| ||| |||||||||||||||||||||||| ||||||||||||| | |||||||||||||

Sbjct 1195893 CTGCGTCCGATTAGGTAGTTGGTGGGGTAATGGCCCACCAAGCCTACGATCGGTACGGGC 1195834

Query 242 CATGAGAGTGGGAGCCCGGAGATGGACACTGAGACACGGGTCCAGGCCCTACGGGGCGCA 301

| |||||| |||||||||||||||| |||||||||||| |||||||||||||||||||

Sbjct 1195833 CTTGAGAGAGGGAGCCCGGAGATGGGGACTGAGACACGGCCCCAGGCCCTACGGGGCGCA 1195774

Query 302 GCAGGCGCGAAACCTCCGCAATGCGGGCAACCGCGACGGGGGGACCCCCAGTGCCGTGGC 361

||||||||||||||||| |||||| | || |||| ||||| | ||| |||||| ||

Sbjct 1195773 GCAGGCGCGAAACCTCCACAATGCACGAAAGTGCGATGGGGGAATCCCAAGTGCCTATGC 1195714

Query 362 AACGCCACGGCTTTTCCGGAGTGTAAAAAGCTCCGGGAATAAGGGCTGGGCAAGGCCGGT 421

| || ||||||||| ||| |||| | || |||||||||||||||||||| |||||

Sbjct 1195713 ACAGCATAGGCTTTTCCCAAGTCTAAACAACTTGGGGAATAAGGGCTGGGCAAGTCCGGT 1195654

Query 422 GGCAGCCGCCGCGGTAATACCGGCGGCCCGAGTGGTGGCCGCTATTATTGGGCCTAAAGC 481

| |||| |||||||||| |||||||||||||||||| ||| || ||||||||||||||||

Sbjct 1195653 GCCAGCAGCCGCGGTAACACCGGCGGCCCGAGTGGTAGCCACTCTTATTGGGCCTAAAGC 1195594

Query 482 GTCCGTAGCCGGGCCCGTAAGTCCCTGGCGAAATCCCACGGCTCAACCGTGGGGCTTGCT 541

|||||||||| | | ||||||| ||| |||||| |||||| |||||| ||||| ||

Sbjct 1195593 GTCCGTAGCCTGTTCAGTAAGTCTCTGTTTAAATCCTACGGCTTAACCGTAGGGCTGGC- 1195535

Query 542 GGGGATACTGCGGGCCTTGGGACCGGGAGAGGCCGGGGGTACCCCTGGGGTAGGGGTGAA 601

| |||||||| || ||||||||||||||||| | |||||| | ||||||| ||||||

Sbjct 1195534 AGAGATACTGCTGGACTTGGGACCGGGAGAGGAAGAGGGTACTTCGGGGGTAGCGGTGAA 1195475

Query 602 ATCCTATAATCCCAGGGGGACCGCCAGTGGCGAAGGCGCCCGGCTGGAACGGGTCCGACG 661

|| | | ||||| | |||||| || |||||||||| | | |||||||||||||||||

Sbjct 1195474 ATGCGTTGATCCCTGAGGGACCACCTATGGCGAAGGCACTCTTCTGGAACGGGTCCGACG 1195415

Query 662 GTGAGGGACGAAGGCCAGGGGAGCGAACCGGATTAGATACCCGGGTAGTCCTGGCTGTAA 721

|||||||||||| |||||||||||||||||||||||||||||||||||||||||| ||||

Sbjct 1195414 GTGAGGGACGAAAGCCAGGGGAGCGAACCGGATTAGATACCCGGGTAGTCCTGGCCGTAA 1195355

Query 722 AGGATGCGGGCTAGGTGTCGGGCGAGCTTCGAGCTCGCCC-GGTGCCGAAGGGAAGCCGT 780

| |||| ||||||||| | | ||| | || ||||||||||||||||| |

Sbjct 1195354 ACTTTGCGAACTAGGTGTCATCTGGACCTCGGG-TCCAGGTGGTGCCGAAGGGAAGCCAT 1195296

Query 781 TAAGCCCGCCGCCTGGGGAGTACGGCCGCAAGGCTGAAACTTAAAGGAATTGGCGGGGGA 840

|||| ||||||||||||||||||| |||||| |||||||||||||||||||||||||||

Sbjct 1195295 TAAGTTCGCCGCCTGGGGAGTACGGTCGCAAGACTGAAACTTAAAGGAATTGGCGGGGGA 1195236

Query 841 GCACTACAAGGGGTGGAGCGTGCGGTTTAATTGGATTCAACGCCGGGAACCTCACCGGGG 900

|||| |||| ||||||||| ||||||||||||||||||||||||||| | |||||| ||

Sbjct 1195235 GCACCACAACGGGTGGAGCCTGCGGTTTAATTGGATTCAACGCCGGGCATCTCACCAGGA 1195176

Query 901 GCGACGGCAGGATGAAGGCCAGGCTGAAGGTCTTGCCGGACACGCCGAGAGGAGGTGCAT 960

||||| ||| |||||||| |||| ||| | ||| || || ||| |||||| |||||||

Sbjct 1195175 GCGACAGCATGATGAAGGTCAGGTTGACGACCTTACCTGAAGCGCTGAGAGGTGGTGCAT 1195116

Query 961 GGCCGCCGTCAGCTCGTACCGTGAGGCGTCCACTTAAGTGTGGTAACGAGCGAGACCCGC 1020

|||| ||||||||||||||| ||||||||| |||||| ||||||||||||||||||

Sbjct 1195115 GGCCATCGTCAGCTCGTACCGCGAGGCGTCCTGTTAAGTCAGGTAACGAGCGAGACCCGT 1195056

Query 1021 GCCCCCCAGTTGCCAGTC-CTTCCCGCTGGGGAGGAGGCACTCTGGGGGGACCGCCGGCG 1079

|||| ||||| | | |||| | | | ||| |||||||| ||||||||| || |

Sbjct 1195055 GCCCTAT-GTTGCTACTTTCTTCTC-CGGAGGAA-AGGCACTCATAGGGGACCGCTGGTG 1194999

Query 1080 ATAAGCCGGAGGAAGGAGCGGGCGACGGTAGGTCAGTATGCCCCGAAACCCCCGGGCTAC 1139

||| || ||||||||||||||| ||| |||||| | |||||||||| | || |||||||

Sbjct 1194998 TTAAACCAGAGGAAGGAGCGGGCAACGATAGGTCCGCATGCCCCGAATCTCCTGGGCTAC 1194939

Query 1140 ACGCGCGCTACAATGGGCGGGACAATGGGATCCGACCCCGAAAGGGGAAGGGAATCCCCT 1199

||||| |||||||||| ||||||||||| | |||| || | |||||| |||| | |

Sbjct 1194938 ACGCGGGCTACAATGGTTAGGACAATGGGAAGCAACCCTGAGAAGGGAAGCAAATCTCTT 1194879

Query 1200 AAACCCGCCCCCAGTTCGGATCGCGGGCTGCAACTCGCCCGCGTGAAGCTGGAATCCCTA 1259

||||| | ||||||||||| |||||| ||||||||| |||||||||||||||| ||

Sbjct 1194878 AAACCTAATCGTAGTTCGGATCGTGGGCTGTAACTCGCCCACGTGAAGCTGGAATCCGTA 1194819

Query 1260 GTACCCGC-GTGTCATCATCGCGCGGCGAATACGTCCCTGCTCCTTGCACACACCGCCCG 1318

||| ||| || |||| || |||| |||| |||||||||||||||||||||||||||

Sbjct 1194818 GTAATCGCAGT-TCATAATACTGCGGTGAATGTGTCCCTGCTCCTTGCACACACCGCCCG 1194760

Query 1319 TCA 1321

|||

Sbjct 1194759 TCA 1194757

Score = 1088 bits (589), Expect = 0.0

Identities = 1082/1323 (82%), Gaps = 21/1323 (2%)

Strand=Plus/Plus

Query 4 CGACTAAGCCATGCGAGTCATGGGGCGCCTTGCGCGCACCGGCGGACGGCTCAGTAACAC 63

||||||||||||||||||| | | | ||| | || ||||||||||||||||||||

Sbjct 1230211 CGACTAAGCCATGCGAGTC-T-ATG-GACTTCGGTCCA-TGGCGGACGGCTCAGTAACAC 1230266

Query 64 GTCGGTAACCTACCCTCGGGAGGGGGATAACCCCGGGAAACTGGGGCTAATCCCCCATAG 123

|| | |||||||||||| || |||| |||||| |||||||||| || |||| ||||||||

Sbjct 1230267 GTGGCTAACCTACCCTCAGGTGGGGCATAACCTCGGGAAACTGAGGATAATACCCCATAG 1230326

Query 124 G-CCTGAGGTACTGGAA-GGTCCTCAGGCCGAAAGGGGCTCTGCCCGCCCGAGGATGGGC 181

| ||||| ||||| |||| | |||| || | | ||||| |||||||||

Sbjct 1230327 GAAAAGAGGT-TTGGAATAATCCT-TTTCTGAAA--GGATAT--CCGCCTGAGGATGGGG 1230380

Query 182 CGGCGGCCGATTAGGTAGTTGGTGGGGTAACGGCCCACCAAGCCGAAGATCGGTACGGGC 241

| ||| |||||||||||||||||||||||| ||||||||||||| | |||||||||||||

Sbjct 1230381 CTGCGTCCGATTAGGTAGTTGGTGGGGTAATGGCCCACCAAGCCTACGATCGGTACGGGC 1230440

Query 242 CATGAGAGTGGGAGCCCGGAGATGGACACTGAGACACGGGTCCAGGCCCTACGGGGCGCA 301

| |||||| |||||||||||||||| |||||||||||| |||||||||||||||||||

Sbjct 1230441 CTTGAGAGAGGGAGCCCGGAGATGGGGACTGAGACACGGCCCCAGGCCCTACGGGGCGCA 1230500

Query 302 GCAGGCGCGAAACCTCCGCAATGCGGGCAACCGCGACGGGGGGACCCCCAGTGCCGTGGC 361

||||||||||||||||| |||||| | || |||| ||||| | ||| |||||| ||

Sbjct 1230501 GCAGGCGCGAAACCTCCACAATGCACGAAAGTGCGATGGGGGAATCCCAAGTGCCTATGC 1230560

Query 362 AACGCCACGGCTTTTCCGGAGTGTAAAAAGCTCCGGGAATAAGGGCTGGGCAAGGCCGGT 421

| || ||||||||| ||| |||| | || |||||||||||||||||||| |||||

Sbjct 1230561 ACAGCATAGGCTTTTCCCAAGTCTAAACAACTTGGGGAATAAGGGCTGGGCAAGTCCGGT 1230620

Query 422 GGCAGCCGCCGCGGTAATACCGGCGGCCCGAGTGGTGGCCGCTATTATTGGGCCTAAAGC 481

| |||| |||||||||| |||||||||||||||||| ||| || ||||||||||||||||

Sbjct 1230621 GCCAGCAGCCGCGGTAACACCGGCGGCCCGAGTGGTAGCCACTCTTATTGGGCCTAAAGC 1230680

Query 482 GTCCGTAGCCGGGCCCGTAAGTCCCTGGCGAAATCCCACGGCTCAACCGTGGGGCTTGCT 541

|||||||||| | | ||||||| ||| |||||| |||||| |||||| ||||| ||

Sbjct 1230681 GTCCGTAGCCTGTTCAGTAAGTCTCTGTTTAAATCCTACGGCTTAACCGTAGGGCTGGC- 1230739

Query 542 GGGGATACTGCGGGCCTTGGGACCGGGAGAGGCCGGGGGTACCCCTGGGGTAGGGGTGAA 601

| |||||||| || ||||||||||||||||| | |||||| | ||||||| ||||||

Sbjct 1230740 AGAGATACTGCTGGACTTGGGACCGGGAGAGGAAGAGGGTACTTCGGGGGTAGCGGTGAA 1230799

Query 602 ATCCTATAATCCCAGGGGGACCGCCAGTGGCGAAGGCGCCCGGCTGGAACGGGTCCGACG 661

|| | | ||||| | |||||| || |||||||||| | | |||||||||||||||||

Sbjct 1230800 ATGCGTTGATCCCTGAGGGACCACCTATGGCGAAGGCACTCTTCTGGAACGGGTCCGACG 1230859

Query 662 GTGAGGGACGAAGGCCAGGGGAGCGAACCGGATTAGATACCCGGGTAGTCCTGGCTGTAA 721

|||||||||||| |||||||||||||||||||||||||||||||||||||||||| ||||

Sbjct 1230860 GTGAGGGACGAAAGCCAGGGGAGCGAACCGGATTAGATACCCGGGTAGTCCTGGCCGTAA 1230919

Query 722 AGGATGCGGGCTAGGTGTCGGGCGAGCTTCGAGCTCGCCC-GGTGCCGAAGGGAAGCCGT 780

| |||| ||||||||| | | ||| | || ||||||||||||||||| |

Sbjct 1230920 ACTTTGCGAACTAGGTGTCATCTGGACCTCGGG-TCCAGGTGGTGCCGAAGGGAAGCCAT 1230978

Query 781 TAAGCCCGCCGCCTGGGGAGTACGGCCGCAAGGCTGAAACTTAAAGGAATTGGCGGGGGA 840

|||| ||||||||||||||||||| |||||| |||||||||||||||||||||||||||

Sbjct 1230979 TAAGTTCGCCGCCTGGGGAGTACGGTCGCAAGACTGAAACTTAAAGGAATTGGCGGGGGA 1231038

Query 841 GCACTACAAGGGGTGGAGCGTGCGGTTTAATTGGATTCAACGCCGGGAACCTCACCGGGG 900

|||| |||| ||||||||| ||||||||||||||||||||||||||| | |||||| ||

Sbjct 1231039 GCACCACAACGGGTGGAGCCTGCGGTTTAATTGGATTCAACGCCGGGCATCTCACCAGGA 1231098

Query 901 GCGACGGCAGGATGAAGGCCAGGCTGAAGGTCTTGCCGGACACGCCGAGAGGAGGTGCAT 960

||||| ||| |||||||| |||| ||| | ||| || || ||| |||||| |||||||

Sbjct 1231099 GCGACAGCATGATGAAGGTCAGGTTGACGACCTTACCCGAAGCGCTGAGAGGTGGTGCAT 1231158

Query 961 GGCCGCCGTCAGCTCGTACCGTGAGGCGTCCACTTAAGTGTGGTAACGAGCGAGACCCGC 1020

|||| ||||||||||||||| ||||||||| |||||| ||||||||||||||||||

Sbjct 1231159 GGCCATCGTCAGCTCGTACCGCGAGGCGTCCTGTTAAGTCAGGTAACGAGCGAGACCCGT 1231218

Query 1021 GCCCCCCAGTTGCCAGTC-CTTCCCGCTGGGGAGGAGGCACTCTGGGGGGACCGCCGGCG 1079

|||| ||||| | | |||| | | | ||| |||||||| ||||||||| || |

Sbjct 1231219 GCCCTAT-GTTGCTACTTTCTTCTC-CGGAGGAA-AGGCACTCATAGGGGACCGCTGGTG 1231275

Query 1080 ATAAGCCGGAGGAAGGAGCGGGCGACGGTAGGTCAGTATGCCCCGAAACCCCCGGGCTAC 1139

||| || ||||||||||||||| ||| |||||| | |||||||||| | || |||||||

Sbjct 1231276 TTAAACCAGAGGAAGGAGCGGGCAACGATAGGTCCGCATGCCCCGAATCTCCTGGGCTAC 1231335

Query 1140 ACGCGCGCTACAATGGGCGGGACAATGGGATCCGACCCCGAAAGGGGAAGGGAATCCCCT 1199

||||| |||||||||| ||||||||||| | |||| || | |||||| |||| | |

Sbjct 1231336 ACGCGGGCTACAATGGTTAGGACAATGGGAAGCAACCCTGAGAAGGGAAGCAAATCTCTT 1231395

Query 1200 AAACCCGCCCCCAGTTCGGATCGCGGGCTGCAACTCGCCCGCGTGAAGCTGGAATCCCTA 1259

||||| | ||||||||||| |||||| ||||||||| |||||||||||||||| ||

Sbjct 1231396 AAACCTAATCGTAGTTCGGATCGTGGGCTGTAACTCGCCCACGTGAAGCTGGAATCCGTA 1231455

Query 1260 GTACCCGC-GTGTCATCATCGCGCGGCGAATACGTCCCTGCTCCTTGCACACACCGCCCG 1318

||| ||| || |||| || |||| |||| |||||||||||||||||||||||||||

Sbjct 1231456 GTAATCGCAGT-TCATAATACTGCGGTGAATGTGTCCCTGCTCCTTGCACACACCGCCCG 1231514

Query 1319 TCA 1321

|||

Sbjct 1231515 TCA 1231517

> gi|116753325|ref|NC_008553.1| Methanothrix thermoacetophila PT,

complete sequence

Length=1879471

Score = 972 bits (526), Expect = 0.0

Identities = 1037/1287 (81%), Gaps = 22/1287 (2%)

Strand=Plus/Plus

Query 45 GCGGACGGCTCAGTAACACGTCGGTAACCTACCCTCGGGAGGGGGATAACCCCGGGAAAC 104

||| || |||||||||||||| | ||||| |||| ||| || |||| ||||||||||

Sbjct 845603 GCGCACTGCTCAGTAACACGTGGACAACCTGCCCTGAGGACTGGAATAATCCCGGGAAAC 845662

Query 105 TGGGGCTAATCCCCCATAGGCCTGAGGTACTGGAAGGTCCTCAGGCCGAAAGGGGCTCTG 164

||||| |||| || ||| | | || | |||||| | || ||| |||| ||||| |

Sbjct 845663 TGGGGGTAATTCCAGATATGTCAGAACTCCTGGAATGGGTTCTGGCAGAAA--GGCTCCG 845720

Query 165 CCCGCCCGAGGATGGGCCGGCGGCCGATTAGG-TAGTTGGTGGG-GTAACGGCCCACCAA 222

|||| |||||||| | |||||| || ||| ||| | ||||| |||||| || | |

Sbjct 845721 -GCGCCTCAGGATGGGTCTGCGGCCTATCAGGGTAG-TAGTGGGTGTAACGTACCTACTA 845778

Query 223 GCCGAAGATCGGTACGGGCCATGAGAGTGGGAGCCCGGAGATGGACACTGAGACACGGGT 282

||| | || |||||||| |||||| ||||||||||||||| |||||||| | |

Sbjct 845779 GCCTACGACGGGTACGGGTTGTGAGAGCAAGAGCCCGGAGATGGATTCTGAGACATGAAT 845838

Query 283 CCAGGCCCTACGGGGCGCAGCAGGCGCGAAACCTCCGCAATGCGGGCAACCGCGACGGGG 342

||||||||||||||| ||||||||||||||| || |||||||||||||||||| ||

Sbjct 845839 CCAGGCCCTACGGGGTGCAGCAGGCGCGAAAACTTTACAATGCGGGCAACCGCGATAAGG 845898

Query 343 GGACCCCCAGTGC--CGTGGCAACGCCACGGCTTTTCCGGAGTG--TAAAAAGC-TCCGG 397

||||| | ||||| || ||| || |||| || || || |||||||| |

Sbjct 845899 GGACCTCGAGTGCTGGGTTACAA-ACC-TGGCTGTT--GGGCTGCCTAAAAAGCAGTCTA 845954

Query 398 GAATAAGGGCTGGGCAAGGCCGGTGGCAGCCGCCGCGGTAATACCGGCGGCCCGAGTGGT 457

| |||||| ||||||| |||||| ||||||||||||||| ||||||||| ||||||||

Sbjct 845955 TAGCAAGGGCCGGGCAAGACCGGTGCCAGCCGCCGCGGTAACACCGGCGGCTCGAGTGGT 846014

Query 458 GGCCGCTATTATTGGGCCTAAAGCGTCCGTAGCCGGGCCCGTAAGTCCCTGGCGAAATCC 517

|||||||||||||| |||||| ||| |||||||| | |||||| || | ||||||

Sbjct 846015 AACCGCTATTATTGGGTCTAAAGGGTCTGTAGCCGGCCGACTAAGTCTCTTGGGAAATCT 846074

Query 518 CACGGCTCAACCGTGGGGCTTGCTGGGGATACT-GCGGGCCTTGGGACCGGGAGAGGCCG 576

| ||||| || |||| | ||||||||| | || |||||||||||||||||

Sbjct 846075 GGCATCTCAAGTGTCAGGCTGCCAGGGGATACTGGTCGG-CTTGGGACCGGGAGAGGTGA 846133

Query 577 GGGGTACCCCTGGGGTAGGGGTGAAATCCTATAATCCCAGGGGGACCGCCAGTGGCGAAG 636

| |||||| | ||||||||||||||||| | |||||| | |||||| ||||||||||||

Sbjct 846134 GAGGTACCTCGGGGGTAGGGGTGAAATCTTGTAATCCTCGAGGGACCACCAGTGGCGAAG 846193

Query 637 GCGCCCGGCTGGAACGGGTCCGACGGTGAGGGACGAAGGCCAGGGGAGCGAACCGGATTA 696

||| | | |||||| |||||||| ||||||||| || ||||| ||||||||||||

Sbjct 846194 GCGTCTCACCAGAACGGATCCGACGGCAAGGGACGAAAGCTAGGGGCACGAACCGGATTA 846253

Query 697 GATACCCGGGTAGTCCTGGCTGTAAAGGATGCGGGCTAGGTGTCGGGCGAGCTTCGAGCT 756

||||||||||||||||| || ||||| ||| | |||||||||||| | | | ||| |

Sbjct 846254 GATACCCGGGTAGTCCTAGCCGTAAACGATACTCGCTAGGTGTCGGCCACGGTGCGACCG 846313

Query 757 CGCCCGGTGCCGAAGGGAAGCCGTTAAGCCCGCCGCCTGGGGAGTACGGCCGCAAGGCTG 816

| |||||||| ||||||||||| |||| ||| |||||| ||||||||||||||||||

Sbjct 846314 TGGTCGGTGCCGTAGGGAAGCCGTGAAGCGAGCCACCTGGGAAGTACGGCCGCAAGGCTG 846373

Query 817 AAACTTAAAGGAATTGGCGGGGGAGCACTACAAGGGGTGGAGCGTGCGGTTTAATTGGAT 876

|||||||||||||||||||||||||||| |||| ||||||||| ||||||||||||||||

Sbjct 846374 AAACTTAAAGGAATTGGCGGGGGAGCACCACAACGGGTGGAGCCTGCGGTTTAATTGGAT 846433

Query 877 TCAACGCCGGGAACCTCACCGGGGGCGACGGCAGGATGAAGGCCAGGCTGAAGGTCTTGC 936

|||||||||| || || |||||||||||| ||| ||||||| |||||||||| ||| |

Sbjct 846434 TCAACGCCGGAAAGCTTACCGGGGGCGACAGCAATATGAAGGTCAGGCTGAAGACCTTAC 846493

Query 937 CGGACACGCCGAGAGGAGGTGCATGGCCGCCGTCAGCTCGTACCGTGAGGCGTCCACTTA 996

|||| ||| |||||| |||||||||||| |||||| |||||| |||| || ||| |||

Sbjct 846494 CGGATTCGCTGAGAGGTGGTGCATGGCCGTCGTCAGTTCGTACTGTGAAGCATCCTGTTA 846553

Query 997 AGTGTGGTAACGAGCGAGACCCGCGCCCCCCAGTTGCCAGTCCTTCCCGCTGGGGAGGAG 1056

||| || |||||||||||||| ||||| | ||||||||| | |||| | ||||||| |

Sbjct 846554 AGTCAGGCAACGAGCGAGACCCACGCCCAC-AGTTGCCAG-CGATCCCTCCGGGGAGGCG 846611

Query 1057 GC-ACTCTGGGGGGACCGCCGGCGATAAGCCGGAGGAAGGAGCGGGCGACGGTAGGTCAG 1115

| |||||| ||||||||||| | ||| |||||||||||| |||| ||||||||||||

Sbjct 846612 GGTACTCTGTGGGGACCGCCGCTGCTAAAGCGGAGGAAGGAGTGGGCAACGGTAGGTCAG 846671

Query 1116 TATGCCCCGAAACCCCCGGGCTACACGCGCGCTACAATGGGCGGGACAATGGG-ATCCGA 1174

||||||||||| ||||||||||||||||| |||||||||| ||| |||||||| || |||

Sbjct 846672 TATGCCCCGAATCCCCCGGGCTACACGCGGGCTACAATGGTCGGTACAATGGGTAT-CGA 846730

Query 1175 CCCCGAAAGGGGAAGGGAATCCCCTAAACCCGCCCCCAGTTCGGATCGCGGGCTGCAACT 1234

|||||||||||| ||| ||||||||||| ||| | ||||||||| | |||||| ||||

Sbjct 846731 CCCCGAAAGGGGTAGGCAATCCCCTAAAACCGATCGTAGTTCGGATTGAGGGCTGAAACT 846790

Query 1235 CGCCCGCGTGAAGCTGGAATCCCTAGTACCCGCGTGTCATCATCGCGCGGCGAATACGTC 1294

||||| | |||||||||||||| ||||| ||||| ||| || ||||| |||||||||

Sbjct 846791 CGCCCTCATGAAGCTGGAATCCGTAGTAATCGCGTTTCAACAGAACGCGGTGAATACGTC 846850

Query 1295 CCTGCTCCTTGCACACACCGCCCGTCA 1321

|||||||||||||||||||||||||||

Sbjct 846851 CCTGCTCCTTGCACACACCGCCCGTCA 846877

Score = 972 bits (526), Expect = 0.0

Identities = 1037/1287 (81%), Gaps = 22/1287 (2%)

Strand=Plus/Minus

Query 45 GCGGACGGCTCAGTAACACGTCGGTAACCTACCCTCGGGAGGGGGATAACCCCGGGAAAC 104

||| || |||||||||||||| | ||||| |||| ||| || |||| ||||||||||

Sbjct 1618589 GCGCACTGCTCAGTAACACGTGGACAACCTGCCCTGAGGACTGGAATAATCCCGGGAAAC 1618530

Query 105 TGGGGCTAATCCCCCATAGGCCTGAGGTACTGGAAGGTCCTCAGGCCGAAAGGGGCTCTG 164

||||| |||| || ||| | | || | |||||| | || ||| |||| ||||| |

Sbjct 1618529 TGGGGGTAATTCCAGATATGTCAGAACTCCTGGAATGGGTTCTGGCAGAAA--GGCTCCG 1618472

Query 165 CCCGCCCGAGGATGGGCCGGCGGCCGATTAGG-TAGTTGGTGGG-GTAACGGCCCACCAA 222

|||| |||||||| | |||||| || ||| ||| | ||||| |||||| || | |

Sbjct 1618471 -GCGCCTCAGGATGGGTCTGCGGCCTATCAGGGTAG-TAGTGGGTGTAACGTACCTACTA 1618414

Query 223 GCCGAAGATCGGTACGGGCCATGAGAGTGGGAGCCCGGAGATGGACACTGAGACACGGGT 282

||| | || |||||||| |||||| ||||||||||||||| |||||||| | |

Sbjct 1618413 GCCTACGACGGGTACGGGTTGTGAGAGCAAGAGCCCGGAGATGGATTCTGAGACATGAAT 1618354

Query 283 CCAGGCCCTACGGGGCGCAGCAGGCGCGAAACCTCCGCAATGCGGGCAACCGCGACGGGG 342

||||||||||||||| ||||||||||||||| || |||||||||||||||||| ||

Sbjct 1618353 CCAGGCCCTACGGGGTGCAGCAGGCGCGAAAACTTTACAATGCGGGCAACCGCGATAAGG 1618294

Query 343 GGACCCCCAGTGC--CGTGGCAACGCCACGGCTTTTCCGGAGTG--TAAAAAGC-TCCGG 397

||||| | ||||| || ||| || |||| || || || |||||||| |

Sbjct 1618293 GGACCTCGAGTGCTGGGTTACAA-ACC-TGGCTGTT--GGGCTGCCTAAAAAGCAGTCTA 1618238

Query 398 GAATAAGGGCTGGGCAAGGCCGGTGGCAGCCGCCGCGGTAATACCGGCGGCCCGAGTGGT 457

| |||||| ||||||| |||||| ||||||||||||||| ||||||||| ||||||||

Sbjct 1618237 TAGCAAGGGCCGGGCAAGACCGGTGCCAGCCGCCGCGGTAACACCGGCGGCTCGAGTGGT 1618178

Query 458 GGCCGCTATTATTGGGCCTAAAGCGTCCGTAGCCGGGCCCGTAAGTCCCTGGCGAAATCC 517

|||||||||||||| |||||| ||| |||||||| | |||||| || | ||||||

Sbjct 1618177 AACCGCTATTATTGGGTCTAAAGGGTCTGTAGCCGGCCGACTAAGTCTCTTGGGAAATCT 1618118

Query 518 CACGGCTCAACCGTGGGGCTTGCTGGGGATACT-GCGGGCCTTGGGACCGGGAGAGGCCG 576

| ||||| || |||| | ||||||||| | || |||||||||||||||||

Sbjct 1618117 GGCATCTCAAGTGTCAGGCTGCCAGGGGATACTGGTCGG-CTTGGGACCGGGAGAGGTGA 1618059

Query 577 GGGGTACCCCTGGGGTAGGGGTGAAATCCTATAATCCCAGGGGGACCGCCAGTGGCGAAG 636

| |||||| | ||||||||||||||||| | |||||| | |||||| ||||||||||||

Sbjct 1618058 GAGGTACCTCGGGGGTAGGGGTGAAATCTTGTAATCCTCGAGGGACCACCAGTGGCGAAG 1617999

Query 637 GCGCCCGGCTGGAACGGGTCCGACGGTGAGGGACGAAGGCCAGGGGAGCGAACCGGATTA 696

||| | | |||||| |||||||| ||||||||| || ||||| ||||||||||||

Sbjct 1617998 GCGTCTCACCAGAACGGATCCGACGGCAAGGGACGAAAGCTAGGGGCACGAACCGGATTA 1617939

Query 697 GATACCCGGGTAGTCCTGGCTGTAAAGGATGCGGGCTAGGTGTCGGGCGAGCTTCGAGCT 756

||||||||||||||||| || ||||| ||| | |||||||||||| | | | ||| |

Sbjct 1617938 GATACCCGGGTAGTCCTAGCCGTAAACGATACTCGCTAGGTGTCGGCCACGGTGCGACCG 1617879

Query 757 CGCCCGGTGCCGAAGGGAAGCCGTTAAGCCCGCCGCCTGGGGAGTACGGCCGCAAGGCTG 816

| |||||||| ||||||||||| |||| ||| |||||| ||||||||||||||||||

Sbjct 1617878 TGGTCGGTGCCGTAGGGAAGCCGTGAAGCGAGCCACCTGGGAAGTACGGCCGCAAGGCTG 1617819

Query 817 AAACTTAAAGGAATTGGCGGGGGAGCACTACAAGGGGTGGAGCGTGCGGTTTAATTGGAT 876

|||||||||||||||||||||||||||| |||| ||||||||| ||||||||||||||||

Sbjct 1617818 AAACTTAAAGGAATTGGCGGGGGAGCACCACAACGGGTGGAGCCTGCGGTTTAATTGGAT 1617759

Query 877 TCAACGCCGGGAACCTCACCGGGGGCGACGGCAGGATGAAGGCCAGGCTGAAGGTCTTGC 936

|||||||||| || || |||||||||||| ||| ||||||| |||||||||| ||| |

Sbjct 1617758 TCAACGCCGGAAAGCTTACCGGGGGCGACAGCAATATGAAGGTCAGGCTGAAGACCTTAC 1617699

Query 937 CGGACACGCCGAGAGGAGGTGCATGGCCGCCGTCAGCTCGTACCGTGAGGCGTCCACTTA 996

|||| ||| |||||| |||||||||||| |||||| |||||| |||| || ||| |||

Sbjct 1617698 CGGATTCGCTGAGAGGTGGTGCATGGCCGTCGTCAGTTCGTACTGTGAAGCATCCTGTTA 1617639

Query 997 AGTGTGGTAACGAGCGAGACCCGCGCCCCCCAGTTGCCAGTCCTTCCCGCTGGGGAGGAG 1056

||| || |||||||||||||| ||||| | ||||||||| | |||| | ||||||| |

Sbjct 1617638 AGTCAGGCAACGAGCGAGACCCACGCCCAC-AGTTGCCAG-CGATCCCTCCGGGGAGGCG 1617581

Query 1057 GC-ACTCTGGGGGGACCGCCGGCGATAAGCCGGAGGAAGGAGCGGGCGACGGTAGGTCAG 1115

| |||||| ||||||||||| | ||| |||||||||||| |||| ||||||||||||

Sbjct 1617580 GGTACTCTGTGGGGACCGCCGCTGCTAAAGCGGAGGAAGGAGTGGGCAACGGTAGGTCAG 1617521

Query 1116 TATGCCCCGAAACCCCCGGGCTACACGCGCGCTACAATGGGCGGGACAATGGG-ATCCGA 1174

||||||||||| ||||||||||||||||| |||||||||| ||| |||||||| || |||

Sbjct 1617520 TATGCCCCGAATCCCCCGGGCTACACGCGGGCTACAATGGTCGGTACAATGGGTAT-CGA 1617462

Query 1175 CCCCGAAAGGGGAAGGGAATCCCCTAAACCCGCCCCCAGTTCGGATCGCGGGCTGCAACT 1234

|||||||||||| ||| ||||||||||| ||| | ||||||||| | |||||| ||||

Sbjct 1617461 CCCCGAAAGGGGTAGGCAATCCCCTAAAACCGATCGTAGTTCGGATTGAGGGCTGAAACT 1617402

Query 1235 CGCCCGCGTGAAGCTGGAATCCCTAGTACCCGCGTGTCATCATCGCGCGGCGAATACGTC 1294

||||| | |||||||||||||| ||||| ||||| ||| || ||||| |||||||||

Sbjct 1617401 CGCCCTCATGAAGCTGGAATCCGTAGTAATCGCGTTTCAACAGAACGCGGTGAATACGTC 1617342

Query 1295 CCTGCTCCTTGCACACACCGCCCGTCA 1321

|||||||||||||||||||||||||||

Sbjct 1617341 CCTGCTCCTTGCACACACCGCCCGTCA 1617315

> gi|386000717|ref|NC_017527.1| Methanosaeta harundinacea 6Ac,

complete sequence

Length=2559043

Score = 950 bits (514), Expect = 0.0

Identities = 1062/1329 (80%), Gaps = 27/1329 (2%)

Strand=Plus/Plus

Query 4 CGACTAAGCCATGCGAGTCATGGGGCGCCTTGCGCGCAC-CGGCGGACGGCTCAGTAACA 62

|||||||||||||||||| || ||| || | || ||||||| |||||||||||

Sbjct 797361 CGACTAAGCCATGCGAGT--TGAATGTTCTT-CGTGAACATGGCGGACTGCTCAGTAACA 797417

Query 63 CGTCGGTAACCTACCCTCGGGAGGGGGATAACCCCGGGAAACTGGGGCTAATCCCCCATA 122

||| | |||||||||| ||| || |||| || |||||||||||| |||| || |||

Sbjct 797418 CGTGGACAACCTACCCTTGGGTCTGGCATAATCCTGGGAAACTGGGGATAATTCCGGATA 797477

Query 123 GGCCTGAGGTACTGGAAGGTCCTCAGGCCGAAAGGGGCTCTGCCCGCCCGAGGATGGGCC 182

|||| || | |||||| | || ||||||||| ||| | ||||| |||||||| |

Sbjct 797478 GGCCACAGATGCTGGAATGCGCTGTGGCCGAAAG---CTCCG-GCGCCCAAGGATGGGTC 797533

Query 183 GGCGGCCGATTAGG-TAGTTGGTGGG-GTAACGGCCCACCAAGCCGAAGATCGGTACGGG 240

|||||| || ||| | | | ||||| |||| | || | |||| | || ||||||||

Sbjct 797534 TGCGGCCTATCAGGGTTG-TAGTGGGTGTAATGTACCTACTAGCCTACGACGGGTACGGG 797592

Query 241 CCATGAGAGTGGGAGCCCGGAGATGGACACTGAGACACGGGTCCAGGCCCTACGGGGCGC 300

|||||| ||||||||||||||| |||||||||| |||||||||||||||| ||

Sbjct 797593 TTGTGAGAGCAAGAGCCCGGAGATGGATTCTGAGACACGAATCCAGGCCCTACGGGGTGC 797652

Query 301 AGCAGGCGCGAAACCTCCGCAATGCGGGCAACCGCGACGGGGGGACCCCCAGTGCCGTGG 360

||||||||||||| || ||||||||| ||||| || ||| | | | |||||| |

Sbjct 797653 AGCAGGCGCGAAAACTTTACAATGCGGGAAACCGTGATAAGGGAATCTCGAGTGCC--AG 797710

Query 361 CA-A-CGCCACGGCTTTTCCGGAGTG--TAAAAAGC-TCCGGGAATAAGGGCTGGGCAAG 415

|| | ||| | ||| || || |||||||| || | | |||||| |||||||

Sbjct 797711 CATATAATGTTGGC-TGTCCAGA-TGCCTAAAAAGCATCTGTTAGCAAGGGCCGGGCAAG 797768

Query 416 GCCGGTGGCAGCCGCCGCGGTAATACCGGCGGCCCGAGTGGTGGCCGCTATTATTGGGCC 475

|||||| ||||||||||||||| |||||||||||||||||| ||||| |||||||| |

Sbjct 797769 ACCGGTGCCAGCCGCCGCGGTAACACCGGCGGCCCGAGTGGTAACCGCTTTTATTGGGTC 797828

Query 476 TAAAGCGTCCGTAGCCGGGCCCGTAAGTCCCTGGCGAAATCCCACGGCTCAACCGTGGGG 535

||||| ||| |||||||| | |||||||||| | |||||| | ||| ||| || ||

Sbjct 797829 TAAAGGGTCTGTAGCCGGCCAAGTAAGTCCCTTGGGAAATCTGGCAGCTTAACTGTCAGG 797888

Query 536 CTTGCT-GGGGATACTGCGGGCCTTGGGACCGGGAGAGGCCGGGGGTACCCCTGGGGTAG 594

|| ||| ||||||||||| || ||||||||||||||||| | |||||| |||||||

Sbjct 797889 CT-GCTAGGGGATACTGCTGGGCTTGGGACCGGGAGAGGTGAGAGGTACCTTGGGGGTAG 797947

Query 595 GGGTGAAATCCTATAATCCCAGGGGGACCGCCAGTGGCGAAGGCGCCCGGCTGGAACGGG 654

|||||||||| | | |||| |||||||| ||||||||||||||| | | |||||||

Sbjct 797948 GGGTGAAATCTTGTGATCCTCGGGGGACCACCAGTGGCGAAGGCGTCTCACCAGAACGGG 798007

Query 655 TCCGACGGTGAGGGACGAAGGCCAGGGGAGCGAACCGGATTAGATACCCGGGTAGTCCTG 714

||||||||| ||||||||| || ||||| |||||||||||||||||||||||||||||

Sbjct 798008 TCCGACGGTAAGGGACGAAAGCTAGGGGCACGAACCGGATTAGATACCCGGGTAGTCCTA 798067

Query 715 GCTGTAAAGGATGCGGGCTAGGTGTCGGGCGAGCTTCGAGCTCGCCCGGTGCCGAAGGGA 774

|| ||||| ||||| |||||||||| | | | | ||| | | ||||||| |||||

Sbjct 798068 GCCGTAAACGATGCTCGCTAGGTGTCAGTCACGGTGCGACCGTGATTGGTGCCGTAGGGA 798127

Query 775 AGCCGTTAAGCCCGCCGCCTGGGGAGTACGGCCGCAAGGCTGAAACTTAAAGGAATTGGC 834

|||||| |||| ||| |||||| |||| |||||||||||||||||||||||||||||||

Sbjct 798128 AGCCGTGAAGCGAGCCACCTGGGAAGTATGGCCGCAAGGCTGAAACTTAAAGGAATTGGC 798187

Query 835 GGGGGAGCACTACAAGGGGTGGAGCGTGCGGTTTAATTGGATTCAACGCCGGGAACCTCA 894

||||||||||||||| ||||||||| |||||||||||||||||||||||||| || ||||

Sbjct 798188 GGGGGAGCACTACAACGGGTGGAGCCTGCGGTTTAATTGGATTCAACGCCGGAAATCTCA 798247

Query 895 CCGGGGGCGACGGCAGGATGAAGGCCAGGCTGAAGGTCTTGCCGGACACGCCGAGAGGAG 954

||||||||||| ||| ||||||| |||||||||| ||| || || || |||||| |

Sbjct 798248 CCGGGGGCGACAGCAATATGAAGGTCAGGCTGAAGACCTTACCCGATTAGCTGAGAGGTG 798307

Query 955 GTGCATGGCCGCCGTCAGCTCGTACCGTGAGGCGTCCACTTAAGTGTGGTAACGAGCGAG 1014

||||||||||| |||||| |||||| |||| || ||| |||||| || ||||||||||

Sbjct 798308 GTGCATGGCCGTCGTCAGTTCGTACTGTGAAGCATCCTGTTAAGTCAGGCAACGAGCGAG 798367

Query 1015 ACCCGCGCCCCCCAGTTGCCAGTCCTTCCCGCTGGGGAGGAGGC-ACTCTGGGGGGACCG 1073

|||| || || | ||||||||| | | ||| | |||| | || || ||| || |||||

Sbjct 798368 ACCCACGTCCAC-AGTTGCCAG-CATACCCTCCGGGGTGATGGGTACACTGTGGAGACCG 798425

Query 1074 CCGGCGATAAGCCGGAGGAAGGAGCGGGCGACGGTAGGTCAGTATGCCCCGAAACCCCCG 1133

||| | ||| |||||||||||| |||| ||||||||||||||||||||||| | ||||

Sbjct 798426 CCGCTGCTAAAGCGGAGGAAGGAGTGGGCAACGGTAGGTCAGTATGCCCCGAATCTCCCG 798485

Query 1134 GGCTACACGCGCGCTACAATGGGCGGGACAATGGG-ATCCGACCCCGAAAGGGGAAGGGA 1192

||||||||||| |||||||||| || |||||||| ||| ||||||||||||||||| |

Sbjct 798486 GGCTACACGCGGGCTACAATGGTTGGTACAATGGGCATCT-ACCCCGAAAGGGGAAGGAA 798544

Query 1193 ATCCCCTAAACCCGCCCCCAGTTCGGATCGCGGGCTGCAACTCGCCCGCGTGAAGCTGGA 1252

||| | |||| || | ||||||||| | |||||||||||||||| | ||||||||||

Sbjct 798545 ATCTCTTAAAGCCAATCGTAGTTCGGATTGAGGGCTGCAACTCGCCCTCATGAAGCTGGA 798604

Query 1253 ATCCCTAGTACCCGCGTGTCATCATCGCGCGGCGAATACGTCCCTGCTCCTTGCACACAC 1312

|||| ||||| ||||| ||| || ||||| |||||||||||||||||||||||||||

Sbjct 798605 ATCCGTAGTAATCGCGTTTCAACAGAACGCGGTGAATACGTCCCTGCTCCTTGCACACAC 798664

Query 1313 CGCCCGTCA 1321

|||||||||

Sbjct 798665 CGCCCGTCA 798673

> gi|851305307|ref|NZ_CP009501.1| Methanosarcina thermophila TM-1

chromosome, complete genome

Length=3127379

Score = 935 bits (506), Expect = 0.0

Identities = 1061/1332 (80%), Gaps = 25/1332 (2%)

Strand=Plus/Plus

Query 1 GTCCGACTAAGCCATGCGAGTCATGGGGCGCCTTGCGCGCAC-CGGCGGACGGCTCAGTA 59

|| || |||||||||||||||||| | ||| || | || |||| || ||||||||

Sbjct 104998 GTTCGCCTAAGCCATGCGAGTCAT-ATG-TTCTT-CGTGAACATGGCGTACTGCTCAGTA 105054

Query 60 ACACGTCGGTAACCTACCCTCGGGAGGGGGATAACCCCGGGAAACTGGGGCTAATCCCCC 119

|||||| | |||||| |||| |||| ||||||||||||||||||||||| |||| ||

Sbjct 105055 ACACGTGGATAACCTGCCCTTGGGACCGGGATAACCCCGGGAAACTGGGGATAATACCGG 105114

Query 120 ATA-GGCCTGAGGTACTGGAAGGTCCTCAGGCCGAAAGGGGCTCTGCCCGCCCGAGGATG 178

||| || | | | |||||| | | | | || ||| || | || | |||| ||||||

Sbjct 105115 ATAACGCAT-ATCTGCTGGAATG-CTTTATGCGTAAAATGGATTTGTCTGCCCAAGGATG 105172

Query 179 GGCCGGCGGCCGATTAGGTAGTTGGTGGG-GTAACGGCCCACCAAGCCGAAGATCGGTAC 237

|| | |||||| || ||||||| ||||| |||| | || | |||| | || |||||

Sbjct 105173 GGTCTGCGGCCTATCAGGTAGT-AGTGGGTGTAATGTACCTACTAGCCTACGACGGGTAC 105231

Query 238 GGGCCATGAGAGTGGGAGCCCGGAGATGGACACTGAGACACGGGTCCAGGCCCTACGGGG 297

||| |||||| ||||||||||||||| |||||||| | ||||||||||||||||

Sbjct 105232 GGGTTGTGAGAGCAAGAGCCCGGAGATGGATTCTGAGACATGAATCCAGGCCCTACGGGG 105291

Query 298 CGCAGCAGGCGCGAAACCTCCGCAATGCGGGCAACCGCGACGGGGGGACCCCCAGTGCC- 356

|||||||||||||||| || ||||||||| ||||| || |||||| || ||||||

Sbjct 105292 CGCAGCAGGCGCGAAAACTTTACAATGCGGGAAACCGTGATAAGGGGACACCGAGTGCCA 105351

Query 357 GTGGCA-ACGCCACGGCTTTTCCGGA-GTGTAAAAAGC-TCCGGGAATAAGGGCTGGGCA 413

| || | | ||| | ||| || |||||||| | || | | |||||| |||||

Sbjct 105352 GCATCATATG--TTGGC-TGTCCAGATGTGTAAAATACATCTGTTAGCAAGGGCCGGGCA 105408

Query 414 AGGCCGGTGGCAGCCGCCGCGGTAATACCGGCGGCCCGAGTGGTGGCCGCTATTATTGGG 473

|| |||||| ||||||||||||||| ||||||||||||||||||| || |||||||||

Sbjct 105409 AGACCGGTGCCAGCCGCCGCGGTAACACCGGCGGCCCGAGTGGTGATCGTGATTATTGGG 105468

Query 474 CCTAAAGCGTCCGTAGCCGGGCCCGTAAGTCC-CTGGCGAAATCCCACGGCTCAACCGTG 532

|||||| |||||||||||| || ||||| | || |||||| |||||||||||||

Sbjct 105469 TCTAAAGGGTCCGTAGCCGGTTTGGTCAGTCCTCCGG-GAAATCTGACGGCTCAACCGTT 105527

Query 533 GGGCTTGCTGGGGATACTGCGGGCCTTGGGACCGGGAGAGGCCGGGGGTACCCCTGGGGT 592

||||| | | ||||||||| | ||||| ||||||||||| | ||||| | |||||

Sbjct 105528 AGGCTTTCGGTGGATACTGCCAGACTTGGAACCGGGAGAGGTAAGAGGTACTACAGGGGT 105587

Query 593 AGGGGTGAAATCCTATAATCCCAGGGGGACCGCCAGTGGCGAAGGCGCCCGGCTGGAACG 652

||| |||||||| | ||||||| | | |||| || |||||||||||| | | |||||

Sbjct 105588 AGGAGTGAAATCTTGTAATCCCTGTGAGACCACCTGTGGCGAAGGCGTCTTACCAGAACG 105647

Query 653 GGTCCGACGGTGAGGGACGAAGGCCAGGGGAGCGAACCGGATTAGATACCCGGGTAGTCC 712

||| ||||||||||||||||| || |||| ||||||||||||||||||||||||||||

Sbjct 105648 GGTTCGACGGTGAGGGACGAAAGCTGGGGGCACGAACCGGATTAGATACCCGGGTAGTCC 105707

Query 713 TGGCTGTAAAGGATGCGGGCTAGGTGTCGGGCGAGCTTCGAGCTCGCCCGGTGCCGAAGG 772

|| ||||| ||||| |||||||||| ||| | ||| | | | ||||||| |||

Sbjct 105708 CAGCCGTAAACGATGCTCGCTAGGTGTCAGGCATGGCGCGACCGTGTCTGGTGCCGCAGG 105767

Query 773 GAAGCCGTTAAGCCCGCCGCCTGGGGAGTACGGCCGCAAGGCTGAAACTTAAAGGAATTG 832

|||||||| |||| ||| |||||| ||||||||||||||||||||||||||||||||||

Sbjct 105768 GAAGCCGTGAAGCGAGCCACCTGGGAAGTACGGCCGCAAGGCTGAAACTTAAAGGAATTG 105827

Query 833 GCGGGGGAGCACTACAAGGGGTGGAGCGTGCGGTTTAATTGGATTCAACGCCGGGAACCT 892

|||||||||||| |||| ||||||||| ||||||||||||||| |||||||||| | ||

Sbjct 105828 GCGGGGGAGCACAACAACGGGTGGAGCCTGCGGTTTAATTGGACTCAACGCCGGACAACT 105887

Query 893 CACCGGGGGCGACGGCAGGATGAAGGCCAGGCTGAAGGTCTTGCCGGACACGCCGAGAGG 952

|||||||| |||| ||| ||| |||||||||| ||| |||||| || ||| ||||||

Sbjct 105888 CACCGGGGACGACAGCAATATGTAGGCCAGGCTAAAGACCTTGCCTGAATCGCTGAGAGG 105947

Query 953 AGGTGCATGGCCGCCGTCAGCTCGTACCGTGAGGCGTCCACTTAAGTGTGGTAACGAGCG 1012

||||||||||||| || ||| |||||| |||| || ||| |||||| || ||||||||

Sbjct 105948 AGGTGCATGGCCGTCGCCAGTTCGTACTGTGAAGCATCCTGTTAAGTCAGGCAACGAGCG 106007

Query 1013 AGACCCGCGCCCCCCAGTTGCCAGTCCT-TCCCGCTGGGGAGGA-GGC-ACTCTGGGGGG 1069

||||||| |||| | ||| |||| | | ||| | ||| | || || |||||| ||||

Sbjct 106008 AGACCCGTGCCCACT-GTTACCAG-CATGTCCTCC-GGG-ACGATGGGTACTCTGTGGGG 106063

Query 1070 ACCGCCGGCGATAAGCCGGAGGAAGGAGCGGGCGACGGTAGGTCAGTATGCCCCGAAACC 1129

||||||| | ||| |||||||||| |||||| |||||||||||||||||||||||

Sbjct 106064 ACCGCCGATGTTAAATCGGAGGAAGGTGCGGGCTACGGTAGGTCAGTATGCCCCGAATTT 106123

Query 1130 CCCGGGCTACACGCGCGCTACAATGGGCGGGACAATGGGATCCGACCCCGAAAGGGGAAG 1189

||||||||||||||| ||||||||| ||||||||||| || ||||||| ||||| |

Sbjct 106124 CCCGGGCTACACGCGGGCTACAATGAATGGGACAATGGGTCCCTACCCCGAGAGGGGTTG 106183

Query 1190 GGAATCCCCTAAACCCGCCCCCAGTTCGGATCGCGGGCTGCAACTCGCCCGCGTGAAGCT 1249

| |||| || |||||| || ||||||||||| |||||| ||||||||| |||||||||

Sbjct 106184 GTAATCTCCCAAACCCATCCGTAGTTCGGATCGAGGGCTGTAACTCGCCCTCGTGAAGCT 106243

Query 1250 GGAATCCCTAGTACCCGCGTGTCATCATCGCGCGGCGAATACGTCCCTGCTCCTTGCACA 1309

||||||| ||||| ||||| ||| || |||||| ||||||||||||||||||||||||

Sbjct 106244 GGAATCCGTAGTAATCGCGTTTCAATATAGCGCGGTGAATACGTCCCTGCTCCTTGCACA 106303

Query 1310 CACCGCCCGTCA 1321

||||||||||||

Sbjct 106304 CACCGCCCGTCA 106315

Score = 935 bits (506), Expect = 0.0

Identities = 1057/1326 (80%), Gaps = 25/1326 (2%)

Strand=Plus/Minus

Query 7 CTAAGCCATGCGAGTCATGGGGCGCCTTGCGCGCAC-CGGCGGACGGCTCAGTAACACGT 65

|||||||||||||||||| | ||| || | || |||| || ||||||||||||||

Sbjct 2188410 CTAAGCCATGCGAGTCAT-ATG-TTCTT-CGTGAACATGGCGTACTGCTCAGTAACACGT 2188354

Query 66 CGGTAACCTACCCTCGGGAGGGGGATAACCCCGGGAAACTGGGGCTAATCCCCCATA-GG 124

| |||||| |||| |||| ||||||||||||||||||||||| |||| || ||| |

Sbjct 2188353 GGATAACCTGCCCTTGGGACCGGGATAACCCCGGGAAACTGGGGATAATACCGGATAACG 2188294

Query 125 CCTGAGGTACTGGAAGGTCCTCAGGCCGAAAGGGGCTCTGCCCGCCCGAGGATGGGCCGG 184

| | | | |||||| | | | | || ||| || | || | |||| |||||||| | |

Sbjct 2188293 CAT-ATCTGCTGGAATG-CTTTATGCGTAAAATGGATTTGTCTGCCCAAGGATGGGTCTG 2188236

Query 185 CGGCCGATTAGGTAGTTGGTGGG-GTAACGGCCCACCAAGCCGAAGATCGGTACGGGCCA 243

||||| || ||||||| ||||| |||| | || | |||| | || ||||||||

Sbjct 2188235 CGGCCTATCAGGTAGT-AGTGGGTGTAATGTACCTACTAGCCTACGACGGGTACGGGTTG 2188177

Query 244 TGAGAGTGGGAGCCCGGAGATGGACACTGAGACACGGGTCCAGGCCCTACGGGGCGCAGC 303

|||||| ||||||||||||||| |||||||| | ||||||||||||||||||||||

Sbjct 2188176 TGAGAGCAAGAGCCCGGAGATGGATTCTGAGACATGAATCCAGGCCCTACGGGGCGCAGC 2188117

Query 304 AGGCGCGAAACCTCCGCAATGCGGGCAACCGCGACGGGGGGACCCCCAGTGCC-GTGGCA 362

|||||||||| || ||||||||| ||||| || |||||| || |||||| | ||

Sbjct 2188116 AGGCGCGAAAACTTTACAATGCGGGAAACCGTGATAAGGGGACACCGAGTGCCAGCATCA 2188057

Query 363 -ACGCCACGGCTTTTCCGGA-GTGTAAAA-AGCTCCGGGAATAAGGGCTGGGCAAGGCCG 419

| | ||| | ||| || |||||||| | || | | |||||| ||||||| |||

Sbjct 2188056 TATG--TTGGC-TGTCCAGATGTGTAAAATACATCTGTTAGCAAGGGCCGGGCAAGACCG 2188000

Query 420 GTGGCAGCCGCCGCGGTAATACCGGCGGCCCGAGTGGTGGCCGCTATTATTGGGCCTAAA 479

||| ||||||||||||||| ||||||||||||||||||| || ||||||||| |||||

Sbjct 2187999 GTGCCAGCCGCCGCGGTAACACCGGCGGCCCGAGTGGTGATCGTGATTATTGGGTCTAAA 2187940

Query 480 GCGTCCGTAGCCGGGCCCGTAAGTCC-CTGGCGAAATCCCACGGCTCAACCGTGGGGCTT 538

| |||||||||||| || ||||| | || |||||| ||||||||||||| |||||

Sbjct 2187939 GGGTCCGTAGCCGGTTTGGTCAGTCCTCCGG-GAAATCTGACGGCTCAACCGTTAGGCTT 2187881

Query 539 GCTGGGGATACTGCGGGCCTTGGGACCGGGAGAGGCCGGGGGTACCCCTGGGGTAGGGGT 598

| | ||||||||| | ||||| ||||||||||| | ||||| | |||||||| ||

Sbjct 2187880 TCGGTGGATACTGCCAGACTTGGAACCGGGAGAGGTAAGAGGTACTACAGGGGTAGGAGT 2187821

Query 599 GAAATCCTATAATCCCAGGGGGACCGCCAGTGGCGAAGGCGCCCGGCTGGAACGGGTCCG 658

|||||| | ||||||| | | |||| || |||||||||||| | | |||||||| ||

Sbjct 2187820 GAAATCTTGTAATCCCTGTGAGACCACCTGTGGCGAAGGCGTCTTACCAGAACGGGTTCG 2187761

Query 659 ACGGTGAGGGACGAAGGCCAGGGGAGCGAACCGGATTAGATACCCGGGTAGTCCTGGCTG 718

||||||||||||||| || |||| |||||||||||||||||||||||||||| || |

Sbjct 2187760 ACGGTGAGGGACGAAAGCTGGGGGCACGAACCGGATTAGATACCCGGGTAGTCCCAGCCG 2187701

Query 719 TAAAGGATGCGGGCTAGGTGTCGGGCGAGCTTCGAGCTCGCCCGGTGCCGAAGGGAAGCC 778

|||| ||||| |||||||||| ||| | ||| | | | ||||||| |||||||||

Sbjct 2187700 TAAACGATGCTCGCTAGGTGTCAGGCATGGCGCGACCGTGTCTGGTGCCGCAGGGAAGCC 2187641

Query 779 GTTAAGCCCGCCGCCTGGGGAGTACGGCCGCAAGGCTGAAACTTAAAGGAATTGGCGGGG 838

|| |||| ||| |||||| ||||||||||||||||||||||||||||||||||||||||

Sbjct 2187640 GTGAAGCGAGCCACCTGGGAAGTACGGCCGCAAGGCTGAAACTTAAAGGAATTGGCGGGG 2187581

Query 839 GAGCACTACAAGGGGTGGAGCGTGCGGTTTAATTGGATTCAACGCCGGGAACCTCACCGG 898

|||||| |||| ||||||||| ||||||||||||||| |||||||||| | ||||||||

Sbjct 2187580 GAGCACAACAACGGGTGGAGCCTGCGGTTTAATTGGACTCAACGCCGGACAACTCACCGG 2187521

Query 899 GGGCGACGGCAGGATGAAGGCCAGGCTGAAGGTCTTGCCGGACACGCCGAGAGGAGGTGC 958

|| |||| ||| ||| |||||||||| ||| |||||| || ||| ||||||||||||

Sbjct 2187520 GGACGACAGCAATATGTAGGCCAGGCTAAAGACCTTGCCTGAATCGCTGAGAGGAGGTGC 2187461

Query 959 ATGGCCGCCGTCAGCTCGTACCGTGAGGCGTCCACTTAAGTGTGGTAACGAGCGAGACCC 1018

||||||| || ||| |||||| |||| || ||| |||||| || ||||||||||||||

Sbjct 2187460 ATGGCCGTCGCCAGTTCGTACTGTGAAGCATCCTGTTAAGTCAGGCAACGAGCGAGACCC 2187401

Query 1019 GCGCCCCCCAGTTGCCAGTCCT-TCCCGCTGGGGAGGA-GGC-ACTCTGGGGGGACCGCC 1075

| |||| | ||| |||| | | ||| | ||| | || || |||||| ||||||||||

Sbjct 2187400 GTGCCCACT-GTTACCAG-CATGTCCTCC-GGG-ACGATGGGTACTCTGTGGGGACCGCC 2187345

Query 1076 GGCGATAAGCCGGAGGAAGGAGCGGGCGACGGTAGGTCAGTATGCCCCGAAACCCCCGGG 1135

| | ||| |||||||||| |||||| ||||||||||||||||||||||| ||||||

Sbjct 2187344 GATGTTAAATCGGAGGAAGGTGCGGGCTACGGTAGGTCAGTATGCCCCGAATTTCCCGGG 2187285

Query 1136 CTACACGCGCGCTACAATGGGCGGGACAATGGGATCCGACCCCGAAAGGGGAAGGGAATC 1195

||||||||| ||||||||| ||||||||||| || ||||||| ||||| || ||||

Sbjct 2187284 CTACACGCGGGCTACAATGAATGGGACAATGGGTCCCTACCCCGAGAGGGGTTGGTAATC 2187225

Query 1196 CCCTAAACCCGCCCCCAGTTCGGATCGCGGGCTGCAACTCGCCCGCGTGAAGCTGGAATC 1255

|| |||||| || ||||||||||| |||||| ||||||||| |||||||||||||||

Sbjct 2187224 TCCCAAACCCATCCGTAGTTCGGATCGAGGGCTGTAACTCGCCCTCGTGAAGCTGGAATC 2187165

Query 1256 CCTAGTACCCGCGTGTCATCATCGCGCGGCGAATACGTCCCTGCTCCTTGCACACACCGC 1315

| ||||| ||||| ||| || |||||| ||||||||||||||||||||||||||||||

Sbjct 2187164 CGTAGTAATCGCGTTTCAATATAGCGCGGTGAATACGTCCCTGCTCCTTGCACACACCGC 2187105

Query 1316 CCGTCA 1321

||||||

Sbjct 2187104 CCGTCA 2187099

Score = 918 bits (497), Expect = 0.0

Identities = 1056/1328 (80%), Gaps = 29/1328 (2%)

Strand=Plus/Minus

Query 7 CTAAGCCATGCGAGTCATGGGGCGCCTTGCGCGCAC-CGGCGGACGGCTCAGTAACACGT 65

|||||||||||||||||| | ||| || | || |||| || ||||||||||||||

Sbjct 1525037 CTAAGCCATGCGAGTCAT-ATG-TTCTT-CGTGAACATGGCGTACTGCTCAGTAACACGT 1524981

Query 66 CGGTAACCTACCCTCGGGAGGGGGATAACCCCGGGAAACTGGGGCTAATCCCCCATA-GG 124

| |||||| |||| ||| ||||||||||||||||||||||| |||| || ||| |

Sbjct 1524980 GGATAACCTGCCCTTGGGTCTGGGATAACCCCGGGAAACTGGGGATAATACCGGATAACG 1524921

Query 125 CCTGAGGTACTGGAAGGTCCTCAGGCCG-AAAGGGGCTCTGCCCGCCCGAGGATGGGCCG 183

| | | | |||||| | | | | || ||| || || | | |||| |||||||| |

Sbjct 1524920 CAT-ATATGCTGGAATG-CTTTATGCGTAAAATGGATTC-GTCTGCCCAAGGATGGGTCT 1524864

Query 184 GCGGCCGATTAGGTAGTTGGTGGG-GTAACGGCCCACCAAGCCGAAGATCGGTACGGGCC 242

|||||| || ||||||| ||||| |||| | || | |||| | | ||||||||

Sbjct 1524863 GCGGCCTATCAGGTAGT-AGTGGGTGTAATGTACCTACTAGCCTACAACGGGTACGGGTT 1524805

Query 243 ATGAGAGTGGGAGCCCGGAGATGGACACTGAGACACGGGTCCAGGCCCTACGGGGCGCAG 302

|||||| ||||||||||||||| |||||||| | |||||||||||||||||||||

Sbjct 1524804 GTGAGAGCAAGAGCCCGGAGATGGATTCTGAGACATGAATCCAGGCCCTACGGGGCGCAG 1524745

Query 303 CAGGCGCGAAACCTCCGCAATGCGGGCAACCGCGACGGGGGGACCCCCAGTGCC-GTGGC 361

||||||||||| || ||||||||| ||||| || |||||| || |||||| | |

Sbjct 1524744 CAGGCGCGAAAACTTTACAATGCGGGAAACCGTGATAAGGGGACACCGAGTGCCAGCATC 1524685

Query 362 A-ACGCCACGGCTTTTCCGGA-GTGTAAAA-AGCTCCGGGAATAAGGGCTGGGCAAGGCC 418

| | | ||| | ||| || |||||||| | || | | |||||| ||||||| ||

Sbjct 1524684 ATATG--TTGGC-TGTCCAGATGTGTAAAATACATCTGTTAGCAAGGGCCGGGCAAGACC 1524628

Query 419 GGTGGCAGCCGCCGCGGTAATACCGGCGGCCCGAGTGGTGGCCGCTATTATTGGGCCTAA 478

|||| ||||||||||||||| ||||||||||||||||||| || ||||||||| ||||

Sbjct 1524627 GGTGCCAGCCGCCGCGGTAACACCGGCGGCCCGAGTGGTGATCGTGATTATTGGGTCTAA 1524568

Query 479 AGCGTCCGTAGCCGGGCCCGTAAGTCC-CTGGCGAAATCCCACGGCTCAACCGTGGGGCT 537

|| |||||||||||| || ||||| | || |||||| ||||||||||||| ||||

Sbjct 1524567 AGGGTCCGTAGCCGGTTTGGTCAGTCCTCCGG-GAAATCTGACGGCTCAACCGTTAGGCT 1524509

Query 538 TGCTGGGGATACTGCGGGCCTTGGGACCGGGAGAGGCCGGGGGTACCCCTGGGGTAGGGG 597

| | | ||||||||| | ||||| ||||||||||| | ||||| | |||||||| |

Sbjct 1524508 TTCGGTGGATACTGCCAGACTTGGAACCGGGAGAGGTAAGAGGTACTACAGGGGTAGGAG 1524449

Query 598 TGAAATCCTATAATCCCAGGGGGACCGCCAGTGGCGAAGGCGCCCGGCTGGAACGGGTCC 657

||||||| | ||||||| | | |||| || |||||||||||| | | |||||||| |

Sbjct 1524448 TGAAATCTTGTAATCCCTGTGAGACCACCTGTGGCGAAGGCGTCTTACCAGAACGGGTTC 1524389

Query 658 GACGGTGAGGGACGAAGGCCAGGGGAGCGAACCGGATTAGATACCCGGGTAGTCCTGGCT 717

|||||||||||||||| || |||| |||||||||||||||||||||||||||| ||

Sbjct 1524388 GACGGTGAGGGACGAAAGCTGGGGGCACGAACCGGATTAGATACCCGGGTAGTCCCAGCC 1524329

Query 718 GTAAAGGATGCGGGCTAGGTGTCGGGCGAGCTTCGAGCTCGCCCGGTGCCGAAGGGAAGC 777

||||| ||||| |||||||||| ||| | ||| | | | ||||||| ||||||||

Sbjct 1524328 GTAAACGATGCTCGCTAGGTGTCAGGCATGGCGCGACCGTGTCTGGTGCCGCAGGGAAGC 1524269

Query 778 CGTTAAGCCCGCCGCCTGGGGAGTACGGCCGCAAGGCTGAAACTTAAAGGAATTGGCGGG 837

||| |||| ||| |||||| |||||||||||||||||||||||||||||||||||||||

Sbjct 1524268 CGTGAAGCGAGCCACCTGGGAAGTACGGCCGCAAGGCTGAAACTTAAAGGAATTGGCGGG 1524209

Query 838 GGAGCACTACAAGGGGTGGAGCGTGCGGTTTAATTGGATTCAACGCCGGGAACCTCACCG 897

||||||| |||| ||||||||| ||||||||||||||| |||||||||| | |||||||

Sbjct 1524208 GGAGCACAACAACGGGTGGAGCCTGCGGTTTAATTGGACTCAACGCCGGACAACTCACCG 1524149

Query 898 GGGGCGACGGCAGGATGAAGGCCAGGCTGAAGGTCTTGCCGGACACGCCGAGAGGAGGTG 957

||| |||| ||| ||| |||||||||||||| |||||| || ||| |||||||||||

Sbjct 1524148 GGGACGACAGCAAAATGTAGGCCAGGCTGAAGACCTTGCCTGAATCGCTGAGAGGAGGTG 1524089

Query 958 CATGGCCGCCGTCAGCTCGTACCGTGAGGCGTCCACTTAAGTGTGGTAACGAGCGAGACC 1017

|||||||| || ||| |||||| |||| || ||| |||||| || |||||||||||||

Sbjct 1524088 CATGGCCGTCGCCAGTTCGTACTGTGAAGCATCCTGTTAAGTCAGGCAACGAGCGAGACC 1524029

Query 1018 CGCGCCCCCCAGTTGCCAGTCCT-TCCCGCTGGGGAGGA-GGC-ACTCTGGGGGGACCGC 1074

|| |||| | ||| |||| | | ||| | ||| | || || |||||| |||||||||

Sbjct 1524028 CGTGCCCACT-GTTACCAG-CATGTCCTCC-GGG-ACGATGGGTACTCTGTGGGGACCGC 1523973

Query 1075 CGGCGATAAGCCGGAGGAAGGAGCGGGCGACGGTAGGTCAGTATGCCCCGAAACCCCCGG 1134

|| | ||| |||||||||| |||||| ||||||||||||||||||||||| |||||

Sbjct 1523972 CGATGTTAAATCGGAGGAAGGTGCGGGCCACGGTAGGTCAGTATGCCCCGAATTTCCCGG 1523913

Query 1135 GCTACACGCGCGCTACAATGGGCGGGACAATGGGATCCGACCCC-GAAAGGGGAAGGGAA 1193

|||||||||| ||||||||| ||||||||||| ||| |||| |||| ||| || ||

Sbjct 1523912 GCTACACGCGGGCTACAATGAATGGGACAATGGG-TCCCTCCCCTGAAAAGGGCTGGTAA 1523854

Query 1194 TCCCCTAAACCCGCCCCCAGTTCGGATCGCGGGCTGCAACTCGCCCGCGTGAAGCTGGAA 1253

|| || |||||| || ||||||||||| |||||| ||||||||| |||||||||||||

Sbjct 1523853 TCTCCCAAACCCATCCGTAGTTCGGATCGAGGGCTGTAACTCGCCCTCGTGAAGCTGGAA 1523794

Query 1254 TCCCTAGTACCCGCGTGTCATCATCGCGCGGCGAATACGTCCCTGCTCCTTGCACACACC 1313

||| ||||| ||||| ||| || |||||| ||||||||||||||||||||||||||||

Sbjct 1523793 TCCGTAGTAATCGCGTTTCAATATAGCGCGGTGAATACGTCCCTGCTCCTTGCACACACC 1523734

Query 1314 GCCCGTCA 1321

||||||||

Sbjct 1523733 GCCCGTCA 1523726

> gi|383318453|ref|NC_017034.1| Methanocella conradii HZ254, complete

sequence

Length=2378438

Score = 787 bits (426), Expect = 0.0

Identities = 1037/1334 (78%), Gaps = 33/1334 (2%)

Strand=Plus/Plus

Query 1 GTCCGACTAAGCCATGCGAGTCATGGGGCGCCTTGCGCGCACCGGCGGACGGCTCAGTAA 60

|||||| ||||||||||||||| || || | || || | ||||| || |||||||||

Sbjct 514722 GTCCGATTAAGCCATGCGAGTCGTGAGGGG---TGAGC-CCTCGGCGTACTGCTCAGTAA 514777

Query 61 CACGTCGGTAACCTACCCTCGGGAGGGGGATAACCCCGGGAAACTGGGGCTAATCCCCCA 120

||||| | || || ||| | ||||||||||||||||||||||| |||| || |

Sbjct 514778 CACGTGGACAATCTGCCCAAAAGTCCGGGATAACCCCGGGAAACTGGGGATAATACCGGA 514837

Query 121 TAGGCC-TGAGGTACTGGAAGGTCCT-CAGGCCGAAAGGGGCTCTGCCCGCCCGAGGATG 178

|||||| | | |||||| | ||| || |||| | | | || |||||

Sbjct 514838 TAGGCCACCAATTGCTGGAATGGCCTGGTGGTTGAAACGAG---AG---GCTTTTGGATG 514891

Query 179 GGCCGGCGGCCGATTAGGTAGTTGGTGGGGTAACGGCCCACCAAGCC-GAAGATCGGTAC 237

|| | ||||| |||||||| | | || |||||| || | ||| | | ||| ||||

Sbjct 514892 GGTCTGCGGCGGATTAGGTTGACGCCGGTGTAACGTACCGGCGTGCCTGTA-ATCCGTAC 514950

Query 238 GGGCCATGAGAGTGGGAGCCCGGAGATGGACACTGAGACACGGGTCCAGGCCCTACGGGG 297

||| || ||| ||||||||||||||| |||||||||| ||||||||||||||||

Sbjct 514951 GGGTTGTGGGAGCAAGAGCCCGGAGATGGATTCTGAGACACGAATCCAGGCCCTACGGGG 515010

Query 298 CGCAGCAGGCGCGAAACCTCCGCAATGCGGGCAA-CCGCGACGGGGGGACCCCCAGTGCC 356

|||||||||||||||| ||| |||||| ||||| | |||| ||||||| | |||| |

Sbjct 515011 CGCAGCAGGCGCGAAAACTCTACAATGCAGGCAATCTGCGATAGGGGGACATCGAGTGGC 515070

Query 357 GTGGCAACGCCACG-GCTTTTCCGGA-GTGT-AAAAA-GCTCCGGGAATAAGGGCTGGGC 412

| | | | | || | ||| || | ||||| | | | | |||||| |||

Sbjct 515071 AT--CTTCTTAAGGTGCCTGTCCAACCGTCTAAAAAACGGT-TGTTAGCAAGGGCCGGGT 515127

Query 413 AAGGCCGGTGGCAGCCGCCGCGGTAATACCGGCGGCCCGAGTGGTGGCCGCTATTATTGG 472

||| |||||| ||||||||||||||||||||||||| ||||||||||||| ||||||||

Sbjct 515128 AAGACCGGTGCCAGCCGCCGCGGTAATACCGGCGGCTCGAGTGGTGGCCGATATTATTGA 515187

Query 473 GCCTAAAGCGTCCGTAGCCGGGCCCGTAAGT-CCCTGGCGAAATCCCACGGCTCAACCGT 531

| |||||| |||||||||||| | |||| |||||| ||||||| ||||| ||||||

Sbjct 515188 GTCTAAAGGGTCCGTAGCCGGCTTTGCAAGTCCCCTGG-GAAATCCAGCGGCTTAACCGT 515246

Query 532 GGGGCTTGCTGGGGATACTGCGGGCCTTGGGACCGGGAGAGGCCGGGGGTACCCCTGGGG 591

|||| | |||||||| | |||||||| ||||||||| | ||||| | ||||

Sbjct 515247 TGGGCGCCCATGGGATACTACATTGCTTGGGACTGGGAGAGGCGAGAGGTACTCGAGGGG 515306

Query 592 TAGGGGTGAAATCCTATAATCCCAGGGGGACCGCCAGTGGCGAAGGCGCCCGGCTGGAAC 651

||||||||||||||| |||||| ||||||| || |||||||||||| | || ||||

Sbjct 515307 TAGGGGTGAAATCCTGTAATCCTTCGGGGACCACCGGTGGCGAAGGCGTCTCGCCAGAAC 515366

Query 652 GGGTCCGACGGTGAGGGACGAAGGCCAGGGGAGCGAACCGGATTAGATACCCGGGTAGTC 711

||||||||||||||||||||| || ||||| |||||||||||||||||||||||||||

Sbjct 515367 AGGTCCGACGGTGAGGGACGAAAGCTAGGGGCACGAACCGGATTAGATACCCGGGTAGTC 515426

Query 712 CTGGCTGTAAAGGATGCGGGCTAGGTGTCGGGCGAGCTTCGAGC-TCGCCC-GGTGCCGA 769

|| || ||||| ||||| |||||||||| ||| ||| | | | |||||||

Sbjct 515427 CTAGCCGTAAACGATGCCCGCTAGGTGTCA--CGATAATCGTGAATTATCGTGGTGCCGT 515484

Query 770 AGGGAAGCCGTTAAGCCCGCCGCCTGGGGAGTACGGCCGCAAGGCTGAAACTTAAAGGAA 829

|||||||||| |||| ||| | |||| |||||| |||||||| |||||||||||||||

Sbjct 515485 AGGGAAGCCGCGAAGCGGGCCACTTGGGAAGTACGACCGCAAGGTTGAAACTTAAAGGAA 515544

Query 830 TTGGCGGGGGAGCACTACAAGGGGTGGAGCGTGCGGTTTAATTGGATTCAACGCCGGGAA 889

||||||||||||||| |||| ||||||||| |||||||||||||||||||||||||||||

Sbjct 515545 TTGGCGGGGGAGCACCACAACGGGTGGAGCCTGCGGTTTAATTGGATTCAACGCCGGGAA 515604

Query 890 CCTCACCGGGGGCGACGGCAGGATGAAGGCCAGGCTGAAGGTCTTGCCGGACACGCCGAG 949

|| |||||| |||| | | ||||||||||||| |||| |||||||||| || |||

Sbjct 515605 GCTTACCGGGATCGACAGTTGAATGAAGGCCAGGCCGAAGACCTTGCCGGACTAGCTGAG 515664

Query 950 AGGAGGTGCATGGCCGCCGTCAGCTCGTACCGTGAGGCGTCCACTTAAGTGTGGTAACGA 1009

|||||||||||||||| |||||| |||||||||||||||||| |||||| || |||||

Sbjct 515665 AGGAGGTGCATGGCCGTCGTCAGTTCGTACCGTGAGGCGTCCTGTTAAGTCAGGCAACGA 515724

Query 1010 GCGAGACCCGCGCCCCCCAGTTGCCAGTCCTTCCCGCTGGGGA-GGAG-GCACTCTGGGG 1067

||||||||| | || | ||||| | | ||| || | ||| || | | || ||| ||

Sbjct 515725 GCGAGACCCATGTCCACT-GTTGCTAACGCGTCC-GC-GAGGACGGCGAGTACACTGTGG 515781

Query 1068 GGACCGCCGGCGATAAGCCGGAGGAAGGAGCGGGCGACGGTAGGTCAGTATGCCCCGAAA 1127

||| || |||| |||| | |||||||| || |||||||||||||||||||||||||

Sbjct 515782 AGACTGCTGGCGCTAAGTCAGAGGAAGGGTTGGTCGACGGTAGGTCAGTATGCCCCGAAT 515841

Query 1128 CCCCCGGGCTACACGCGCGCTACAATGGGCGGGACAATGGGATCCGACCCCGAAAGGGGA 1187

||||||||||||||| |||||||||| | |||||||||| | || |||| ||| ||

Sbjct 515842 ATCCCGGGCTACACGCGGGCTACAATGGACAGGACAATGGGTAACAACACCGAGAGGTGA 515901

Query 1188 AGGGAATCCCCTAAACCCGCCCCCAGTTCGGATCGCGGGCTGCAACTCGCCCGCGTGAAG 1247

|| |||| | |||||| | ||| ||||||||| | |||||| ||| ||||| | |||||

Sbjct 515902 AGTTAATCTCTTAAACCTGTCCCTAGTTCGGATTGAGGGCTGAAACCCGCCCTCATGAAG 515961

Query 1248 CTGGAATCCCTAGTACCCGCGTGTCATCATCGCGCGGCGAATACGTCCCTGCTCCTTGCA 1307

|||||||| ||||| ||| | ||| | | |||| ||||||||||||||||||||||

Sbjct 515962 ATGGAATCCGTAGTAATCGCATTTCAAAACAGTGCGGTGAATACGTCCCTGCTCCTTGCA 516021

Query 1308 CACACCGCCCGTCA 1321

||||||||||||||

Sbjct 516022 CACACCGCCCGTCA 516035

Score = 787 bits (426), Expect = 0.0

Identities = 1037/1334 (78%), Gaps = 33/1334 (2%)

Strand=Plus/Minus

Query 1 GTCCGACTAAGCCATGCGAGTCATGGGGCGCCTTGCGCGCACCGGCGGACGGCTCAGTAA 60

|||||| ||||||||||||||| || || | || || | ||||| || |||||||||

Sbjct 1679216 GTCCGATTAAGCCATGCGAGTCGTGAGGGG---TGAGC-CCTCGGCGTACTGCTCAGTAA 1679161

Query 61 CACGTCGGTAACCTACCCTCGGGAGGGGGATAACCCCGGGAAACTGGGGCTAATCCCCCA 120

||||| | || || ||| | ||||||||||||||||||||||| |||| || |

Sbjct 1679160 CACGTGGACAATCTGCCCAAAAGTCCGGGATAACCCCGGGAAACTGGGGATAATACCGGA 1679101

Query 121 TAGGCC-TGAGGTACTGGAAGGTCCT-CAGGCCGAAAGGGGCTCTGCCCGCCCGAGGATG 178

|||||| | | |||||| | ||| || |||| | | | || |||||

Sbjct 1679100 TAGGCCACCAATTGCTGGAATGGCCTGGTGGTTGAAACGAG---AG---GCTTTTGGATG 1679047

Query 179 GGCCGGCGGCCGATTAGGTAGTTGGTGGGGTAACGGCCCACCAAGCC-GAAGATCGGTAC 237

|| | ||||| |||||||| | | || |||||| || | ||| | | ||| ||||

Sbjct 1679046 GGTCTGCGGCGGATTAGGTTGACGCCGGTGTAACGTACCGGCGTGCCTGTA-ATCCGTAC 1678988

Query 238 GGGCCATGAGAGTGGGAGCCCGGAGATGGACACTGAGACACGGGTCCAGGCCCTACGGGG 297

||| || ||| ||||||||||||||| |||||||||| ||||||||||||||||

Sbjct 1678987 GGGTTGTGGGAGCAAGAGCCCGGAGATGGATTCTGAGACACGAATCCAGGCCCTACGGGG 1678928

Query 298 CGCAGCAGGCGCGAAACCTCCGCAATGCGGGCAA-CCGCGACGGGGGGACCCCCAGTGCC 356

|||||||||||||||| ||| |||||| ||||| | |||| ||||||| | |||| |

Sbjct 1678927 CGCAGCAGGCGCGAAAACTCTACAATGCAGGCAATCTGCGATAGGGGGACATCGAGTGGC 1678868

Query 357 GTGGCAACGCCACG-GCTTTTCCGGA-GTGT-AAAAA-GCTCCGGGAATAAGGGCTGGGC 412

| | | | | || | ||| || | ||||| | | | | |||||| |||

Sbjct 1678867 AT--CTTCTTAAGGTGCCTGTCCAACCGTCTAAAAAACGGT-TGTTAGCAAGGGCCGGGT 1678811

Query 413 AAGGCCGGTGGCAGCCGCCGCGGTAATACCGGCGGCCCGAGTGGTGGCCGCTATTATTGG 472

||| |||||| ||||||||||||||||||||||||| ||||||||||||| ||||||||

Sbjct 1678810 AAGACCGGTGCCAGCCGCCGCGGTAATACCGGCGGCTCGAGTGGTGGCCGATATTATTGA 1678751

Query 473 GCCTAAAGCGTCCGTAGCCGGGCCCGTAAGT-CCCTGGCGAAATCCCACGGCTCAACCGT 531

| |||||| |||||||||||| | |||| |||||| ||||||| ||||| ||||||

Sbjct 1678750 GTCTAAAGGGTCCGTAGCCGGCTTTGCAAGTCCCCTGG-GAAATCCAGCGGCTTAACCGT 1678692

Query 532 GGGGCTTGCTGGGGATACTGCGGGCCTTGGGACCGGGAGAGGCCGGGGGTACCCCTGGGG 591

|||| | |||||||| | |||||||| ||||||||| | ||||| | ||||

Sbjct 1678691 TGGGCGCCCATGGGATACTACATTGCTTGGGACTGGGAGAGGCGAGAGGTACTCGAGGGG 1678632

Query 592 TAGGGGTGAAATCCTATAATCCCAGGGGGACCGCCAGTGGCGAAGGCGCCCGGCTGGAAC 651

||||||||||||||| |||||| ||||||| || |||||||||||| | || ||||

Sbjct 1678631 TAGGGGTGAAATCCTGTAATCCTTCGGGGACCACCGGTGGCGAAGGCGTCTCGCCAGAAC 1678572

Query 652 GGGTCCGACGGTGAGGGACGAAGGCCAGGGGAGCGAACCGGATTAGATACCCGGGTAGTC 711

||||||||||||||||||||| || ||||| |||||||||||||||||||||||||||

Sbjct 1678571 AGGTCCGACGGTGAGGGACGAAAGCTAGGGGCACGAACCGGATTAGATACCCGGGTAGTC 1678512

Query 712 CTGGCTGTAAAGGATGCGGGCTAGGTGTCGGGCGAGCTTCGAGC-TCGCCC-GGTGCCGA 769

|| || ||||| ||||| |||||||||| ||| ||| | | | |||||||

Sbjct 1678511 CTAGCCGTAAACGATGCCCGCTAGGTGTCA--CGATAATCGTGAATTATCGTGGTGCCGT 1678454

Query 770 AGGGAAGCCGTTAAGCCCGCCGCCTGGGGAGTACGGCCGCAAGGCTGAAACTTAAAGGAA 829

|||||||||| |||| ||| | |||| |||||| |||||||| |||||||||||||||

Sbjct 1678453 AGGGAAGCCGCGAAGCGGGCCACTTGGGAAGTACGACCGCAAGGTTGAAACTTAAAGGAA 1678394

Query 830 TTGGCGGGGGAGCACTACAAGGGGTGGAGCGTGCGGTTTAATTGGATTCAACGCCGGGAA 889

||||||||||||||| |||| ||||||||| |||||||||||||||||||||||||||||

Sbjct 1678393 TTGGCGGGGGAGCACCACAACGGGTGGAGCCTGCGGTTTAATTGGATTCAACGCCGGGAA 1678334

Query 890 CCTCACCGGGGGCGACGGCAGGATGAAGGCCAGGCTGAAGGTCTTGCCGGACACGCCGAG 949

|| |||||| |||| | | ||||||||||||| |||| |||||||||| || |||

Sbjct 1678333 GCTTACCGGGATCGACAGTTGAATGAAGGCCAGGCCGAAGACCTTGCCGGACTAGCTGAG 1678274

Query 950 AGGAGGTGCATGGCCGCCGTCAGCTCGTACCGTGAGGCGTCCACTTAAGTGTGGTAACGA 1009

|||||||||||||||| |||||| |||||||||||||||||| |||||| || |||||

Sbjct 1678273 AGGAGGTGCATGGCCGTCGTCAGTTCGTACCGTGAGGCGTCCTGTTAAGTCAGGCAACGA 1678214

Query 1010 GCGAGACCCGCGCCCCCCAGTTGCCAGTCCTTCCCGCTGGGGA-GGAG-GCACTCTGGGG 1067

||||||||| | || | ||||| | | ||| || | ||| || | | || ||| ||

Sbjct 1678213 GCGAGACCCATGTCCACT-GTTGCTAACGCGTCC-GC-GAGGACGGCGAGTACACTGTGG 1678157

Query 1068 GGACCGCCGGCGATAAGCCGGAGGAAGGAGCGGGCGACGGTAGGTCAGTATGCCCCGAAA 1127

||| || |||| |||| | |||||||| || |||||||||||||||||||||||||

Sbjct 1678156 AGACTGCTGGCGCTAAGTCAGAGGAAGGGTTGGTCGACGGTAGGTCAGTATGCCCCGAAT 1678097

Query 1128 CCCCCGGGCTACACGCGCGCTACAATGGGCGGGACAATGGGATCCGACCCCGAAAGGGGA 1187

||||||||||||||| |||||||||| | |||||||||| | || |||| ||| ||

Sbjct 1678096 ATCCCGGGCTACACGCGGGCTACAATGGACAGGACAATGGGTAACAACACCGAGAGGTGA 1678037

Query 1188 AGGGAATCCCCTAAACCCGCCCCCAGTTCGGATCGCGGGCTGCAACTCGCCCGCGTGAAG 1247

|| |||| | |||||| | ||| ||||||||| | |||||| ||| ||||| | |||||

Sbjct 1678036 AGTTAATCTCTTAAACCTGTCCCTAGTTCGGATTGAGGGCTGAAACCCGCCCTCATGAAG 1677977

Query 1248 CTGGAATCCCTAGTACCCGCGTGTCATCATCGCGCGGCGAATACGTCCCTGCTCCTTGCA 1307

|||||||| ||||| ||| | ||| | | |||| ||||||||||||||||||||||

Sbjct 1677976 ATGGAATCCGTAGTAATCGCATTTCAAAACAGTGCGGTGAATACGTCCCTGCTCCTTGCA 1677917

Query 1308 CACACCGCCCGTCA 1321

||||||||||||||

Sbjct 1677916 CACACCGCCCGTCA 1677903

Lambda K H

1.33 0.621 1.12

Gapped

Lambda K H

1.28 0.460 0.850

Effective search space used: 78273543845

Database: User specified sequence set (Input: Merged_archaea.fasta).

Posted date: Unknown

Number of letters in database: 60,443,697

Number of sequences in database: 31

Matrix: blastn matrix 1 -2

Gap Penalties: Existence: 0, Extension: 2.5
