# Supplementary material for: Using custom-built primers and nanopore sequencing to evaluate CO-utilizer bacterial and archaeal populations linked to bioH2 production
Source: Sci Rep. 2023 Oct 9;13:17025. doi: 10.1038/s41598-023-44357-3 (PMC10562470; doi:10.1038/s41598-023-44357-3)
Supplement: Supplementary file 2 — Supplementary Information 2. [file 41598_2023_44357_MOESM2_ESM.docx]

filename="Merged_archaea.fasta"

file= open(filename,"r")

sequences={}

active_seq=""

active_def=""

def assignSeq(active_def, active_seq):

if active_seq != "":

sequences.update({active_def: active_seq})

for line in file:

if line.startswith(">"):

assignSeq(active_def, active_seq)

active_seq=""

active_def="|".join(line[1:].split("|")[:-1])+"|"

# active_def="|".join(["1","2","3"])

# active_def=line[1:-1]

print(active_def)

else:

active_seq+=line[:-1]

assignSeq(active_def, active_seq)

tsvfilename="output.tsv"

tsvfile=open(tsvfilename,"r")

alignments={}

for line in tsvfile:

if not line.startswith("#"):

temp=line.split("\t")

alignments.update({ temp[1]: sorted( [int(temp[8]),int(temp[9])]) })

print(alignments)

for seq_def in sequences:

sequences[seq_def]= sequences[seq_def][ alignments[seq_def][0] : alignments[seq_def][1] ]

outputfilename="16Ssequences.fasta"

outputfile=open(outputfilename,"w")

for seq_def in sequences:

outputfile.write(">"+seq_def+"\n")

outputfile.write(sequences[seq_def]+"\n")
